# Acid‐ and Nucleophile‐Gated Photoisomerization of Phosphaindirubin

**DOI:** 10.1002/anie.202519686

**Published:** 2025-11-30

**Authors:** Jacob Jan van der Wal, Jorn D. Steen, Ann‐Kathrin Rückert, Roman Yu. Peshkov, Michiel F. Hilbers, P. Tim Prins, Wybren Jan Buma, Andreas Orthaber, Stefano Crespi

**Affiliations:** ^1^ Department of Chemistry – Ångström Laboratory Uppsala University Box 523 Uppsala 751 20 Sweden; ^2^ Department of Chemistry Humboldt‐Universität Brook‐Taylor‐Str. 2 12489 Berlin Germany; ^3^ Van 't Hoff Institute for Molecular Sciences University of Amsterdam Science Park 904 Amsterdam 1098 XH The Netherlands; ^4^ Institute for Molecules and Materials FELIX Laboratory Radboud University Toernooiveld 7c Nijmegen 6525 ED The Netherlands

**Keywords:** Acidity, Nucleophile, Phosphorus heterocycles, Photoisomerization, Solvent effects

## Abstract

Nature uses protonation and microenvironmental effects to modulate photoisomerization, as seen in rhodopsins and GFP. Inspired by this, we report phosphaindirubin (PI), a visible‐light responsive photoswitch bearing a stereogenic phosphorus center that exhibits reversible *Z*/*E* isomerization controlled by light, acid, and nucleophiles. While structurally related to photoinert isoindigo, phosphaindirubin undergoes efficient *Z*→*E* isomerization in low‐polarity solvents but remains inert in polar media unless protonated. Acid gating alters the excited‐state landscape, enabling switching under light irradiation in acetonitrile. Strikingly, the thermal back‐isomerization of PI is accelerated by nucleophiles, including pyridine and iodide, offering an underexplored mechanism for catalyzing double bond rotation. This triple‐responsiveness to light, acid, and nucleophile enables reversible, fatigue‐resistant cycling between *Z*‐ and *E*‐forms. These findings introduce a new design principle for photoswitches based on dynamic, multi‐stimuli gating of excited‐state and ground‐state reactivity.

## Graphical abstract

**Triply gated isomerization**: In polar solvents, a phosphorus‐containing indirubin photoswitch undergoes visible‐light‐driven Z→E isomerization only when protonated, and reverts thermally via nucleophile‐catalyzed back‐isomerization. This three‐way control by light, acid, and nucleophile enables reversible photoisomerization.

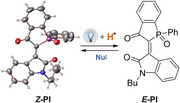

## Introduction

**Figure 1:**
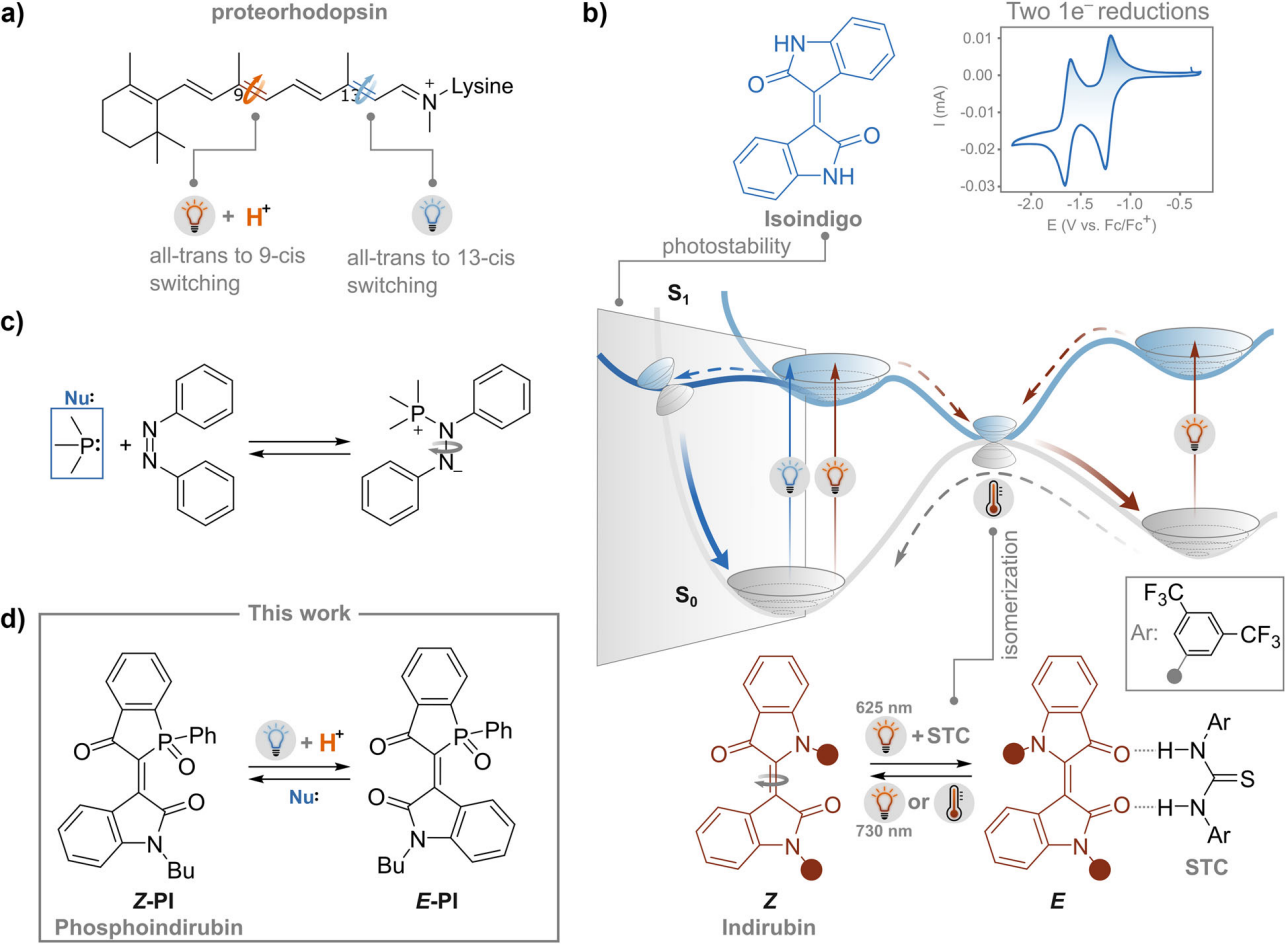
a) In proteorhodopsin, photoisomerization of retinal is selectively gated by protonation, enabling light‐controlled switching between *trans* and 9‐*cis* or 13‐*cis* configurations, depending on the protonation state of the Schiff base. b) Isoindigo (blue) undergoes two reversible reductions but is photochemically inert due to ultrafast internal conversion. Indirubin (red), a closely related indigoid, undergoes efficient red‐light‐induced *Z*/*E* isomerization that can be modulated by the presence of Schreiner's thiourea catalyst (STC). The substituents on the nitrogen atoms of indirubin are alkyl chains. c) Nucleophile‐induced isomerization of azobenzene‐phosphine adducts exemplifies the role of Lewis bases in facilitating double bond rotation. d) This work: phosphaindirubin (**PI**), a photoswitchable indirubin analogue bearing a stereogenic phosphorus center. Light‐induced *Z*→*E* isomerization is gated by protonation, and the thermal back‐reaction can be catalyzed by weak nucleophiles, enabling a photoresponsive system with integrated chemical control.

Nature has evolved a remarkable control over photochemical processes, as exemplified by systems like rhodopsins and green fluorescent proteins (GFP), where the surrounding environment modulates light‐driven isomerization events.^[^ ^1^, ^2^, ^3^ ^]^ In proteorhodopsins, for instance, the presence of a protonated Schiff base and the pH‐dependent dynamics of the chromophore directly influence its switching behavior: protonation biases the photoisomerism between the double bond in position 9 or 13 in the unsaturated chain (Figure 1a).^[^ ^2^ ^]^ Similarly, the excited‐state reactivity and photophysics of GFP and GFP‐like chromophores are fine‐tuned by the local hydrogen‐bonding network, demonstrating how protonation and polarity can gate or suppress excited‐state pathways.^[^ ^4^, ^5^ ^]^ These natural systems highlight the broader principle that external stimuli, such as protons and solvent polarity, can exert subtle yet profound effects on photoisomerization. Inspired by these examples, chemists have long pursued molecular photoswitches that can be externally controlled, enabling light‐triggered, reversible changes in configuration for applications in photobiology,^[^ ^6^ ^]^ catalysis,^[^ ^7^, ^8^ ^]^ smart materials,^[^ ^9^, ^10^ ^]^ and molecular machines,^[^ ^11^, ^12^ ^]^ to name a few.

Recently, a supramolecular approach^[^ ^13^ ^]^ was used to control the quantitative switching of azobenzene via energy transfer,^[^ ^14^ ^]^ the excited state lifetime of an iminothioindoxyl switch in metal‐organic cages,^[^ ^15^ ^]^ and the switching of a spiropyran in a MOF,^[^ ^16^ ^]^ as well as bias the directionality in a molecular motor via non covalent interactions,^[^ ^17^ ^]^ and modulate the red shift of the absorption of the photoswitch indirubin via interaction with Schreiner's thiourea catalyst.^[^ ^18^ ^]^

Indirubin belongs to the prominent family of indigoid photoswitches isomerizing about a C═C bond, which includes indigo,^[^ ^19^, ^20^ ^]^ thioindigo,^[^ ^21^ ^]^ hemiindigo,^[^ ^22^, ^23^, ^24^ ^]^ and hemithioindigo.^[^ ^25^, ^26^, ^27^ ^]^ These switches usually exhibit robust switching, visible‐light absorption and long‐lived metastable states.^[^ ^28^, ^29^ ^]^ Structural modifications allow tuning of both thermal and photochemical pathways,^[^ ^19^, ^30^, ^31^ ^]^ while chiral versions and oxindole‐fused derivatives have expanded the scope to unidirectional motion and complex systems.^[^ ^6^, ^32^, ^33^, ^34^ ^]^ Phosphorus‐based modifications of indigoid scaffolds remain largely underexplored, despite phosphorus offering unique advantages, including a stereogenic P(V) center for chirality and versatile post‐functionalization options. Recently, the Dube group introduced the first water‐soluble chiral phosphoindigoid switches,^[^ ^35^ ^]^ marking a step toward bio‐compatible and modular systems. Building on this foundation, we synthesized the phosphorus derivative of indirubin, dubbed phosphaindirubin (**PI**), which incorporates an oxindole moiety and a benzophospholan‐3‐one core.

We discovered that **PI** is structurally reminiscent of indirubin, and electronically of isoindigo, which is known for its lack of photoisomerism and two reversible one‐electron reductions.^[^ ^36^ ^]^ The photostability of isoindigo originates from a rapid internal conversion to the ground state upon excitation (Figure 1b), impeding any C═C isomerism.^[^ ^37^ ^]^ **PI** retains the reversible electron transfer features of isoindigo in polar solvents, yet diverges profoundly in its photoisomerization dynamics. While isoindigo is limited by rapid excited‐state deactivation, our phosphaindirubin analogue reveals an unexpected level of environmental sensitivity. Indeed, we find that light‐induced switching can be selectively gated by acid and reversed through nucleophilic catalysis, offering fine control over both forward photoisomerization and thermal back‐isomerization. While strong nucleophilic catalysts such as phosphines or electrophiles such as Pd(II) can promote C═C, C═N, and N═N double bond isomerization (Figure 1c),^[^ ^38^, ^39^, ^40^ ^]^ this approach is rarely utilized with weaker nucleophiles or as part of a switching system. In this work, we present a three‐way responsiveness to light, acid and nucleophiles that enables the cyclical operation of phosphaindirubin (Figure 1d), and which suggests a design principle where subtle modifications to the excited and ground state landscapes, mediated by protonation, solvent polarity, and nucleophilic environment, can unlock new photochemical behavior in indigoids and photoswitchable systems.

## Results and Discussion

**Figure 2:**
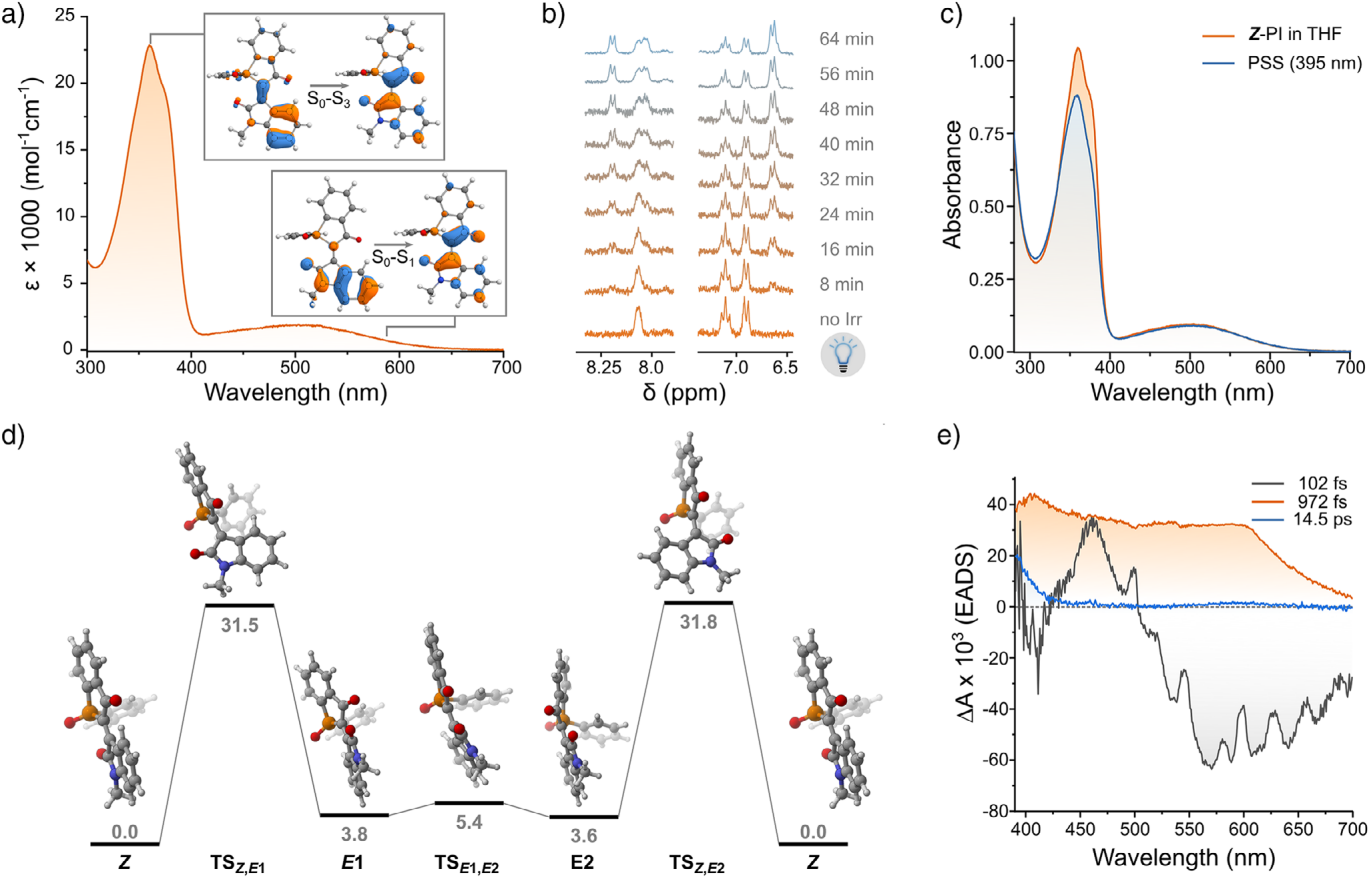
a) UV–vis absorption spectrum of ***Z*‐PI** in MeCN, showing two bright π→π* transitions (S_0_→S_1_ and S_0_→S_3_). Insets show the natural transition orbitals for each transition obtained at the M06‐2X/def2‐TZVPP//r^2^SCAN‐3c level using CPCM(MeCN) as implicit solvent model. b) Time‐resolved ^1^H NMR spectra of ***Z*‐PI** under 395 nm irradiation in THF‐d_8_ at −25 °C, showing *Z*→*E* isomerization. c) UV–vis absorption spectra of ***Z*‐PI** in THF before (orange) and after irradiation at 395 nm (blue). d) Computed ground‐state energy profile for *Z*/*E* interconversion of **PI** at the ωB97X‐V/def2‐TZVPP//r^2^SCAN‐3c level of theory using CPCM(MeCN) as implicit solvent model. All energy differences are reported in kcal·mol^−1^. e) Ultrafast transient absorption spectra of ***Z*‐PI** in MeCN following 375 nm excitation, showing the evolution associated difference spectra (EADS) attributed to the S_3_ excited singlet state (*τ*=102 fs) evolving to the S_1_ (*τ*=972 fs) and finally to a hot‐ground state relaxing back to ***Z*‐PI** (*τ*=14.5 ps).

We synthesized phosphaindirubin via a one‐pot Knoevenagel condensation of *n*‐butylisatin and 1‐phenylbenzophospholan‐3‐one oxide using piperidine as a catalyst.^[^ ^41^, ^42^ ^]^ This reaction selectively afforded the *Z*‐isomer (***Z*‐PI**) as a purple solid in 61% yield. Using DFT and mixed‐reference spin‐flip time‐dependent DFT (MRSF‐TDDFT) calculations with different functionals and basis sets, we confirmed that the *Z‐*isomer is thermodynamically more stable than the *E*‐isomer (Figures 2d and S20). We also prepared the enantioenriched (*R*,*Z*)‐phosphaindirubin, achieving up to 85% enantiomeric excess (Figure S12). By slow evaporation of a 1:1 water‐acetonitrile solution of ***Z*‐PI**, we obtained single crystals suitable for X‐ray diffraction. The structure revealed a C═C bond length of 1.360(3) Å and a C(O)‐N bond length of 1.362(2) Å, which are typical for oxindole‐based photoswitches and indirubin (see Figures 1d and S16).^[^ ^18^, ^43^, ^44^ ^]^ These experimental values closely match those obtained from calculations at the r^2^SCAN‐3c (1.360 and 1.365 Å) and MRSF‐TDDFT‐BH&HLYP (1.356 and 1.360 Å) levels of theory.

We then investigated the photophysical properties of **PI** in solvents of varying polarity and proticity, including toluene, acetonitrile, and methanol (Figures 2a and S13). In all cases, the absorption spectrum displayed two main π→π* transitions whose nature was confirmed by TDDFT calculations (see Section 8.5.1 in the Supporting Information). The low‐energy, broad S_0_→S_1_ band centers at 520 nm tailing up to ∼650 nm, with molar absorptivities ranging from 1.8 to 2.0 × 10^3^ M^−1^·cm^−1^. This transition features partial charge transfer from the oxindole to the benzophospholane moiety, resembling the first excited state observed in isoindigo derivatives (Figures 2a and S36).^[^ ^36^ ^]^ We observed a second, higher‐energy transition (S_0_→S_3_) centered near 360 nm, with a higher molar absorptivity (2.0–2.3 × 10^4^ M^−1^·cm^−1^). This band is localized on the C═C bond and exhibits minimal solvatochromism.

To investigate photoisomerization, we recorded NMR spectra of ***Z*‐PI** in THF‐*d*_8_ at −25 °C during in situ irradiation with a 395 nm LED. Over time, we observed new signals appearing in both the ^1^H and ^31^P NMR spectra, consistent with the formation of the *E*‐isomer (Figures 2b, S86, and S87). At the photostationary state (PSS), we measured a *Z*/*E* ratio of 51:49 in the ^1^H‐NMR spectrum. Upon warming the sample to 25 °C, the *E*‐isomer thermally relaxed back to ***Z*‐PI** (Figure S88). UV–vis absorption spectra recorded during irradiation at 365 or 395 nm showed a slight bathochromic shift in the S_0_→S_3_ band and two isosbestic points at 338 and 550 nm (Figures 2c and S50), consistent with a unimolecular switching process. We observed no isomerization upon irradiation at 505 nm, indicating wavelength‐specific activation and anti‐Kasha behavior.

DFT calculations revealed two distinct metastable *E*‐isomers, which differ in the helical twist of the oxindole carbonyls (Figure 2d). These forms interconvert through a low energy barrier (∼2 kcal·mol^−1^) and return to the *Z*‐isomer via separate thermal pathways with similar activation barriers (∼28 kcal·mol^−1^).

To better understand the influence of solvent polarity on the photochemical behavior of **PI**, we explored toluene, a solvent less polar than THF. Unfortunately, we also observed photodegradation upon prolonged irradiation (Figures S48 and S49). By sparging the solution with argon beforehand, we significantly reduced or delayed this decomposition, suggesting that oxygen participates in a photo‐oxidation process, suggesting the intermediacy of reactive triplet states, whose presence in indigoids is known and was explored thoroughly, for example, by the group of Dube.^[^ ^45^ ^]^ The relatively short lifetimes detected by ultrafast transient absorption spectroscopy in toluene under an argon atmosphere with 375 and 490 nm excitation (Figure S15) are, however, not compatible with long‐lived triplets, consistent with oxygen‐assisted photooxidation from the singlet state. Indeed, excitation with the 375 nm pulse to the second bright transition induces a rapid decay (∼280 fs), characterized by broad stimulated emission centered at 600 nm and excited‐state absorption at 410 nm, into a longer‐lived state (assigned to S_1_, *τ* = 3.3 ps) that absorbs broadly across the visible (Figure S15). This state subsequently evolves into a persistent transient, attributed to the metastable ***E***‐**PI**, with a distinct absorption at 575 nm. Direct excitation of S_1_ (Figure S15, panel b) produces a similar broad transient (*τ* = 1.5 ps), as also observed following the 375 nm excitation, but in this case, no product is formed, confirming the anti‐Kasha behavior of **PI** seen in steady‐state spectroscopy.

The decreased stability in less polar environments prompted us to further examine **PI** in polar solvents such as acetonitrile and methanol. In MeCN and MeOH, irradiation of ***Z*‐PI** with 365, 395, or 505 nm light did not result in detectable isomerization or degradation (Figures S54–S56 and S74–S76, respectively). Ultrafast transient absorption spectroscopy in MeCN showed that **PI** exhibits excited‐state dynamics similar to those in toluene upon excitation to S_1_ with a 490 nm pulse (Figure S14). In contrast, excitation of the bright S_3_ state at 375 nm triggers rapid relaxation through two short‐lived intermediates resembling those in toluene (assigned to S_3_, *τ* ≈ 100 fs, and S_1_, *τ* ≈ 972 fs; Figure 2e), ultimately yielding a transient with spectral features closely matching the ground‐state absorption of **PI**, which we attribute to a hot ground state of ***Z*‐PI** (*τ* = 14.5 ps; Figure 2e).

We attribute the observed ultrafast decay of the excited state to relaxation through a conical intersection (CI), which closely resembles the equilibrium geometry of the ground state (CI*^Z^* in Figure S23) and lies on a different reaction coordinate than the productive conical intersections where the double bond is broken (see CI^Orth‐1^ and CI^Orth‐2^ in Figures S23 and 1b for a pictorial representation of the two competing paths of photostability and isomerization, which are both present in **PI**). CI*^Z^* connects the S_1_ and S_0_ surfaces in a manner highly sensitive to the surrounding polarity, similarly to a non‐productive conical intersection reported in a molecular motor, which also exhibited anti‐Kasha and polarity‐dependent photochemistry.^[^ ^46^ ^]^ Notably, the defining structural feature of CI*^Z^* is an elongated C(O)─N bond within the oxindole fragment, which extends to 1.876 Å at the MRSF‐TDDFT level in the gas phase. Despite this geometrical resemblance, the CI lies relatively high in energy, 25 and 30 kcal·mol^−1^ above the Franck‐Condon point at the MRSF‐TDDFT and SF‐TDDFT levels, respectively (see Figure S23), rendering it less accessible under non‐polar conditions. Consequently, alternative decay channels such as photoisomerization dominate in the gas phase, consistent with our experimental findings in solvents of low polarity. In contrast, modeling the system in methanol, using a combined explicit‐implicit solvation approach, reveals a substantial stabilization of the CI (see Figure S23b). Its energy drops to just 13 kcal·mol^−1^ above the S_1_ minimum, placing it well within reach even from the Franck–Condon S_1_ minimum. This energetic lowering facilitates efficient non‐radiative decay via the CI and suppresses isomerization, consistent with the isoindigo‐like photochemistry observed in polar media.^[^ ^37^ ^]^ These findings indicate that while polar solvents effectively stabilize **PI** against photodegradation, they also suppress photoisomerization in the absence of additional triggers.

**Figure 3:**
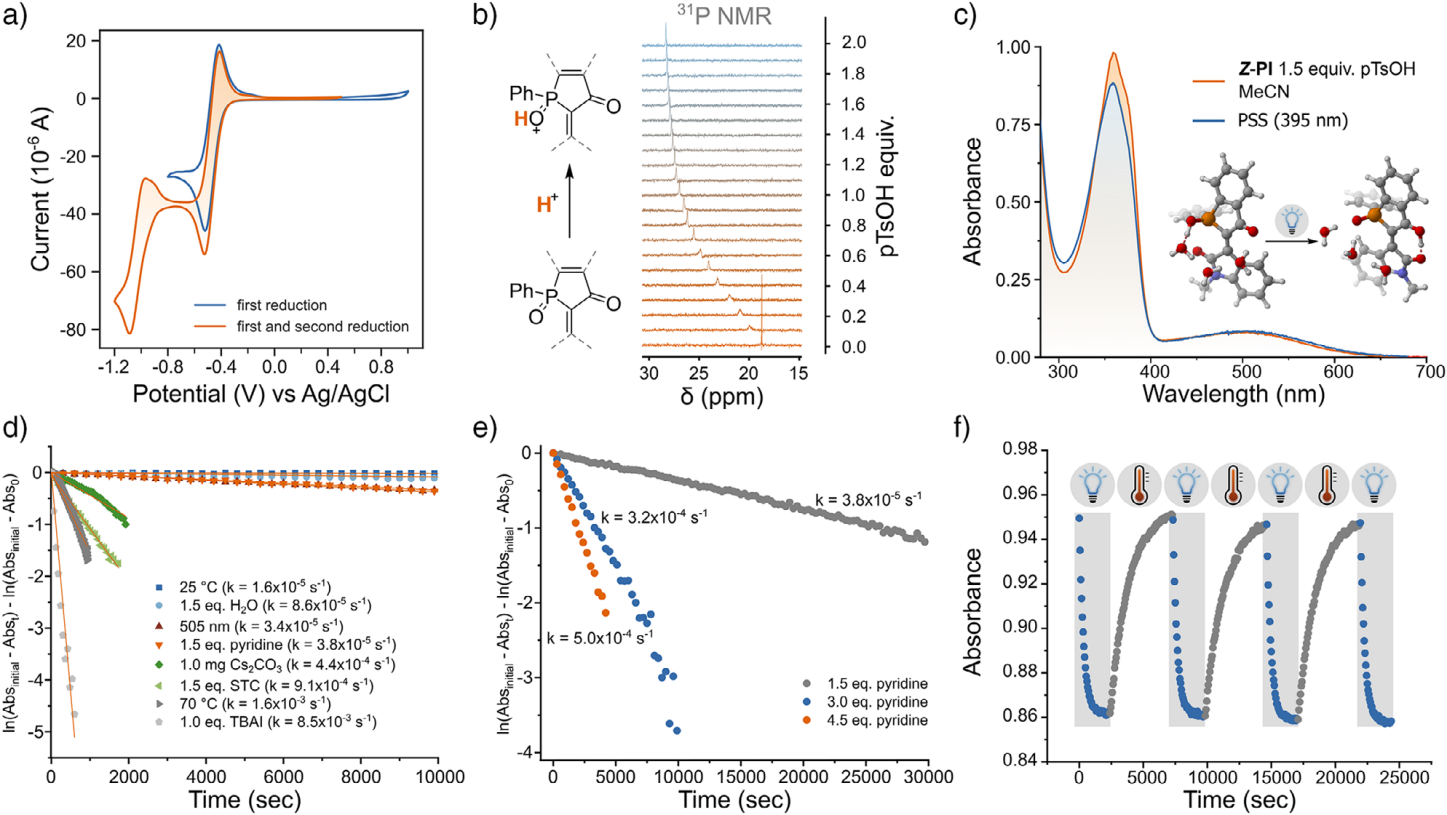
a) Cyclic voltammetry of **PI** (1 mM) in MeCN (0.1 M NBu_4_PF_6_) showing two reductions at −0.40 and −1.00 V versus Ag/AgCl, consistent with two one‐electron redox processes analogous to isoindigo systems. b) Protonation study by ^31^P NMR in MeCN‐d_3_ using incremental equivalents of pTsOH·H_2_O reveals downfield shifts and line broadening consistent with dynamic P═O protonation. c) UV–vis absorption spectra of ***Z*‐PI** in MeCN with 1.5 equiv. pTsOH·H_2_O before (orange) and after irradiation at 395 nm (blue), showing acid‐enabled photoisomerization. d) Thermal back‐isomerization kinetics of **PI** in MeCN after irradiation at 365 nm with various additives, including water, STC, pyridine, Cs_2_CO_3_, TBAI, and at elevated temperature, demonstrating tunable switching rates via nucleophilic catalysis. e) Dependence of back‐isomerization rate of **PI** in MeCN from the PSS at 365 nm on the concentration of pyridine, showing faster thermal kinetics at higher nucleophile concentration. f) Reversible photoswitching cycles (365 nm) of **PI** in MeCN with pyridine and 0.1 equiv. pTsOH·H_2_O, showing full recovery over multiple cycles, consistent with catalytic gating by acid and nucleophile‐promoted isomerization.

The analogy between **PI** and isoindigo is further supported by the electrochemical behavior of **PI**, which exhibits two reversible reduction processes at ca. −0.40 V and −1.00 V versus Ag/AgCl (Figures 3a and S125), underscoring, in addition, the potential of phosphaindirubin as a redox‐active chromophore.

Strikingly, we discovered that photoisomerization could be re‐enabled in MeCN and MeOH by the combination of acid with light (albeit with minimal decomposition observed in MeOH, see Figures S54–S56 and S74–S76). Irradiation of ***Z*‐PI** in MeCN with 1.5 equiv. of *p*‐toluenesulfonic acid monohydrate (pTsOH·H_2_O) using 365 or 395 nm light resulted in clean *Z*→*E* isomerization, as evidenced by a clear isosbestic point at 340 nm (Figure 3c). No switching was observed under 505 nm irradiation, consistent with the anti‐Kasha behavior observed in THF without acid (vide supra). Importantly, neither acid nor light independently was sufficient to induce isomerization in MeCN – only the synergistic effect of both triggered the switch. This light‐gated, acid‐assisted switching mechanism mirrors the dual‐mode activation observed in biological systems such as proteorhodopsin.^[^ ^2^ ^]^

To determine the minimum acid requirement, we conducted a series of irradiation experiments (365 nm) with varying equivalents of pTsOH·H_2_O. Regardless of acid concentration, the same photostationary state was reached, indicating that the acid functions catalytically rather than stoichiometrically (Figure S53). We also tested alternative acidic conditions, including trifluoroacetic acid (TFA), titanium isopropoxide (Ti(O*^i^* Pr)_4_), and 20 vol% hexafluoroisopropanol (HFIP) (Figure S67). All these conditions enabled photoisomerization, although the exact compositions at the photostationary states varied, demonstrating that the protonation environment provides a means to modulate photoswitching behavior.

To probe the nature of the acid‐base equilibria of **PI** in MeCN, we performed titrations with acids of varying strengths. Progressive additions of pTsOH·H_2_O or trifluoromethanesulfonic (triflic) acid resulted in significant downfield shifts in the ^31^P NMR spectrum (Figures 3b and S81–S85). Triflic acid (pK_a_ = 0.7 in MeCN)^[^ ^47^ ^]^ produced a larger shift (Δ*δ* = +22.3 ppm) than pTsOH·H_2_O (pK_a_ = 8.5 in MeCN;^[^ ^48^ ^]^ Δ*δ* = +8.1 ppm), indicating a higher degree of protonation. This semi‐quantitative comparison positions the intrinsic basicity of **PI** between the two acids on the MeCN acidity scale, in agreement with the reported pK_aH_ of triphenylphosphine oxide (2.80 in nitromethane),^[^ ^49^ ^]^ suggesting that **PI** is similar in basicity and exists in a dynamic equilibrium with the protonated form. DFT calculations support preferential protonation at the phosphine oxide rather than the carbonyl groups (see Section 8.4 in the Figures S25–S26).

The experimentally observed ^31^P NMR shifts align with the P═O site being protonated, particularly when explicit water molecules are included in the calculations to model pTsOH·H_2_O conditions (see Section 8.6 in the Figures S38 and S39). Complementary FTIR experiments in MeCN with TFA revealed no loss of the C═O stretch, but did show a shift in the C═C band, further corroborating protonation at P═O (Figures S79 and S80).

We propose that protonation alters the excited‐state potential energy surface of **PI** in a way that promotes isomerization. Notably, spin‐flip TDDFT with explicit methanol shows that protonation contracts the C(O)─N bond in both S_0_ and S_1_ (e.g., in S_1_ 1.444 Å in the unprotonated form against 1.428 Å in the protonated form, see Table S6), while C═C changes are minimal. Also, the calculations with MRSF‐TDDFT are in line with these results. Thus, acid gating attenuates the C(O)─N–driven CI*^Z^* channel, consistent with the observed acid‐enabled switching (see Table S6 and Figure S27). This additional stimulus can partially unlock the otherwise hindered isomerization pathway in polar solvents, particularly when the molecule is excited to higher states than S_1_, where the excess energy increases the likelihood of escaping from geometries near the Franck–Condon minimum.

Irradiation of **PI** in anhydrous MeCN in the presence of 1.5 equiv. of triflic acid initially led to efficient photoisomerization. However, prolonged irradiation under strictly anhydrous conditions resulted in the appearance of new absorption bands consistent with photodecomposition (Figure S58). Interestingly, the addition of trace amounts of water restored the original spectral profile and suppressed decomposition (Figure S65), indicating a stabilizing effect of water on the protonated **PI** species and a requirement for the photoisomerization to happen. While protonation with pTsOH·H_2_O did not result in any shift in absorption maximum, triflic acid in the presence of water caused a red‐shift of + 9 nm in the UV–vis spectrum (Figure S64), which is in line with computational predictions for protonation at the P═O moiety (Figure S37). These observations highlight the sensitivity of the protonation equilibrium, and hence the photostability of **PI**, to subtle changes in the solvation environment.

To better understand the isomerization process, we conducted in situ NMR irradiation experiments in MeCN‐d_3_ in the presence of acid. We found that the extent of isomerization was dependent on sample concentration: no photoisomerization was detected at 7 mM (Figure S90), while limited switching occurred at 3 mM (Figure S91), which was confirmed by LC‐MS analysis of the irradiated samples (Figure S104). The installation of an N‐butyl chain on the oxindole moiety of phosphaindirubin should hamper self‐aggregation and increase solubility. We attribute the concentration‐dependent behavior to an inner filter effect in the NMR tube, which attenuates excitation light at higher concentrations. We also attempted to determine the PSS ratios using LC‐MS (Figures S102 and S103). However, the results varied between replicates, likely due to the presence of water as eluent promoting back‐isomerization (*vide infra*). To address this, we applied constrained non‐negative matrix factorization to the UV–vis absorption spectrum at the PSS to retrieve the fraction of the metastable *E*‐isomer using the known absorption spectrum of the *Z*‐isomer,^[^ ^50^, ^51^ ^]^ enabling determination of PSS populations and quantum yields for photoisomerization in MeCN (*Ф_Z‐E_*: 0.1%, *Ф_E‐Z_*: 1.1% at 365 nm; *Ф_Z‐E_*: 0.2%, *Ф_E‐Z_*: 0.7% at 395 nm, Figures S110–S112). Due to the minimal negative photochromism and spectral separation between the *Z*‐ and *E*‐isomer (see Figures S29 and S110) in combination with the low quantum yields, enrichment of the metastable *E*‐isomer in the PSS is not very high in acetonitrile (30%). This relatively low accumulation of metastable *E*‐isomer can be attributed to the higher quantum yield that is observed for the backward *E*→*Z* photoisomerization (*Ф_E‐Z_* = 1.1%) as opposed to the *Z*→*E* photoisomerization (*Ф_Z‐E_* = 0.1%) at *λ*_365nm_, despite the molar extinction coefficient of the *Z*‐isomer being larger. The observed low quantum yields are in line with those of analogous indirubin^[^ ^18^ ^]^ and arise from a competing, more efficient non‐radiative decay pathway that suppresses the photoisomerization pathway.^[^ ^37^ ^]^ These low quantum yields originate from efficient non‐radiative relaxation through the conical intersection (CI*^Z^*) identified in our excited‐state calculations, characterized by elongation of the C(O)─N bond in the oxindole unit. This geometry facilitates ultrafast internal conversion to the ground state and competes directly with productive C═C rotation, limiting both the accumulation of the *E*‐isomer and the overall switching efficiency.

To better understand the thermal back‐isomerization of **PI**, we examined its behavior in acidic MeCN with varying amounts of water (Figures S113 and S114). Interestingly, water not only slowed down back‐isomerization but also appeared to irreversibly deprotonate **PI**, generating a neutral species that could no longer undergo forward photoisomerization. Larger amounts of water altered the thermal relaxation pathway, suggesting a more complex, multi‐step mechanism involving water itself. This observed sensitivity to water content is analogous to literature observations for indigo photoswitches.^[^ ^52^ ^]^

We hypothesized that water might also act as a nucleophile toward the central C═C double bond and thus modulate the back‐isomerization kinetics. To test this hypothesis, we systematically investigated the influence of various nucleophiles on the thermal back‐isomerization of the *E*‐isomer. Indeed, we found that nucleophiles such as pyridine, DABCO and OH^−^ (obtained from the reaction of the insoluble Cs_2_CO_3_ with the dissolved water) accelerated the apparent rate of back‐isomerization (Figures 3d and S116–S118), consistent with a nucleophile‐assisted reaction at the C═C bond, promoting the formation of the stable *Z*‐isomer. Even strongly hydrogen‐bonding additives such as Schreiner's thiourea catalyst (STC) unexpectedly accelerated back‐isomerization in **PI** (see Figures 3d and S119). This behavior suggests that STC may act as a Lewis acid instead, activating **PI** to react with the residual water in solution.

To decouple nucleophilicity from basicity, we tested tetrabutylammonium iodide (TBAI), a non‐basic, nucleophilic iodide source. The observed rapid back‐isomerization (see Figures 3d, S121, and 122) reinforced the conclusion that nucleophilic activation, rather than general base catalysis, governs the relaxation process. Remarkably, we also observed that **PI** could undergo photoinduced back‐isomerization upon irradiation at 505 nm in MeCN under acidic conditions (Figure S115), selectively addressing the S_1_ state.

Finally, we investigated whether the back‐isomerization rate could be tuned by nucleophile concentration. By varying the amount of pyridine added to the system, we could precisely control both the back‐isomerization rate and the PSS composition (Figure 3e). Higher pyridine concentrations led to faster relaxation and lower *E*‐isomer populations at the PSS. Notably, the use of pyridine allowed for repeated cycling between the *Z*‐ and *E*‐isomers of **PI**. When we combined 4.5 equiv. of pyridine with either 0.1 or 1.5 equivalents of pTsOH in MeCN, **PI** was able to complete three full photo‐switching cycles of photoisomerization and thermal back‐isomerization with no detectable fatigue, even in the presence of catalytic acid and nucleophile (Figures 3f and S123). To the best of our knowledge, this is the first example of an artificial photoswitch where the simultaneous presence of three stimuli, namely light, acid, and a nucleophile, allows precise control of the isomerization. Whether this behavior is observed upon substitution of phosphaindirubin remains an open query; work to establish this is currently being performed.

## Conclusion

While initially designed to explore the impact of phosphorus on switching behavior, we serendipitously discovered a strikingly noncanonical mechanism of control in phosphaindirubin. In apolar or slightly polar solvents, **PI** undergoes light‐induced isomerization, albeit with signs of instability and possible decomposition.

However, in polar solvents such as MeCN, **PI** completely loses its ability to isomerize photochemically unless catalytic amounts of acid are present. This behavior is reminiscent of the proton‐gated switching observed in biological systems, suggesting that protonation alters the excited‐state potential energy surface. This alteration may involve shifting or suppressing a conical intersection in the Franck‐Condon region, which would otherwise funnel the system back to the ground state nonproductively.

In a further unexpected twist, we discovered that weak nucleophiles catalyze the thermal back‐isomerization of the metastable form, a mechanism that is rarely utilized actively as an isomerization strategy in photoswitches. By varying the type and concentration of nucleophile, we were able to control the thermal back‐isomerization precisely.

Taken together, these findings reveal a photoswitching system uniquely responsive to three stimuli: light, acid, and nucleophile. This triply gated control over isomerization and back‐isomerization enables cyclical switching behavior. To the best of our knowledge, this is the first example of an artificial photoswitch that integrates this level of environmental responsiveness.

## Supporting Information

The authors have cited additional references within the Supporting Information.^[^ ^53^, ^54^, ^55^, ^56^, ^57^, ^58^, ^59^, ^60^, ^61^, ^62^, ^63^, ^64^, ^65^, ^66^, ^67^, ^68^, ^69^, ^70^, ^71^, ^72^, ^73^, ^74^, ^75^, ^76^, ^77^, ^78^, ^79^, ^80^, ^81^, ^82^, ^83^, ^84^, ^85^, ^86^, ^87^, ^88^, ^89^, ^90^, ^91^, ^92^, ^93^, ^94^, ^95^, ^96^, ^97^ ^]^

## Conflict of Interests

The authors declare no conflict of interest.

## Supporting information



Supporting Information

Supporting Information

## Data Availability

The data that support the findings of this study are available in the supplementary material of this article.
