# Supplementary material for: Acid‐ and Nucleophile‐Gated Photoisomerization of Phosphaindirubin
Source: Angew Chem Int Ed Engl. 2025 Nov 30;65(10):e19686. doi: 10.1002/anie.202519686 (PMC12955533; doi:10.1002/anie.202519686)
Supplement: Supplementary file 1 — Supporting Information [file ANIE-65-e19686-s001.pdf]

**Table of contents**

1. Experimental Procedures 3
1.1. General Details 3
1.2 X-ray Crystallographic Details 4
1.3 Reagent Information 4
1.4 Transient Absorption Spectroscopy 5
1.5 Computational Methods 6
1.6 Synthetic Procedures 8
2. NMR Spectra 11
3. HRMS 19
4. Circular dichroism (CD) Spectroscopy 19
5. UV-Vis Spectra 20
6. Ultrafast Spectroscopy 21
7. X-ray Crystallographic Data 23
8. Computational Data 24
8.1 Isomers of PI 24
8.2 Energy Barriers and Relative Gibbs Free Energies 26
8.3 Potential Energy Surface, Minima and Conical Intersections 29
8.4 Protonation of Phosphaindirubin 32
8.5 TD-DFT Calculations: Simulated UV-Vis Absorption Spectra 36
8.5.1 Natural Transition Orbitals 45
8.5.2 Protonated Phosphaindirubin 47
8.6 $^{31}$P NMR Calculations 48
8.6.1 Without Water 48
8.6.2 With 3 Water Molecules 49
8.7. Simulated IR spectra 50
9. UV-Vis Irradiation 54
9.1 Toluene 54
9.2 Tetrahydrofuran 56
9.3 Acetonitrile 58
9.3.1 Acid Presence in Solvent 58
9.3.2 Irradiation 60
9.3.3 Irradiation with Various Acids 63
9.3.4 Irradiation with Triflic Acid 64
9.3.5 Effect of Water 67
9.3.6 PSS Modulation 72
9.3.7 Control Experiments 75

9.4 Methanol 77
9.4.1 Irradiation 77
9.4.2 Argon and Water Dependency 80
10. Protonation State Studies 82
10.1 UV-Vis Spectra 82
10.2 FTIR Spectra 83
10.3 NMR Studies 86
11. NMR Irradiation 89
11.1 THF 89
11.2 Chloroform 91
11.3 Acetonitrile 92
12. LCMS-data 94
12.1 THF 94
12.2 Acetonitrile 100
12.2.1 The Determination of the Stable and Metastable Isomer in LCMS 100
12.2.2 The Difference in PSS Determination in LCMS 104
12.2.3 *In-Situ* NMR Irradiation Sample 106
12.2.4 Pyridine 108
13. Quantum Yields 112
13.1 UV-Vis Spectrum Metastable State 112
13.2 Quantum Yields at 365 nm 112
13.3 Quantum Yields at 395 nm 113
14. Back-Isomerization 114
14.1 The Effect of Water 114
14.2 The Effect of Light 116
14.3 The Effect of Bases and Nucleophiles 116
14.3.1 Pyridine 116
14.3.2 Caesium Carbonate (Hydroxy Anion) 118
14.3.3 Schreiner's Thiourea Catalyst (STC) 118
14.3.4 1,4-Diazabicyclo[2.2.2]octane (DABCO) 119
14.3.5 Tetrabutylammonium Iodide (TBAI) 120
15. Cycles with Pyridine 121
16. Cycles with TBAI 121
17. Electrochemistry 122
19. References 123

## 1. Experimental Procedures

### 1.1. General Details

All air-sensitive manipulations were performed under a positive atmosphere of purified argon using the standard high-vacuum Schlenk-line technique. Air Liquide Europe supplied argon. All glassware was oven-dried, cooled under vacuum, and purged with argon before use. Solvents were freshly collected from a *Pure Solve MD7* solvent purification system under argon. NMR spectra were recorded on a Jeol Eclipse+ 400 ($^{1}$H, 400 MHz; $^{13}$C, 101 MHz; $^{31}$P, 162 MHz) spectrometer at 298 K unless noted otherwise. Chemical shift values are quoted in δ (ppm) and coupling constants in *J* (Hz). $^{1}$H chemical shift values are reported relative to tetramethylsilane (TMS) and referenced to the residual proton resonances of the corresponding deuterated solvent signal. $^{13}$C{$^{1}$H} NMR spectra are reported relative to TMS using the natural abundance carbon resonances of the deuterated solvents. The following abbreviations (or combinations thereof) were used to describe multiplicities: s, singlet; d, doublet; t, triplet; m, multiplet; brs, broad singlet. High-resolution mass spectra (HR-MS) were recorded on a Thermo Scientific Orbitrap LTQ XL spectrometer.

Reactions were monitored by thin-layer chromatography (TLC) using Merck TLC Silica Gel 60 $F_{254}$ aluminium sheets or Merck TLC Aluminium oxide 60 $F_{254}$ aluminium sheets and visualised by UV-light (λ = 365 nm or 254 nm). Chromatographic purifications of products were accomplished using flash column chromatography (FC) on pre-packed silica gel columns from Silicycle 60 (0.04 – 0.063 nm/230 – 400 mesh) and reversed-phase flash column chromatography (RPFC) on a pre-packed C18 flash column from TELOS on an automated low-pressure Biotage SP4 using eluent gradients.

High-performance liquid chromatography (HPLC) analysis was carried out using an Agilent 1290 Infinity II HPLC system equipped with a 1290 Infinity II high-speed pump and a 1260 II Infinity DAD HS UV-Vis detector, using an InfinityLab Poroshell 120 EC-C18 column with dimensions of 50 mm x 2.1 mm and 1.9 µm particle size. The HPLC is coupled to an InfinityLab LC/MSD G6125B detector equipped with an electrospray ionization (ESI) source. LC separation was performed with water (A, 0.05% formic acid): acetonitrile (B, 0.05% formic acid) eluent system using the methods *LC1* and *LC2*. *LC1*: 1→10 min: 10% B; 1→10 min: 10%→90% B and 10–11 min: 90% B, 11–12 min: 10% B, Flow rate: 0.8 mL/min. *LC2:* 1→10 min: 10% B; 1→10 min: 10%→90% B and 10–11 min: 90% B, 11–12 min: 10% B, 0.3 mL/min.

UV-Vis irradiation experiments were performed in a screw-cap fluorescence cuvette (Hellma 117.100F-QS), containing a 5 mm PTFE-coated stirring bar (VWR), using a home-built fibre-optic setup as reported in Volker *et al.*.[1] Briefly: consisting of a Quantum Northwest Luma 40 Peltier-based temperature-controlled cuvette holder with four optical windows: an Avantes AvaLight-DH-S-BAL light source coupled with a 400 µm fibre (Avantes FC-UVIR400-1-BX) to an SMA-to-SM1 fibre adapter (Thorlabs CVH100-COL) containing a plano-convex lens (20.1 mm focal length, Thorlabs LA4647) in a 1-inch diameter lens mount (Thorlabs LMR1S/M) placed flush against one side; an Avantes AvaSpec-ULS2048CL-EVO-RS spectrometer coupled with a 400 µm fibre (Avantes FC-UVIR400-1-BX) to an SMA-to-SM1 fibre adapter (Thorlabs CVH100-COL) containing a plano-convex lens (20.1 mm focal length, Thorlabs LA4647) in a 1-inch diameter lens mount (Thorlabs LMR1S/M) placed flush against the opposite side; and a 365 nm light-emitting diode (LED) (Thorlabs M365FP1) coupled with a 600 µm fibre (Thorlabs M114L01) to an adjustable fibre collimator (Thorlabs CFCS5-A) mounted in an adapter (Thorlabs AD15F2) in a 1-inch diameter lens mount (Thorlabs

LMR1S/M) placed flush against one of the remaining optical ports (*i.e.* orthogonal to the spectrometer light path).

Quantum yields for **PI** were determined in acetonitrile following the procedure and instrumentation described in Volker *et al.,*[1] together with our recently developed graphical user interface.[2] The absorptivity of the metastable (*E*-) isomer of **PI** was determined by the weighted subtraction of the spectrum of the stable (*Z*-) isomer from the spectrum of $PSS_{365}$. The molar absorptivity spectrum of the metastable isomer was obtained by constrained non-negative matrix factorization.[3,4]

Fourier transform infrared spectra (FTIR) were recorded on a Perkin Elmer Spectrum ONE spectrometer in transmission mode using a liquid cell ($CaF_2$, SPECAC).

Circular dichroism (CD) spectra were collected on a Chirascan VX (Applied Photophysics) equipped with a temperature-controlled cell Peltier holder. They were recorded from 300-600 nm with a 1 s acquisition time per data point using a 2 mm cuvette and a cell temperature of 25 °C.

Electrochemical measurements were performed on a Metrohm Autolab potentiostat (PGSTAT204) using the software Nova 2.1.4 at an analyte concentration of 1.0 mM in argon-purged acetonitrile containing 0.1 M tetrabutylammonium hexafluorophosphate ($TBAPF_6$). The electrodes employed were a 3 mm diameter Teflon-shrouded glassy carbon (GC) working electrode (CH Instruments), a Pt wire auxiliary electrode, and an Ag/AgCl wire reference electrode.

### 1.2 X-ray Crystallographic Details

Single crystals were mounted on a fibre loop and fixated using Fomblin oil. The data was collected on a Bruker D8 APEX-II equipped with an APEX-II CCD camera using MoKα radiation (λ = 0.71073 Å). Data reduction was performed with SAINT, and absorption corrections for the area detector were performed using SADABS.[5] Structures were solved by direct methods and refined by the least squares method on $F^2$ using the SHELX and OLEX2 software suites, respectively.[6,7] All the non-hydrogen atoms were refined using an anisotropic model, and all the hydrogen atoms were constrained in geometrical positions to their parent atom. Crystallographic data are presented in **Table S1**. Deposition number CCDC 2479875 contains the supplementary crystallographic data for this paper. These data can be obtained free of charge via www.ccdc.cam.ac.uk/data_request/cif, by emailing data_request@ccdc.cam.ac.uk, or by contacting The Cambridge Crystallographic Data Centre, 12 Union Road, Cambridge CB21EZ, UK; fax: +441223336033.

### 1.3 Reagent Information

*ⁿ*BuLi (1.6 M in hexanes), P,P-dichlorophenylphosphine, anhydrous pyridine, (*S*)-(-)-4-Isopropyl-5,5-dimethyl-2-oxazolidinone, and 2-bromobenzonitrile were purchased from Sigma-Aldrich and used as received. All solvents were of reagent grade for both synthesis and purification and purchased from VWR.

### 1.4 Transient Absorption Spectroscopy

Time-resolved UV-Vis spectroscopy for **PI** in MeCN and toluene was performed using 100 fs pulses centered at 800 nm, produced by an integrated Ti:Sapphire Regenerative Amplifier Laser (Solstice Ace), at an output power of 2.3 mJ on average, at a repetition rate of 3 kHz. Each 800 nm pulse is split to form a pump and a probe pulse. The pump pulse is obtained by sending the major part of the 800 nm pulse into an optical parametric amplifier (TOPAS-Twins), yielding a 3 kHz, 100 fs, tunable laser pulse (1160-2600 nm), at powers of 10μJ/pulse. The aforementioned tunable laser pulse is obtained from the signal beam and/or the idler beam. By directing either of the latter, or both, into nonlinear crystals, one can perform frequency doubling (second harmonic generation), quadrupling (fourth harmonic generation), or frequency mixing (sum or difference frequency generation) to access a wide range of wavelengths. The probe pulse is generated by focusing the 800 nm pulse onto a $CaF_2$ window, producing a white light continuum between 325-750 nm. The light path of the probe contains a gold reflector mounted on a motorized optical delay line, which changes the path length of the probe beam, and therefore the relative timing of the pulses. The relative polarization between the pump and probe pulses was set at the magic angle (54.7°) to minimize contributions from molecular reorientation. The probe beam was then passed through the sample with the residual 800 nm filtered using a short pass filter, and the light was directed onto the slit of a spectrograph (Andor Kymera 139i), focused onto a grating and dispersed onto a 512-pixel CCD camera. Half of the pump pulses were blocked using a synchronized chopper, allowing the measurement of the intensity of the probe with and without the pump present on the sample. The difference in absorbance was then calculated as:

$$\Delta Abs = -{}^{10}\log\frac{I_{pump+probe}}{I_{onlyprobe}}$$

The measured energy at the sample at 375 nm was 2.7 μJ and at 490 nm was 7.7 μJ . We used a sample of about 0.5-1.5 optical density (OD). The sample was mounted on an automated, movable, two-axis stage, controlled by in-house software to avoid bleaching of the solution. The data was processed with Glotaran 1.5.1[8] using global analysis, applying a sequential decay kinetic scheme.

## 1.5 Computational Methods

The structure of the molecule used for the calculations is the phosphaindirubin with a methyl group instead of a butyl group on the nitrogen. It is still designated **PI**.

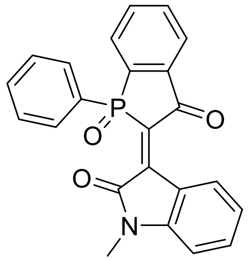


**Figure S1.** The molecular structure of **PI** used for the calculations: *N*-Me instead of *N*-Bu.

Pre-optimization of the *Z*-isomer (***Z***) and *E*-isomers (***E1*** and ***E2***) was carried out with the semi-empirical tight-binding software xTB 6.6.1 at the GFN2-xTB level.[9,10] Density functional theory (DFT) and spin-flip (SF-)DFT geometry optimizations and single-point calculations were carried out with the ORCA 5.0.4 and 6.0.0 quantum chemical packages, respectively.[11,12]

The transition states were first found using pysisyphus[13] at the GFN2-xTB level and were re-optimized using ORCA 5.0.4 at the r$^2$SCAN-3c level[14] with broken symmetry.[15]

Single point DFT calculations of the ground state and transition states were carried out with the functional ωB97X-V,[16–18] unless stated otherwise, using the def2-TZVPP basis set,[19] which is corrected with the geometrical Counterpoise Correction gCP,[20] and the Conductor-like Polarizable Continuum Model (CPCM) for solvation.[21] The simulated UV-Vis absorption spectra were calculated using time-dependent DFT (TDDFT) calculations with ORCA 5.0.4 at the M06-2X[22] level with the def2-TZVPP[19] basis set, unless stated otherwise, together with the CPCM model[21] on the optimized structures for 15 roots. The NMR chemical shifts are the isotropic values calculated with ORCA 5.0.4 (using the *NMR* keyword) at the ωB97X-V level with the def2-QZVP[19,23] basis set together with the CPCM solvation model.

SF-DFT optimizations were carried out using the BH&HLYP[24] functional with the def2-SVP[19] basis set, corrected for basis set superposition error with gCP correction. Implicit solvation was carried out using a solvation model based on density (SMD, methanol).[[25]]

Mixed-reference spin-flip time-dependent DFT (MRSF-TDDFT) calculations were performed with the quantum chemical software OpenQP[26] using the functional BH&HLYP[24] and a custom basis set: 6-31G(d) for H,C,N,O[27–29] and 6-311G(d) for P[30,31] as described at The Basis Set Exchange.[32–34] Since this custom basis set, named 6-31G(d)/6-311G(d), was not included in the installation of OpenQP at the time of writing, we added it ourselves before compilation of the software (see figshare repository link on the next page).

For MRSF-TDDFT geometry optimizations of minima at the ground and excited state and transition states, OpenQP at the BH&HLYP/6-31G(d)/6-311G(d) level was used for gradient calculation, while ORCA 6.0.1[35–37,11] was used as an external optimizer using a wrapper script developed in our group.[38]

To locate minimum energy conical intersections using MRSF-TDDFT, OpenQP was used at the BH&HLYP/6-31G(d)/6-311G(d) level of theory, employing the Update Branching Plane (UBP) search algorithm.

To generate the potential energy surfaces at the $S_0$, $S_1$, $S_2$ and $T_1$ states, firstly CREST 3.0 was used to optimize the constrained geometries for the scans of the rotation dihedral angle and the C-N bond length (between the carbonyl C atom and the N atom in the oxindole part, **Figure S21**) at the non-self-consistent GFN0-xTB level for the $S_1$ state,[39,40] followed by single-point calculation of the thus-obtained geometries at the MRSF-TDDFT/BH&HLYP/6-31G(d)/6-311G(d) level with three roots.

The Quantum Cluster Growth (QCG) algorithm[41] of CREST 3.0[39] was used for the addition of explicit water molecules.

The energy diagrams were created using the program Energy_Diagram_Plotter_CDXML.[42] The natural transition orbitals were generated using Multiwfn.[43,44]

The cartesian coordinates of the optimized structures are provided as .xyz files (including the energy as a comment on the second line) in the figshare repository with the following DOI: 10.6084/m9.figshare.29851256

## 1.6 Synthetic Procedures

Phenylphosphonite[45], methyl methyl(phenyl)phosphinate[45,46], methyl(phenyl)phosphinic chloride[45,46], (*S*)-4-*i*Pr-5,5-dimethyl-3-[(*Rp*)-methyl(phenyl)phosphinoyl]oxazolidine-2-one[46] and 1-butyl-indoline-2,3-dione[47] were synthesized according to literature procedures.

Synthesis of 1-phenylbenzophospholan-3-one oxide – **benzophospholane (BP)**

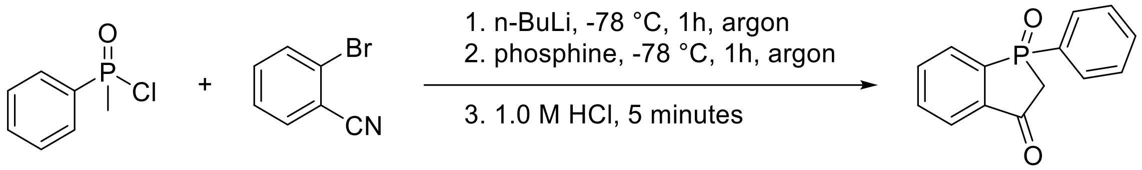


The procedure was modified from previously reported literature.[48] To an oven-dried 50 mL Schlenk flask, THF *(30 mL)* was added, and 2-bromobenzonitrile *(0.532 g, 2.9 mmol, 2.0 eq.)* was dissolved under an argon atmosphere. This was cooled to -78 °C using an acetone-liquid nitrogen bath. *n*-Butyl lithium *(1.6 M in hexanes, 1.85 mL, 2.9 mmol, 2.0 eq.)* was added dropwise at -78 °C and stirred at this temperature for 1 hour. Followed by the addition of methyl(phenyl)phosphinic chloride *(0.2 mL, 1.45 mmol, 1.0 eq.)* at -78 °C and stirred at this temperature for 1 hour. The reaction mixture was allowed to warm slowly to room temperature and was stirred for 2 hours. The reaction was worked up by the addition of 1.0 M hydrochloric acid solution *(20 mL)* and extracted with ethyl acetate *(3 x 40 mL)*. Washed with brine *(50 mL)*, dried over anhydrous sodium sulfate, and concentrated in vacuo. Purified by FC *(100% Ethyl acetate)*. This afforded a yellow oil *(241 mg, 69%)*.

$^{1}$H NMR ($CDCl_3$, 400 MHz): δ 8.06 (dddt, $J$ = 7.3, 2.4, 1.6, 0.7 Hz, 1H), 7.92 – 7.85 (m, 1H), 7.85 – 7.74 (m, 2H), 7.56 (dtt, $J$ = 14.8, 6.9, 1.4 Hz, 3H), 7.51 – 7.43 (m, 2H), 3.34 – 3.10 (m, 2H).

$^{13}$C{$^{1}$H} NMR ($CDCl_3$, 101 MHz): δ 194.5 (d, $J_{PC}$ = 13.5 Hz), 141.8 (d, $J_{PC}$ = 91.4 Hz), 141.5 (d, $J_{PC}$ = 12.5 Hz), 136.1 (d, $J_{PC}$ = 10.9 Hz), 133.8 (d, $J_{PC}$ = 2.4 Hz), 132.7 (d, $J$ = 3.1 Hz), 131.1 (d, $J_{PC}$ = 105.5 Hz), 130.8 (d, $J_{pc}$ = 10.7 Hz), 129.5 (d, $J_{pc}$ = 6.0 Hz), 129.2 (d, $J_{PC}$ = 13.0 Hz), 124.9 (d, $J$ = 11.3 Hz), 40.3 (d, $J_{PC}$ = 40.3 Hz).

$^{31}$P NMR ($CDCl_3$, 101 MHz): δ +30.5.

LCMS-ESI$^{+}$ (m/z): calculated for $C_{14}H_{12}O_2P^{+}$ $[M+H]^{+}$ 243.1; found 243.0.

R$_f$: 0.30 (100% ethyl acetate).

The analytical data is in agreement with the published data.[48]

Synthesis of (S)-1-phenylbenzophospholane-3-one oxide – **benzophospholane (BP)**

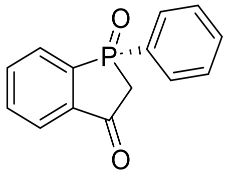

Following the same procedure reported for compound **5**, using (*S*)-4-*i*Pr-5,5-dimethyl-3-[(*Rp*)-methyl(phenyl)phosphinoyl]oxazolidine-2-one.This afforded a yellow oil *(50 mg, 47%)*.

$^{1}$H NMR ($CDCl_3$, 400 MHz): δ 8.06 (dddt, *J* = 7.3, 2.4, 1.6, 0.7 Hz, 1H), 7.92 – 7.85 (m, 1H), 7.85 – 7.74 (m, 2H), 7.56 (dtt, *J* = 14.8, 6.9, 1.4 Hz, 3H), 7.51 – 7.43 (m, 2H), 3.34 – 3.10 (m, 2H).

$^{13}$C{$^{1}$H} NMR ($CDCl_3$, 101 MHz): δ 194.5 (d, $J_{PC}$ = 13.5 Hz), 141.8 (d, $J_{PC}$ = 91.4 Hz), 141.5 (d, $J_{PC}$ = 12.5 Hz), 136.1 (d, $J_{PC}$ = 10.9 Hz), 133.8 (d, $J_{PC}$ = 2.4 Hz), 132.7 (d, *J* = 3.1 Hz), 131.1 (d, $J_{PC}$ = 105.5 Hz), 130.8 (d, $J_{pc}$ = 10.7 Hz), 129.5 (d, $J_{pc}$ = 6.0 Hz), 129.2 (d, $J_{PC}$ = 13.0 Hz), 124.9 (d, *J* = 11.3 Hz), 40.3 (d, $J_{PC}$ = 40.3 Hz).

$^{31}$P NMR ($CDCl_3$, 101 MHz): δ +30.5.

LCMS-ESI$^+$ (m/z): calculated for $C_{14}H_{12}O_2P^+$ $[M+H]^+$ 243.1; found 243.0.

R*f*: 0.30 (100% ethyl acetate).

The analytical data is in agreement with the published data.[48]

Synthesis of (*Z*)-1-butyl-3-(1-oxido-3-oxo-1-phenyl-3-hydrophosphindol-2-ylidene)indoline-2-one – **Phosphaindirubin (PI)**

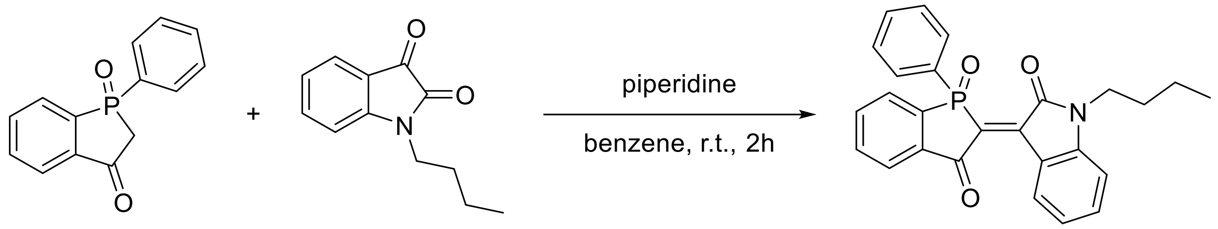


The procedure was modified from previously reported literature.[47] An oven-dried 15 mL crimp-top vial was evacuated for 15 minutes and backfilled with argon. Under an inert atmosphere, benzophospholane *(40.2 mg, 0.17 mmol, 1.0 eq.)* and 1-butylindoline-2,3-dione *(35.2 mg, 0.18 mmol, 1.0 eq.)* were added and dissolved in anhydrous benzene *(1 mL)*. Followed by the addition of 2 drops of piperidine, and the reaction was stirred at room temperature for 120 minutes. The reaction mixture was diluted with ethyl acetate *(20 mL)* and washed with water *(2 x 20 mL)* and brine *(20 mL)*. The organic layer was dried over anhydrous sodium sulfate and concentrated in vacuo. The product was purified by FC *(0-75% gradient of ethyl acetate in pentane)*. This afforded a purple solid *(43.4 mg, 61%)*.

$^{1}$H NMR ($CDCl_3$, 400 MHz): δ 9.22 (d, *J* = 8.0 Hz, 1H, **H$_{9}$**), 8.17 – 8.12 (m, 1H, **H$_{16}$**), 7.85 – 7.79 (m, 1H, **H$_{19}$**), 7.77 – 7.71 (m, 3H, **H$_{17}$+H$_{18}$**), 7.71 – 7.67 (m, 1H, **H$_{22}$**), 7.49 – 7.42 (m, 2H, **H$_{7}$+H$_{24}$**), 7.42 – 7.36 (m, 2H, **H$_{23}$**), 7.10 (td, *J* = 7.8, 1.0 Hz, 1H, **H$_{8}$**), 6.77 (d, *J* = 7.8 Hz, 1H, **H$_{6}$**), 3.69 – 3.54 (m, 2H, **H$_{4}$**), 1.55 (m, *J* = 7.5 Hz, 2H, **H$_{3}$**), 1.24 – 1.16 (m, 2H, **H$_{2}$**), 0.84 (t, *J* = 7.4 Hz, 3H, **H$_{1}$**).

$^{13}$C{$^{1}$H} NMR ($CDCl_3$, 101 MHz): δ 189.3 (d, $^{2}J_{PC}$ = 30.3 Hz, **C$_{14}$**), 165.9 (**C$_{12}$**), 148.4 (**C$_{5}$**), 143.9 (d, $^{2}J_{PC}$ = 4.7 Hz, **C$_{11}$**), 142.9 (d, $^{1}J_{PC}$ = 96.1 Hz, **C$_{13}$**), 140.5 (d, $^{2}J_{PC}$ = 7.7 Hz, **C$_{15}$**), 136.1 (d, $^{3}J_{PC}$ = 10.7 Hz, **C$_{18}$**), 135.7 (**C$_{7}$**), 133.3 (d, $^{4}J_{PC}$ = 1.5 Hz, **C$_{17}$**), 133.2 (d, $^{1}J_{PC}$ = 95.2 Hz, **C$_{20}$**), 132.0 (d, $^{1}J_{PC}$ = 117.5 Hz, **C$_{21}$**), 131.9 (**C$_{24}$**), 131.6 (d, $^{4}J_{PC}$ = 3.3 Hz, **C$_{9}$**), 131.2 (d, $^{2}J_{PC}$ = 11.4 Hz, **C$_{22}$**), 129.8 (d, $^{2}J_{PC}$ = 6.4 Hz, **C$_{19}$**), 128.3 (d, $^{3}J_{PC}$ = 13.9 Hz, **C$_{23}$**), 124.8 (d, $^{3}J_{PC}$ = 9.0 Hz, **C$_{16}$**), 123.0 (**C$_{8}$**), 121.0 (d, $^{3}J_{PC}$ = 12.0 Hz, **C$_{10}$**), 109.0 (**C$_{6}$**), 40.0 (**C$_{4}$**), 29.3 (**C$_{3}$**), 20.1 (**C$_{2}$**), 13.7 (**C$_{1}$**).

$^{31}$P NMR ($CDCl_3$, 101 MHz): δ +20.8.

HRMS (ESI$^{+}$, methanol) (m/z): calculated for $C_{26}H_{22}NO_3PNa^{+}$ [M+Na]$^{+}$ 450.1235; found: 450.1225.

LCMS-ESI$^{+}$ (m/z): calculated for $C_{26}H_{23}NO_3P^{+}$ [M+H]$^{+}$ 428.1; found 428.1.

UV-Vis (MeCN, nm): $\lambda_{max}$ 267 (2250), 360 (22800), 518 (1800).

UV-Vis ($PhCH_3$, nm): $\lambda_{max}$ 285 (11200), 364 (20300), 516 (1800).

UV-Vis (MeOH, nm): $\lambda_{max}$ 267 (22300), 363 (21700), 496 (1600).

R*f*: 0.33 (100% ethyl acetate).

## 2. NMR Spectra

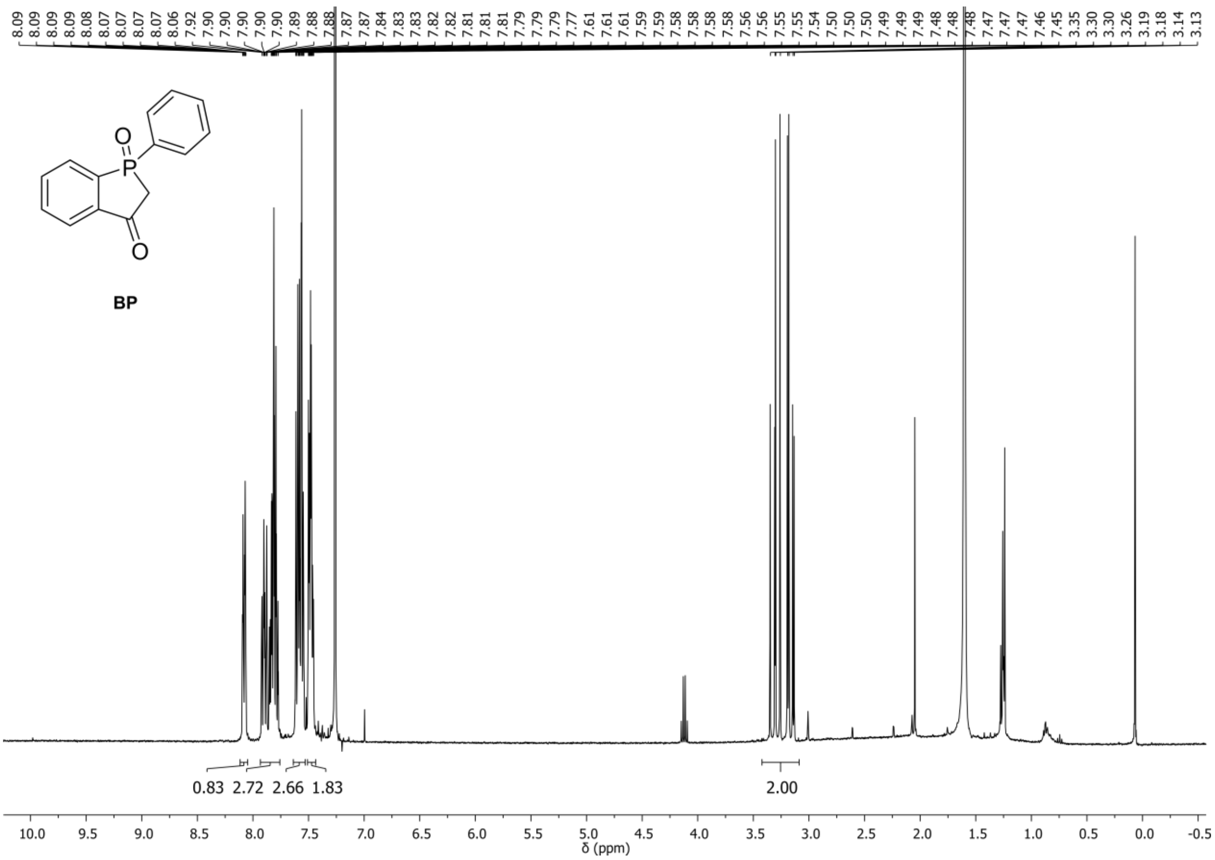


**Figure S2.** $^{1}$H NMR of **BP** in $CDCl_3$ at 25 °C. The additional solvent peaks belong to ethyl acetate.

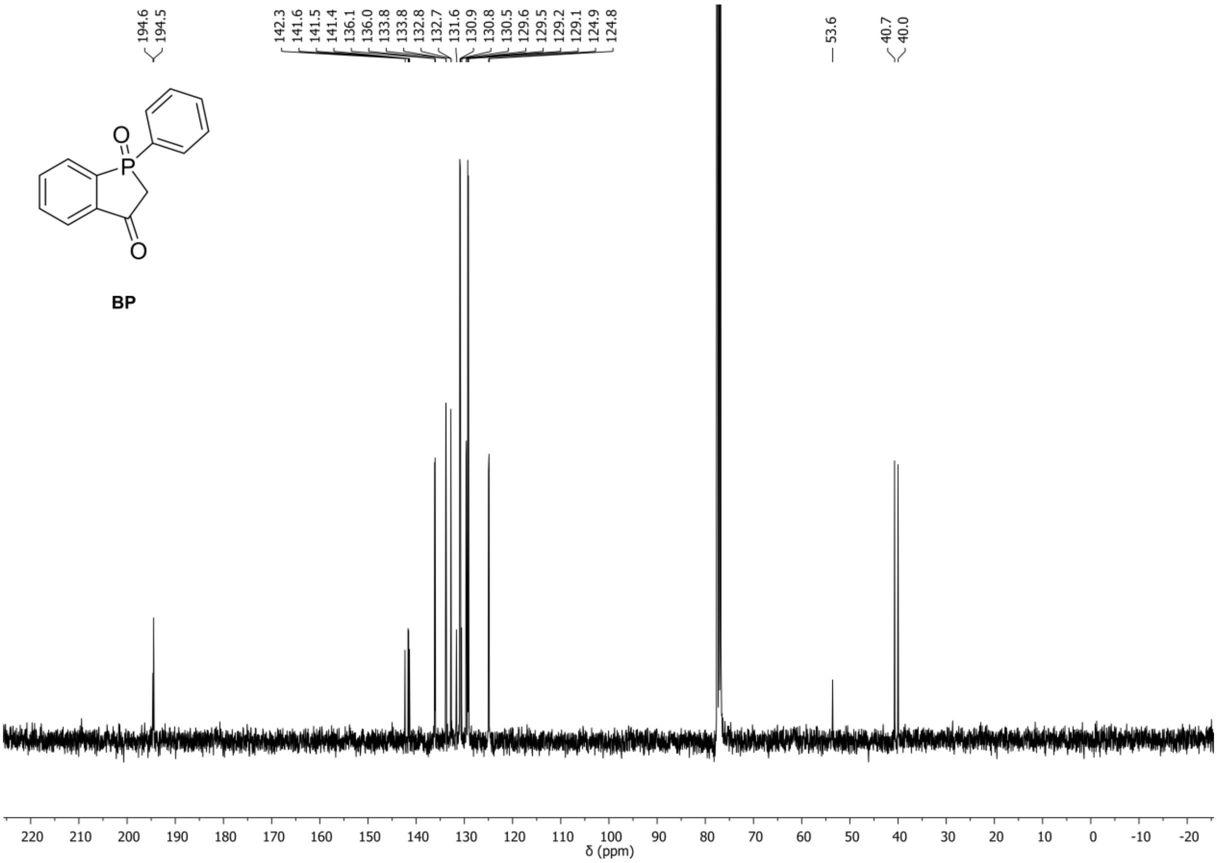


**Figure S3.** $^{13}C\{^{1}H\}$ NMR of **BP** in $CDCl_3$ at 25 °C. The additional solvent peak belongs to $CH_2Cl_2$ (δ = 29.8 ppm).

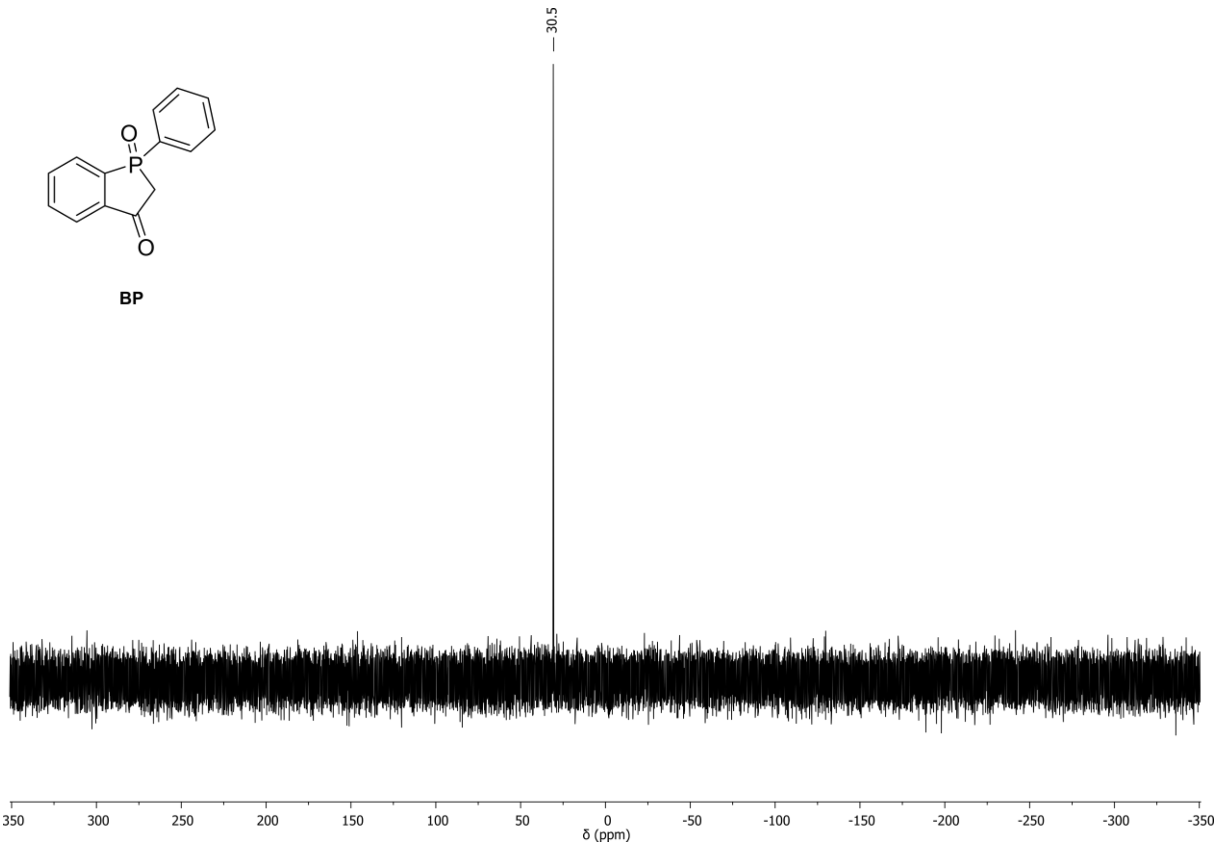


**Figure S4.** $^{31}P$ NMR of **BP** in $CDCl_3$ at 25 °C.

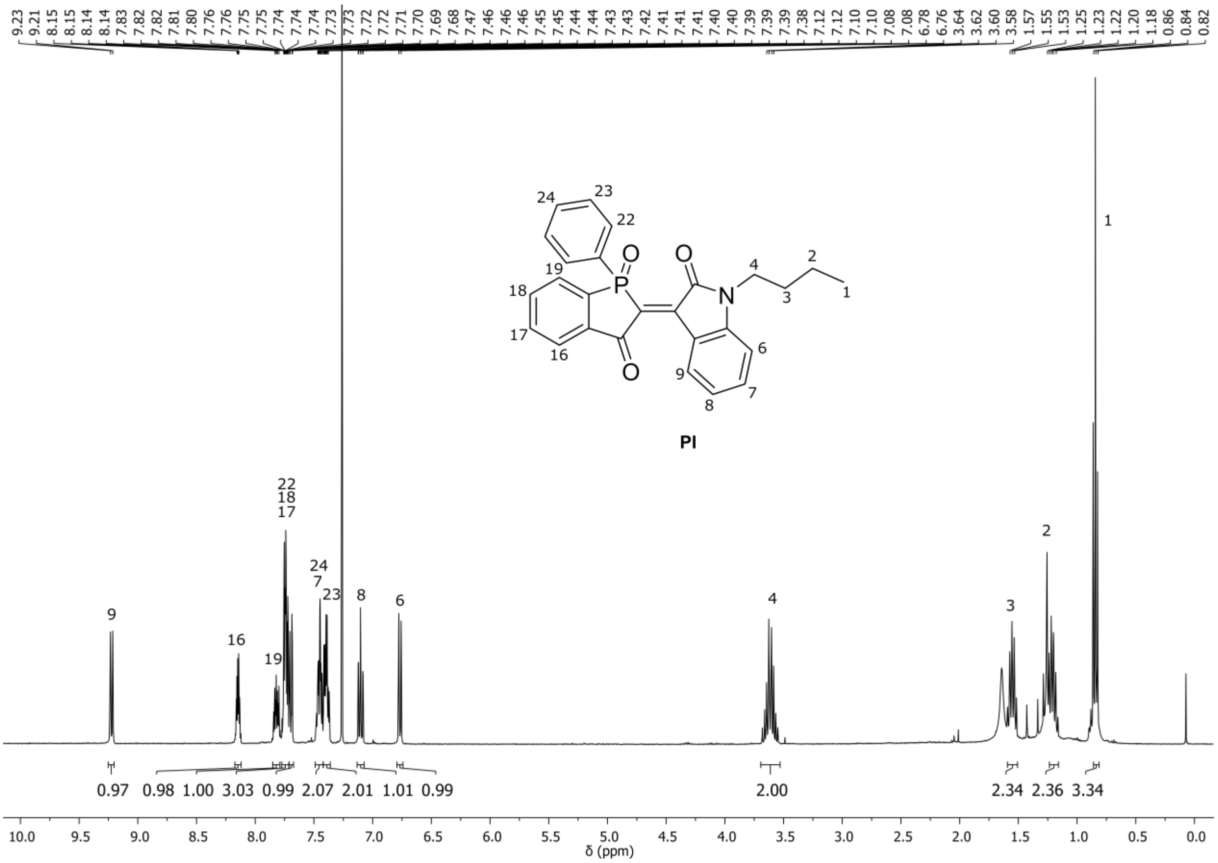

**Figure S5.** $^{1}H$ NMR of **PI** in $CDCl_3$ at 25 °C.

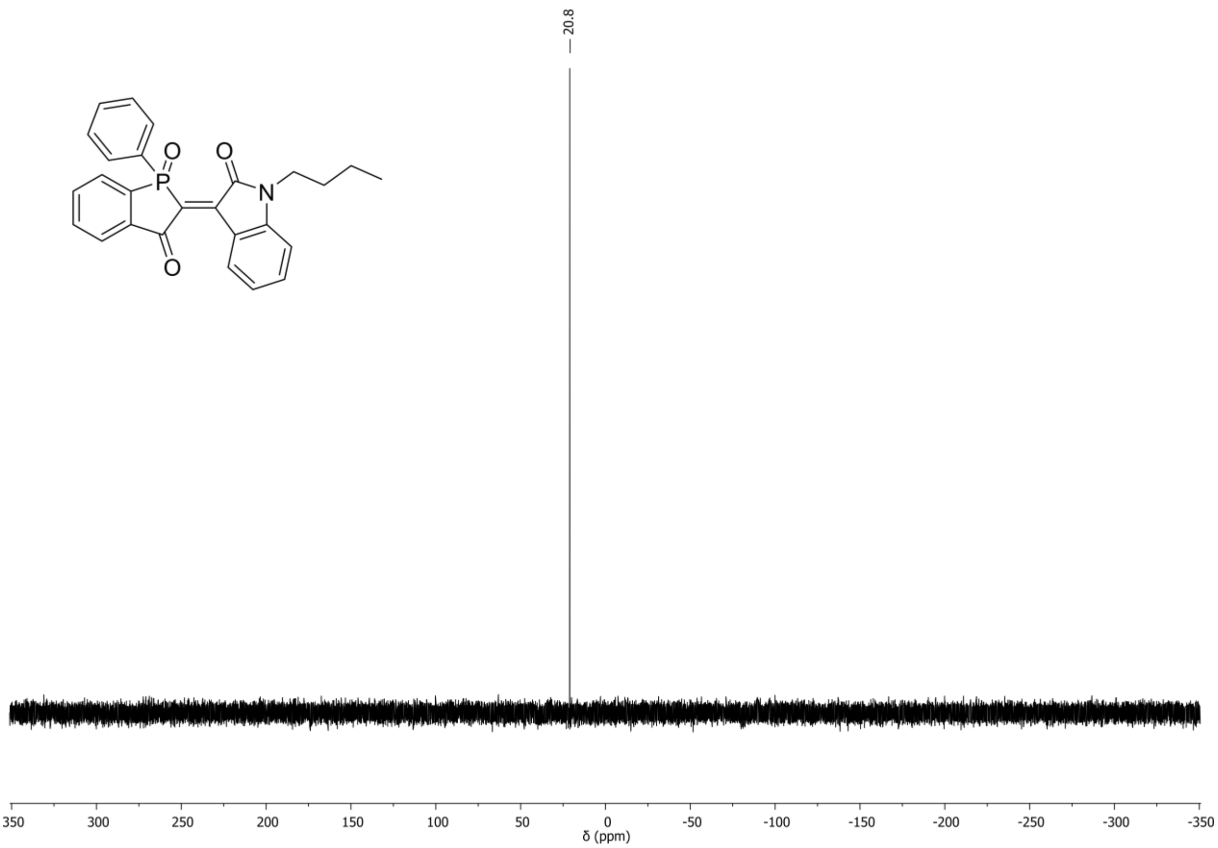

**Figure S6.** $^{31}P$ NMR of **PI** in $CDCl_3$ at 25 °C.

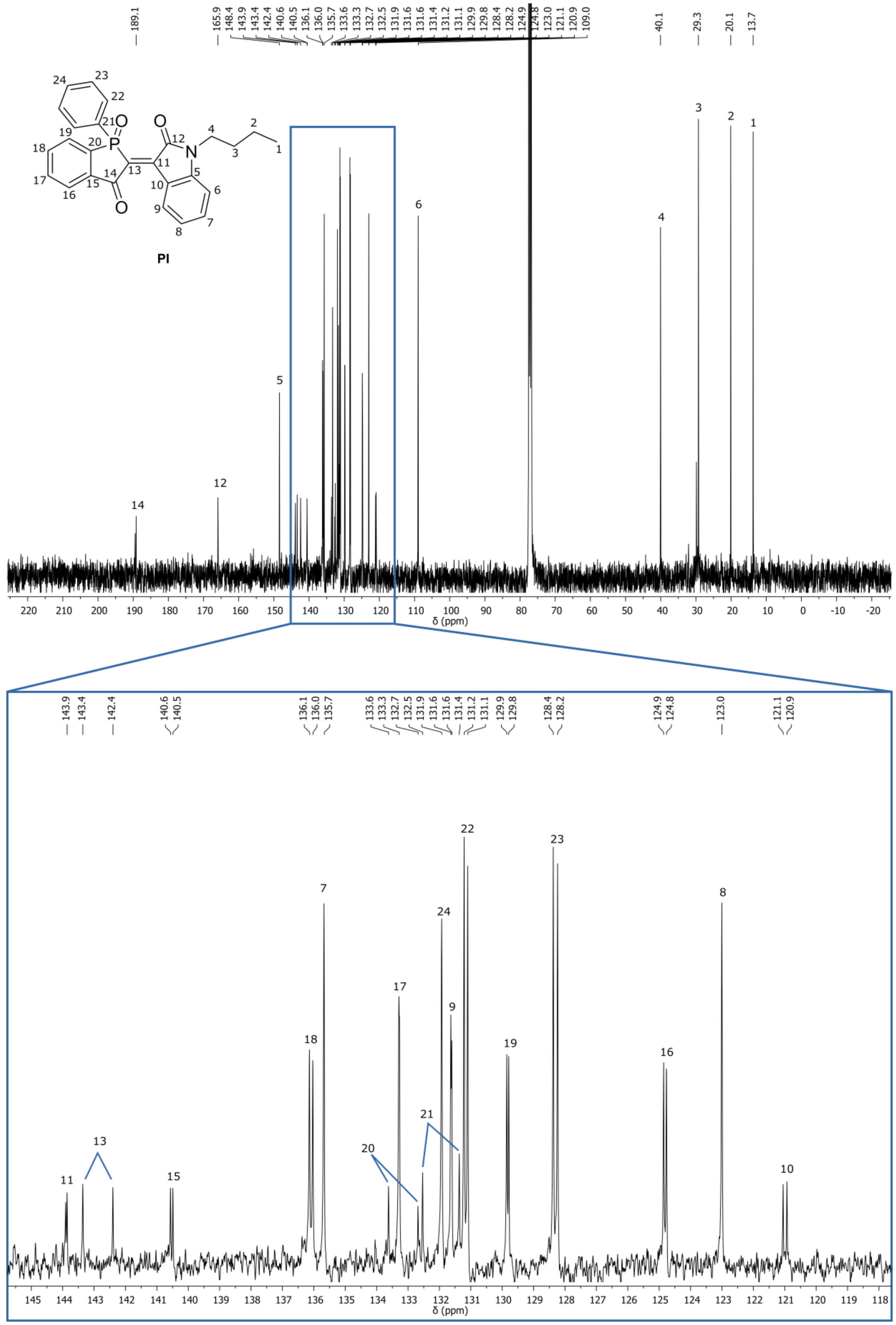

**Figure S7.** $^{13}C\{^{1}H\}$ NMR of **PI** in $CDCl_3$ at 25 °C, and zoom-in of the $^{13}C\{^{1}H\}$ NMR of **PI**. The additional solvent peak belongs to grease (δ = 29.8 ppm).

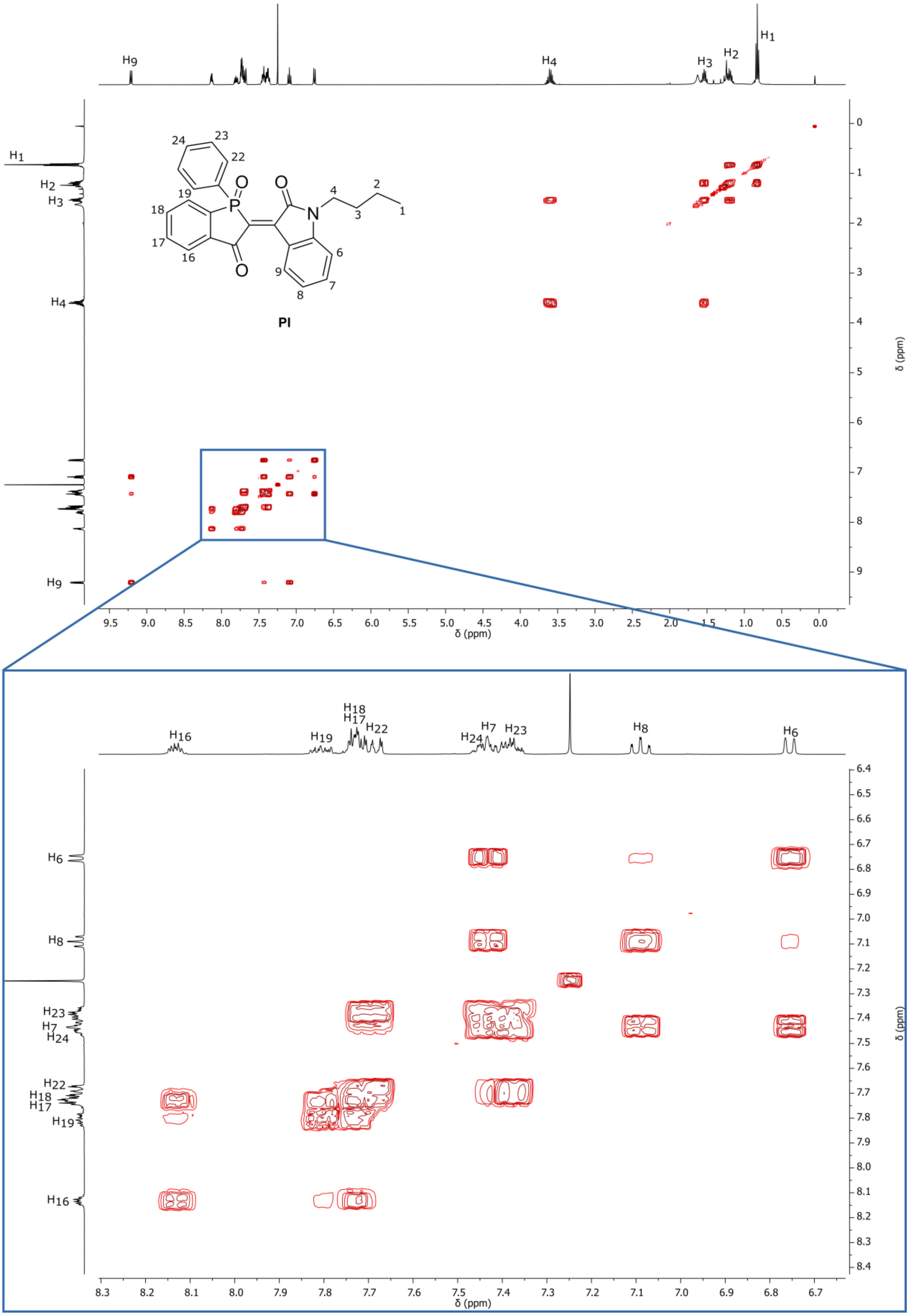


**Figure S8.** The upper spectrum shows the $^{1}$H-$^{1}$H COSY NMR of **PI** in $CDCl_3$ at 25 °C, and the lower spectrum is a zoom in of the $^{1}$H-$^{1}$H COSY NMR of **PI**.

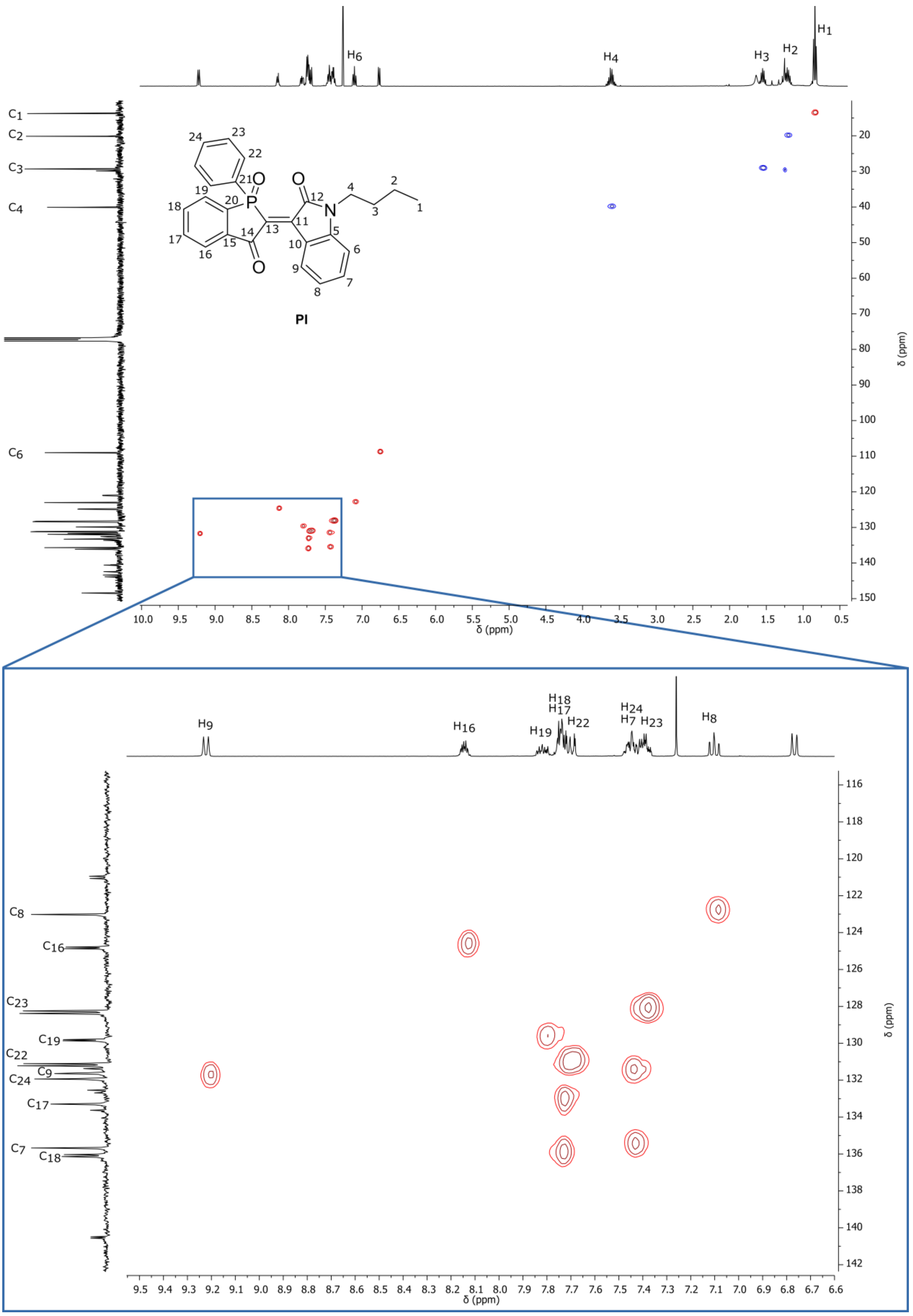


**Figure S9.** The upper spectrum shows the $^{1}H$-$^{13}C\{^{1}H\}$ HSQC NMR of **PI** in $CDCl_3$ at 25 °C, and the lower spectrum is a zoom-in of the $^{1}H$-$^{13}C\{^{1}H\}$ HSQC NMR of **PI**.

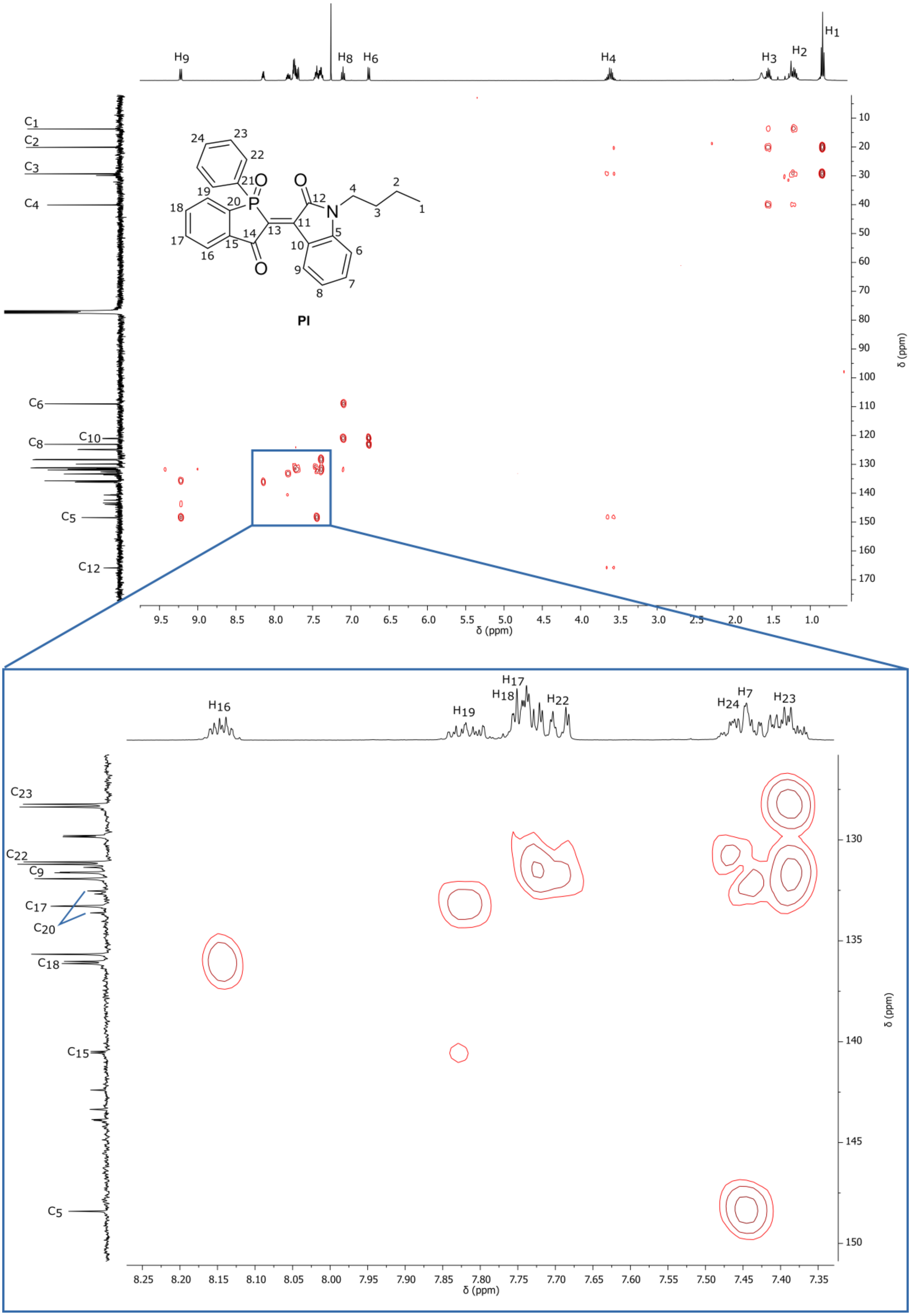


**Figure S10.** The upper spectrum shows the $^1$H-$^{13}$C{$^1$H} HMBC NMR of **PI** in $CDCl_3$ at 25 °C, and the lower spectrum is a zoom-in of the $^1$H-$^{13}$C{$^1$H} HMBC NMR of **PI**.

## 3. HRMS

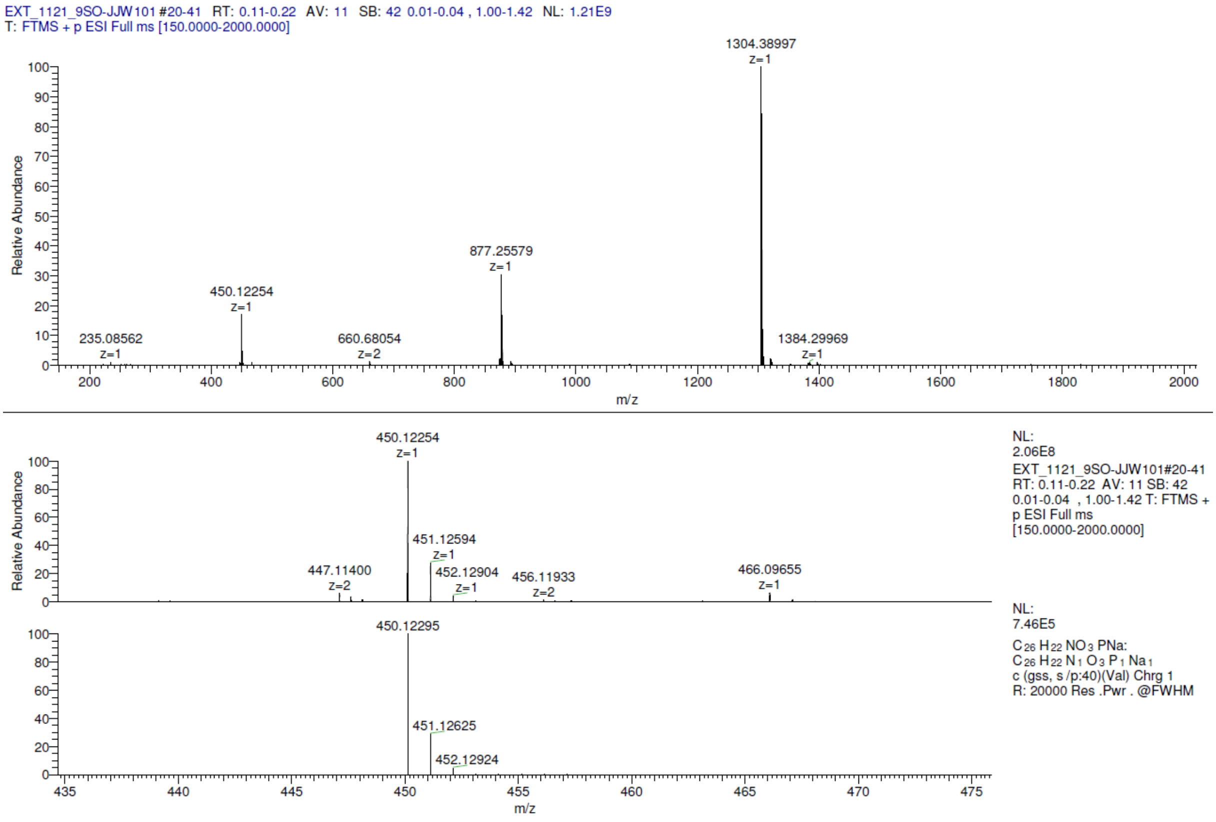


**Figure S11.** The HRMS spectrum of **PI**. The upper trace shows the measured spectrum, the lower trace a simulated spectrum for the specified molecular formula.

## 4. Circular dichroism (CD) Spectroscopy

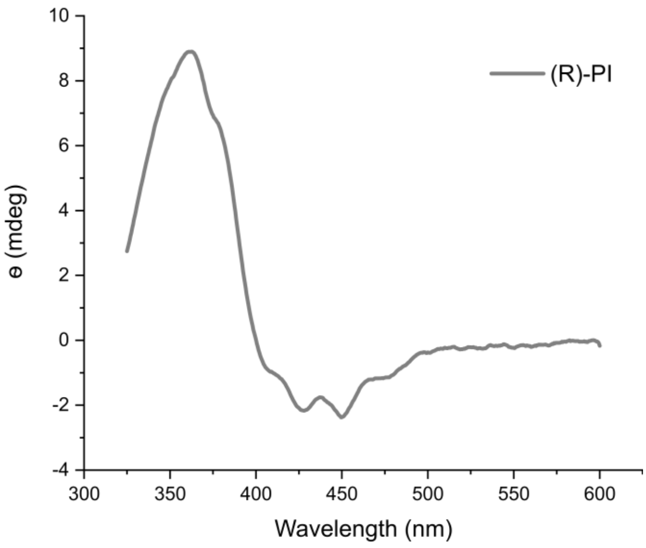


**Figure S12.** The CD-spectrum of **(*R*)-PI** in MeCN at 25 °C.

## 5. UV-Vis Spectra

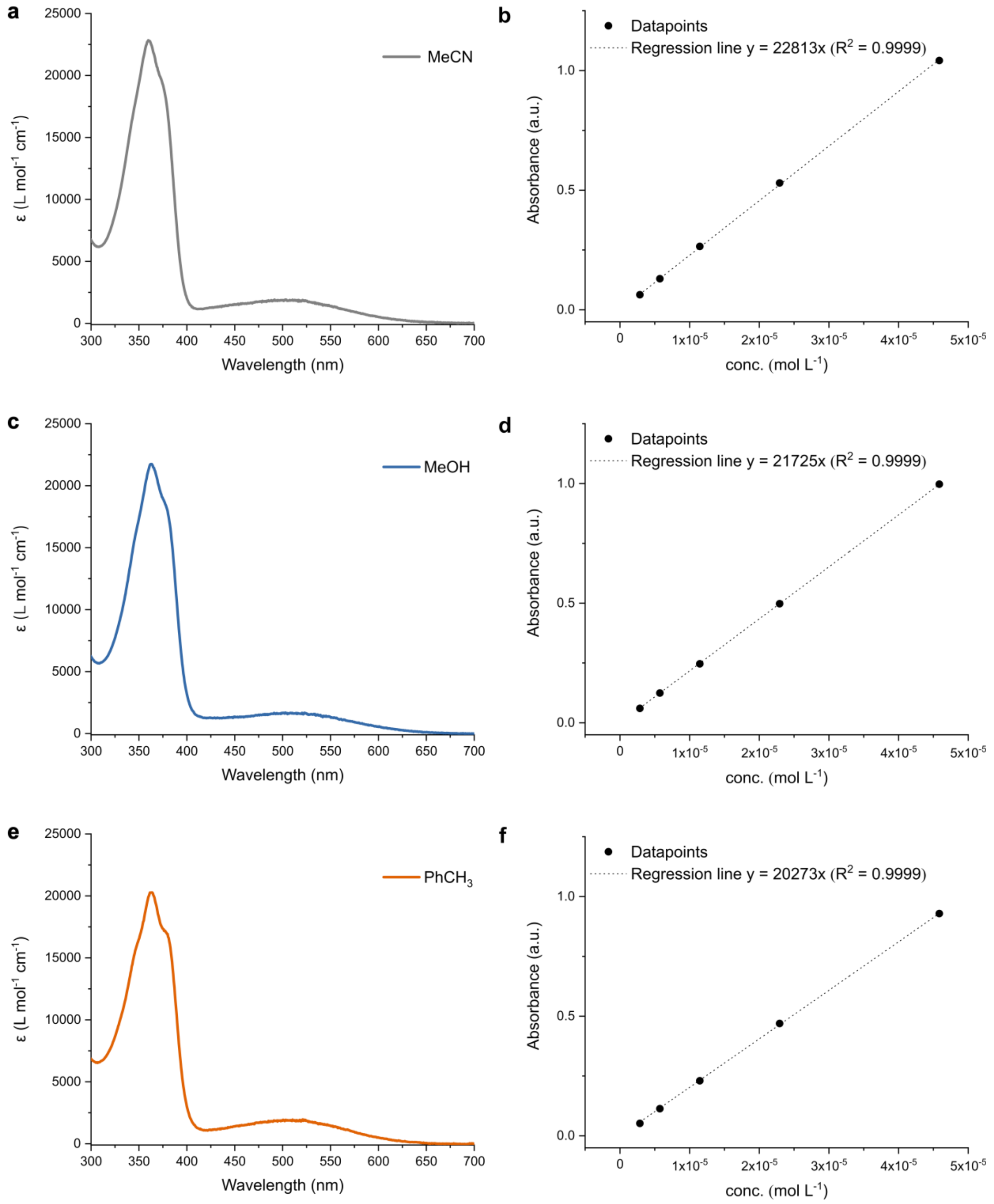


**Figure S13. a** The UV-Vis spectrum of **PI** (4.6 x $10^{-2}$ mM) in MeCN. **b** The regression analysis for the molar absorption coefficient ($\varepsilon$) in MeCN at 360 nm. **c** The UV-Vis spectrum of **PI** (4.6 x $10^{-2}$ mM) in MeOH. **d** The regression analysis for the molar absorption coefficient ($\varepsilon$) in MeOH at 363 nm. **e** The UV-Vis spectrum of **PI** (4.6 x $10^{-2}$ mM) in $PhCH_3$. **f** The regression analysis for the molar absorption coefficient ($\varepsilon$) in $PhCH_3$ at 361 nm.

## 6. Ultrafast Spectroscopy

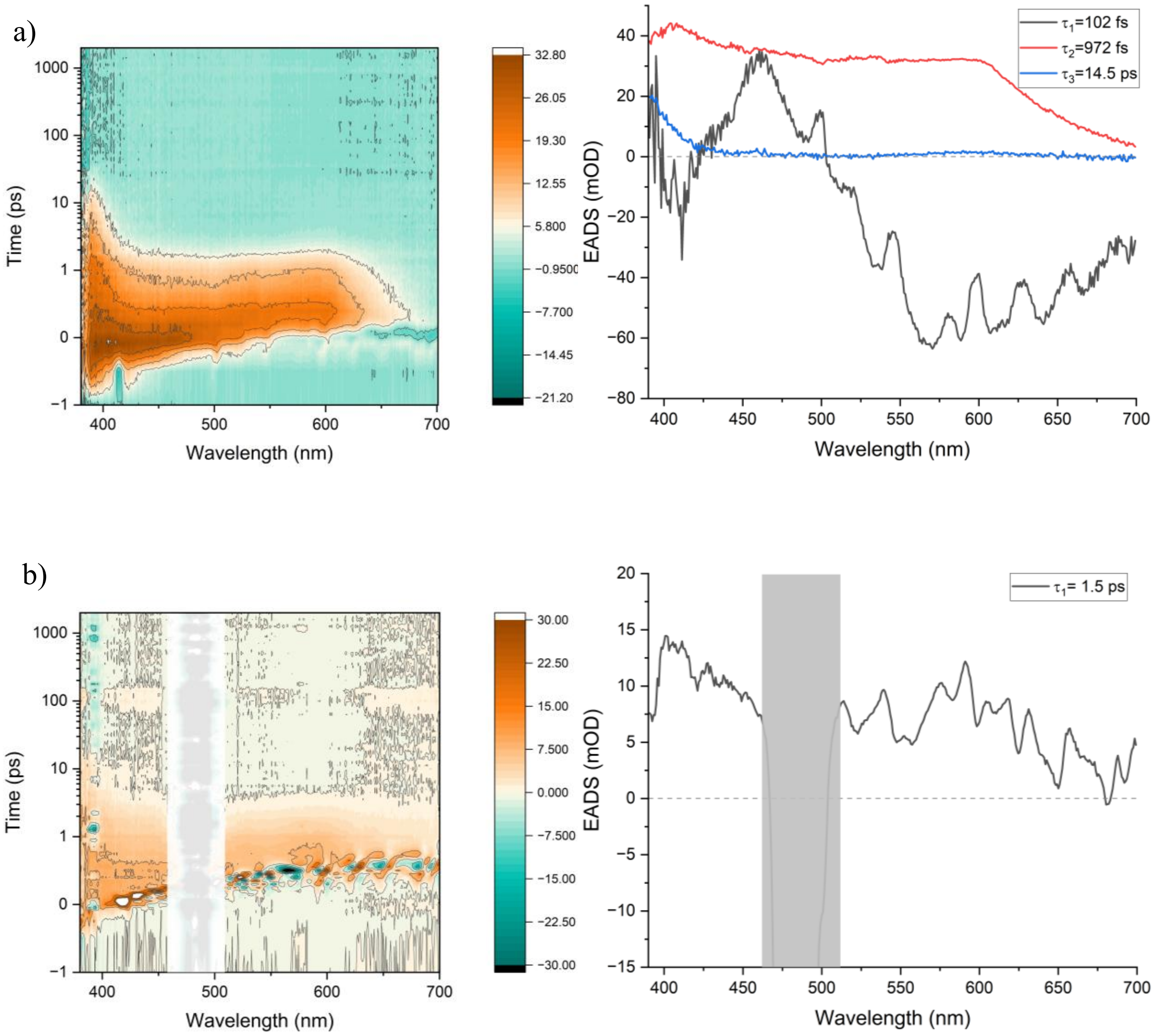


**Figure S14.** Kinetic Heatmap after excitation and the Evolution Associated Difference Spectra (EADS) obtained from a global fit of the femtosecond transient absorption spectral data recorded for **PI** in MeCN with laser pump at a) 375 nm and b) 490 nm. Excitation to the second bright transition with the 375 nm pulse leads to a rapid decay (approximately 100 fs, characterized by stimulated emission centered at 600 nm and excited-state absorption at 450 nm) to a longer-lived state (attributed to $S_1$, $\tau$ = 972 fs) that absorbs broadly in the visible range. This state evolves, forming a transient with a lifetime of $\tau$ = 14.5 ps, attributed to the vibrational cooling of **PI** in its ground state, in its stable form. Excitation to $S_1$ (in b)) leads to a similar broad absorbing transient with a lifetime of 1.5 fs as observed for the excitation at 375 nm ($\tau_2$ in a)). In both cases, no product is formed. The molecule returns to the stable form of **PI**.

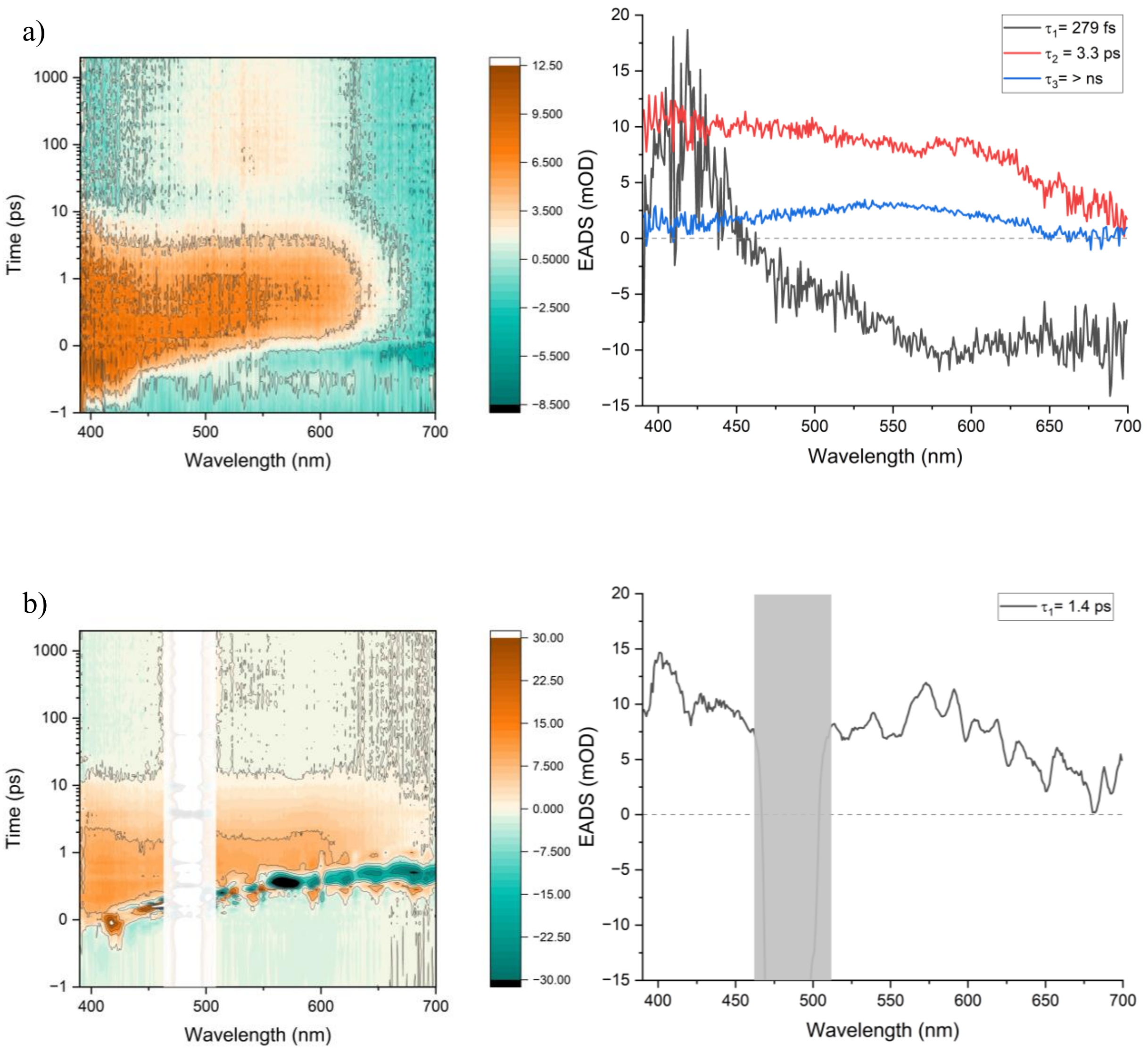


**Figure S15.** Kinetic Heatmap after excitation (chirp-corrected) and the Evolution Associated Difference Spectra (EADS) obtained from a global fit of the femtosecond transient absorption spectral data recorded for **PI** in toluene with laser pump at a) 375 nm and b) 490 nm. Excitation to the second bright transition with the 375 nm pulse leads to a rapid decay (approximately 280 fs, characterized by a broad stimulated emission centered at 600 nm and excited-state absorption at 410 nm), followed by a longer-lived state (attributed to $S_1$, $\tau = 3.3$ ps) that absorbs broadly in the visible. This state evolves, forming a long-lived transient, attributed to the metastable state of **PI** in its ground state, with absorption at 575 nm. Excitation to $S_1$ (in b)) leads to a similar broad absorbing transient with a lifetime of 1.5 fs as observed for the excitation at 375 nm ($\tau_2$ in a)). In the second case, no product is formed, confirming the anti-Kasha behavior of **PI** observed in steady-state spectroscopy.

## 7. X-ray Crystallographic Data

**Table S1.** Refinement details for the X-ray structure of **PI**.

| | | | |
|---|---|---|---|
| Empirical formula | $C_{26}H_{22}NO_3P$, $H_2O$ | Z | 2 |
| Formula weight | 445.43 | $\rho_{calc}$g/cm$^3$ | 1.310 |
| Crystal System | Triclinic | μ/mm$^{-1}$ | 0.155 |
| Space group | *P-1* | GOF | 1.062 |
| T/K | 180 | $2\theta$ range (deg) | 4.828-50.03 |
| a [Å] | 9.5339(9) | Refs collected | 29785 |
| b [Å] | 11.2253(10) | Final *R* indexes [$I$>=$2\sigma(I)$] | $R_1$ = 0.0478, $wR_2$ = 0.1013 |
| c [Å] | 12.0141(11) | Final *R* indexes [*all data*] | $R_1$ = 0.0780, $wR_2$ = 0.1166 |
| α [°] | 82.943(4) | Data/restraints/parameters | 3955/0/293 |
| β [°] | 73.080(4) | Independent reflections | 3955 [$R_{int}$=0.0675, $R_{sigma}$=0.0440] |
| γ [°] | 66.632(4) | Radiation | MoKα (λ=0.71073) |
| V [A$^3$] | 1129.16(18) | F(000) | 468.0 |

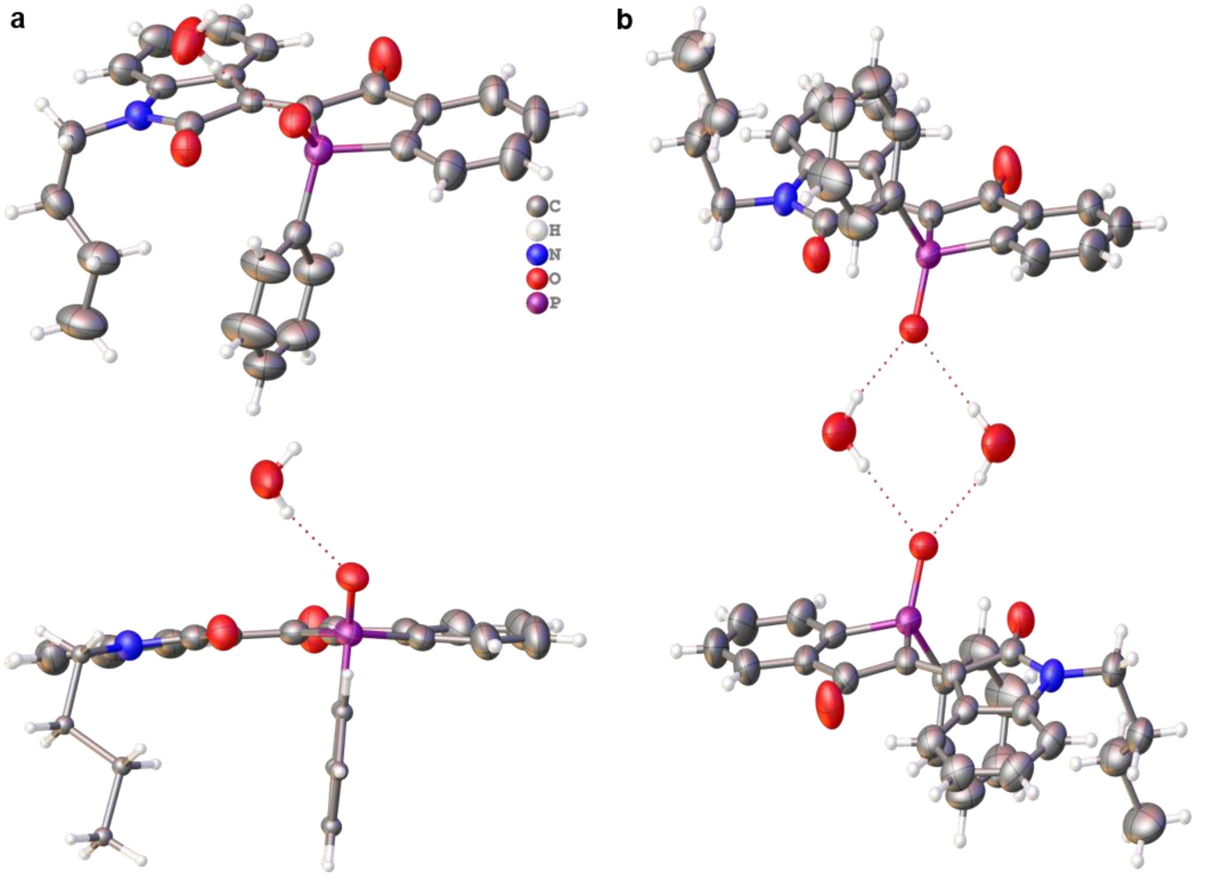


**Figure S16.** Solid state structure of **PI**. **a**,**b** Top and side view of the asymmetric unit plotted at 50% ellipsoid levels (butyl/phenyl groups in wireframe and water solvate omitted for clarity). **B** Hydrogen bonded packing motif in the solid.

## 8. Computational Data

### 8.1 Isomers of PI

The thermodynamically more stable isomer was determined from the crystal structure to be the structure with the phosphine oxide and the oxindole-carbonyl on the same side of the molecule, i.e. ***Z***, as observed previously for indirubin-based photoswitches.[49] For the metastable isomer (***E***), we obtained two different conformers (named ***E1*** and ***E2***) distinguished by the relative orientation of the oxindole-carbonyl and phosphoryl groups (**Figure S17**).

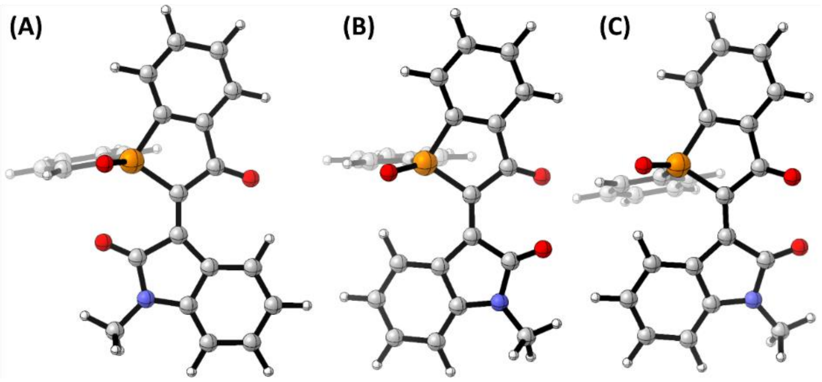


**Figure S17.** The optimized geometries (r$^2$SCAN-3c) of (A) ***Z***, (B) ***E1***, and (C) ***E2***.

The difference between ***E1*** and ***E2*** lies in the relative positions of the carbonyl groups: in ***E1***, the carbonyl and the phosphoryl group of the benzophospholane are on different sides of the oxindole plane, while in ***E2***, the carbonyl and the phosphoryl group of the benzophospholane are on the same side (**Figure S18**). The transition state between the metastable states (***$TS_{E1,E2}$***) shows a more planar structure compared to ***E1*** and ***E2***.

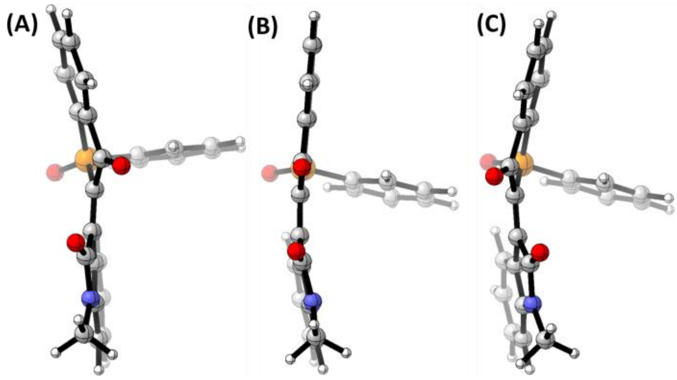


**Figure S18.** The optimized geometries (r$^2$SCAN-3c) of (A) ***E1***, (B) ***$TS_{E1,E2}$***, and (C) ***E2***.

For both transition states of double-bond isomerization (***TS**$_{Z,E1}$* and ***TS**$_{Z,E2}$*), the oxindole unit is perpendicular to the benzophospholane unit (**Figure S19**). To distinguish between both transition states, the relative position of the carbonyl group on the oxindole unit is indicative: for the TS connecting ***Z*** and ***E1*** (***TS**$_{Z,E1}$*), the oxindole carbonyl group is on the same side as the phosphoryl group of the benzophospholane unit, while for the TS connecting ***Z*** and ***E2*** (***TS**$_{Z,E2}$*), the oxindole carbonyl group is on the opposite side compared to the phosphoryl group of the benzophospholane unit.

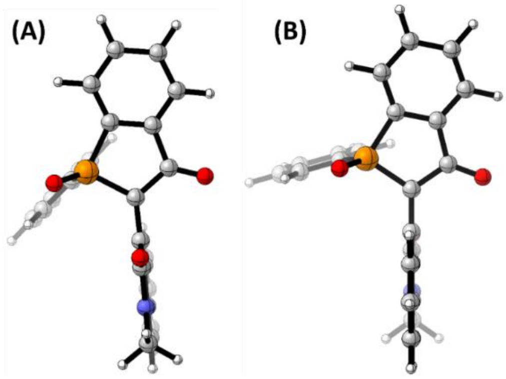


**Figure S19.** The optimized structures ($r^2$SCAN-3c) of (A) ***TS**$_{Z,E1}$* and (B) ***TS**$_{Z,E2}$*.

**8.2 Energy Barriers and Relative Gibbs Free Energies**

Figure **S30** shows the ground-state energy profile of the optimized minima and transition states of **PI**. The trend in relative energies is consistent for all other functionals as well (**Table S2**, **Table S3**, **Table S4**,

**Table S5**).

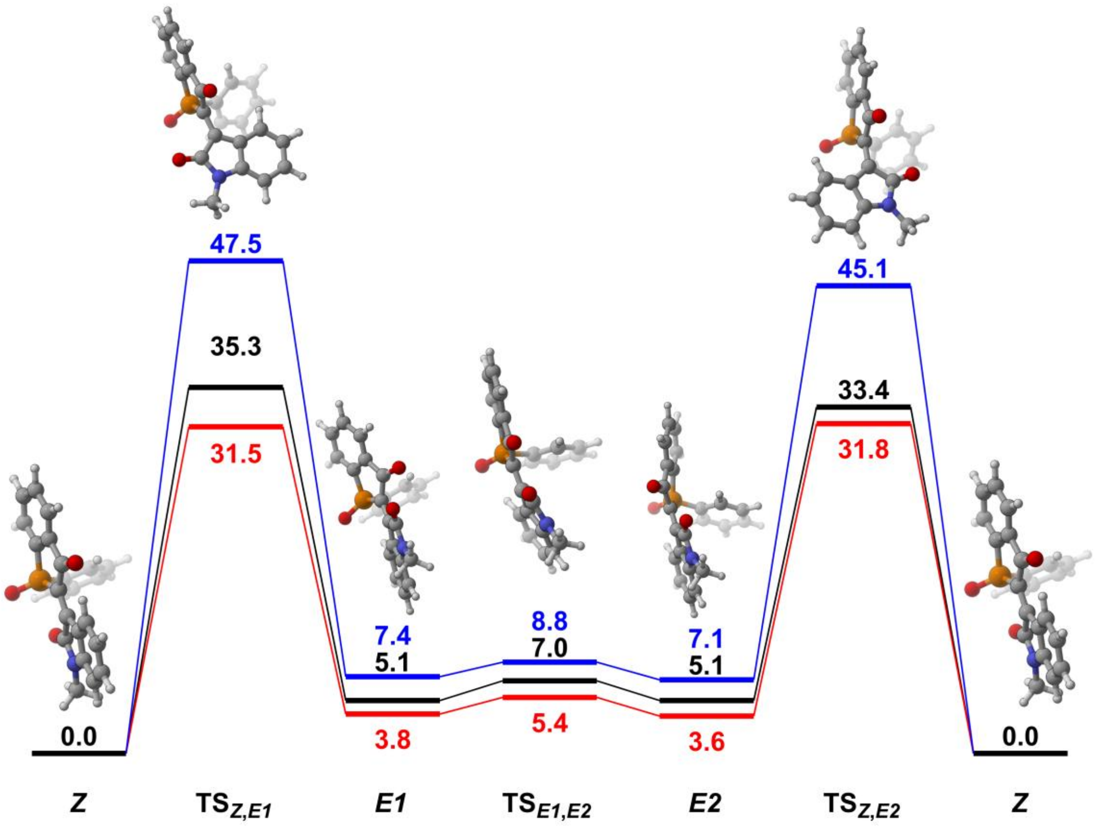


**Figure S20.** Energy diagram of **PI** with acetonitrile (red, ωB97X-V/def2-TZVPP/CPCM($CH_3CN$)//$r^2$SCAN-3c/CPCM($CH_3CN$)), in gas phase (black, ωB97X-V/def2-TZVPP//$r^2$SCAN-3c), and gas phase with MRSF-TDDFT (blue, MRSF-TDDFT/BH&HLYP/6-31G(d)/6-311G(d)). ***Z*** = stable state; ***E1*** = metastable state 1; ***E2*** = metastable state 2; $TS_{a,b}$ = the transition state between a and b. Energies are Gibbs free energies, except those obtained from MRSF-TDDFT, and are given in kcal/mol. The geometries depicted are from the calculations with the acetonitrile solvent model.

Notably, in cyclohexane and the gas phase (to a smaller extent), the energy of $\boldsymbol{TS_{Z,E1}}$ is relatively high ($\Delta G^{\ddagger}$ = 38.6 kcal/mol) compared to that of $\boldsymbol{TS_{Z,E2}}$ ($\Delta G^{\ddagger}$ = 33.2 kcal/mol), while, for all other solvents, the energies of the transition states are the same within the error of the calculation. This trend is observed for all functionals (see **Table S2**, **Table S3**, **Table S4**,

**Table S5**).

**Table S2.** Relative Gibbs free energies (kcal/mol) calculated with M06-2X-D3ZERO/def2-TZVPP//r$^2$SCAN-3c. ***Z*** = stable state; ***E1*** = metastable state 1; ***E2*** = metastable state 2; $TS_{a,b}$ = the transition state between *a* and *b*.

| | **Gas phase** | **Acetonitrile** | **Cyclohexane** | **Methanol** | **Toluene** |
|---|---|---|---|---|---|
| ***Z*** | 0.0 | 0.0 | 0.0 | 0.0 | 0.0 |
| $\boldsymbol{TS_{Z,E1}}$ | 38.6 | 35.0 | 41.9 | 35.0 | 37.5 |
| ***E1*** | 5.2 | 3.9 | 5.1 | 3.9 | 5.1 |
| $\boldsymbol{TS_{E1,E2}}$ | 7.1 | 5.6 | 7.0 | 5.6 | 6.7 |
| ***E2*** | 5.3 | 3.8 | 5.1 | 3.8 | 5.1 |
| $\boldsymbol{TS_{Z,E2}}$ | 36.7 | 35.3 | 36.6 | 35.3 | 36.6 |

**Table S3.** Relative Gibbs free energies (kcal/mol) calculated with PW6B95-D4/def2-TZVPP//r$^2$SCAN-3c. ***Z*** = stable state; ***E1*** = metastable state 1; ***E2*** = metastable state 2; $TS_{a,b}$ = the transition state between *a* and *b*.

| | **Gas phase** | **Acetonitrile** | **Cyclohexane** | **Methanol** | **Toluene** |
|---|---|---|---|---|---|
| ***Z*** | 0.0 | 0.0 | 0.0 | 0.0 | 0.0 |
| $\boldsymbol{TS_{Z,E1}}$ | 37.0 | 33.4 | 40.3 | 33.5 | 36.0 |
| ***E1*** | 5.5 | 4.0 | 5.3 | 4.0 | 5.4 |
| $\boldsymbol{TS_{E1,E2}}$ | 7.8 | 6.1 | 7.6 | 6.2 | 6.8 |
| ***E2*** | 5.7 | 4.1 | 5.5 | 4.1 | 5.5 |
| $\boldsymbol{TS_{Z,E2}}$ | 35.1 | 33.6 | 34.9 | 33.6 | 34.9 |

**Table S4.** Relative Gibbs free energies (kcal/mol) calculated with ωB97X-V/def2-TZVPP//r$^2$SCAN-3c. ***Z*** = stable state; ***E1*** = metastable state 1; ***E2*** = metastable state 2; $TS_{a,b}$ = the transition state between *a* and *b*.

| | **Gas phase** | **Acetonitrile** | **Cyclohexane** | **Methanol** | **Toluene** |
|---|---|---|---|---|---|
| ***Z*** | 0.0 | 0.0 | 0.0 | 0.0 | 0.0 |
| $\boldsymbol{TS_{Z,E1}}$ | 35.3 | 31.5 | 38.6 | 31.5 | 37.8 |
| ***E1*** | 5.1 | 3.8 | 5.0 | 3.8 | 5.1 |
| $\boldsymbol{TS_{E1,E2}}$ | 7.0 | 5.4 | 6.9 | 5.4 | 8.7 |
| ***E2*** | 5.1 | 3.6 | 4.9 | 3.6 | 4.9 |
| $\boldsymbol{TS_{Z,E2}}$ | 33.4 | 31.8 | 33.2 | 31.8 | 37.9 |

**Table S5.** Relative Gibbs free energies (kcal/mol) calculated with ωB97X-D3/def2-TZVPP//r$^2$SCAN-3c. ***Z*** = stable state; ***E1*** = metastable state 1; ***E2*** = metastable state 2; $TS_{a,b}$ = the transition state between *a* and *b*.

| | **Gas phase** | **Acetonitrile** | **Cyclohexane** | **Methanol** | **Toluene** |
|---|---|---|---|---|---|
| ***Z*** | 0.0 | 0.0 | 0.0 | 0.0 | 0.0 |
| $\boldsymbol{TS_{Z,E1}}$ | 33.5 | 29.9 | 36.9 | 29.9 | 32.5 |
| ***E1*** | 4.8 | 3.5 | 4.7 | 3.5 | 4.8 |
| $\boldsymbol{TS_{E1,E2}}$ | 6.9 | 5.3 | 6.7 | 5.3 | 6.3 |
| ***E2*** | 4.8 | 3.3 | 4.6 | 3.3 | 4.6 |
| $\boldsymbol{TS_{Z,E2}}$ | 31.7 | 30.3 | 31.6 | 30.3 | 31.7 |

## 8.3 Potential Energy Surface, Minima and Conical Intersections

We performed a two-dimensional scan along the rotation dihedral ($\alpha$) and the C-N bond ($r$) (**Figure S21**) for the first excited state (**Figure S22**). The main geometries of interest (minima, transition states, and conical intersections) obtained with MRSF-TDDFT calculations are depicted in **Figure S23**. We were not able to obtain either of the *E*-isomers in the triplet state, since geometry optimization led to one of the orthogonal geometries ($T_1^{Orth\text{-}1}$ and $T_1^{Orth\text{-}2}$). We obtain an approximation of the relative energy from the scan performed along the dihedral (see **Figure S22**): the *E*-isomer is 10 kcal/mol higher in energy than the *Z*-isomer in the triplet state.

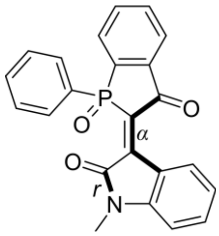


**Figure S21.** The rotation dihedral angle ($\alpha$) and C-N bond distance ($r$) of **PI** were used for optimization of the constrained geometries.

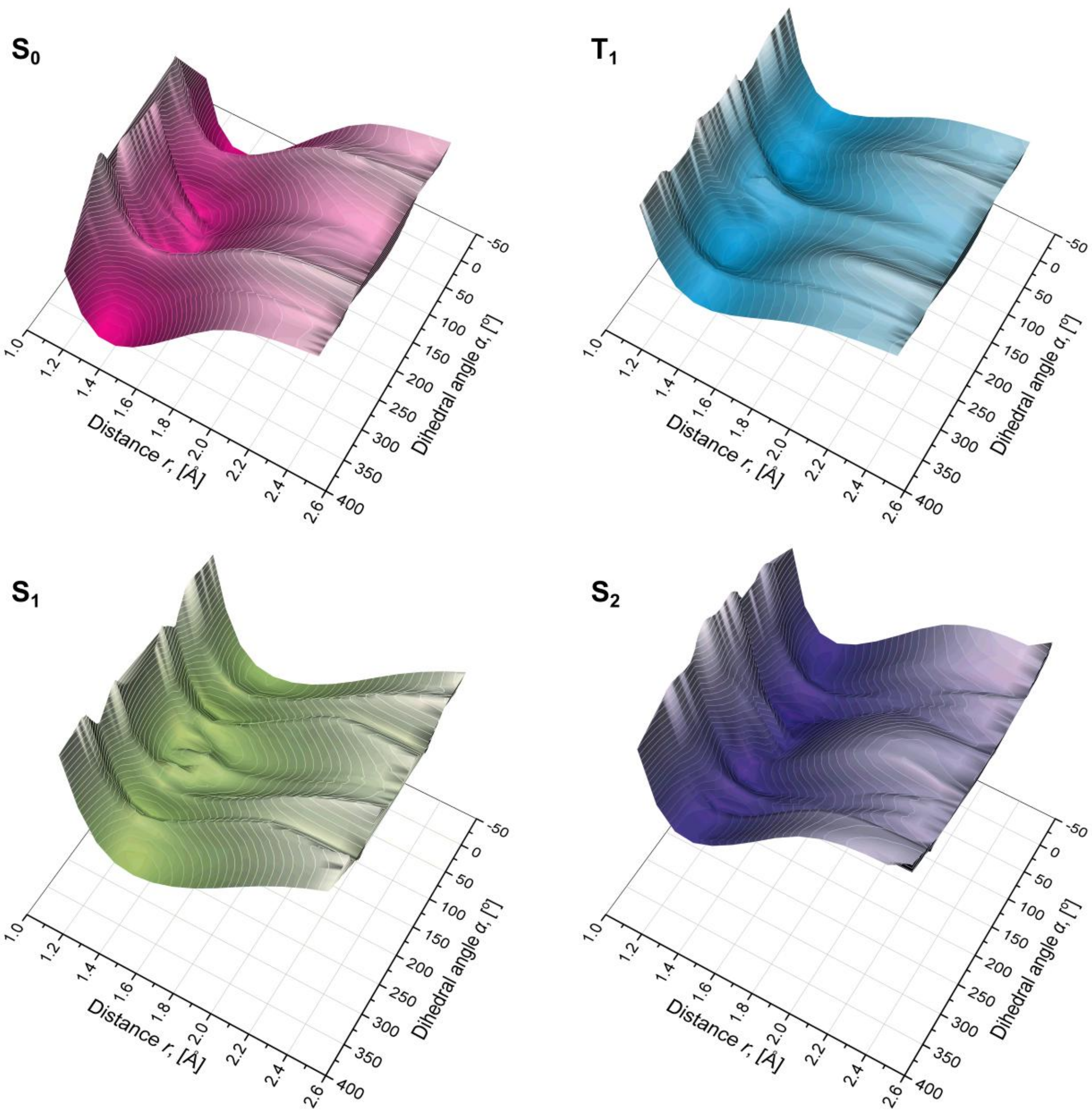


**Figure S22.** Potential energy surfaces of **PI** in the $S_0$, $T_1$, $S_1$, and $S_2$ states obtained by single-point calculations at the MRSF-TDDFT/BH&HLYP/6-31G(d)/6-311G(d) level of the optimized constrained geometries obtained at the $S_1$ state.

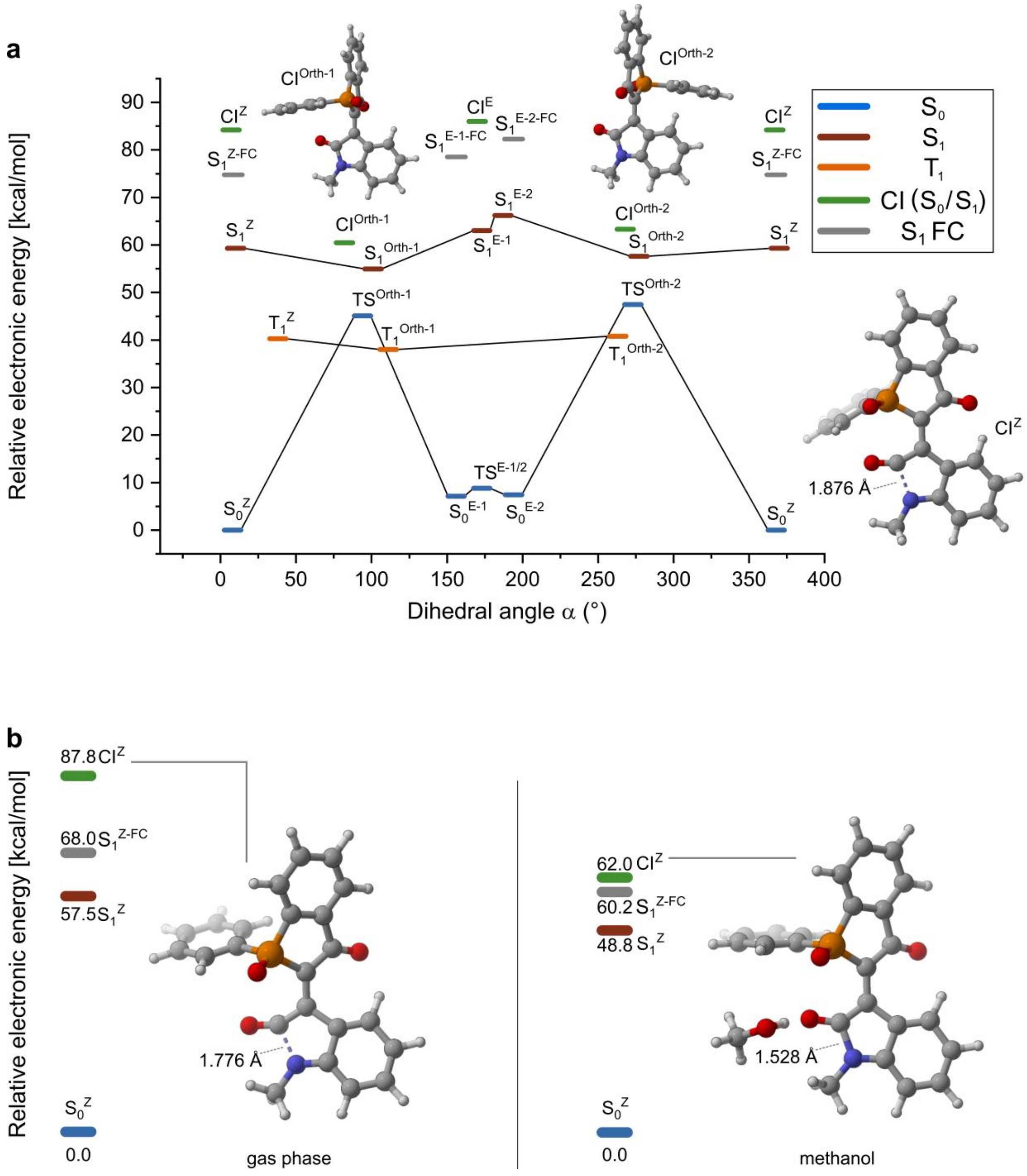


**Figure S23. a**. Main points of interest for **PI** calculated at the MRSF-TDDFT-BH&HLYP/6-31G(d)/6-311G(d) level. $S_0$ (blue), $S_1$ (red), $T_1$ (orange), conical intersections between $S_0$ and $S_1$ (green), and Franck-Condon points at $S_0$ geometries with $S_1$ energies (grey). All values are optimized, apart from the single point of the Franck-Condon point (energy of the $S_1$ at the $S_0$ optimized geometry). Energies are given in kcal/mol. In the inset, the geometry of $CI^Z$ which connects $S_1$ and $S_0$ near the Franck-Condon point. **b**. Effect of solvation on the relative energy and geometry of $CI^Z$. The values are obtained at the SF-DFT-BH&HLYP/def2-SVP level of theory in the gas phase or in SMD(methanol), with an explicit methanol molecule. From the energies it is clear that polarization in the solvent and direct interaction with the molecule of the solvent stabilizes $CI^Z$ considerably. The elongation of the C(O)-N bond is affected by the medium. $CI^Z$ is reached at shorter bond lengths in polar solvents than in the gas phase.

### 8.4 Protonation of Phosphaindirubin

We have investigated the protonation of **PI** on two different sites, the phosphoryl group (**POH**) and the carbonyl group (**COH**) of the benzophospholane unit (**Figure S24**), with a solvation model (**Figure S25**), with a solvation model and three explicit water molecules (**Figure S26**), and in gas phase with MRSF-TDDFT (**Figure S27**).

Overall, **POH-*Z*** is energetically the lowest state, and its *E*-isomer is 12.5 kcal/mol higher in energy. Notably, while protonation of the carbonyl group, i.e. **COH**, results in a significant increase in energy for the *Z*-isomer, its *E*-isomer is relatively low in energy (4.3 kcal/mol *vs* **POH-*Z***), similar to the calculated energy difference for the unprotonated species (see **Figure S20**). The relative stabilisation of **COH-*Z*** is likely due to the formation of a hydrogen bond between the protonated carbonyl group of the benzophospholane unit and the carbonyl group of the oxindole unit. The inclusion of three molecules of water results in a similar scheme of relative energies, with the notable exception that we were not able to obtain the optimised geometry for **COH-*Z*•3H$_2$O**, which instead formed another **POH-*Z*•3H$_2$0** after optimisation.

The P=O bond in the protonated species **POH-*Z*** is slightly elongated compared to that in **PI-*Z***, but it is not as long as expected for a single bond (**Table S7**).

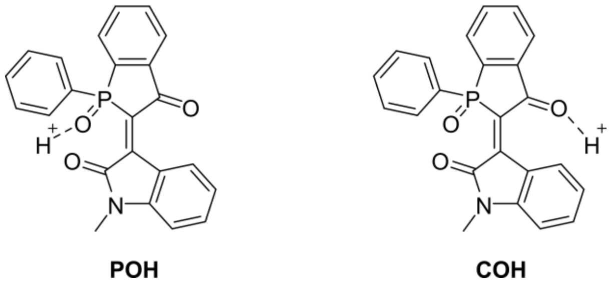


**Figure S24.** The two protonation sites of **PI** that are included in the computational investigation.

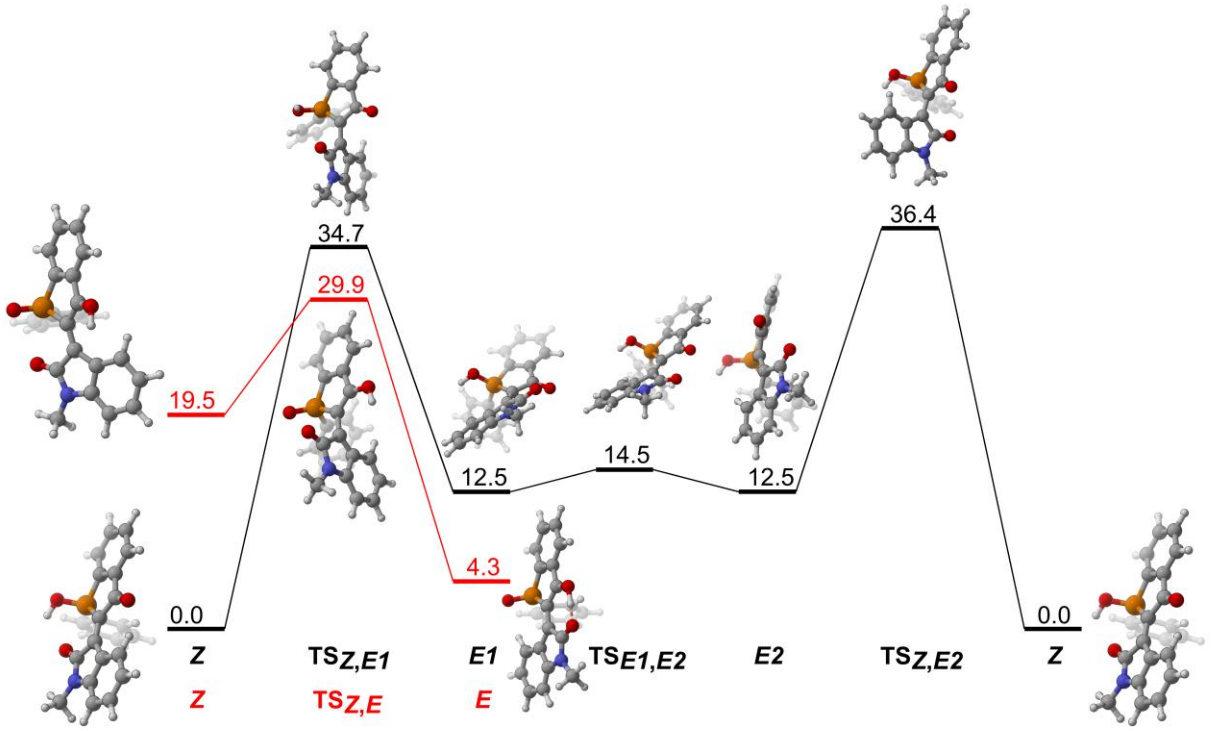


**Figure S25.** Relative Gibbs free energies (kcal mol$^{-1}$) calculated for **POH** (black) and **COH** (red) at the ωB97X-V/def2-TZVPP/CPCM($CH_3CN$)//r$^2$SCAN-3c/CPCM($CH_3CN$) level. For **COH**, we only obtained one *E*-isomer.

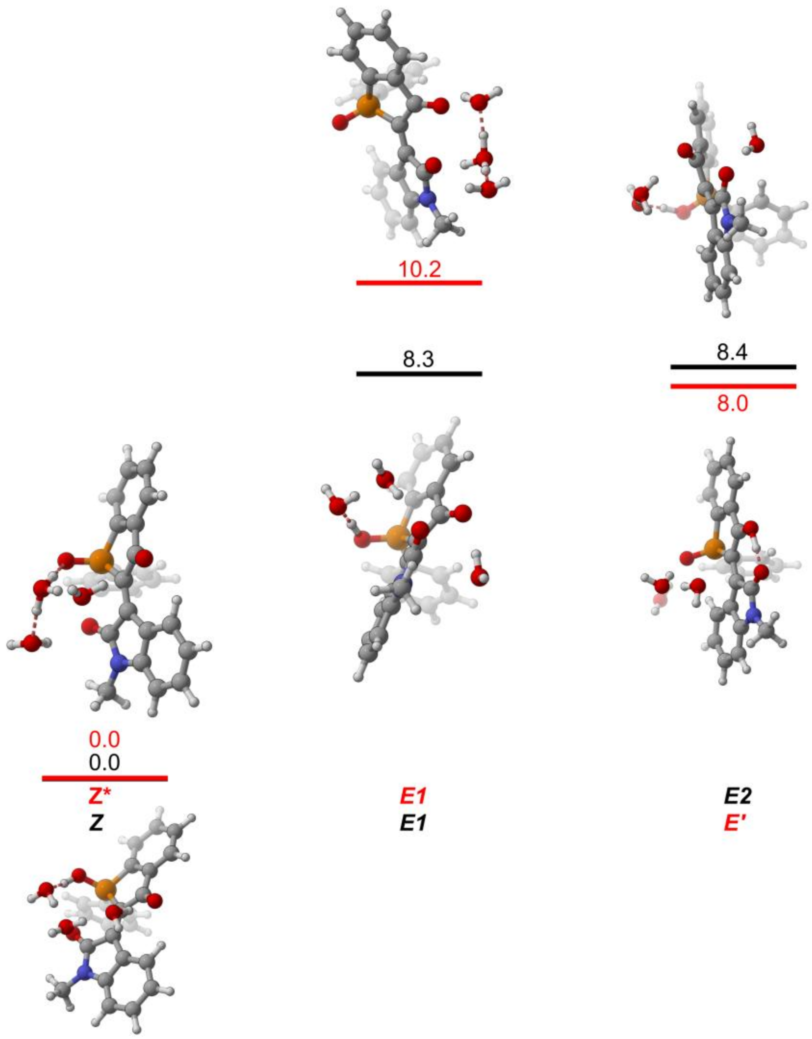


**Figure S26.** Relative Gibbs free energies (kcal mol$^{-1}$) calculated for **PI-POH** (black) and **PI-COH** (red) with three molecules of water at the ωB97X-V/def2-TZVPP/CPCM($CH_3CN$)//r$^2$SCAN-3c/CPCM($CH_3CN$) level. ***Z****: optimization of the PI-COH Z-isomer resulted in **POH-*Z***; ***E´***: *E*-isomer of **COH** adopts a flat geometry due to a bridging hydrogen bond.

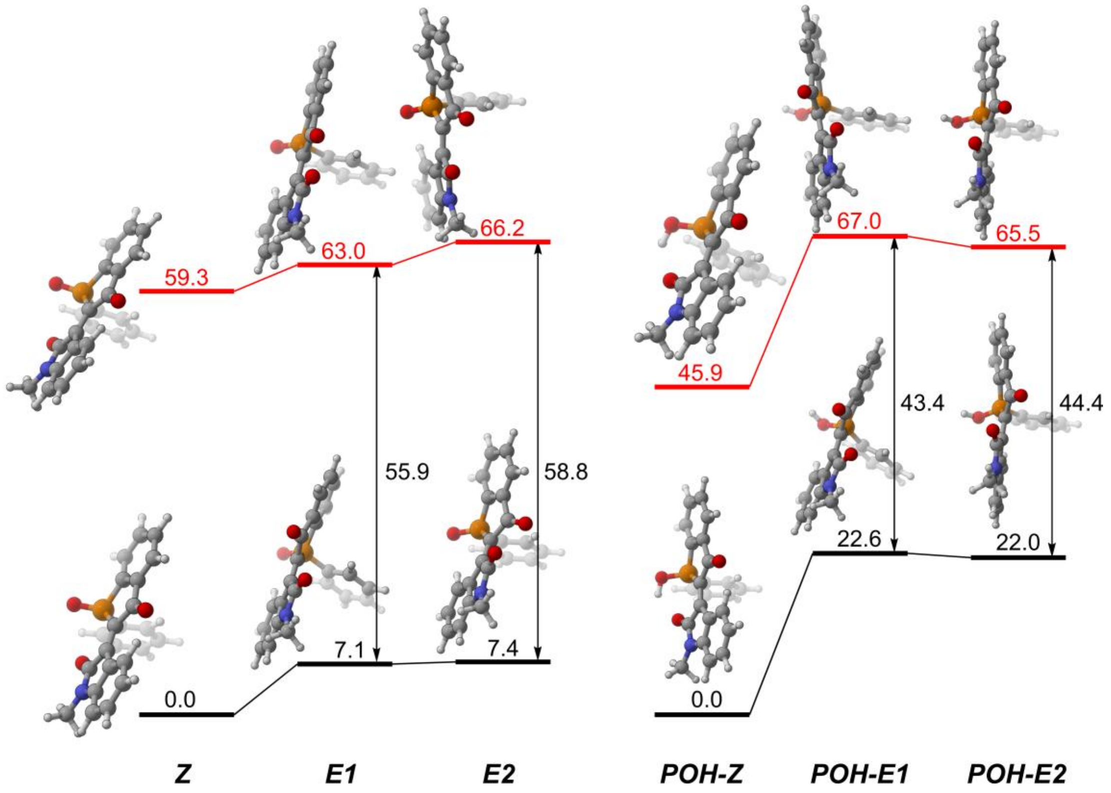


**Figure S27.** Relative electronic energies for the ground state (black) and first excited state (red) of the *Z*- and two *E*-isomers of (left) **PI** and (right) **POH** calculated at the MRSF-TDDFT-BH&HLYP/6-31G(d)/6-311G(d) level. Protonation decreases the $S_0/S_1$ energy difference by 12-15 kcal/mol (21-26%). C=C bond lengths of first excited state: ***Z***, 1.384 Å; **POH-*Z***, 1.393 Å.

**Table S6.** Bond lengths of C=C (Å) and CO-N (Å) for unprotonated **PI**, protonated ***Z*-PI-POH,** and protonated ***Z*-PI-POH** with an explicit MeOH molecule. Geometries optimised at the MRSF-TDDFT-BH&HLYP/6-31G(d)/6-311G(d) and SF-TDDFT-BH&HLYP/def2-SVP/CPCM(methanol).

| Method | **State** | **PI** | **PI-POH** | **PI-POH MeOH** | **PI** | **PI-POH** | **PI-POH MeOH** |
|---|---|---|---|---|---|---|---|
| | | C=C | | | CO-N | | |
| SF | $S_1$ | 1.409 | 1.405 | 1.406 | 1.444 | 1.429 | 1.428 |
| | $S_0$ | 1.377 | 1.374 | 1.374 | 1.351 | 1.338 | 1.335 |
| MRSF | $S_1$ | 1.384 | 1.393 | | 1.484 | 1.448 | |
| | $S_0$ | 1.357 | 1.357 | | 1.360 | 1.334 | |

**Table S7.** Bond length of P=O (Å) for unprotonated and protonated isomers of **PI**. Geometries optimised at the r$^2$SCAN-3c/CPCM($CH_3CN$) level. **E´*: *E*-isomer of **COH** adopts a flat geometry due to a bridging hydrogen bond (see **Figure S26**).

| P=O bond length (Å) | **PI** | **PI•3H₂O** | **POH** | **POH•3H₂O** | **COH** | **COH•3H₂O** |
|---|---|---|---|---|---|---|
| *Z* | 1.51 | 1.52 | 1.60 | 1.55 | 1.50 | 1.53 |
| *E*1 | 1.50 | 1.51 | 1.59 | 1.56 | 1.50 | 1.50 |
| *E*2 | 1.51 | 1.51 | 1.60 | 1.56 | - | 1.51 (***E´***)* |

### 8.5 TD-DFT Calculations: Simulated UV-Vis Absorption Spectra

To determine the most suitable functional and basis set, the simulated UV-Vis absorption spectra (15 roots) were compared to experimental data. The following five functionals were used with the ma-def2-TZVPP basis set: BHHLYP, CAM-B3LYP, M06-2X, PBE*0*, and *ω*B97X-D3. The simulated UV-Vis absorption spectra for each functional were plotted as $\varepsilon$ vs. λ, assuming a Gaussian band shape (characterized by a standard deviation $\sigma = 0.4$ eV), expanding each transition obtained by the calculation.

The calculations resulted consistently in three bands between 200-600 nm (**Figure S28**). The position of the bands obtained with the PBE0 functional was qualitatively similar to those in the experimental data; however, the number and types of transitions associated with the second bright transition did not match the other functionals nor the results obtained with MRSF-TDDFT (see below). Therefore, PBE0 was ruled out. The simulated UV-Vis spectra calculated with the remaining functionals (BHHLYP, CAM-B3LYP, M06-2X and ωB97X-D3) were similar. The bands calculated using M06-2X were shifted to lower energies compared to BHHLYP, CAM-B3LYP and ωB97X-D3, which matched the experimental data. Therefore, M06-2X with the ma-def2-TZVPP basis set was chosen for further calculations. This basis set, however, led to convergence issues for some molecules, which is why we resorted to using the basis set def2-TZVPP.

A comparison of the simulated UV-Vis spectra of the stable (***Z***) and both metastable (***E1*** and ***E2***) states are shown in **Figure S29**. In **Figure S30** to **Figure S35**, the influence of the solvents on the simulated UV-Vis spectra is depicted. The spectra in the gas phase differ from those with solvents, which are characterised by more probable transitions (higher oscillator strengths) and are bathochromically shifted. However, by changing the polarity of the solvent, the position of the bands remained nearly the same, with only a small red shift of the polar solvents compared to the non-polar solvents. Changes in polarity affected the molar absorption coefficient, which is the highest in toluene and the lowest in both acetonitrile and methanol. The ratio between the bands with different solvents remains constant.

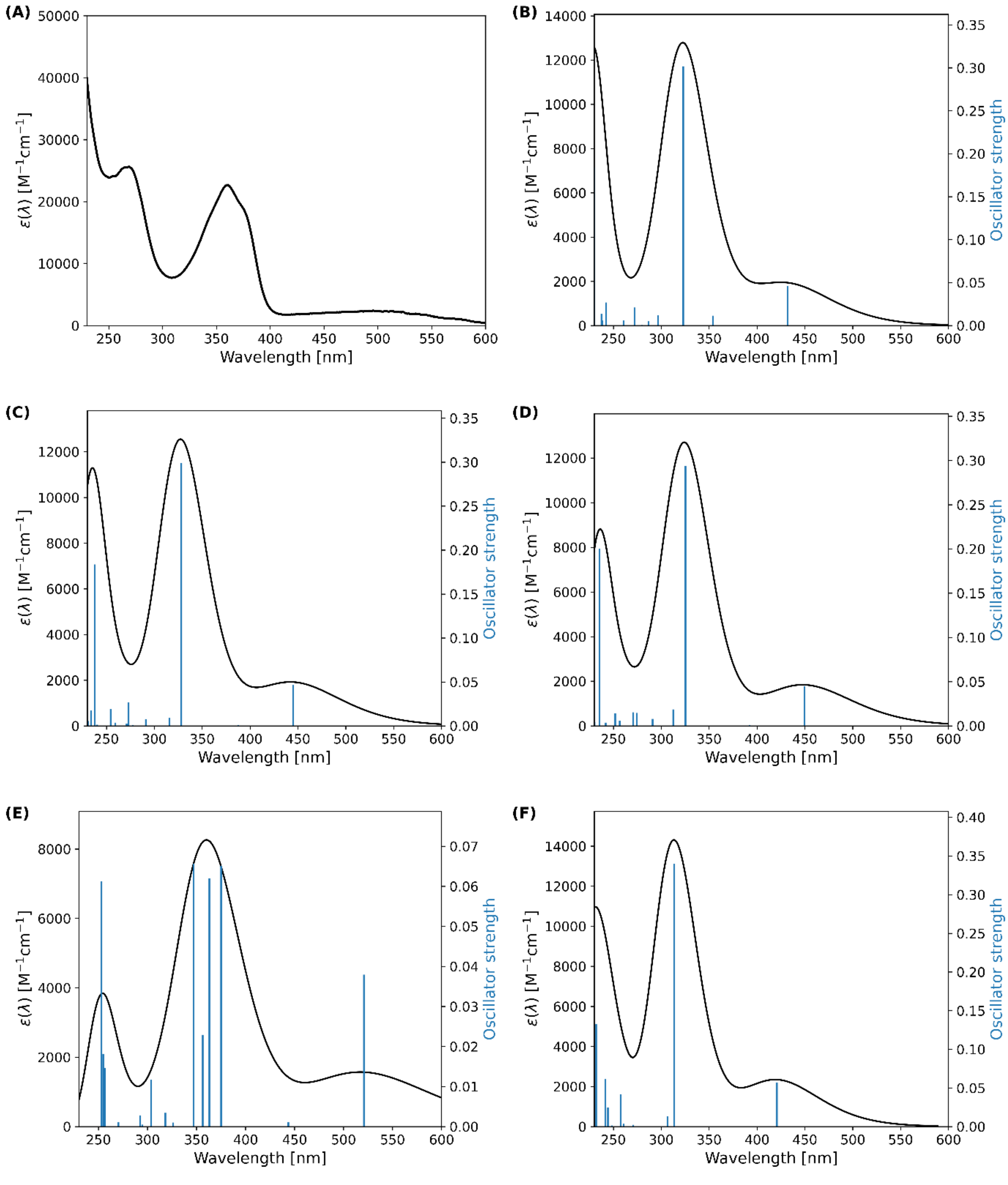


**Figure S28. (A)** Experimentally obtained UV-Vis spectrum of **PI** in acetonitrile at 10 °C. Simulated UV-Vis spectra calculated in acetonitrile with the basis set ma-def2-TZVPP and the functional **(B)** BHHLYP, **(C)** CAM-B3LYP, **(D)** M06-2X, **(E)** PBE0, **(F)** ωB97X-D3. The geometries were optimized at the $r^2$SCAN-3c level. The simulated UV-Vis spectra include the oscillator strength of the calculated roots (blue) and the Gaussian expansion of the states (black), which was converted into the molar absorption coefficient $\varepsilon$.

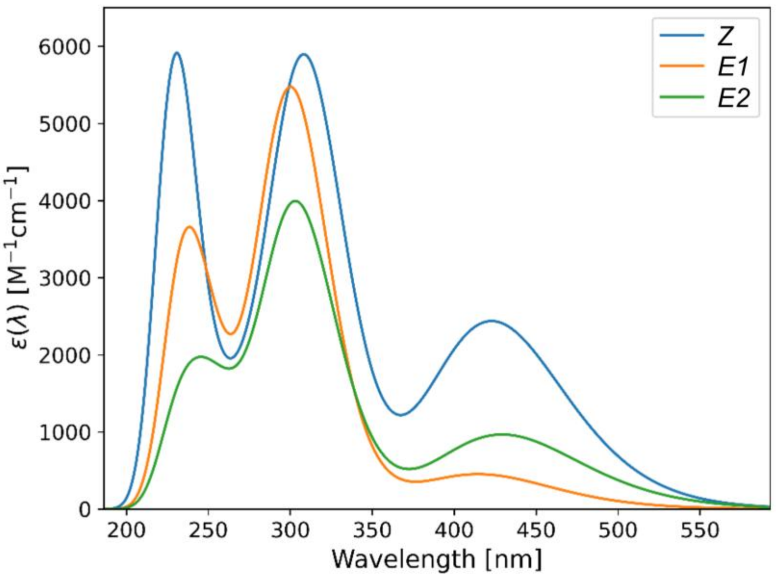


**Figure S29.** Simulated UV-Vis spectra (M06-2X/def2-TZVPP//r$^2$SCAN-3c) of **PI** in the gas phase for ***Z***, ***E1*** and ***E2***.

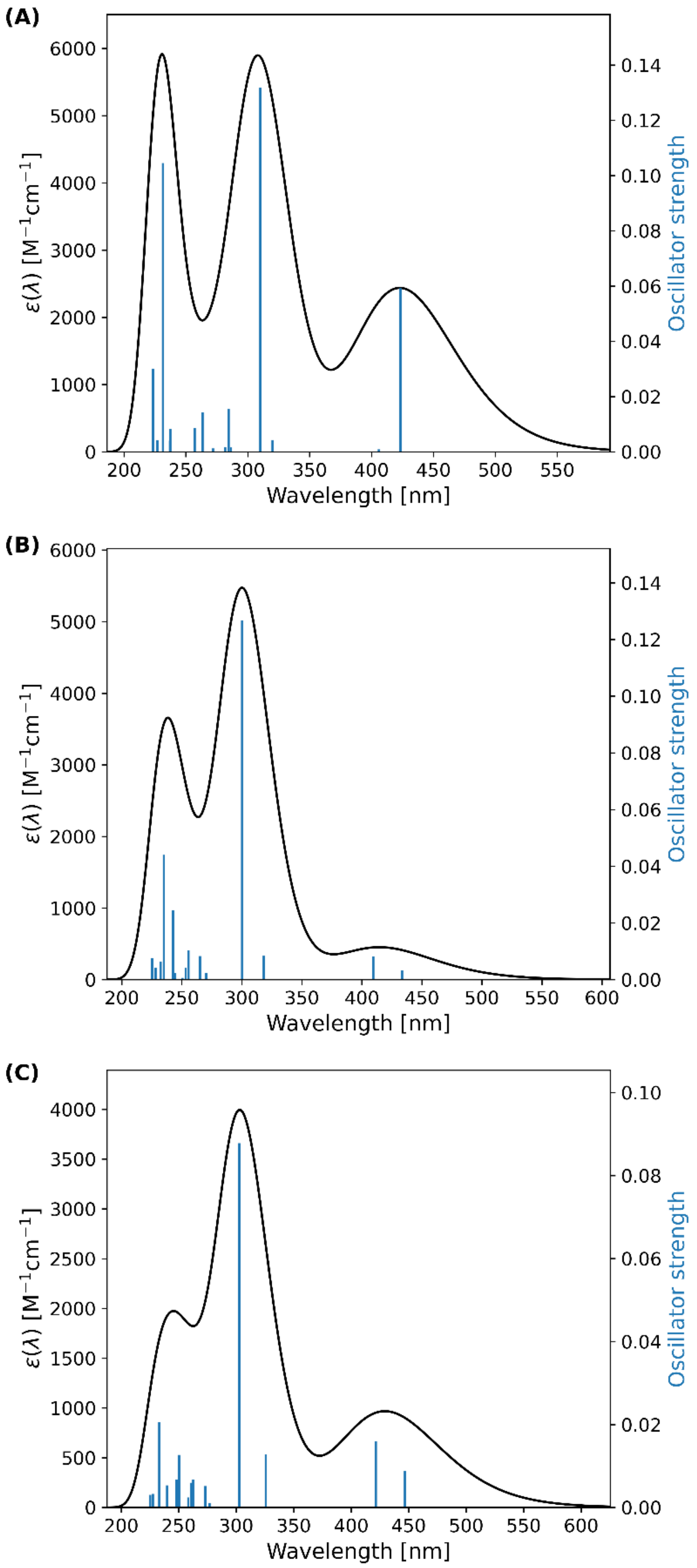


**Figure S30.** Simulated UV-Vis absorption spectra (M06-2X/def2-TZVPP//r$^2$SCAN-3c) of **PI** in the gas phase. (A) ***Z***; (B) ***E1***; (C) ***E2***.

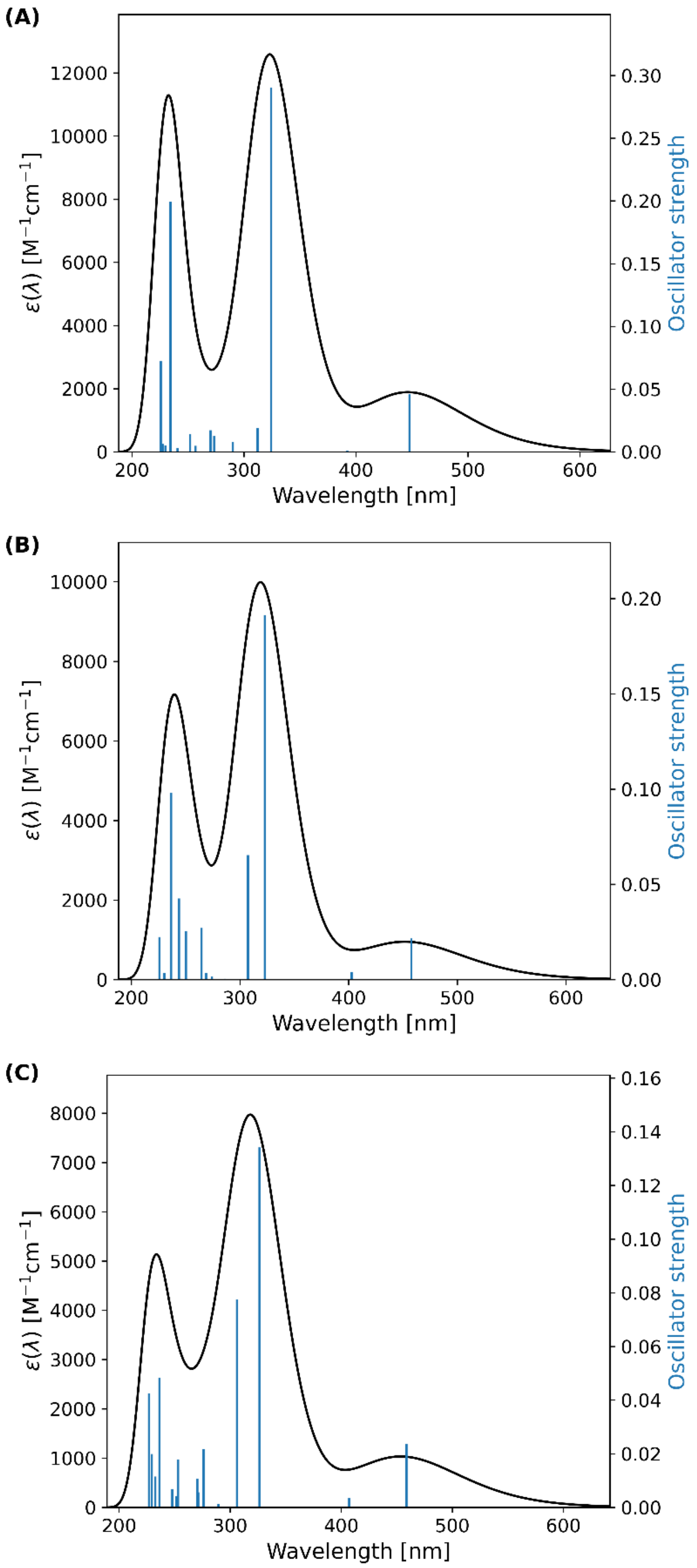


**Figure S31.** Simulated UV-Vis absorption spectra (M06-2X/def2-TZVPP/CPCM($CH_3CN$)//r$^2$SCAN-3c/CPCM($CH_3CN$)) of **PI** in acetonitrile. (A) ***Z***; (B) ***E1***; (C) ***E2***.

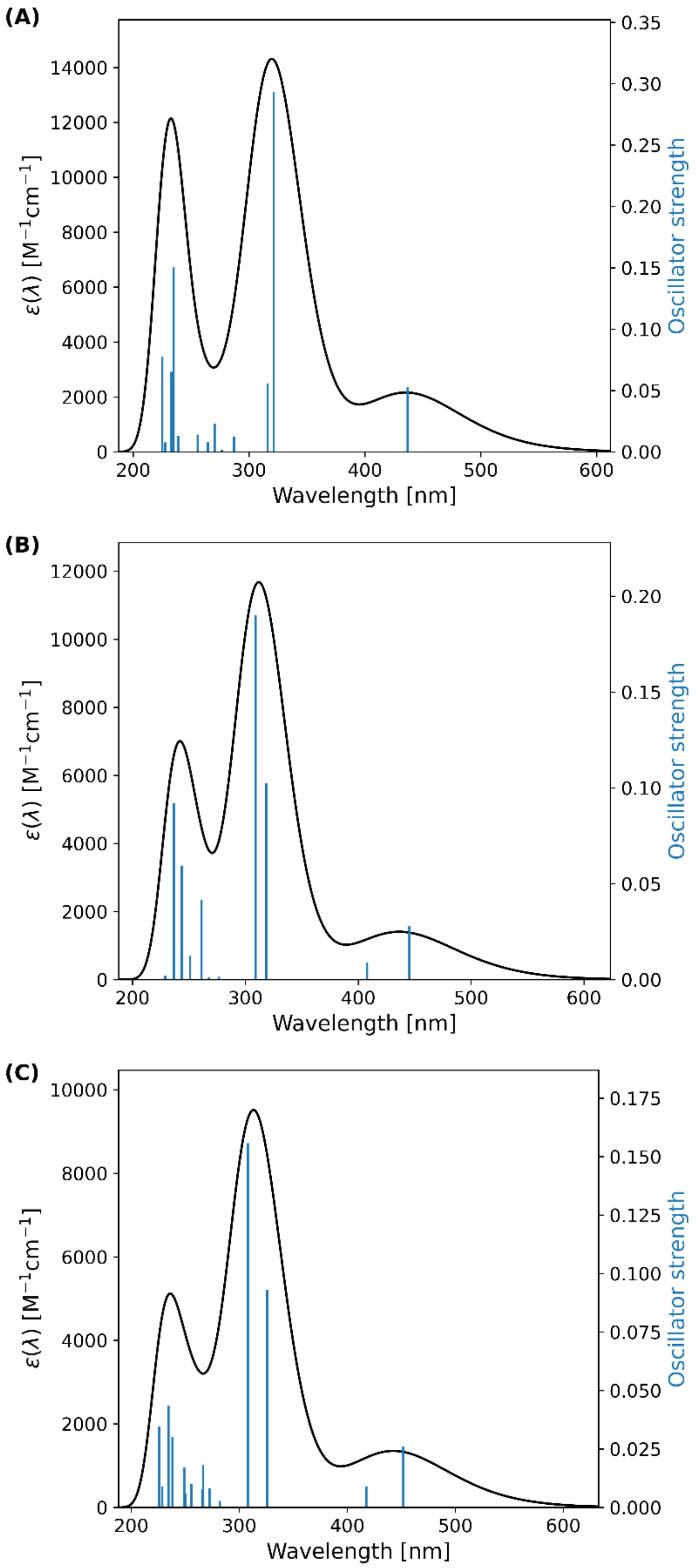


**Figure S32.** Simulated UV-Vis absorption spectra (M06-2X/def2-TZVPP/CPCM(cyclohexane)//r$^2$SCAN-3c/CPCM(cyclohexane)) of **PI** in cyclohexane. (A) ***Z***; (B) ***E1***; (C) ***E2***.

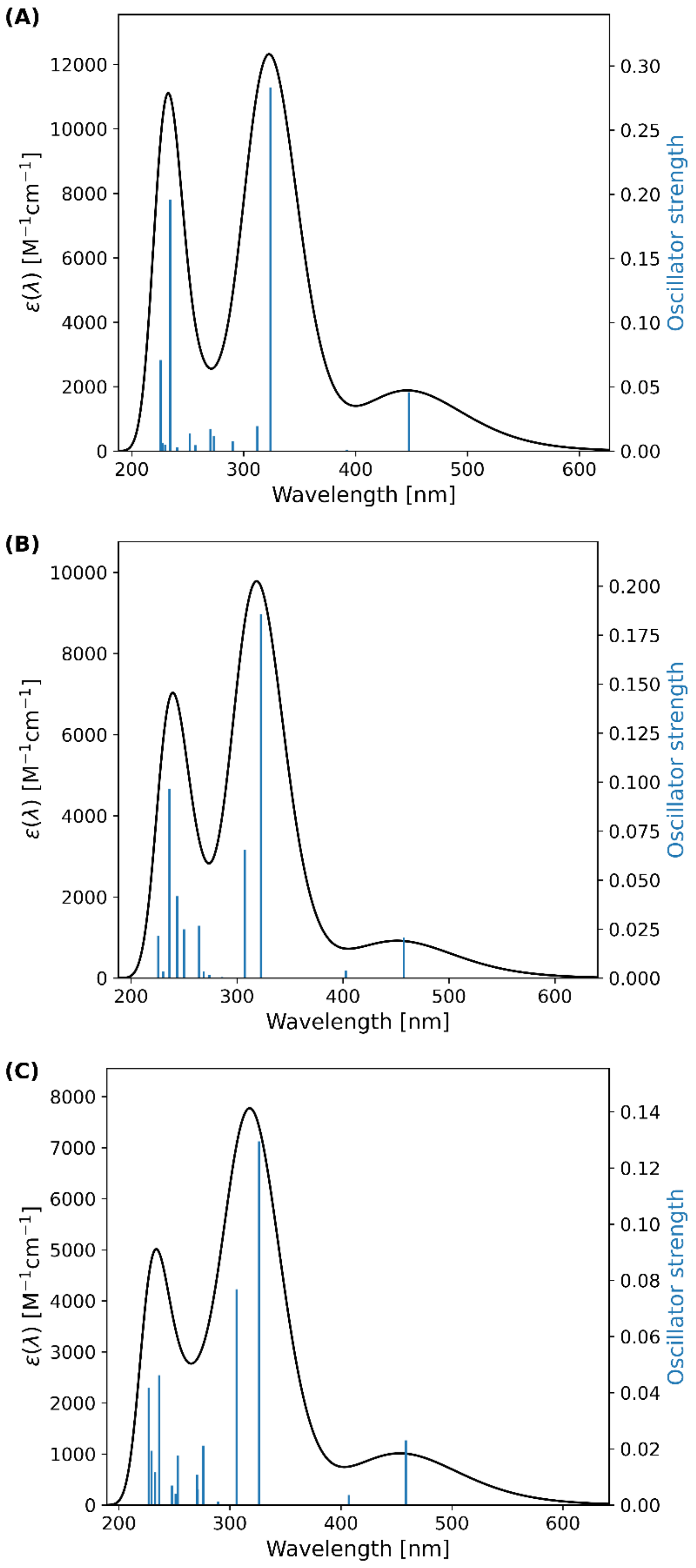


**Figure S33.** Simulated UV-Vis absorption spectra (M06-2X/def2-TZVPP/CPCM($CH_3OH$)//r$^2$SCAN-3c/CPCM($CH_3OH$)) of **PI** in methanol. (A) ***Z***; (B) ***E1***; (C) ***E2***.

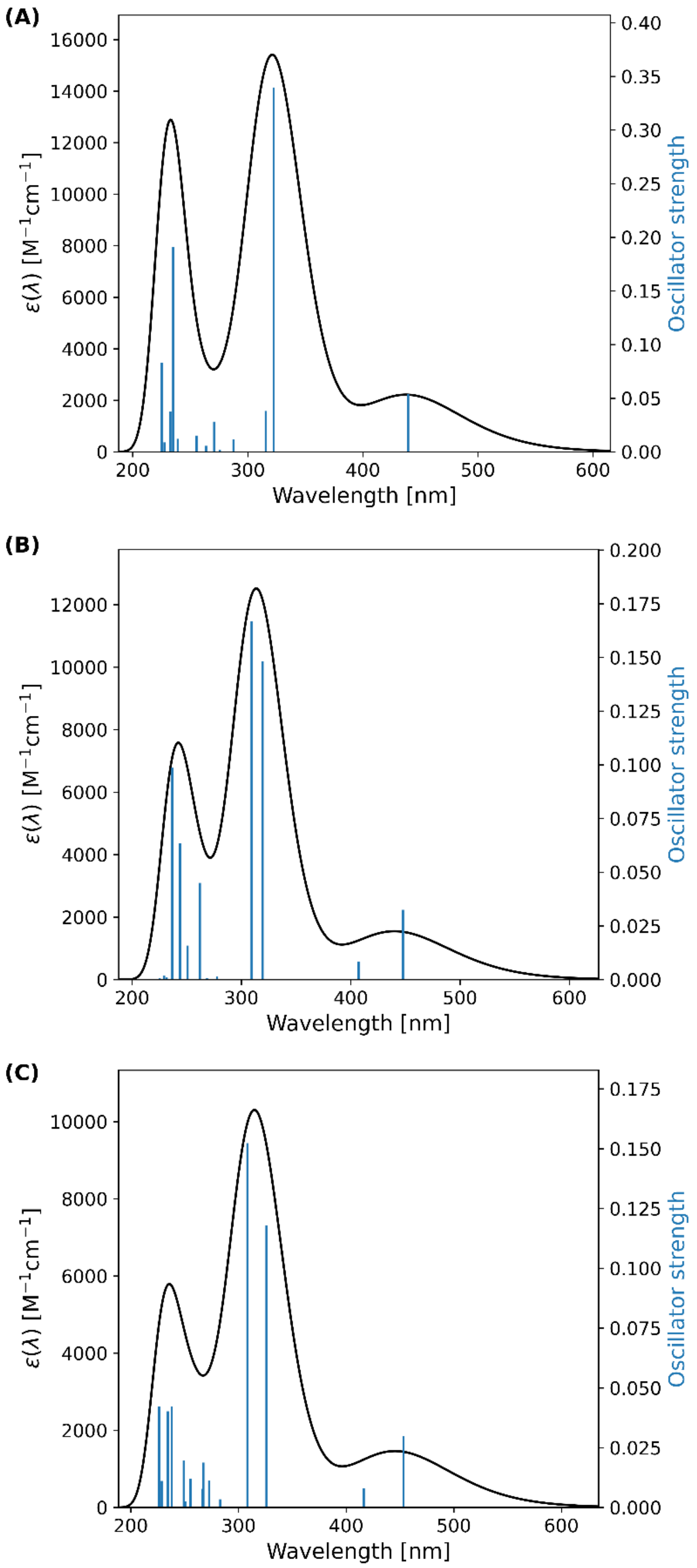


**Figure S34.** Simulated UV-Vis absorption spectra (M06-2X/def2-TZVPP/CPCM($PhCH_3$)//$r^2$SCAN-3c/CPCM($PhCH_3$)) of **PI** in toluene. (A) ***Z***; (B) ***E1***; (C) ***E2***.

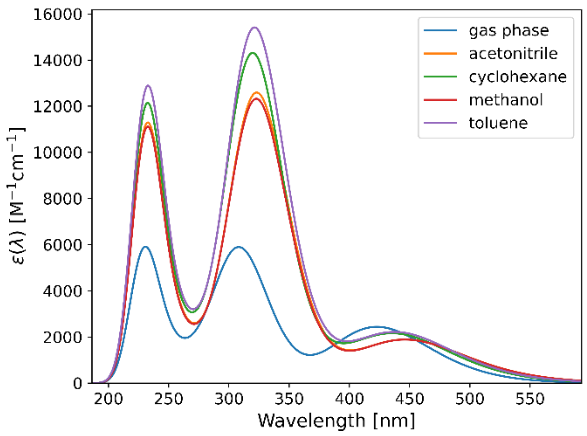


**Figure S35.** Simulated UV-Vis spectra (M06-2X/def2-TZVPP//r$^2$SCAN-3c) of ***Z*-PI** in various solvents.

### 8.5.1 Natural Transition Orbitals

The electronic transitions of the first, third, and fourth roots of **PI** are of particular interest (see **Figure S29**). To determine the type of transition that occurs, the highest occupied and lowest unoccupied natural transition orbitals (NTO) of each root were obtained (**Figure S36**). The NTO for the first root showed a $\pi \rightarrow \pi^*$ transition. The type of orbitals found in both **a** and **b** indicates $\pi$-character, as all of the orbitals were located above and beneath the bonds. The majority of the orbitals were located on the oxindole unit. The occupied NTO for root three **c** showed a different type. These orbitals were mostly located between the atoms, indicating the existence of an $n$-type orbital and, therefore, an $n \rightarrow \pi^*$ transition. The most intense signal in the simulated UV-Vis spectra was obtained for the fourth root. The transition of this root showed again a $\pi \rightarrow \pi^*$ transition, with involvement of the double bond. The types of transition were confirmed by an MRSF-TDDFT calculation of the same molecule. This higher-level calculation provides a more accurate representation of the excited states. The orbitals obtained from MRSF-TDDFT were canonical molecular orbitals (CMO) (see **Figure S36**). However, it is still possible to determine the type of transition by comparing the location of the orbitals. Each different CMO has a varying degree of participation in the transition. The first root of the MRSF-TDDFT calculation with 0.1269 oscillator strength is characterized mainly by the orbital 100 **l** → 101 **m**, revealing the expected $\pi \rightarrow \pi^*$ transition. The second root, characterized by a lower oscillator strength (0.0100), sees orbitals 94 **g** and 97 **j** donating to 101, supporting the $n \rightarrow \pi^*$ transition classification. The remaining orbitals 95, 96 and 99 donating to 101 were assigned for the third root with an oscillator strength of 0.6263. These orbitals also exhibited a $\pi \rightarrow \pi^*$ transition.

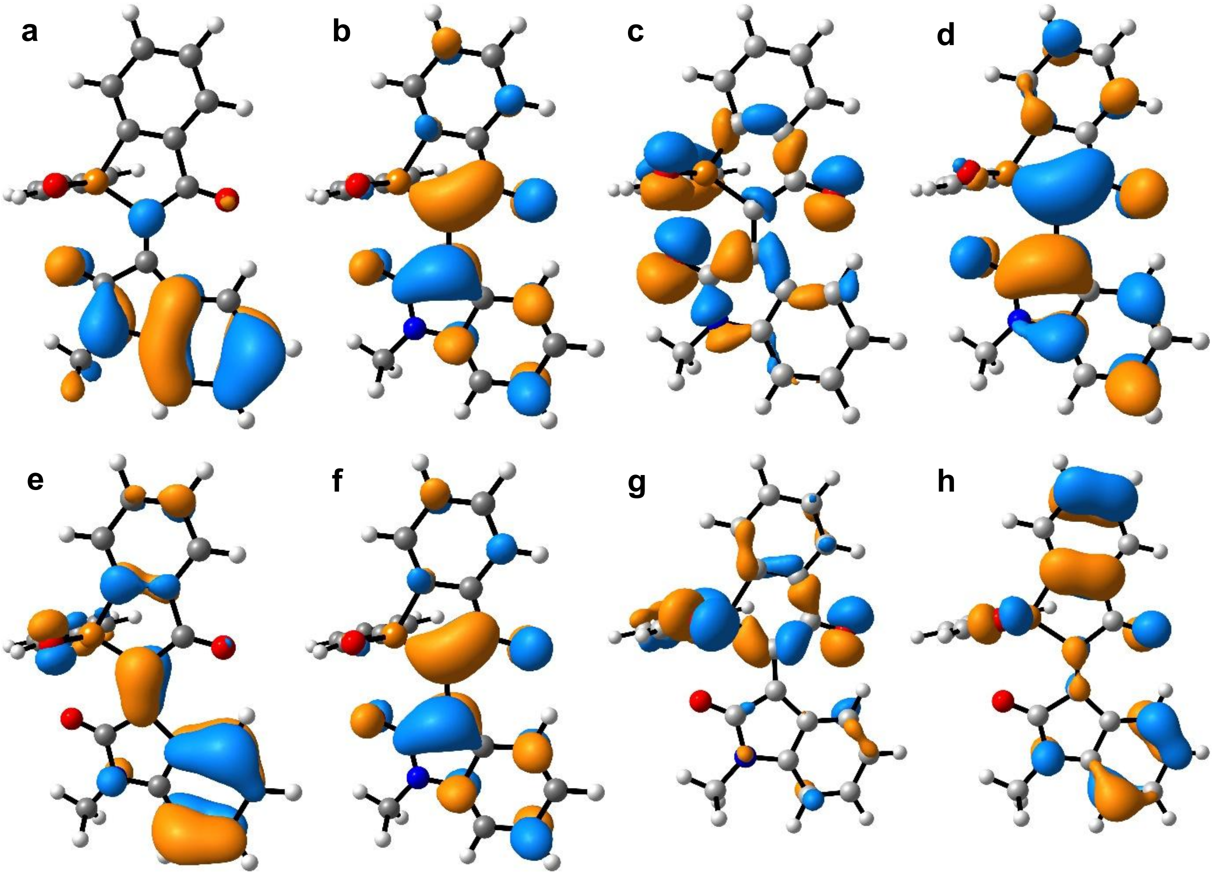

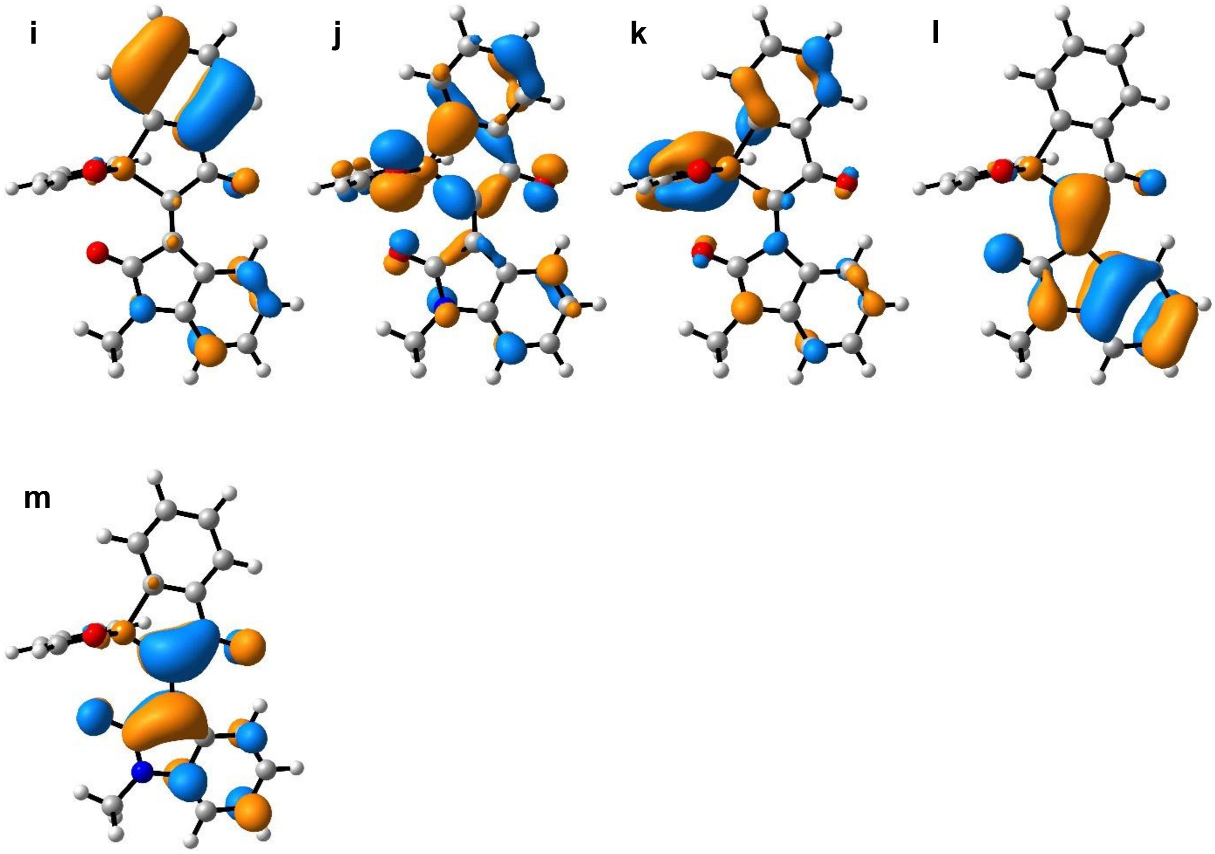


**Figure S36.** Natural transition orbitals (NTO) for **PI-*Z*** in gas phase (M06-2X/def2-TZVPP//r$^2$SCAN-3c) obtained from TDDFT calculations. Root 1: **a** occupied orbital and **b** unoccupied orbital. Root 3: **c** occupied orbital and **d** unoccupied orbital. Root 4: **e** occupied orbital and **f** unoccupied orbital. Occupied canonical molecular orbitals (CMO) for **PI-*Z*** in gas phase (MRSF-BHHLYP/def2-TZVP//MRSF-BHHLYP/6-31G(d)) obtained from MRSF-TDDFT calculation: **g** Orbital 94, **h** Orbital 95, **i** Orbital 96, **j** Orbital 97, **k** Orbital 99, **l** Orbital 100, **m** Orbital 101.

### 8.5.2 Protonated Phosphaindirubin

The simulated UV-Vis spectra of the *Z*- and *E*-isomers of **PI** protonated at two different sites (i.e. **POH** and **COH**) are depicted in **Figure S37**.

The spectra of both **POH-*E*** conformers show a slightly red-shifted visible band ($\lambda_{max}$ 500 nm) compared to **POH-*Z***. The spectrum of **COH-*Z*** shows severely red-shifted UV and visible bands compared to **POH-*Z*** and **COH-*E***.

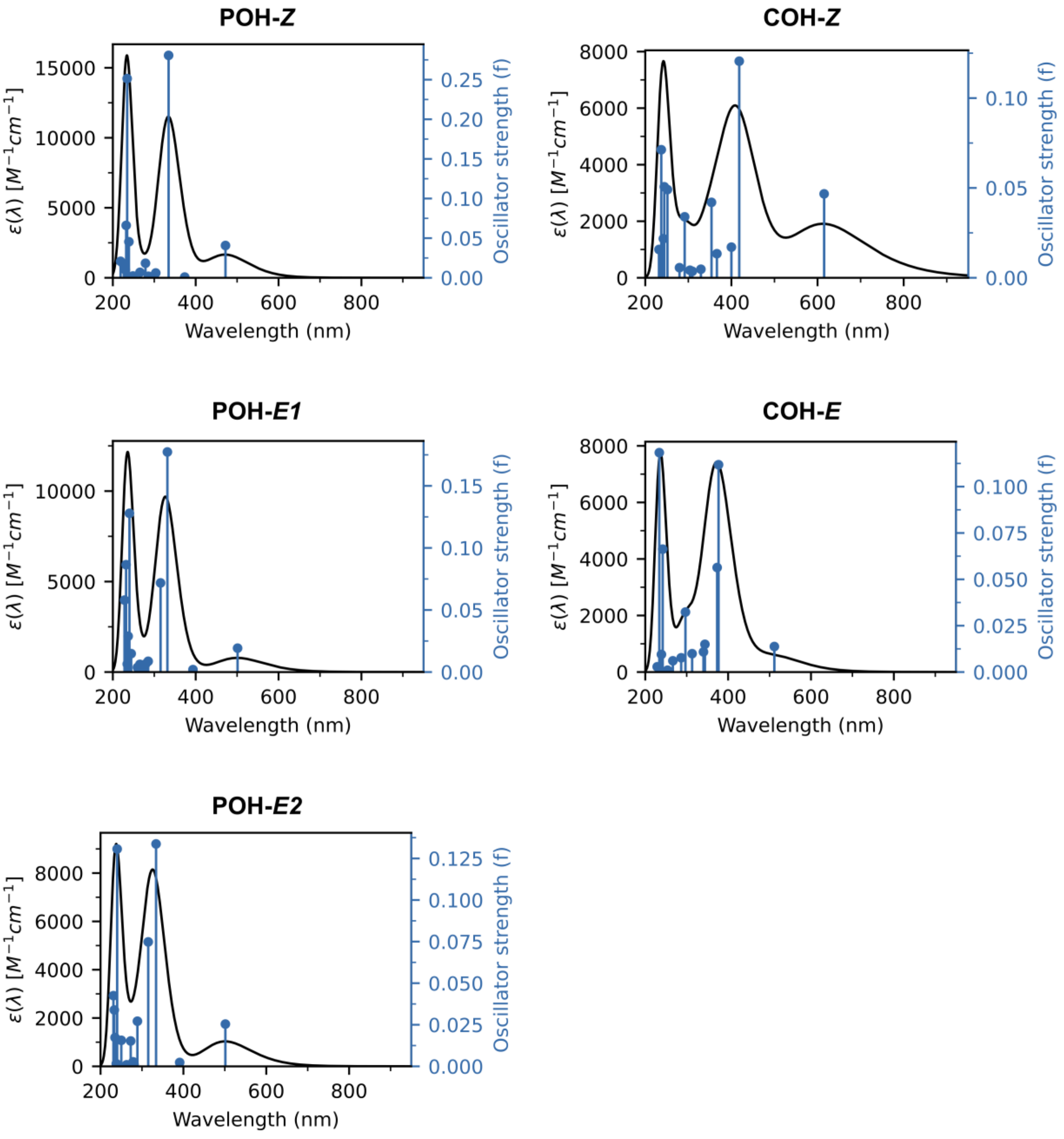


**Figure S37.** Simulated TDDFT spectra of **POH** and **COH** at two different sites obtained at the M06-2X/def2-TZVPP/CPCM($CH_3CN$)//r$^2$SCAN-3c/CPCM($CH_3CN$) level with 15 roots.

## 8.6 $^{31}$P NMR Calculations

### 8.6.1 Without Water

The calculated $^{31}$P NMR chemical shifts for the stable and metastable isomers of **PI** protonated at either the P=O (**POH**) or the C=O of the benzophospholane unit (**COH**) are depicted in **Figure S38**. With **PI-*Z*** as the reference, the ***Z***, ***E1***, and ***E2*** isomers of **PI-POH** are significantly shifted (+35.0, +38.7, and +37.7 ppm, respectively), while the corresponding isomers of **PI-COH** are only shifted by +8.5 and +4.3 ppm, respectively. The ***E1*** and ***E2*** of **PI** are found at -2.2 and +3.8 ppm with respect to **PI-*Z***.

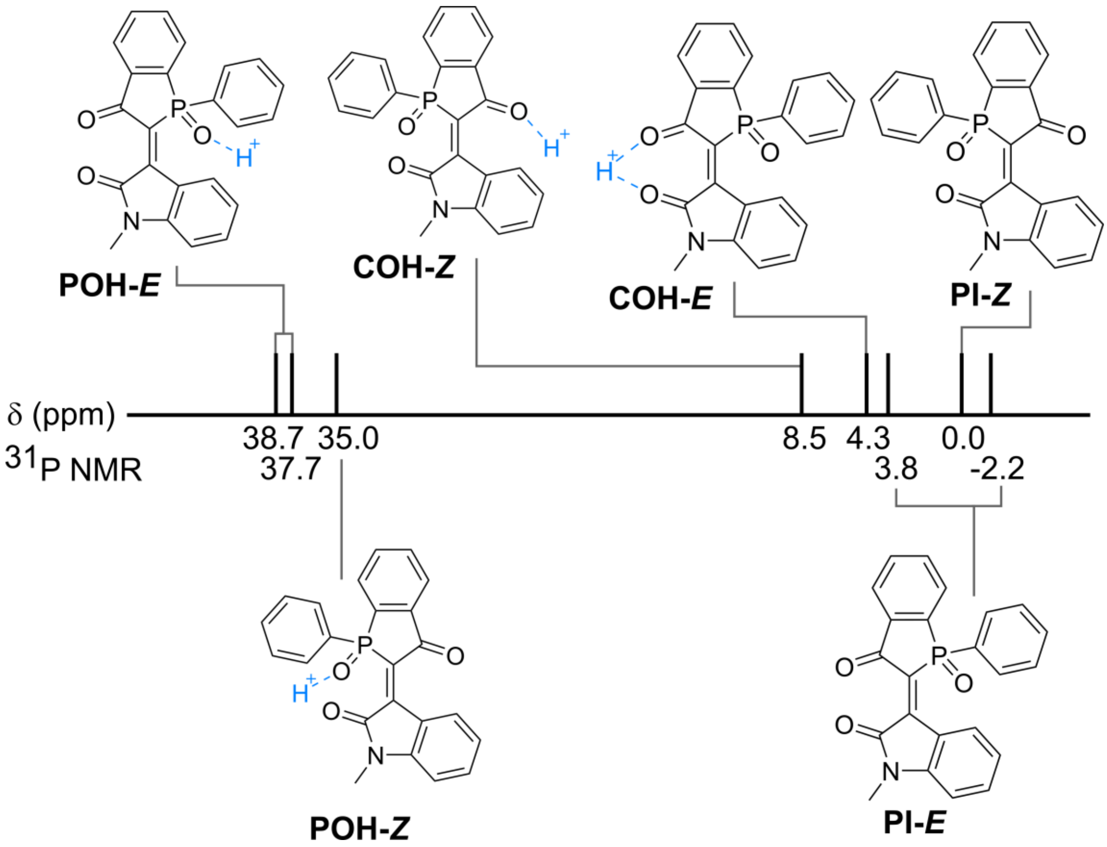


**Figure S38.** Calculated $^{31}$P NMR shifts for the *Z*- and *E*-isomers of **PI** protonated at either the P=O (**POH**) or the C=O of the benzophospholane unit (**COH**) at the ωB97X-V/def2-QZVP/CPCM($CH_3CN$)//r$^2$SCAN-3c/CPCM($CH_3CN$) level. **PI-*Z*** is picked as the reference. See **Figure S25** for depictions of the optimised geometries.

### 8.6.2 With 3 Water Molecules

With the addition of three water molecules, the $^{31}$P chemical shifts of the **POH** isomers have shifted upfield compared to those without water molecules (**Figure S39**).

During optimisation of the **COH-*Z*** isomer with three molecules of water, the protons migrated, resulting in the formation of a PO-H hydrogen bond (1.42 Å) – thus, we labelled it **COH-*Z****. While the energies of **POH-*Z*•3H$_2$O** and **COH-*Z**•3H$_2$O** are the same (see **Figure S26**), the $^{31}$P NMR shifts are different (+11.1 ppm *vs* +2.5 ppm), most likely due to the PO-H hydrogen bond distances of 1.13 Å and 1.42 Å, respectively – a weaker hydrogen bond for **COH-*Z**•3H2O** relates to a smaller chemical shift.

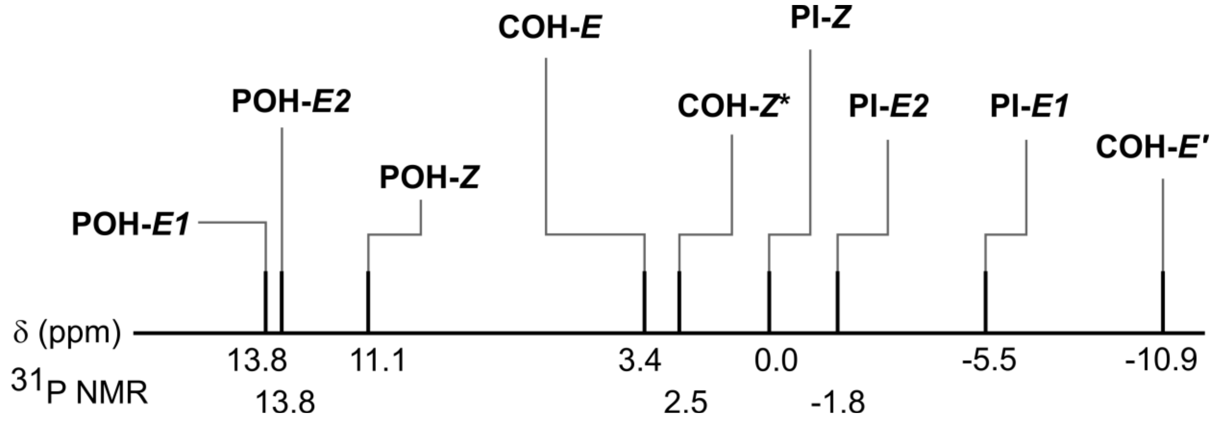


**Figure S39.** Calculated $^{31}$P NMR shifts for the *Z*- and *E*- E-isomers of **PI** protonated at either the P=O (**POH**) or the C=O of the benzophospholane unit (**COH**) with 3 water molecules at the ωB97X-V/def2-QZVP/CPCM(CH$_3$CN)//r$^2$SCAN-3c/CPCM(CH$_3$CN) level. **PI-*Z*** is picked as the reference. **COH-*Z****: optimization of the PI-COH *Z*-isomer resulted in **POH-*Z*** with PO-H bond of 1.42 Å; **COH-*E*´**: *E*-isomer of **COH** adopts a flat geometry due to a bridging hydrogen bond. See **Figure S26** for depictions of the optimised geometries.

## 8.7. Simulated IR spectra

Gaussian broadening was applied to the vertical transitions with a linewidth of 15 $cm^{-1}$ (FWHM).

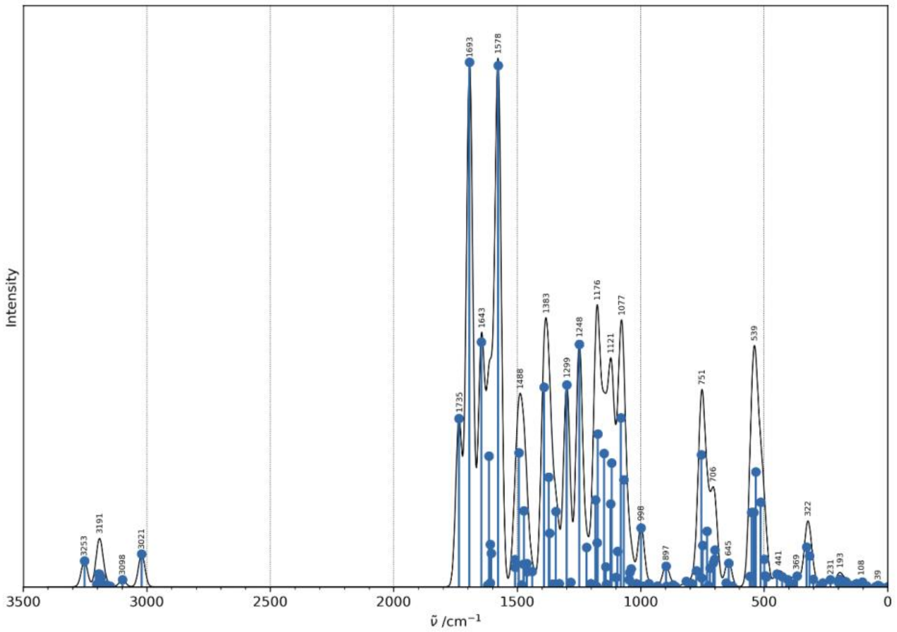


**Figure S40.** Vertical transitions and simulated IR spectra of **PI-*Z*** obtained from geometries optimized at the $r^2$SCAN-3c/CPCM($CH_3CN$) level.

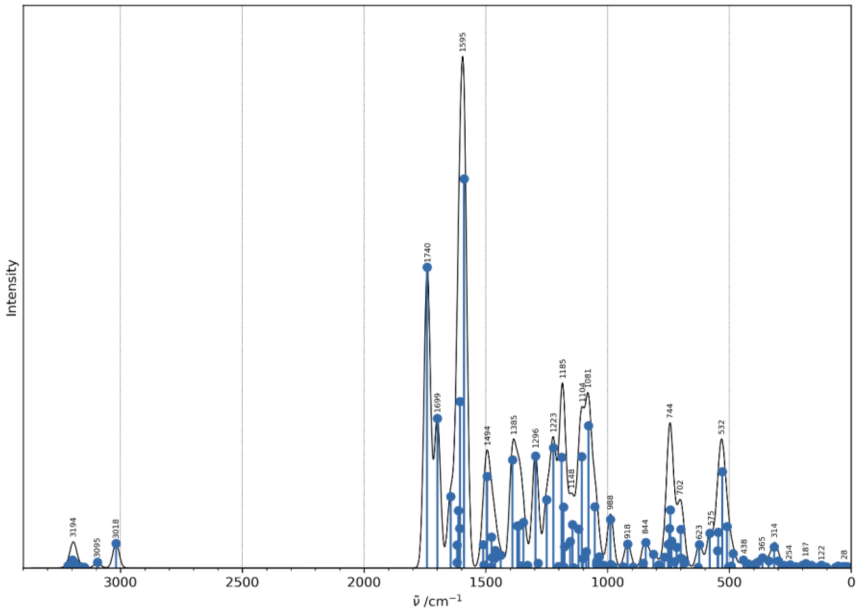


**Figure S41.** Vertical transitions and simulated IR spectra of **PI-*E1*** obtained from geometries optimized at the $r^2$SCAN-3c/CPCM($CH_3CN$) level.

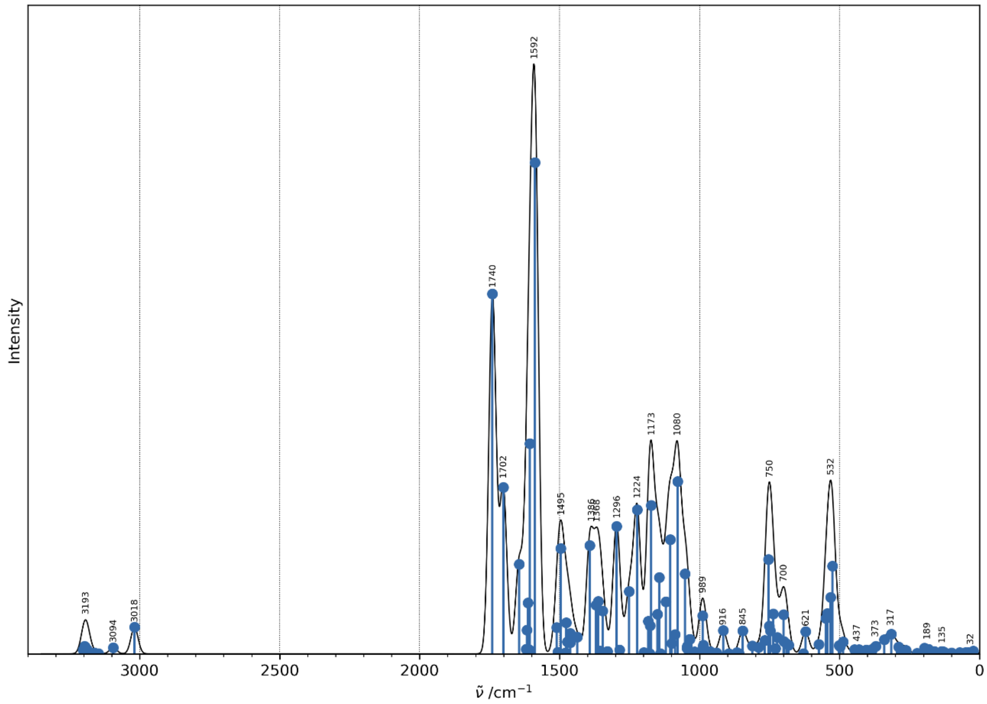


**Figure S42.** Vertical transitions and simulated IR spectra of **PI-*E2*** obtained from geometries optimized at the r$^2$SCAN-3c/CPCM($CH_3CN$) level.

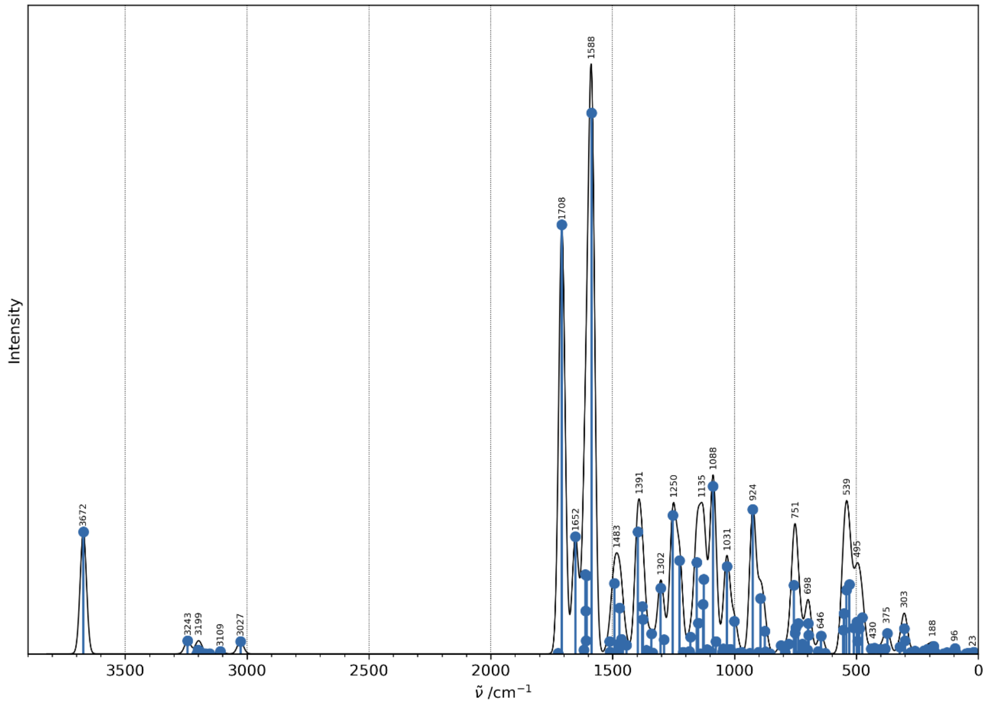


**Figure S43.** Vertical transitions and simulated IR spectra of **POH-*Z*** obtained from geometries optimized at the r$^2$SCAN-3c/CPCM($CH_3CN$) level.

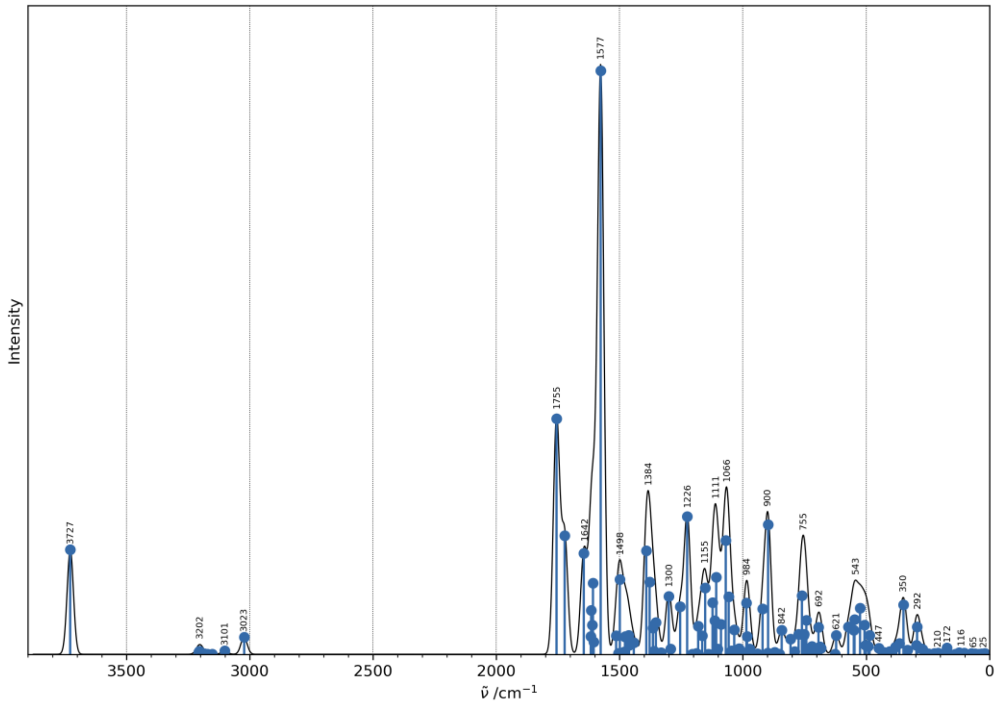


**Figure S44.** The vertical transitions and simulated IR spectra of **POH-*E1*** were obtained from geometries optimized at the r$^2$SCAN-3c/CPCM($CH_3CN$) level.

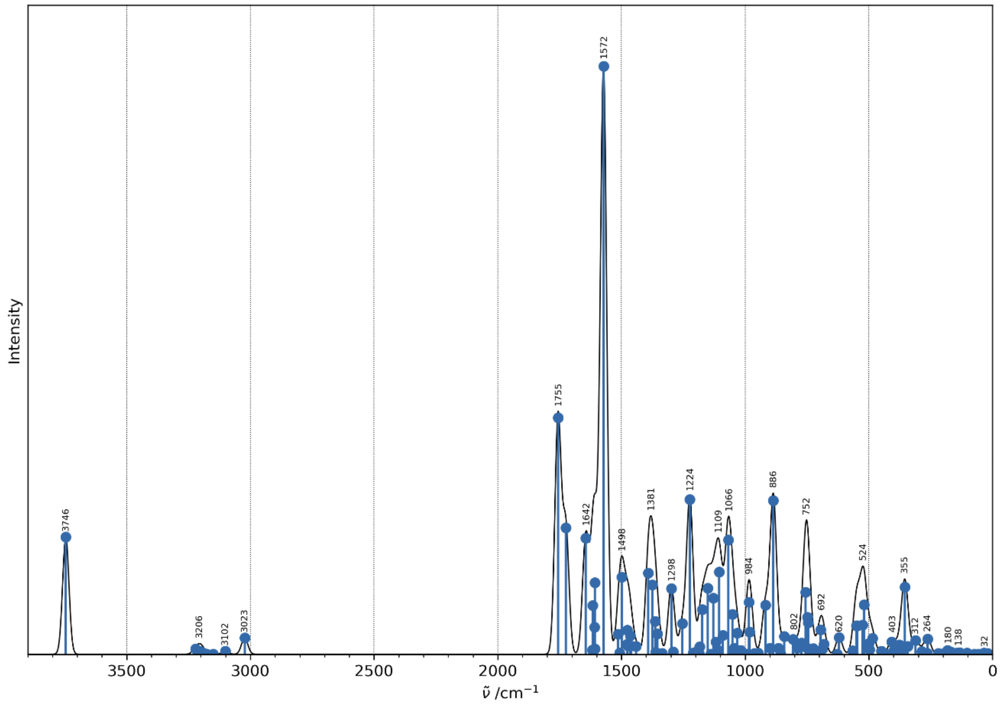


**Figure S45.** Vertical transitions and simulated IR spectra of **POH-*E2*** obtained from geometries optimized at the r$^2$SCAN-3c/CPCM($CH_3CN$) level.

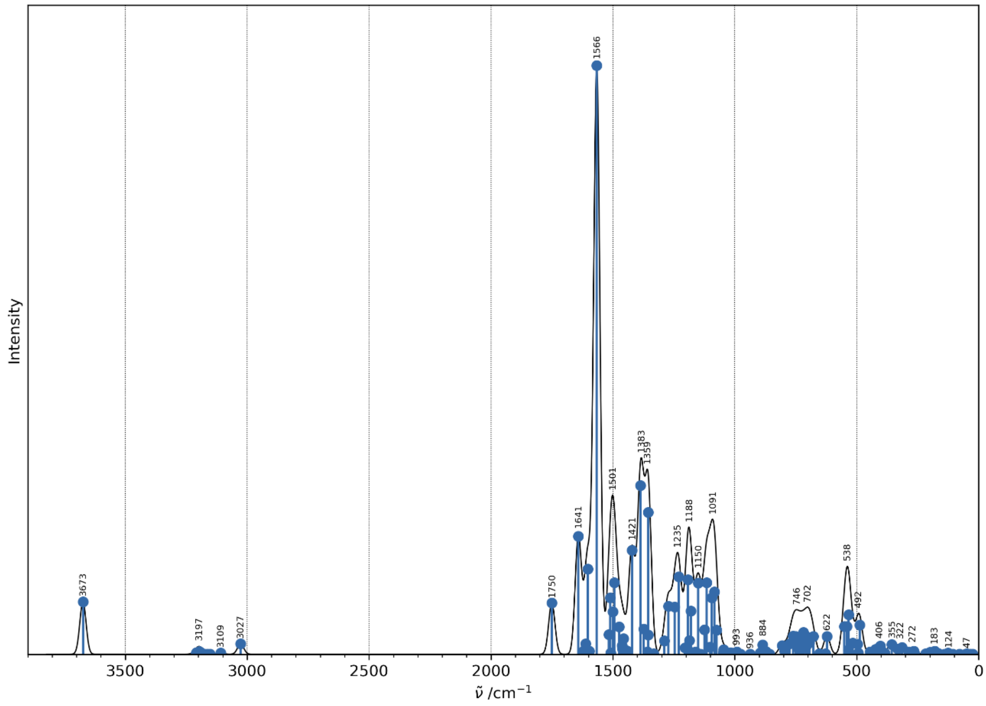


**Figure S46.** The vertical transitions and simulated IR spectra of **COH-*Z*** were obtained from geometries optimized at the r$^2$SCAN-3c/CPCM($CH_3CN$) level.

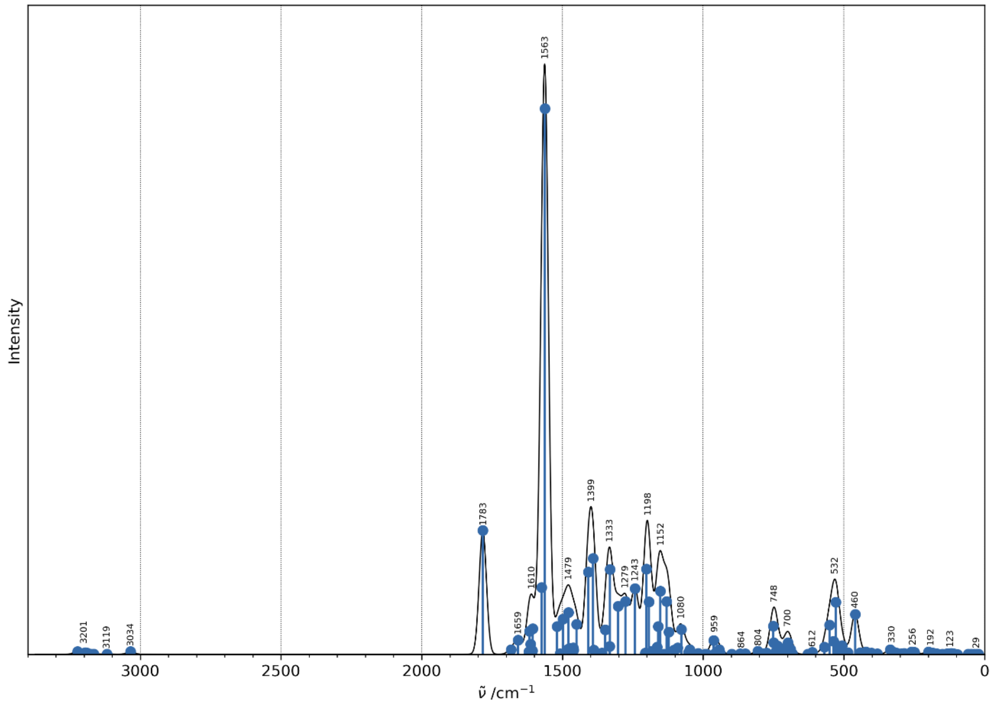


**Figure S47.** The vertical transitions and simulated IR spectra of **COH-*E*** were obtained from geometries optimized at the r$^2$SCAN-3c/CPCM($CH_3CN$) level.

# 9. UV-Vis Irradiation

## 9.1 Toluene

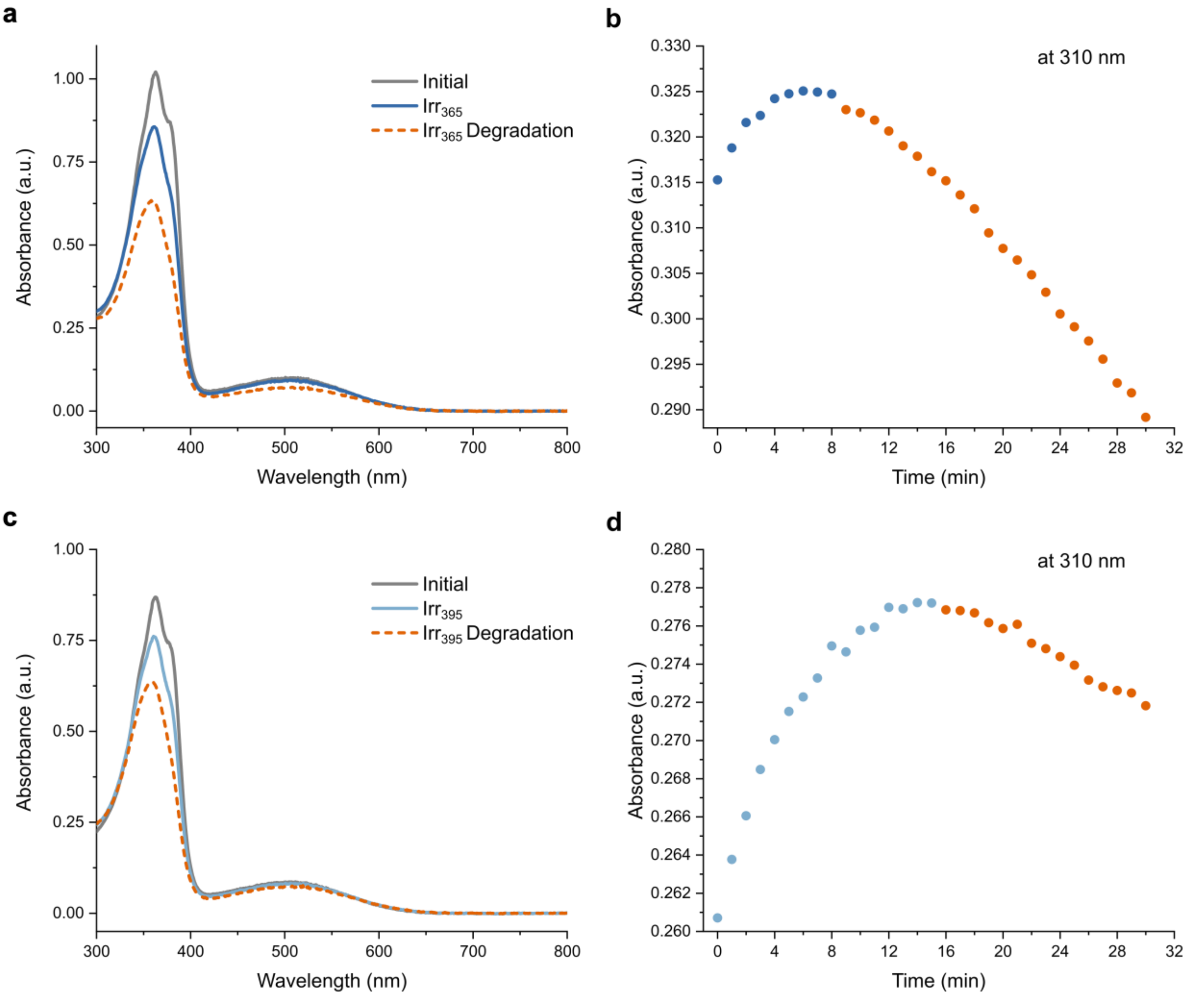


**Figure S48. a** Reached PSS (blue) and degradation (dashed orange) spectra after irradiation of **PI** in $PhCH_3$ ($5.0x10^{-2}$ mM) sparged with argon at λ = 365 nm. **b** The evolution of the wavelength at λλ = 310 nm at $Irr_{365}$. Photoisomerization (grey to blue) is observed over 8 minutes, reaching a PSS, followed by degradation (blue to dashed orange). **c** Reached PSS (light blue) and degradation (dashed orange) spectra after irradiation of **PI** in $PhCH_3$ ($4.3x10^{-2}$ mM) sparged with argon at λ = 395 nm. **b** The evolution of the wavelength at λ = 310 nm at $Irr_{395}$. Photoisomerization (grey to light blue) is observed over 15 minutes, reaching a PSS, followed by degradation (light blue to dashed orange).

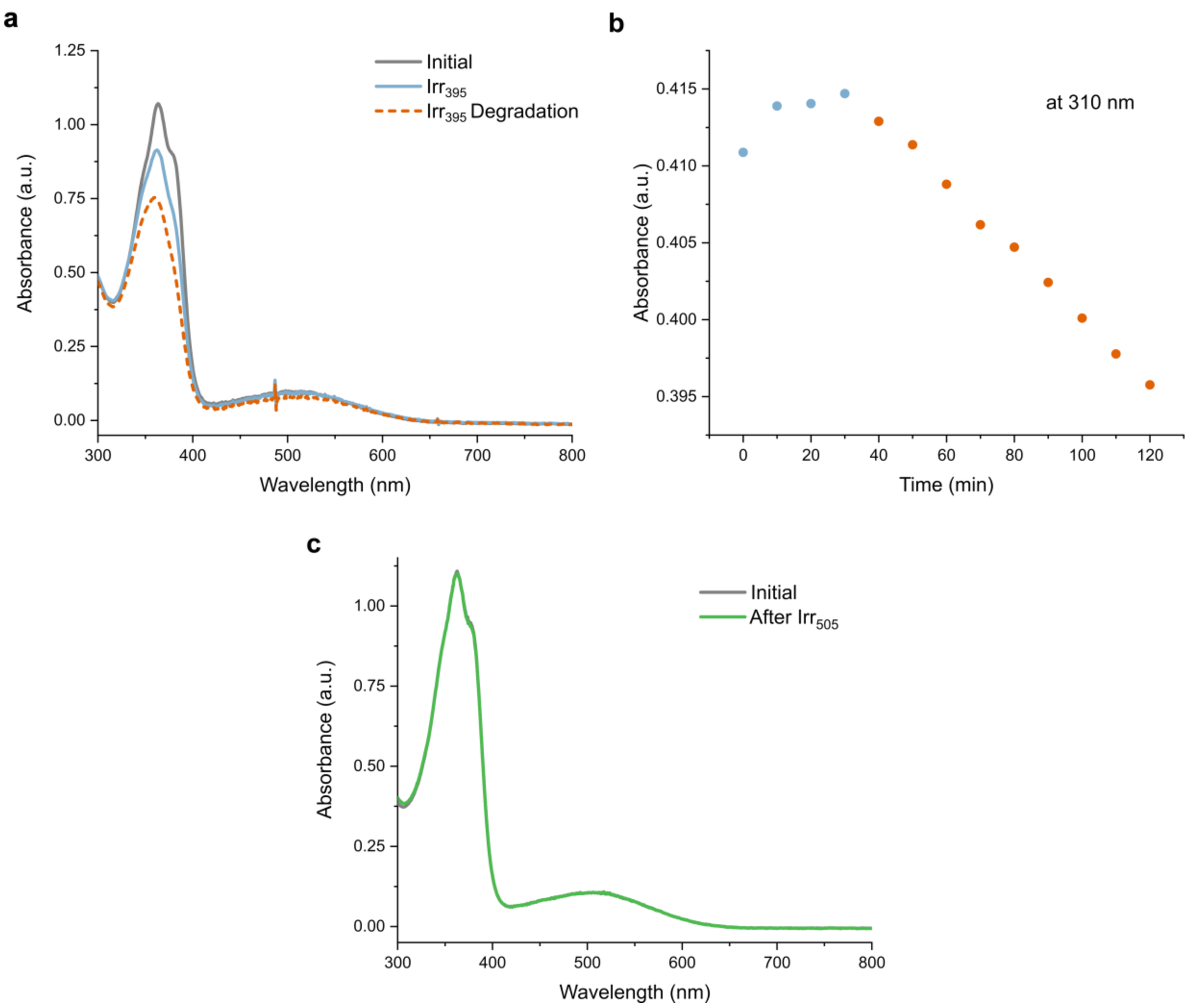


**Figure S49. a** Reached PSS (light blue) and degradation (dashed orange) spectra after irradiation of **PI** in $PhCH_3$ ($4.3x10^{-2}$ mM) sparged with argon at λ = 395 nm. **b** The evolution of the wavelength at λ = 310 nm at $Irr_{395}$. Photoisomerization (grey to light blue) is observed over 30 minutes, reaching a PSS, followed by degradation (light blue to dashed orange). A comparison of Figure **S48d** and Figure **S49b** reveals that control over argon is crucial. **c** Irradiation of **PI** in $PhCH_3$ ($5.5x10^{-2}$ mM) sparged with argon at λ = 505 nm. No switching is observed.

## 9.2 Tetrahydrofuran

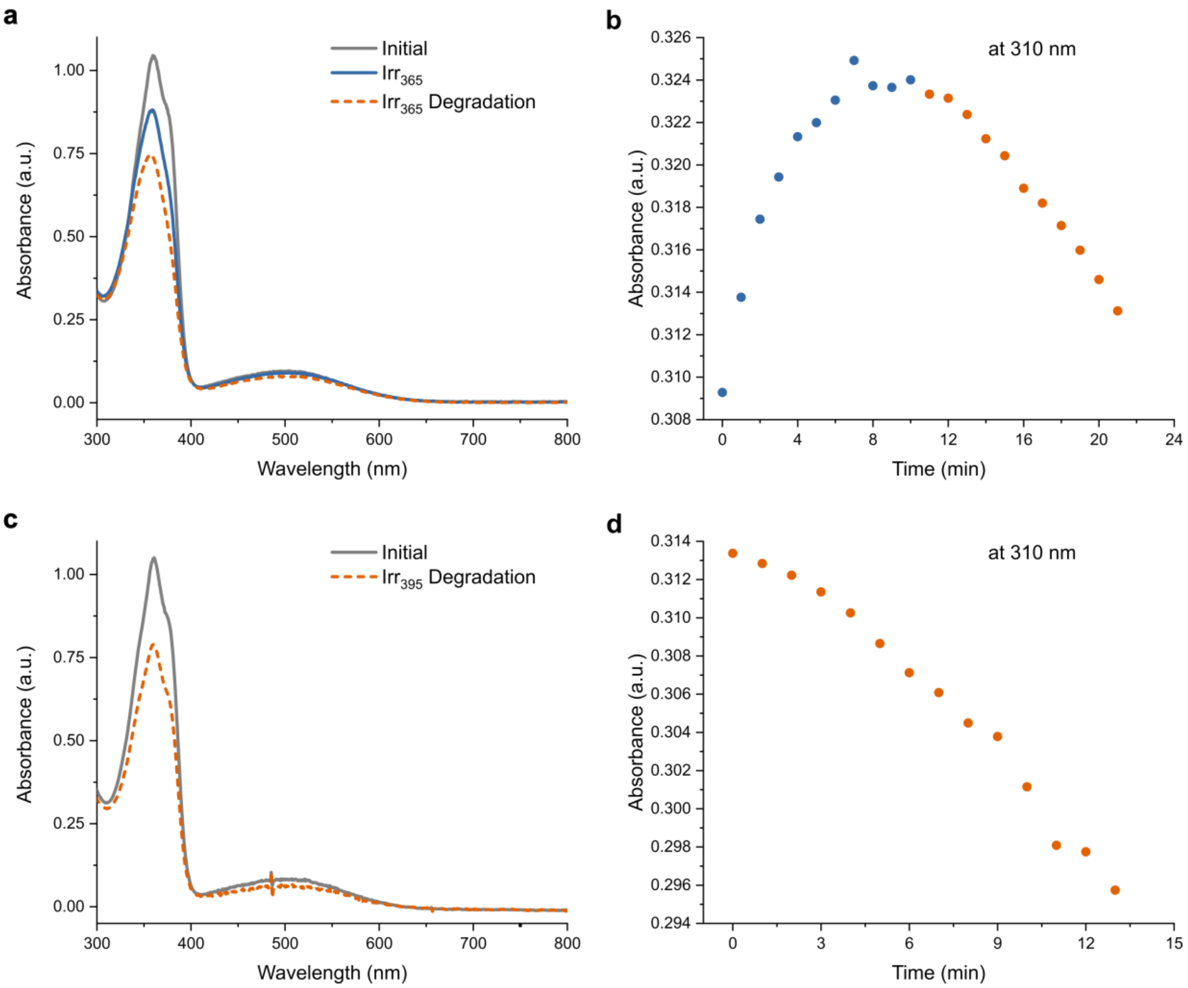


**Figure S50. a** Reached PSS (blue) and degradation (dashed orange) spectra after irradiation of **PI** in THF ($5.2 \times 10^{-2}$ mM) sparged with argon at $\lambda = 365$ nm. **b** Evolution of the wavelength at $\lambda = 310$ nm at $Irr_{365}$. Photoisomerization (grey to blue) is observed over 11 minutes, reaching a PSS, followed by degradation (blue to dashed orange). **c** Irradiation of **PI** in THF ($5.2 \times 10^{-2}$ mM) sparged with argon at $\lambda = 395$ nm. Degradation (grey to dashed orange) is immediately observed upon prolonged irradiation. **d** The evolution of the wavelength at $\lambda = 310$ nm at $Irr_{395}$.

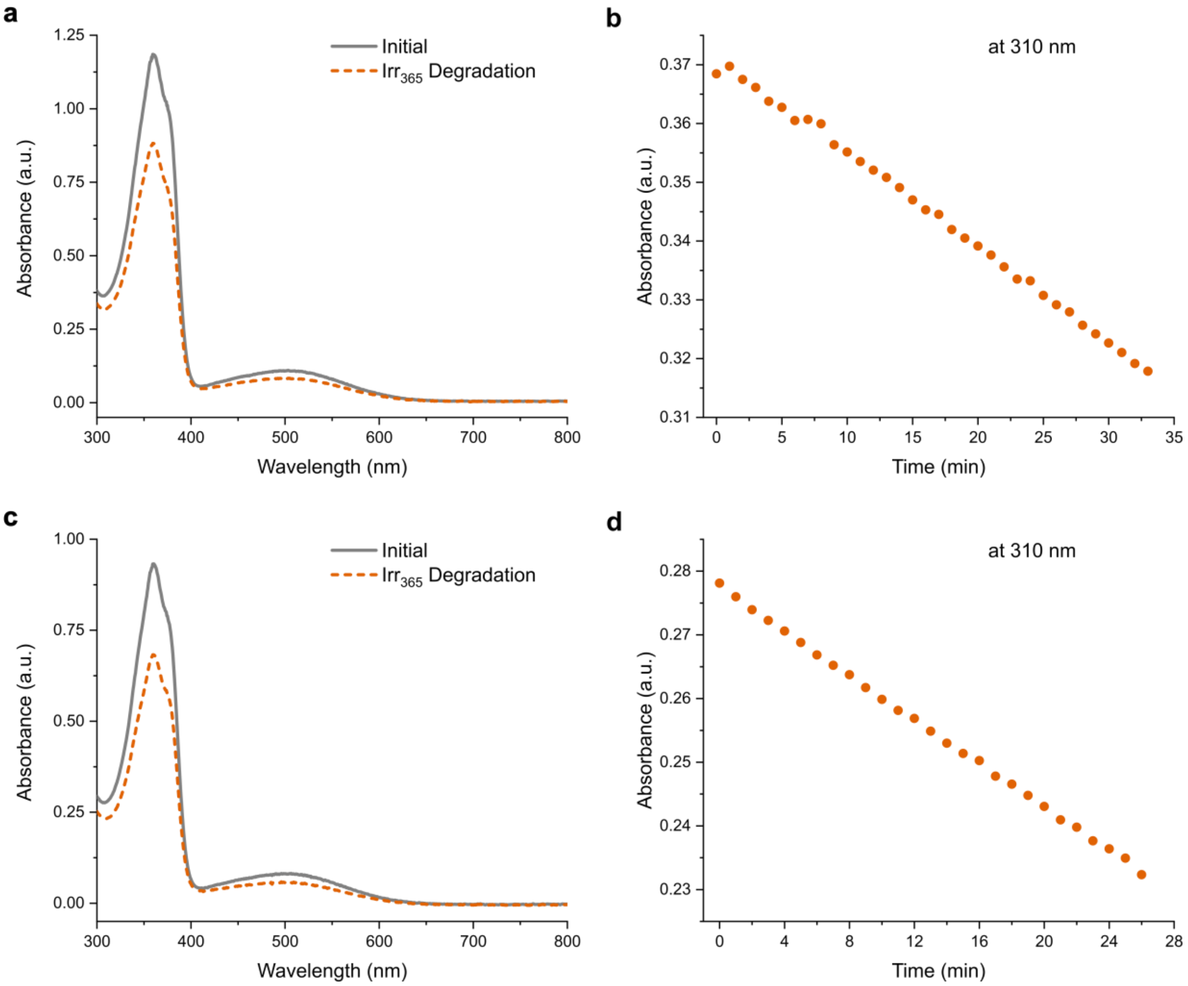


**Figure S51. a** Irradiation of **PI** in THF (5.9x10$^{-2}$ mM) with 10 µL $H_2O$ sparged with argon at $\lambda = 365$ nm. Degradation (grey to dashed orange) is immediately observed upon prolonged irradiation. **b** The evolution of the wavelength at $\lambda = 310$ nm at $Irr_{365}$. **c** Irradiation of **PI** in THF (4.7x10$^{-2}$ mM) with 10 µL $D_2O$ sparged with argon at $\lambda = 365$ nm. Degradation (grey to dashed orange) is immediately observed upon prolonged irradiation. **d** The evolution of the wavelength at $\lambda = 310$ nm at $Irr_{365}$.

## 9.3 Acetonitrile

### 9.3.1 Acid Presence in Solvent

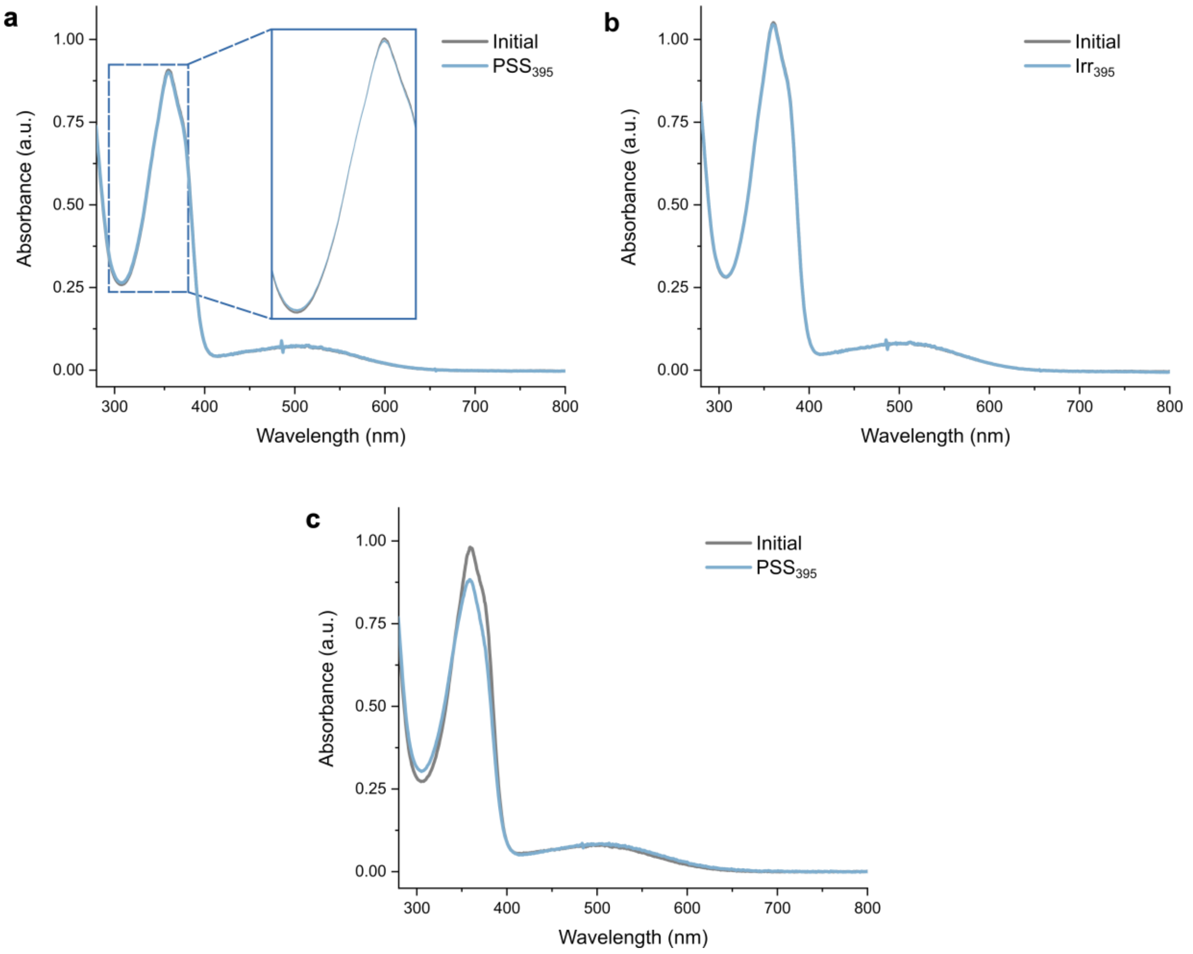


**Figure S52. a** Irradiation of **PI** ($3.9x10^{-2}$ mM) in prolonged opened spectroscopical grade MeCN at λ = 395 nm shows minimal switching. **b** Irradiation of **PI** ($4.6x10^{-2}$ mM) in freshly opened spectroscopical grade MeCN at λ = 395 nm shows no switching. **c** Irradiation of **PI** ($4.4x10^{-2}$ mM) in MeCN with the addition of 1.5 eq. of *p*TsOH at λ = 395 nm shows significant switching.

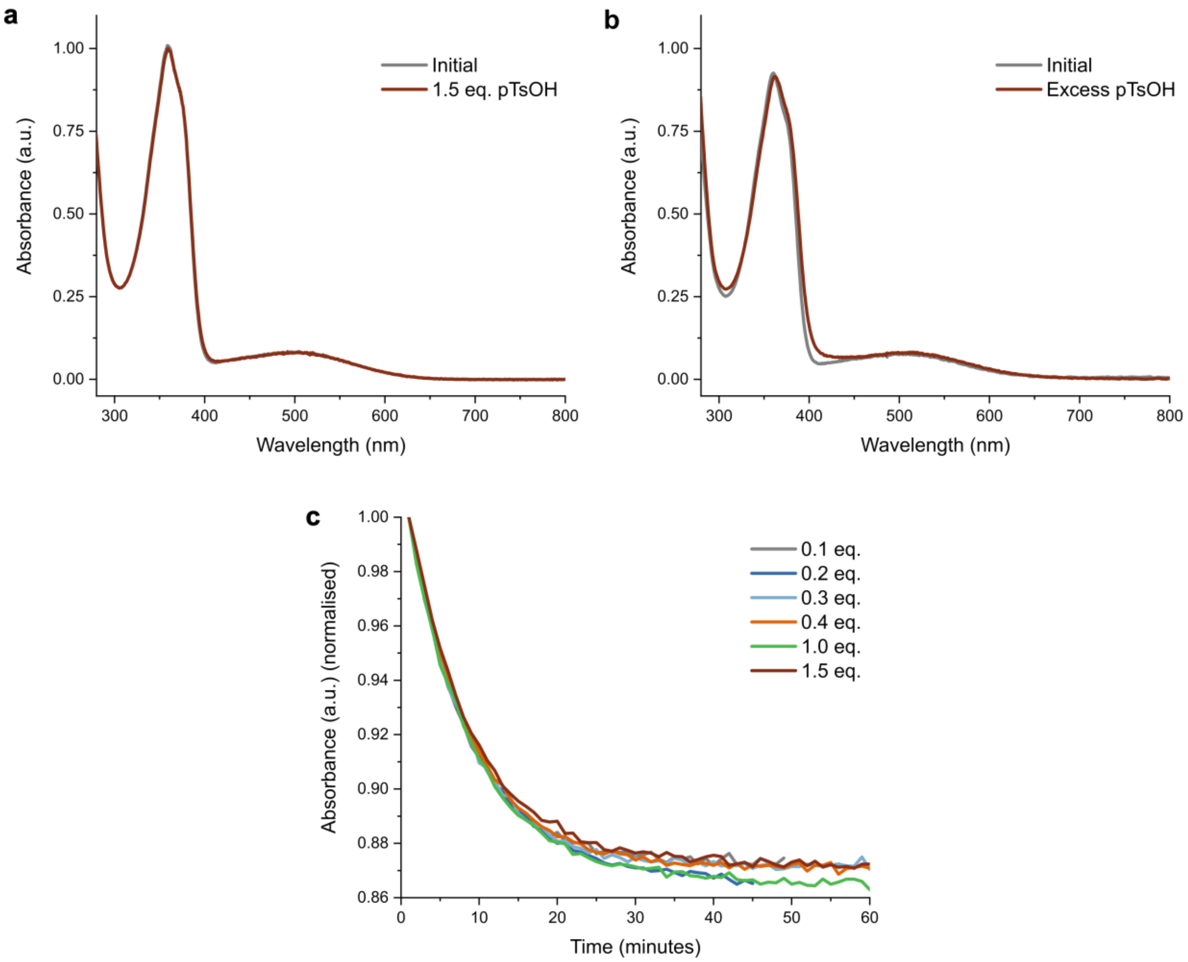


**Figure S53. a** The spectrum of **PI** ($4.4x10^{-2}$ mM) in over basic alumina-filtered MeCN (grey) compared to the normalised spectrum of **PI** ($4.4x10^{-2}$ mM) in over basic alumina-filtered MeCN and 1.5 eq. of *p*TsOH (maroon), the spectra do not change significantly. **b** The spectrum of **PI** ($4.2x10^{-2}$ mM) in over basic alumina-filtered MeCN (grey) compared to the normalised spectrum of **PI** in over basic alumina-filtered acetonitrile and an excess of *p*TsOH (maroon), the spectrum changes significantly. **c** The extent of PSS reached by $Irr_{365}$ adding different amounts of *p*TsOH; the amount of acid does not influence the PSS and acts catalytically.

## 9.3.2 Irradiation

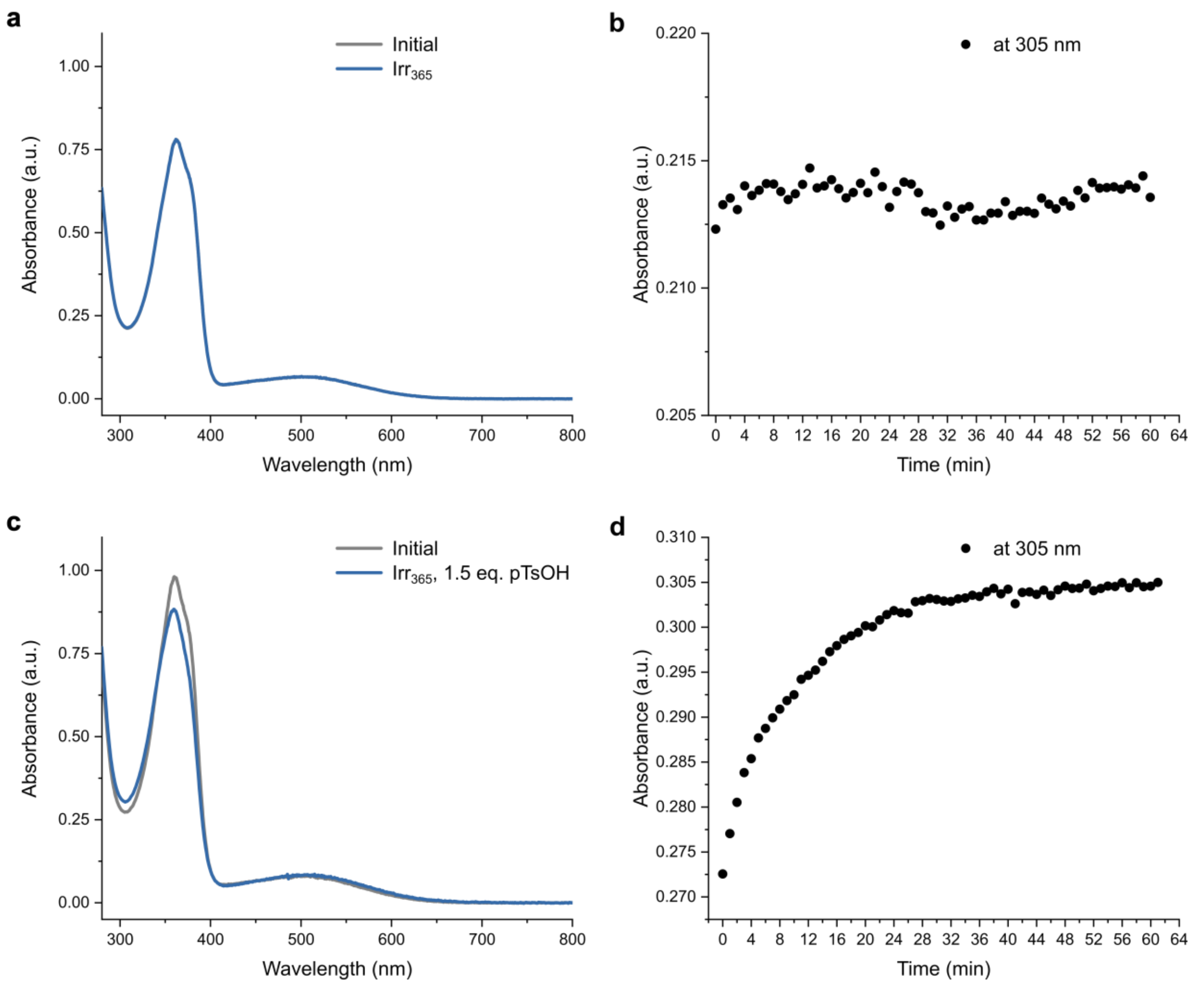


**Figure S54. a** Irradiation of **PI** ($3.5x10^{-2}$ mM) in MeCN at λ = 365 nm. **b** The evolution of the wavelength at λ = 305 nm at $Irr_{365}$. No photoisomerization is observed. **c** Reached PSS (blue) after irradiation of **PI** ($4.4x10^{-2}$ mM) in MeCN with 1.5 eq. of *p*TsOH at λ = 365 nm. Photoisomerization is observed (grey to blue) **d** The evolution of the wavelength at λ = 305 nm at $Irr_{365}$, a PSS is reached after 50 minutes.

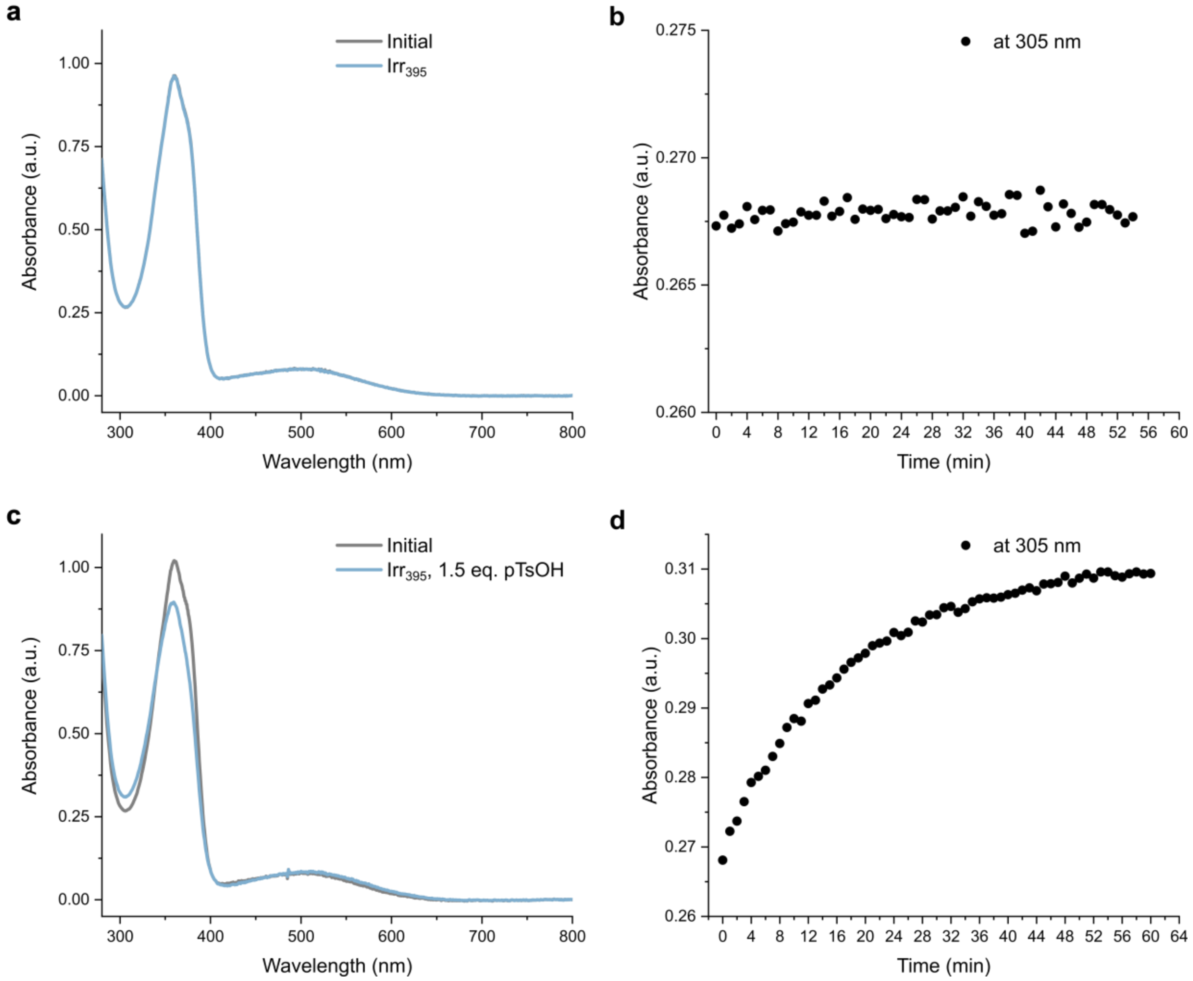


**Figure S55. a** Irradiation of **PI** ($4.4x10^{-2}$ mM) in MeCN at $\lambda$ = 395 nm. **b** The evolution of the wavelength at $\lambda$ = 305 nm at $Irr_{395}$. No photoisomerization is observed. **c** Reached PSS (blue) after irradiation of **PI** ($4.5x10^{-2}$ mM) in MeCN with 1.5 eq. of *p*TsOH at $\lambda$ = 395 nm. Photoisomerization is observed (grey to blue) **d** The evolution of the wavelength at $\lambda$ = 305 nm at $Irr_{395}$, a PSS is reached after 60 minutes.

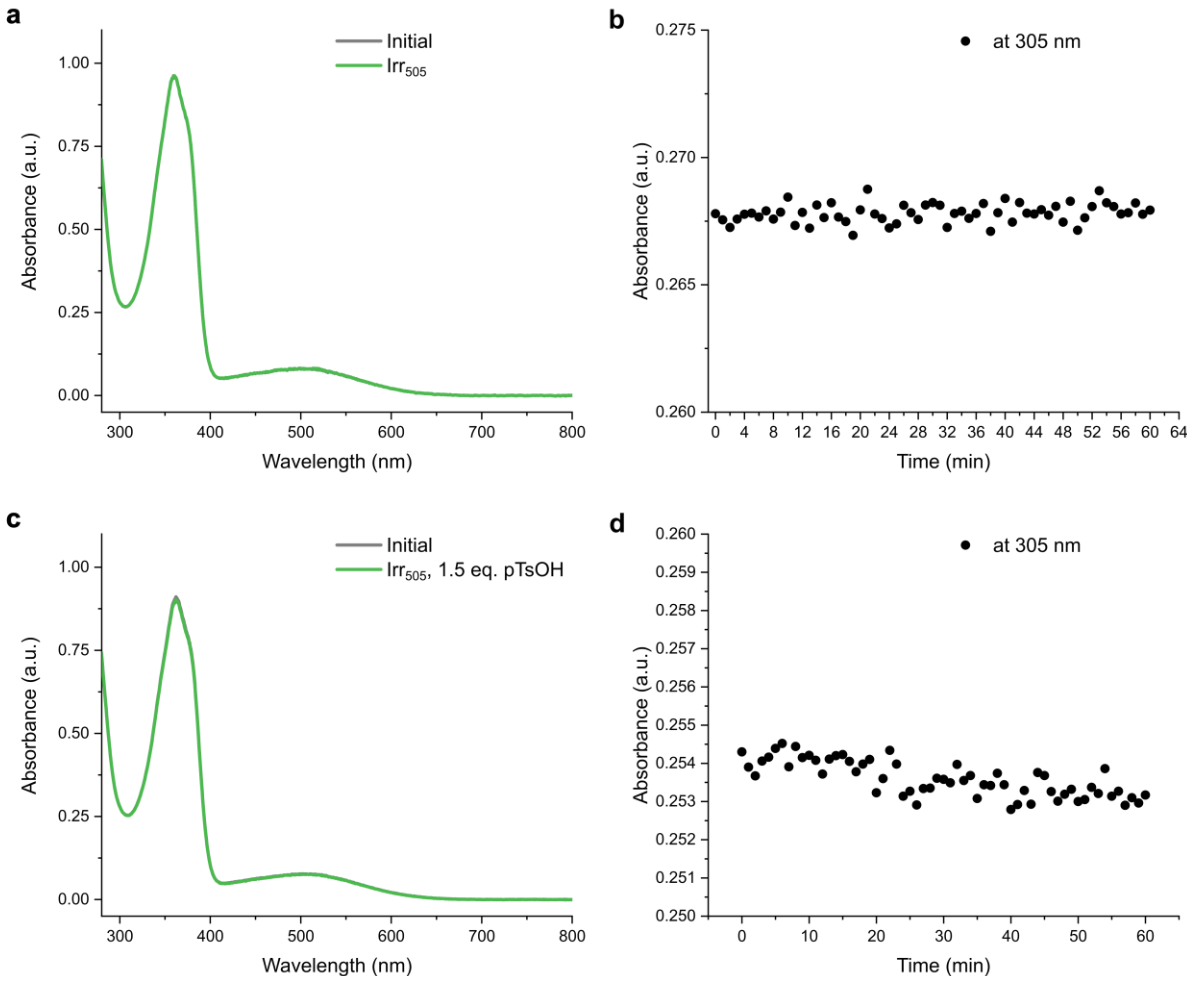


**Figure S56. a** Irradiation of **PI** ($4.3x10^{-2}$ mM) in MeCN at λ = 505 nm. **b** The evolution of the wavelength at λ = 305 nm at $Irr_{505}$. No photoisomerization is observed. **c** Irradiation of **PI** ($4.2x10^{-2}$ mM) in MeCN with 1.5 eq. of *p*TsOH at λ = 505 nm. **d** The evolution of the wavelength at λ = 305 nm at $Irr_{505}$. No photoisomerization is observed.

### 9.3.3 Irradiation with Various Acids

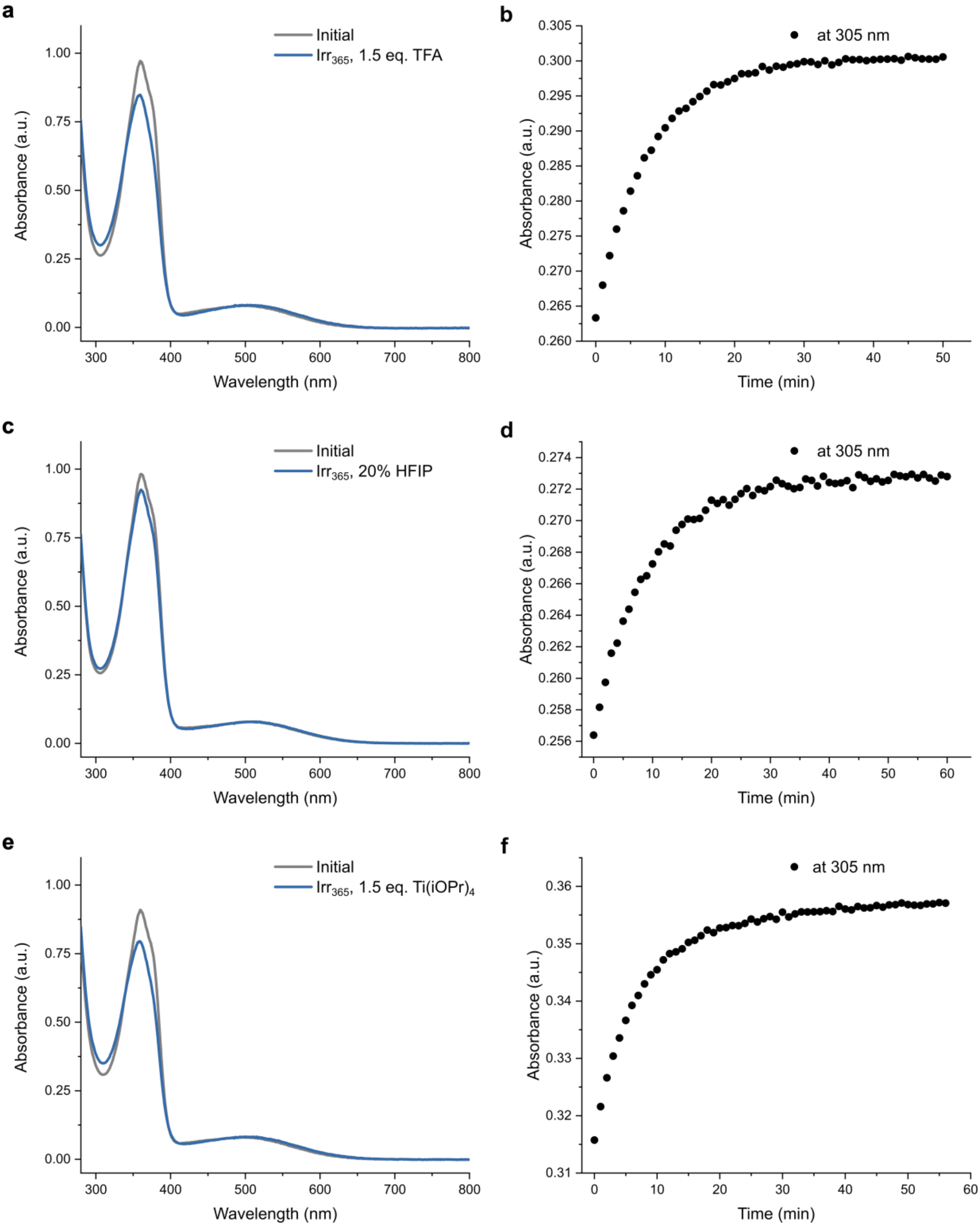


**Figure S57. a** Reached PSS (blue) after irradiation of **PI** (4.4x10$^{-2}$ mM) in MeCN with 1.5 eq. of trifluoroacetic acid (TFA) at λ = 365 nm. Photoisomerization is observed. **b** The evolution of wavelength at λ = 305 nm at $Irr_{365}$, a PSS is reached after 40 minutes. **c** Reached PSS (blue) after irradiation of **PI** (4.4x10$^{-2}$ mM) in MeCN with 20 volume% of HFIP at λ = 365 nm. Photoisomerization is observed. **d** The evolution of wavelength at λ = 305 nm at $Irr_{365}$, a PSS is reached after 50 minutes. **e** Reached PSS (blue) after irradiation of **PI** (4.3x10$^{-2}$ mM) in MeCN with 1.5 eq. of Ti(*i*OPr)$_4$ at λ = 365 nm. Photoisomerization is observed. **f** The evolution of wavelength at λ = 305 nm at $Irr_{365}$, a PSS is reached after 50 minutes.

## 9.3.4 Irradiation with Triflic Acid

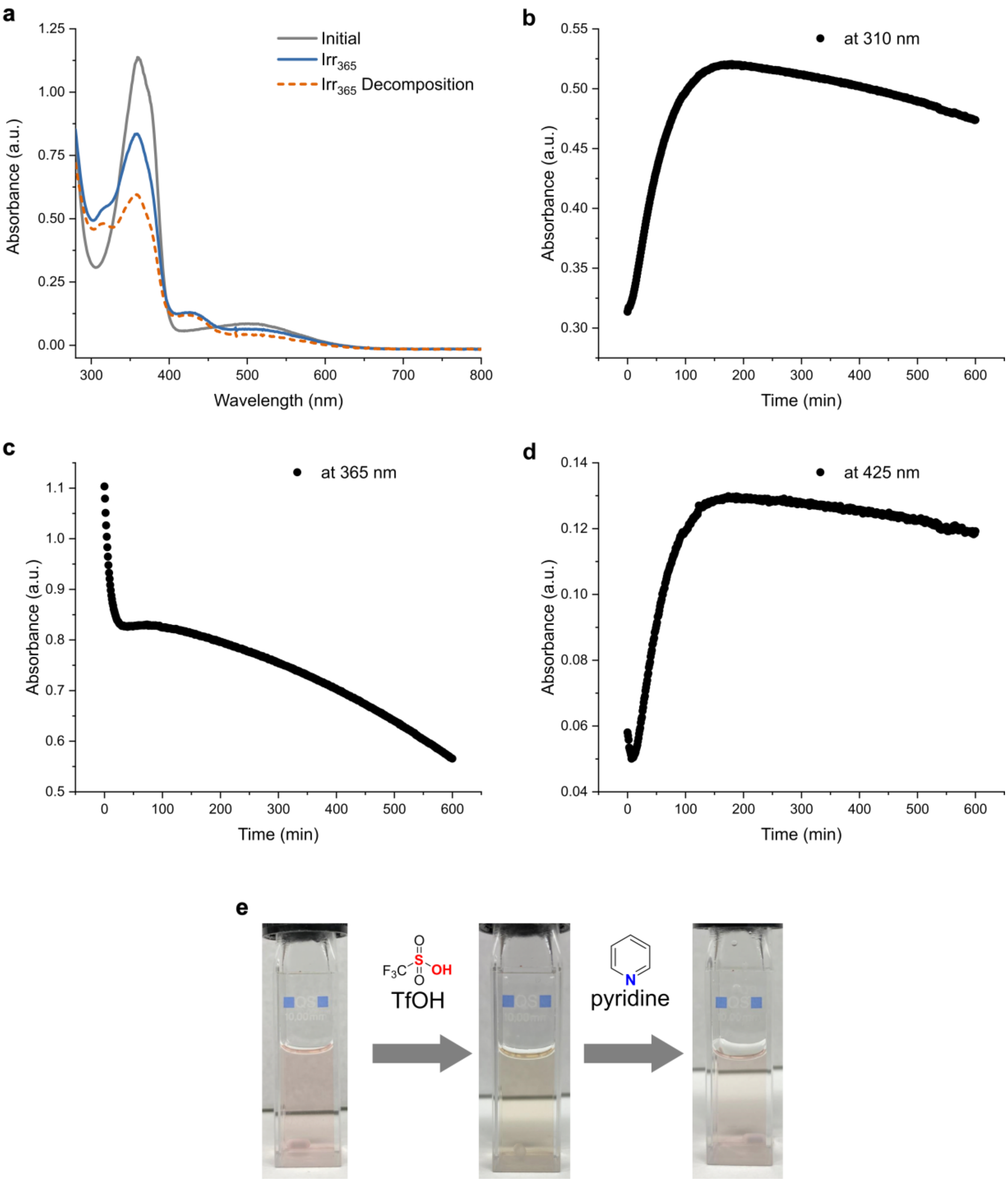


**Figure S58. a** Reached PSS (blue) and degradation (dashed orange) spectra after irradiation of **PI** ($5.0x10^{-2}$ mM) in anhydrous MeCN protonated by anhydrous triflic acid (TfOH) at $\lambda = 365$ nm. Initial photoisomerization is observed, followed by decomposition. **b** The evolution of the wavelength at $\lambda = 310$ nm at $Irr_{365}$. **c** The evolution of the wavelength at $\lambda = 365$ nm at $Irr_{365}$. **d** The evolution of the wavelength at $\lambda = 425$ nm at $Irr_{365}$. In comparison to other acids, two new bands appear at $\lambda = 310$ and 425 nm, followed by their decomposition. **e** Before $Irr_{365,}$ the colour in the cuvette is purple; after $Irr_{365}$ with TfOH, the colour changes to yellow. Addition of pyridine after $Irr_{365}$ reverses the colour back to purple; the intensity is significantly less, in line with the observed degradation.

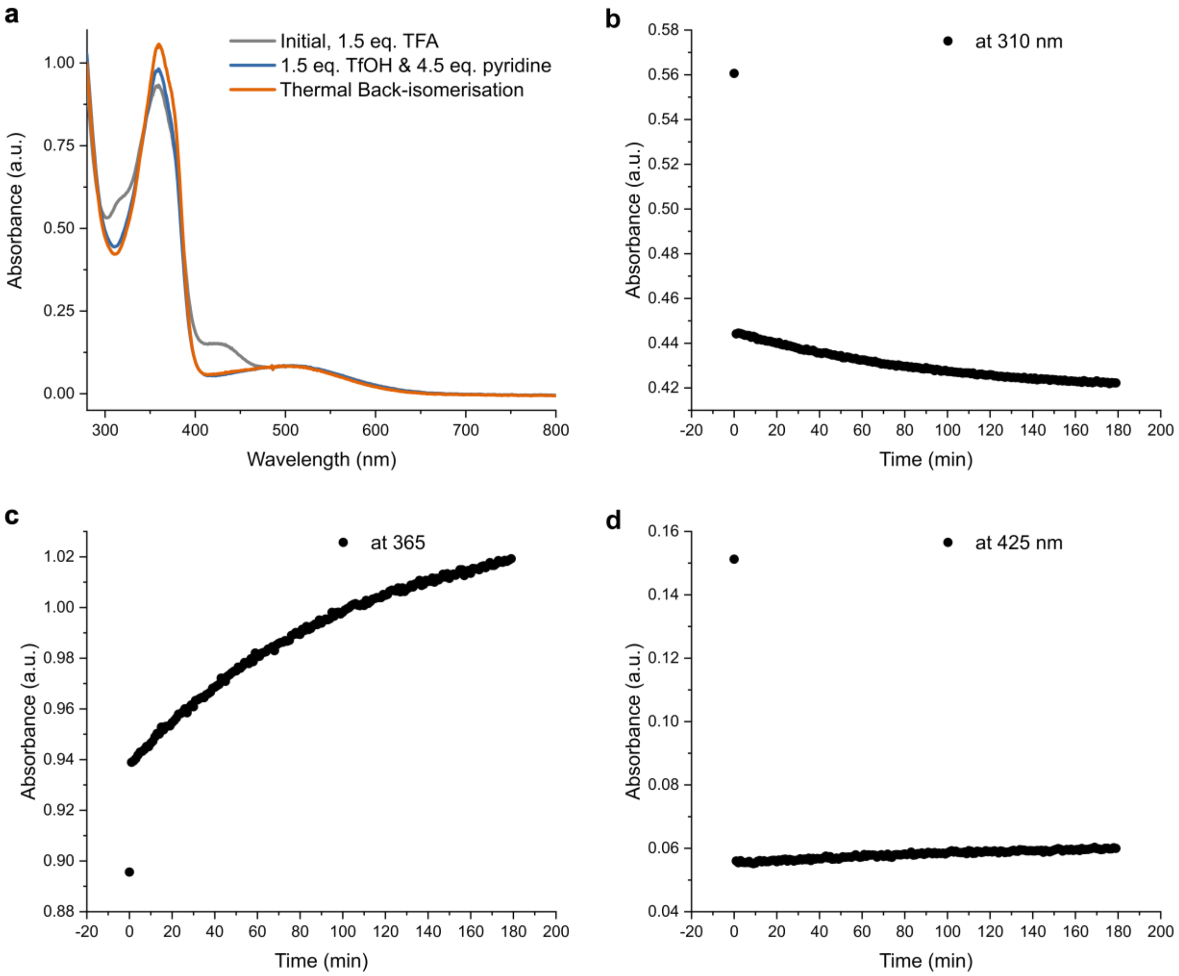


**Figure S59. a** The absorption spectra of the $PSS_{365}$ (grey) $H^+$-**PI** (triflic acid, TfOH) after the addition of 4.5 eq. of anhydrous pyridine (blue) and thermal back-isomerization (orange). (For information about the addition of bases/nucleophiles, see **Section 14**). **b**, **c** and **d** show the evolution of the wavelength at $\lambda$ = 310, 365 and 425 nm, respectively, after the addition of 4.5 eq. of pyridine. After the addition of pyridine, the bands at $\lambda$ = 310 and 425 nm disappear instantaneously and are followed by thermal back-isomerization to the stable state of **PI**.

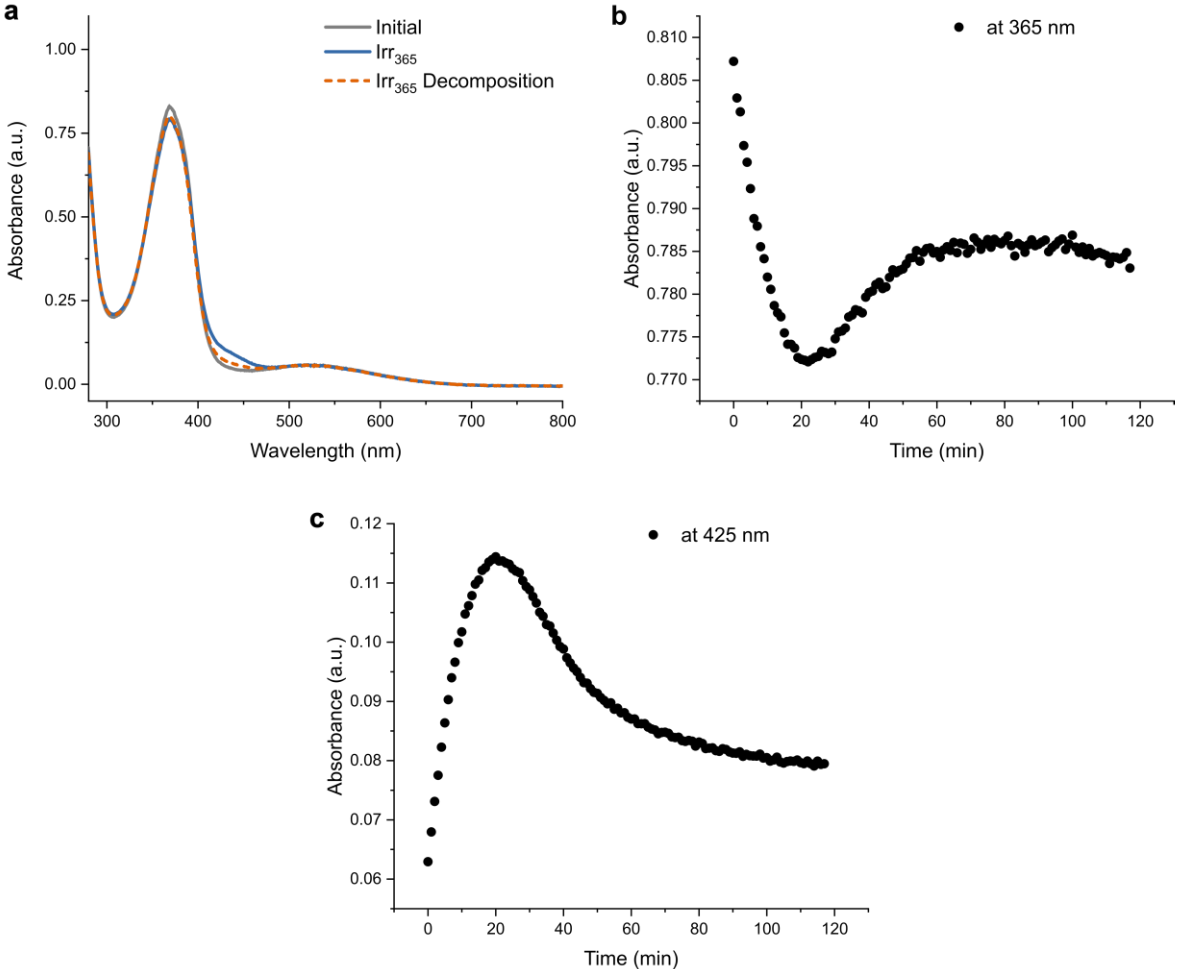


**Figure S60. a** Reached PSS (blue) and decomposition (dashed orange) spectra after irradiation of **PI** ($3.5x10^{-2}$ mM) in spectroscopical grade MeCN protonated by anhydrous triflic acid at $\lambda$ = 365 nm. **b** The evolution of the wavelength at $\lambda$ = 365 nm at $Irr_{365}$. **c** The evolution of the wavelength at $\lambda$ = 425 nm at $Irr_{365}$. In comparison to anhydrous MeCN, only the growth of the band at $\lambda$ = 425 nm is observed, which is followed by decomposition.

### 9.3.5 Effect of Water

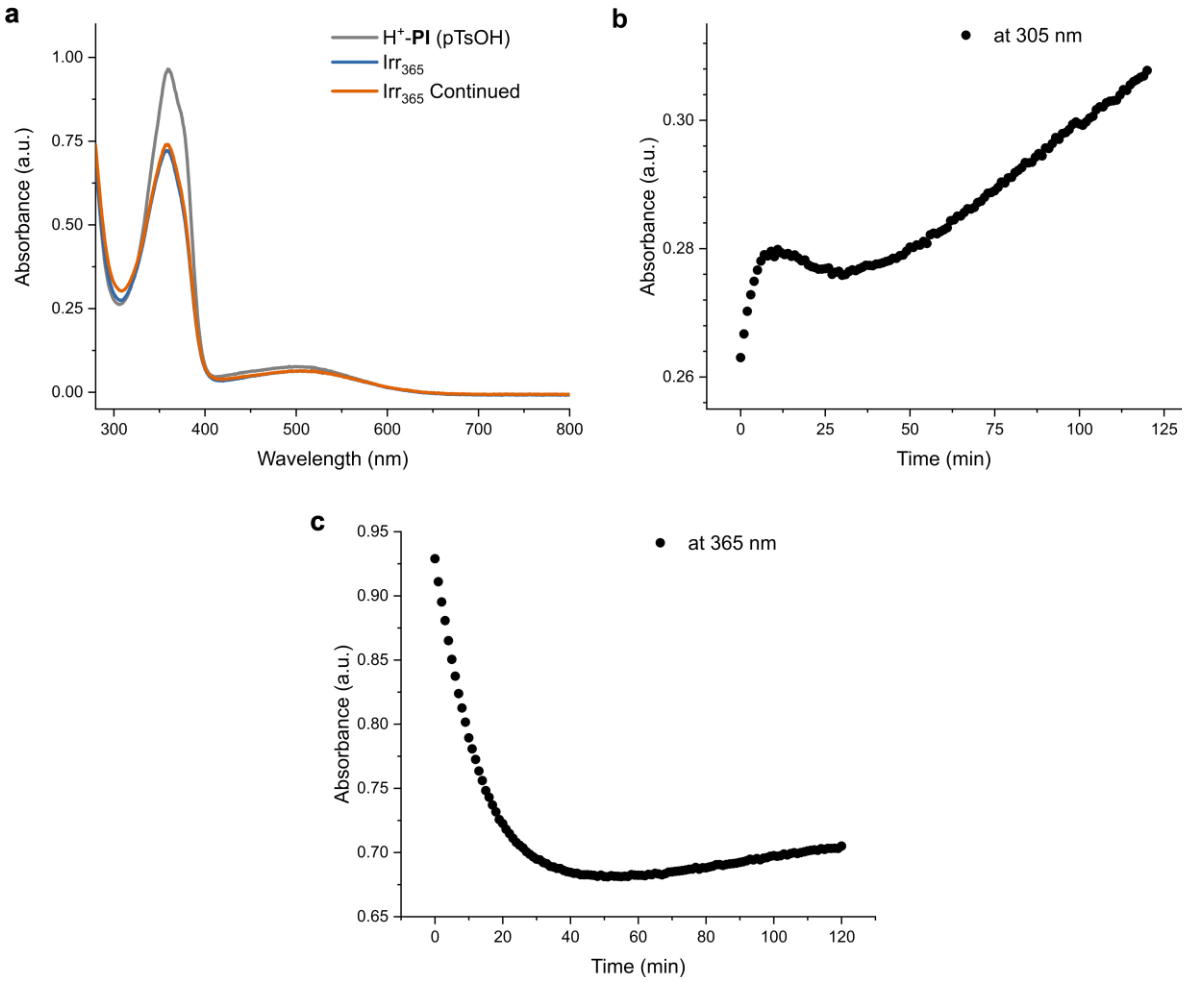


**Figure S61. a** Reached PSS (blue) and continuation (orange) after the irradiation of H$^+$-**PI** (1.5 eq. *p*TsOH) in anhydrous MeCN at λ = 365 nm. **b** The evolution of the wavelength at λ = 305 nm at $Irr_{365}$. **c** The evolution of the wavelength at λ = 365 nm. H$^+$-**PI** initially shows photoisomerization, accompanied by a blue shift in the isosbestic point, indicating decomposition over time.

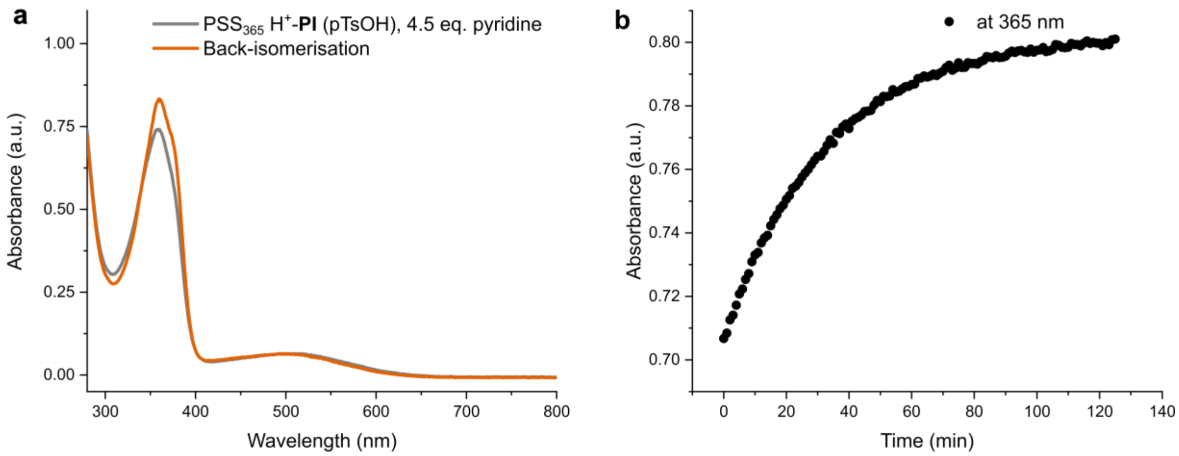


**Figure S62. a** The $PSS_{365}$ (grey) of $H^+$-**PI** (1.5 eq. *p*TsOH) in anhydrous MeCN and the absorption spectrum of **PI** after back-isomerization upon addition of 4.5 eq. of anhydrous pyridine at the $PSS_{365}$. (For information about the addition of bases/nucleophiles, refer to **Section 14**). **b** The evolution of the wavelength at $\lambda$ = 365 nm during the back-isomerization. After the addition of pyridine, **PI** back-isomerises to the stable state; however, the recovery is not full (Refer to **Figure S50a**), indicating the decomposition of **PI** in dry anhydrous conditions.

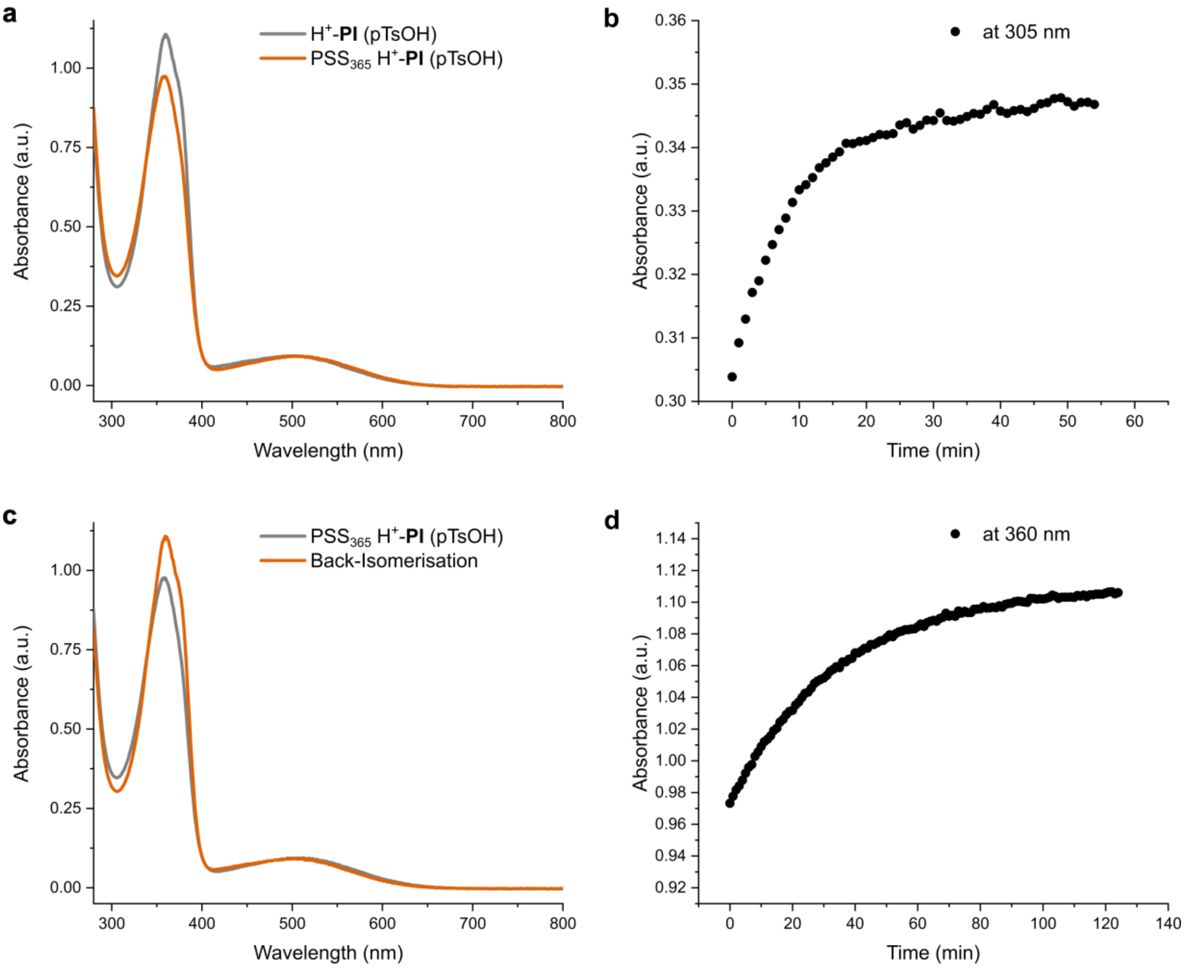


**Figure S63. a** The reached PSS (orange) after irradiation of $H^+$-**PI** ($4.8x10^{-2}$ mM) (1.5 eq. *p*TsOH) in spectroscopical grade MeCN at $\lambda$ = 365 nm. **b** The evolution of the wavelength at $\lambda$ = 305 nm at $Irr_{365}$. A PSS is reached after 50 minutes. **c** The reached absorption spectrum (orange) of **PI** after back-isomerization upon addition of 4.5 eq. of anhydrous pyridine. (For information about the addition of bases/nucleophiles, refer to **Section 14**). **d** The evolution of the wavelength at $\lambda$ = 360 nm during the back-isomerization. **PI** back-isomerises to the stable state, and the recovery is full, indicating no decomposition under spectroscopical grade conditions (i.e. ambient conditions). Highlighting the need for the presence of $H_2O$ in the overall system.

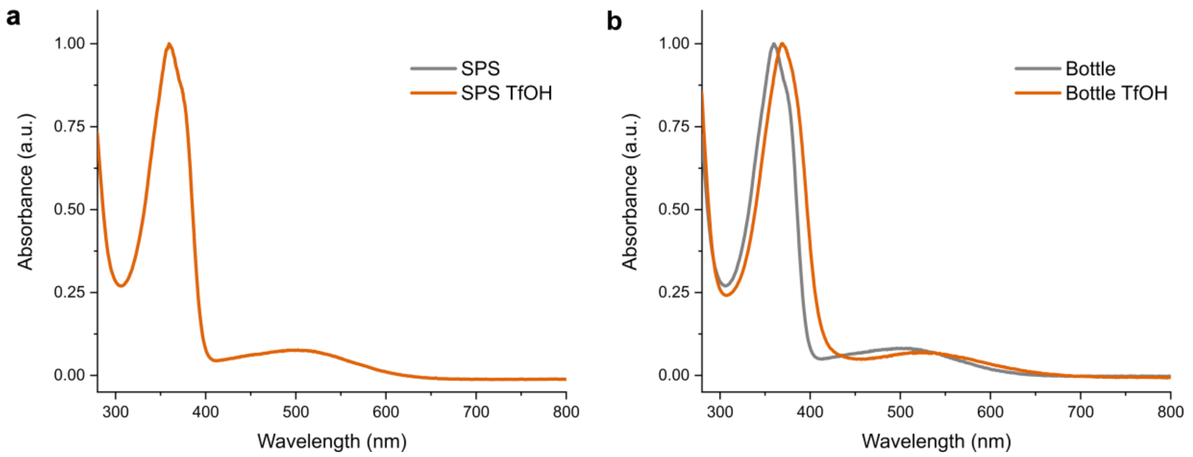


**Figure S64. a** The normalised absorption spectra of **PI** and H$^+$-**PI** (1.5 eq. triflic acid) in anhydrous acetonitrile. The two absorption spectra fully overlap. **b** The normalised absorption spectra of **PI** and H$^+$-**PI** (1.5 eq. triflic acid) in spectroscopical grade MeCN. The spectrum of H$^+$-**PI** shows a bathochromic shift of $\Delta\lambda = 9$ nm (360 to 369 nm, respectively), indicating the influence of water on the extent of direct protonation of **PI** in MeCN (Refer to **Section 10** for information about the protonation of **PI**).

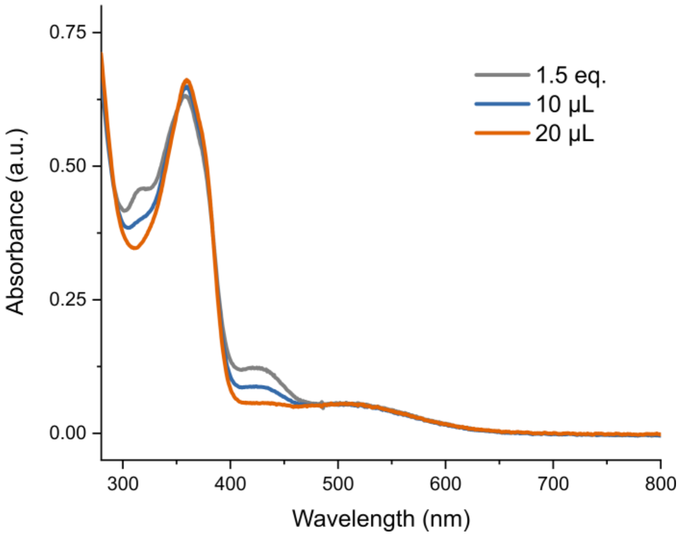


**Figure S65.** The normalised absorption spectra of the addition of varying amounts of $H_2O$ to irradiated ($\lambda = 365$ nm) H$^+$-**PI** (1.5 eq. triflic acid, grey) in anhydrous MeCN. The addition of increasing amounts of water (blue, orange) leads to the disappearance of the bands at $\lambda = 310$ and 425 nm, respectively. Indicating the extent of immediate protonation is diminished by increasing equivalents of $H_2O$ (Refer to **Section 10** for information about the protonation of **PI**).

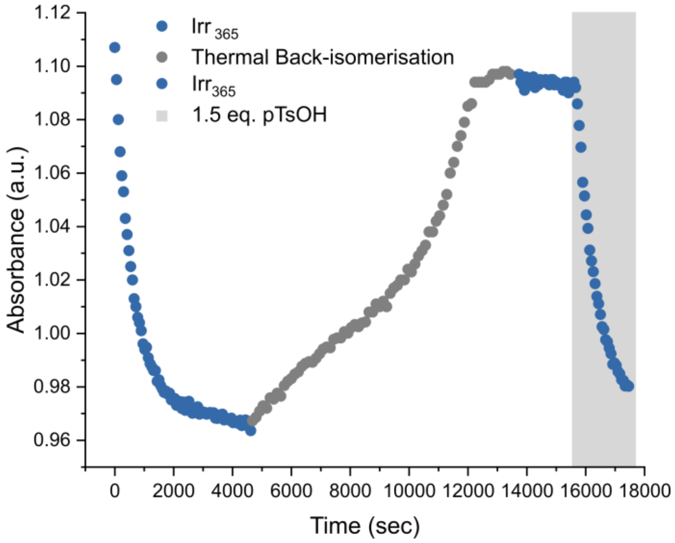


**Figure S66.** Shows The evolution of the wavelength at λ = 360 nm of **PI** in MeCN during $Irr_{365}$ and thermal back-isomerization. The initial trace (blue) shows the irradiation of **PI** with 1.5 eq. of *p*TsOH at λ = 365 nm until a PSS was reached. The second trace (grey) shows the thermal back-isomerization of **PI**, which was followed by subsequent irradiation with λ = 365 nm (straight blue trace). After thermal back-isomerization, the **PI** did not photoisomerise towards the metastable state, and the acid in the system had been consumed by water. Another 1.5 eq. of *p*TsOH (light grey box) was added, and the molecule photoisomerised immediately. Indicating that the water acts as a base and deprotonates the **PI**.

### 9.3.6 PSS Modulation

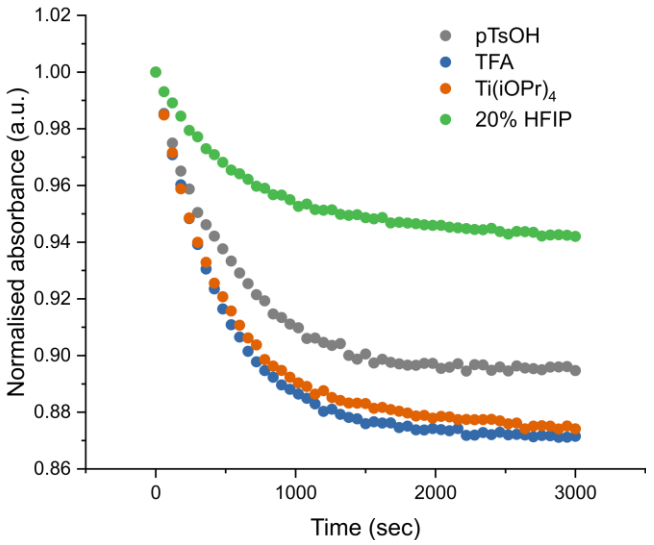


**Figure S67.** The normalised traces were monitored at λ = 360 nm for the irradiation of **PI** at λ = 365 nm, in combination with a variety of acids (*p*TsOH, trifluoroacetic acid (TFA), Ti(*i*OPr)$_4$ and 20% HFIP). The spectra show that different PSSs are reached by varying the acid used in the system, highlighting the ability to modulate the PSS.

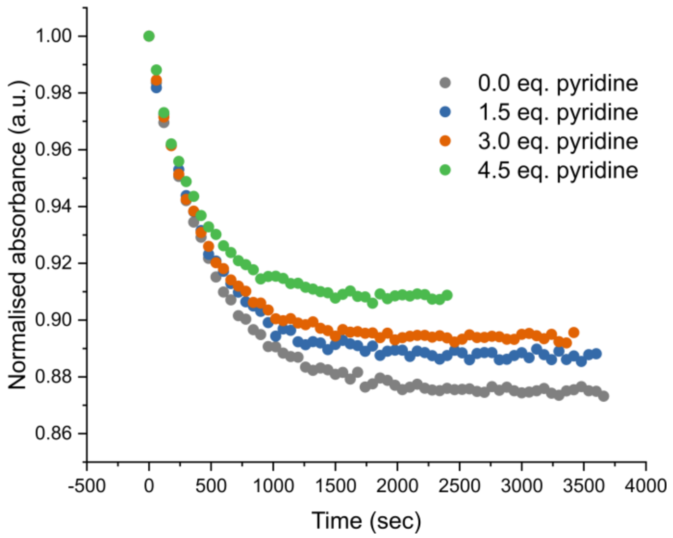


**Figure S68.** The normalised traces were monitored at λ = 360 nm for the irradiation of H$^+$-**PI** (1.5 eq. of *p*TsOH) at λ = 365 nm, and increasing equivalents of pyridine. The spectra show that different PSSs are reached with varying amounts of pyridine present in the system, highlighting the ability to modulate the PSS. (For information about the addition of bases/nucleophiles, refer to **Section 14**)

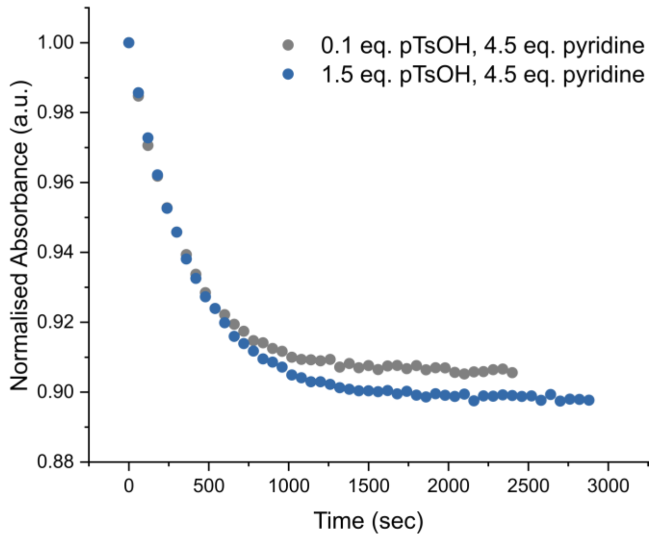


**Figure S69.** The normalised traces were monitored at λ = 360 nm for the irradiation of $H^+$-**PI** (4.5 eq. of pyridine) at λ = 365 nm, and varying equivalents of *p*TsOH. The spectra show that different PSSs are reached with varying amounts of acid present in combination with pyridine in the system, highlighting the ability to modulate the PSS. (For information about the addition of bases/nucleophiles, refer to **Section 14**)

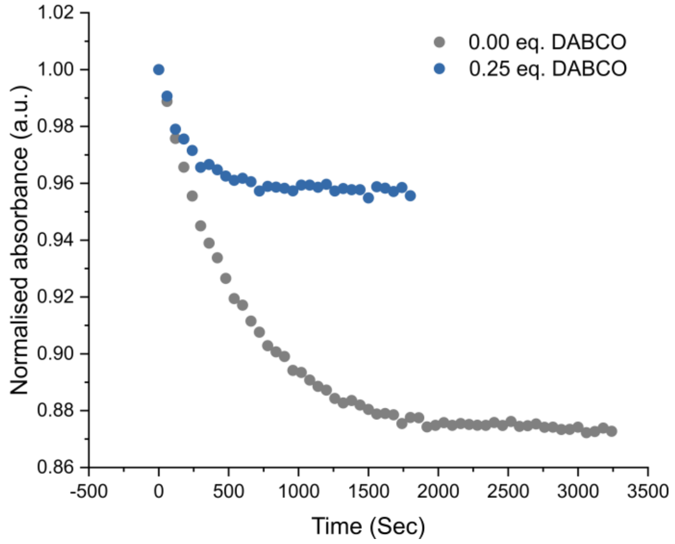


**Figure S70.** The normalised traces were monitored at λ = 360 nm for the irradiation of $H^+$-**PI** (1.5 eq. of *p*TsOH) at λ = 365 nm, compared to the presence of 0.25 eq. of DABCO. The spectra show that different PSSs are reached by adding DABCO to the system, highlighting the ability to modulate the PSS. (For information about the addition of bases/nucleophiles, refer to **Section 14**)

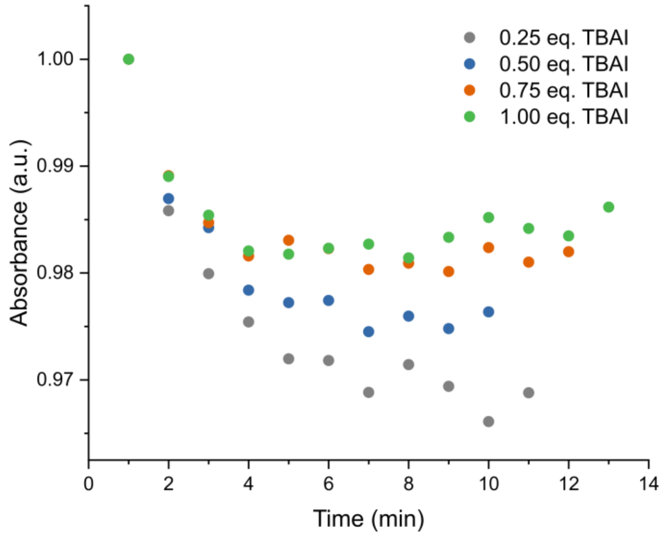


**Figure S71.** The normalised traces were monitored at λ = 360 nm for the irradiation of $H^{+}$-**PI** (1.5 eq. of *p*TsOH) at λ = 365 nm, and increasing equivalents of TBAI. The spectra show that different PSSs are reached by adding TBAI to the system, highlighting the ability to modulate the PSS. (For information about the addition of bases/nucleophiles, refer to **Section 14**)

## 9.3.7 Control Experiments

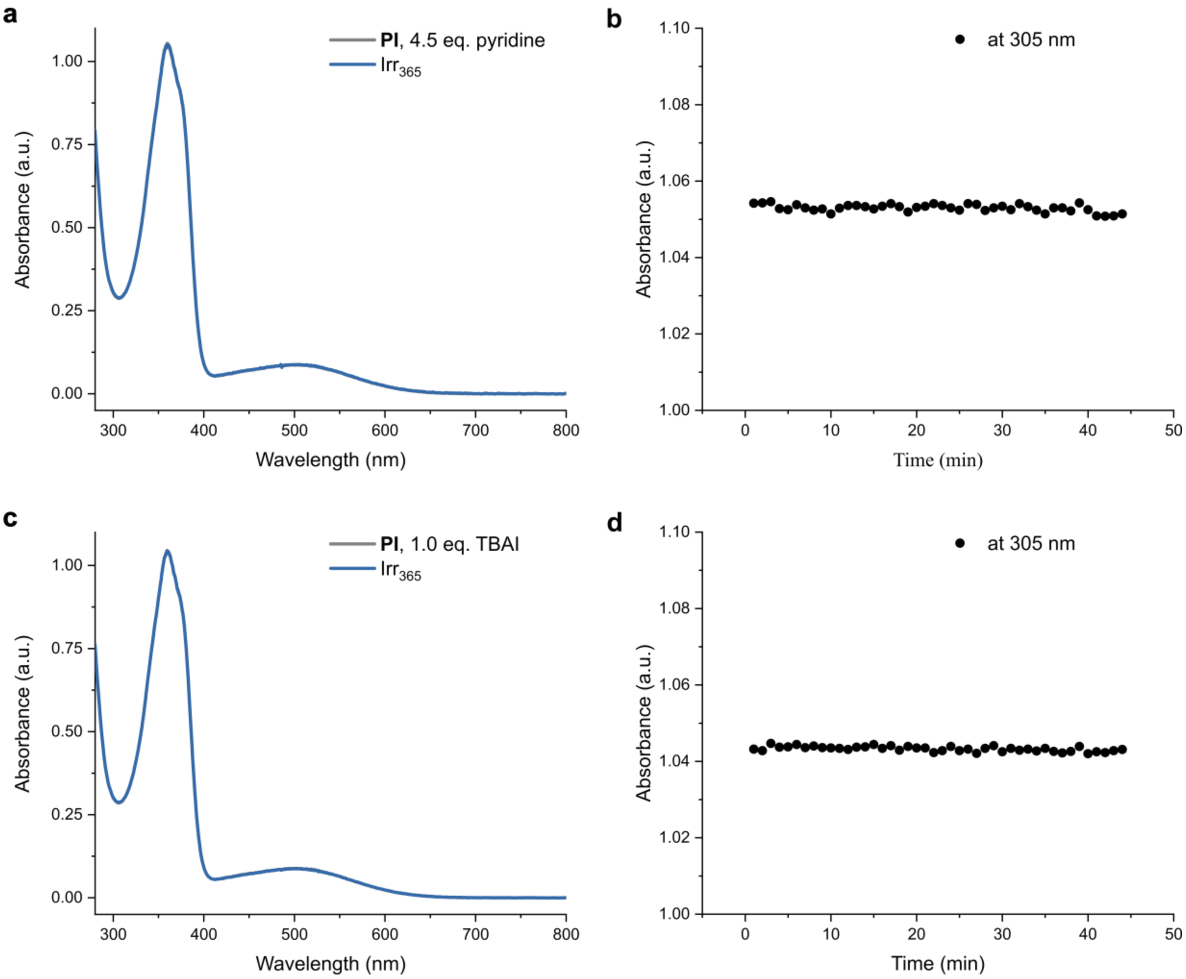


**Figure S72. a** The absorption spectrum of **PI** with 4.5 eq. of pyridine in MeCN irradiated at $\lambda = 365$ nm. **b** The evolution of the wavelength at 305 nm at $Irr_{365}$. Shows that **PI** is stable in the presence of pyridine when irradiated. **c** The absorption spectrum of **PI** with 1.0 eq. of TBAI in MeCN irradiated at $\lambda = 365$ nm. **d** The evolution of the wavelength at 305 nm at $Irr_{365}$. Shows that **PI** is stable in the presence of TBAI when irradiated. (For information about the addition of bases/nucleophiles, refer to **Section 14**). These experiments indicate that the mere presence of a nucleophile in combination with irradiation does not switch the **PI**.

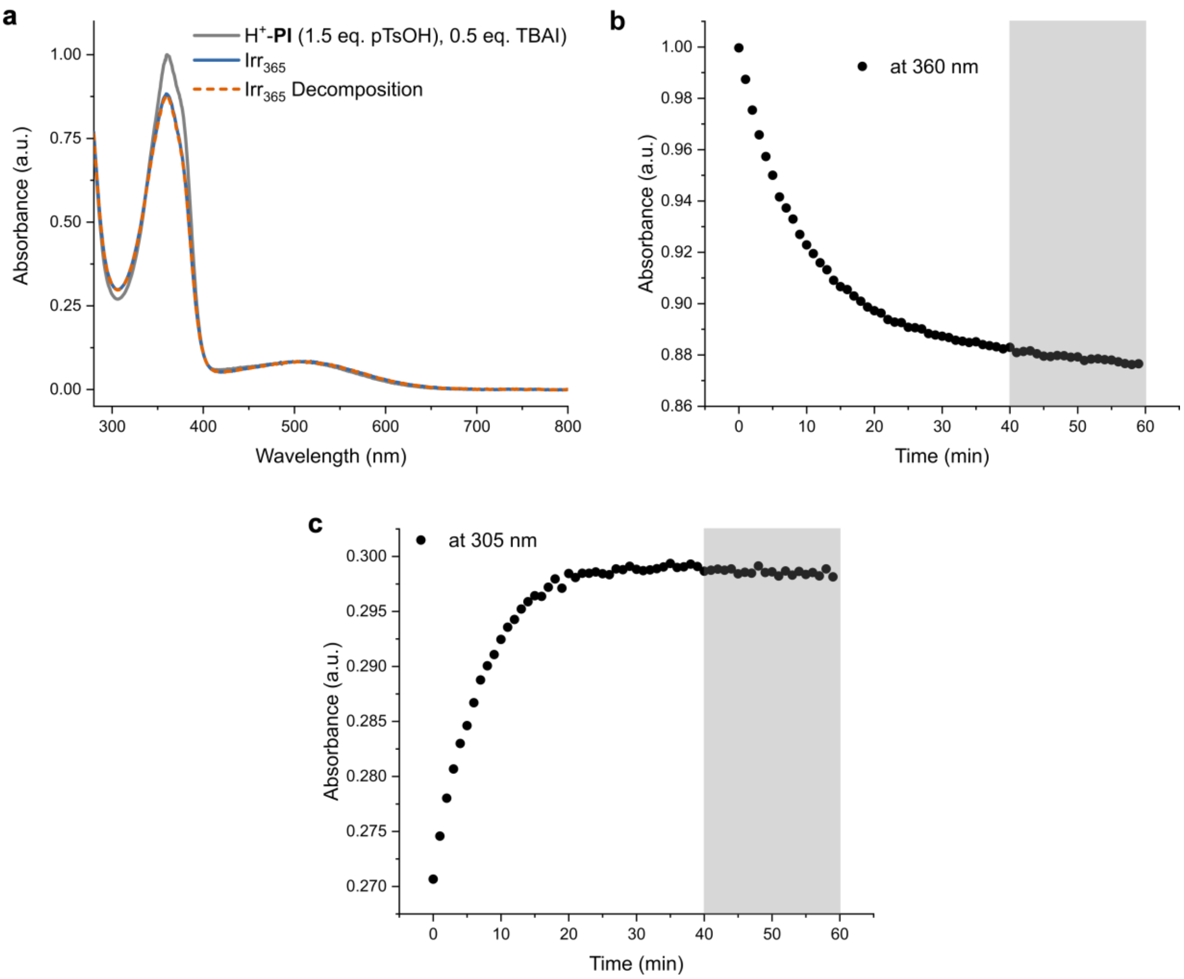


**Figure S73.** **a** The reached PSS (blue) and decomposition (dashed orange) after irradiation of $H^+$-**PI** ($4.4x10^{-2}$ mM) (1.5 eq. *p*TsOH and 0.5 eq. TBAI) in spectroscopical grade MeCN at $\lambda$ = 365 nm. **b** The evolution of the wavelength at $\lambda$ = 360 nm at $Irr_{365}$, where the light grey rectangle shows the decomposition of **PI**. **c** The evolution of the wavelength at $\lambda$ = 305 nm at $Irr_{365}$, where the light grey rectangle shows the decomposition of **PI**. A PSS is reached after 40 minutes; prolonged irradiation shows degradation. (For information about the addition of bases/nucleophiles, refer to **Section 14**).

## 9.4 Methanol

### 9.4.1 Irradiation

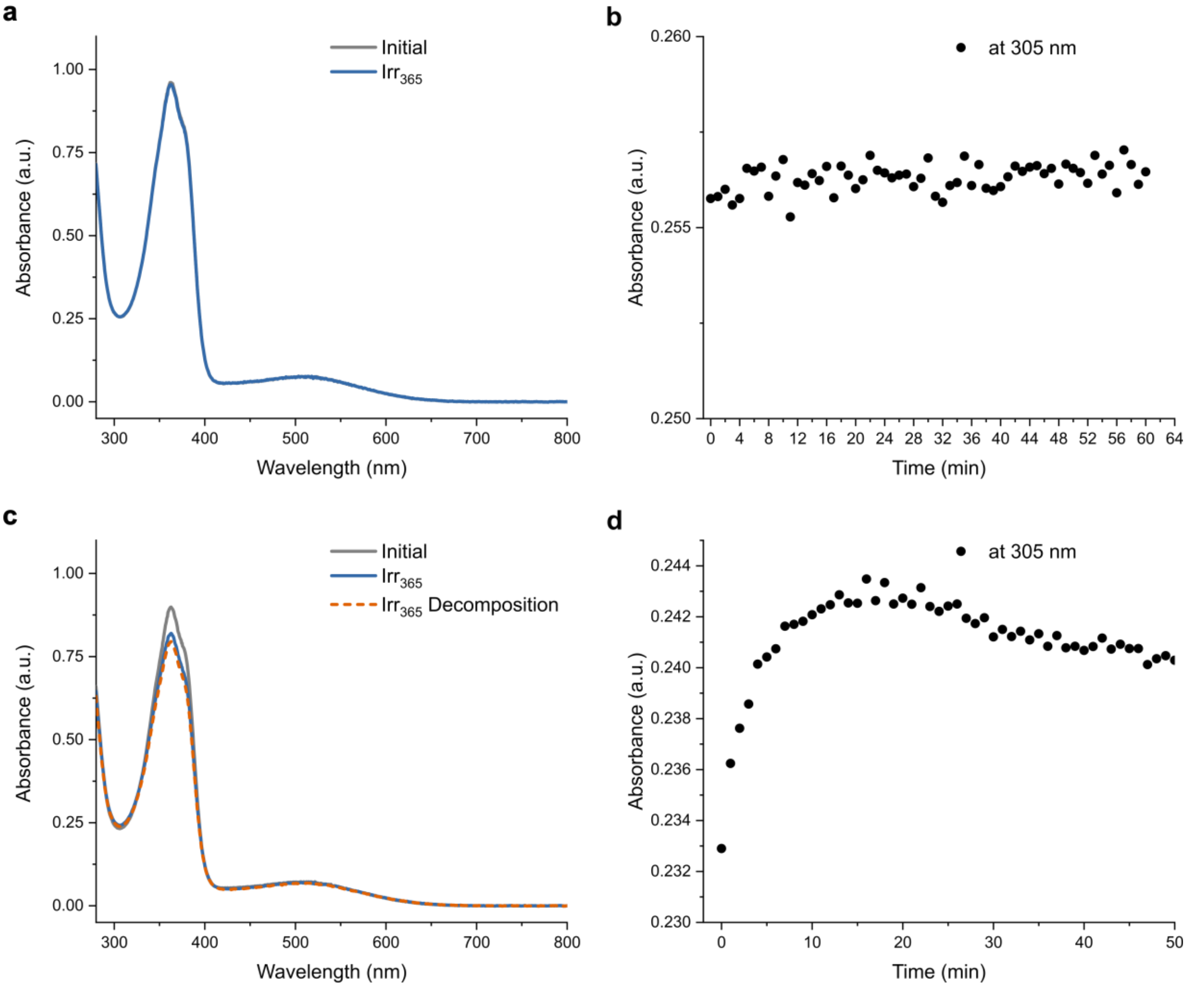


**Figure S74. a** Irradiation of **PI** ($4.5x10^{-2}$ mM) in MeOH at λ = 365 nm. **b** The evolution of the wavelength at λ = 305 nm at $Irr_{365}$. No photoisomerization is observed. **c** Reached PSS (blue) and degradation (dashed orange) after irradiation of **PI** ($4.3x10^{-2}$ mM) in MeOH with 1.5 eq. of *p*TsOH at λ = 365 nm. **d** The evolution of the wavelength at λ = 305 nm at $Irr_{365}$. Photoisomerization (grey to blue) is observed over 20 minutes, reaching a PSS, followed by degradation (blue to dashed orange).

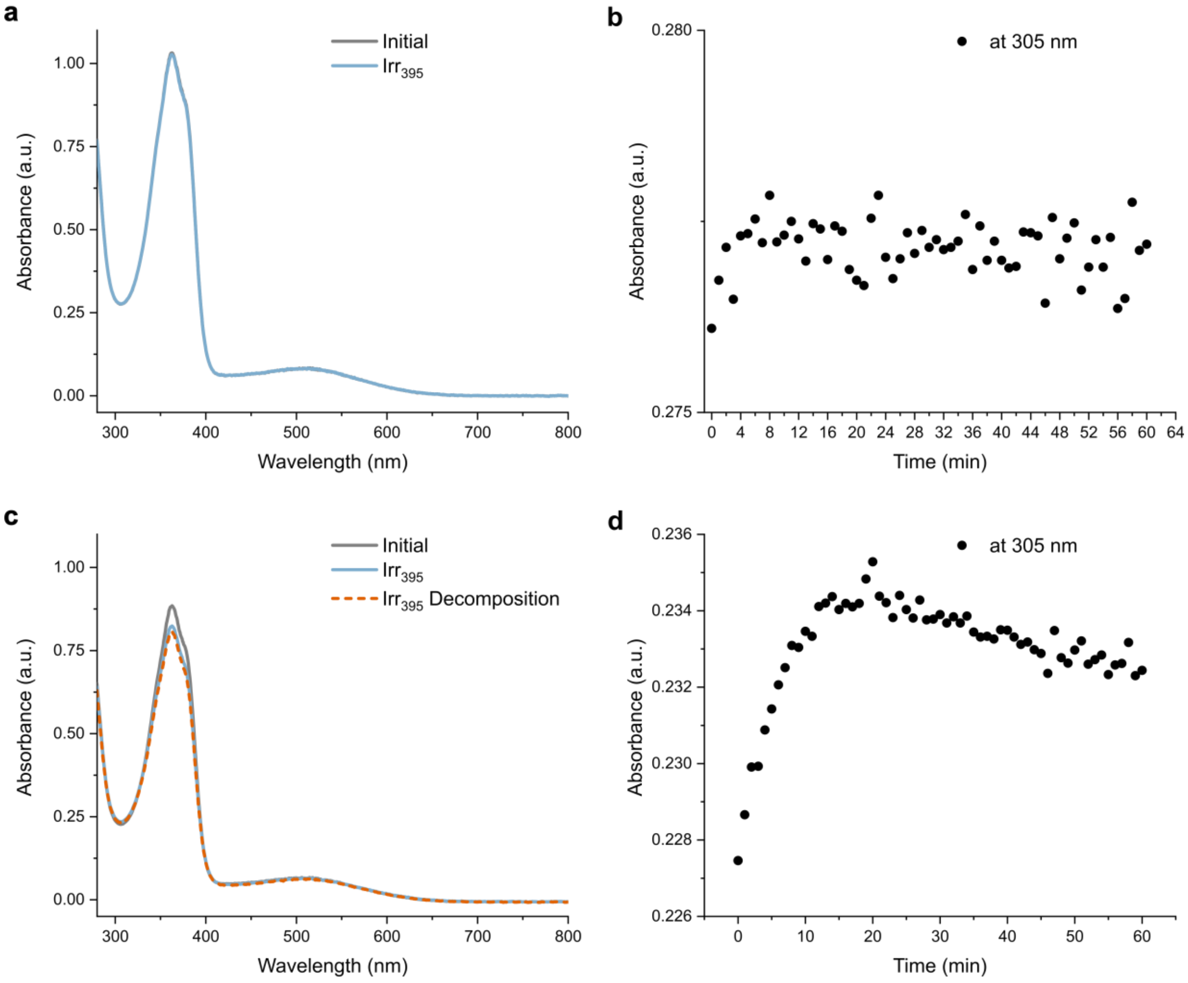


**Figure S75.** **a** Irradiation of **PI** ($4.5x10^{-2}$ mM) in MeOH at λ = 395 nm. **b** The evolution of the wavelength at λ = 305 nm at $Irr_{395}$. No photoisomerization is observed. **c** Reached PSS (light blue) and degradation (dashed orange) after irradiation of **PI** ($4.3x10^{-2}$ mM) in MeOH with 1.5 eq. of *p*TsOH at λ = 395 nm. **d** The evolution of the wavelength at λ = 305 nm at $Irr_{395}$. Photoisomerization (grey to light blue) is observed over 20 minutes, reaching a PSS, followed by degradation (light blue to dashed orange).

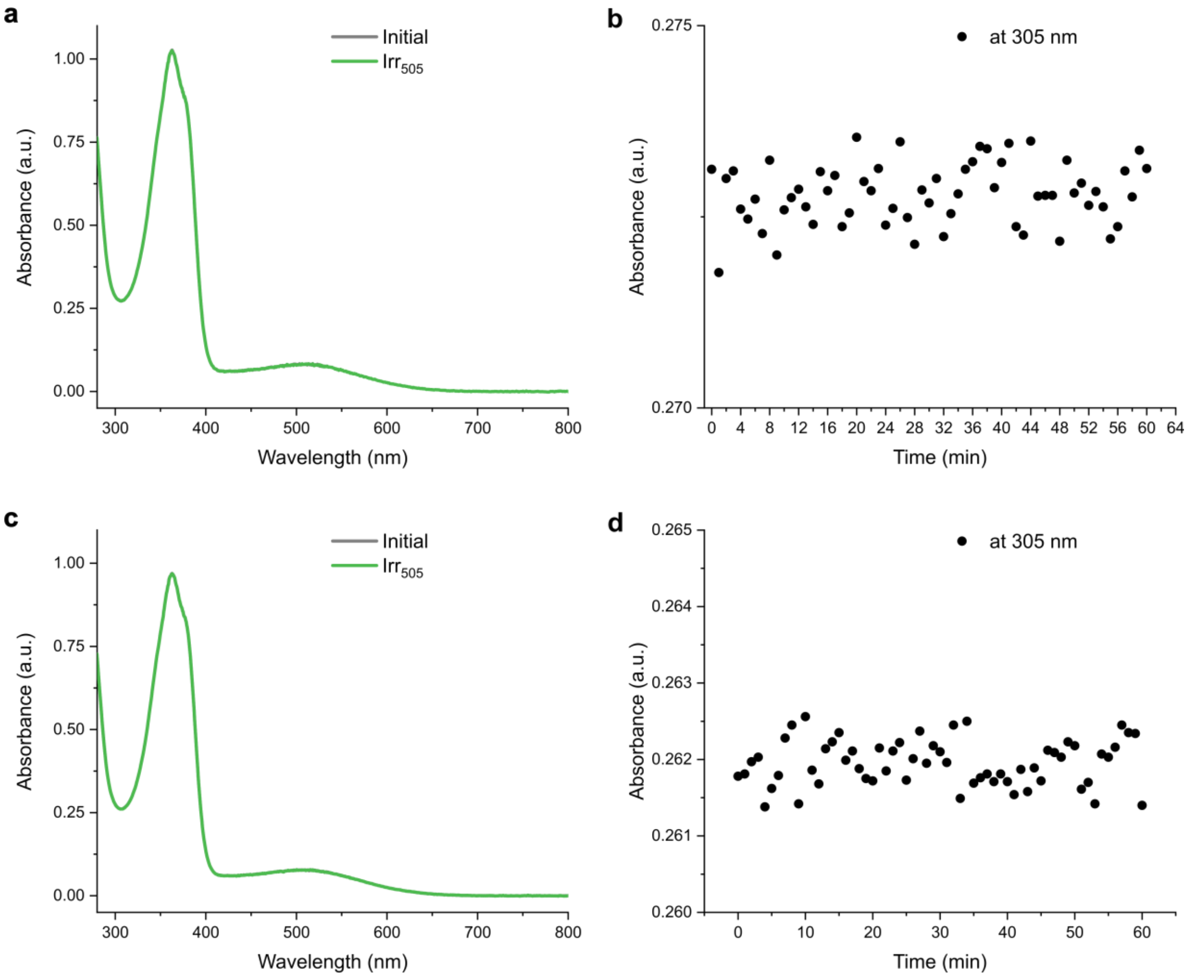


**Figure S76. a** Irradiation of **PI** ($4.4x10^{-2}$ mM) in MeOH at λ = 505 nm. **b** The evolution of the wavelength at λ = 305 nm at $Irr_{505}$. No photoisomerization is observed. **c** Irradiation of **PI** ($4.4x10^{-2}$ mM) in MeOH with 1.5 eq. of *p*TsOH at λ = 505 nm. **d** The evolution of the wavelength at λ = 305 nm at $Irr_{505}$. No photoisomerization is observed.

## 9.4.2 Argon and Water Dependency

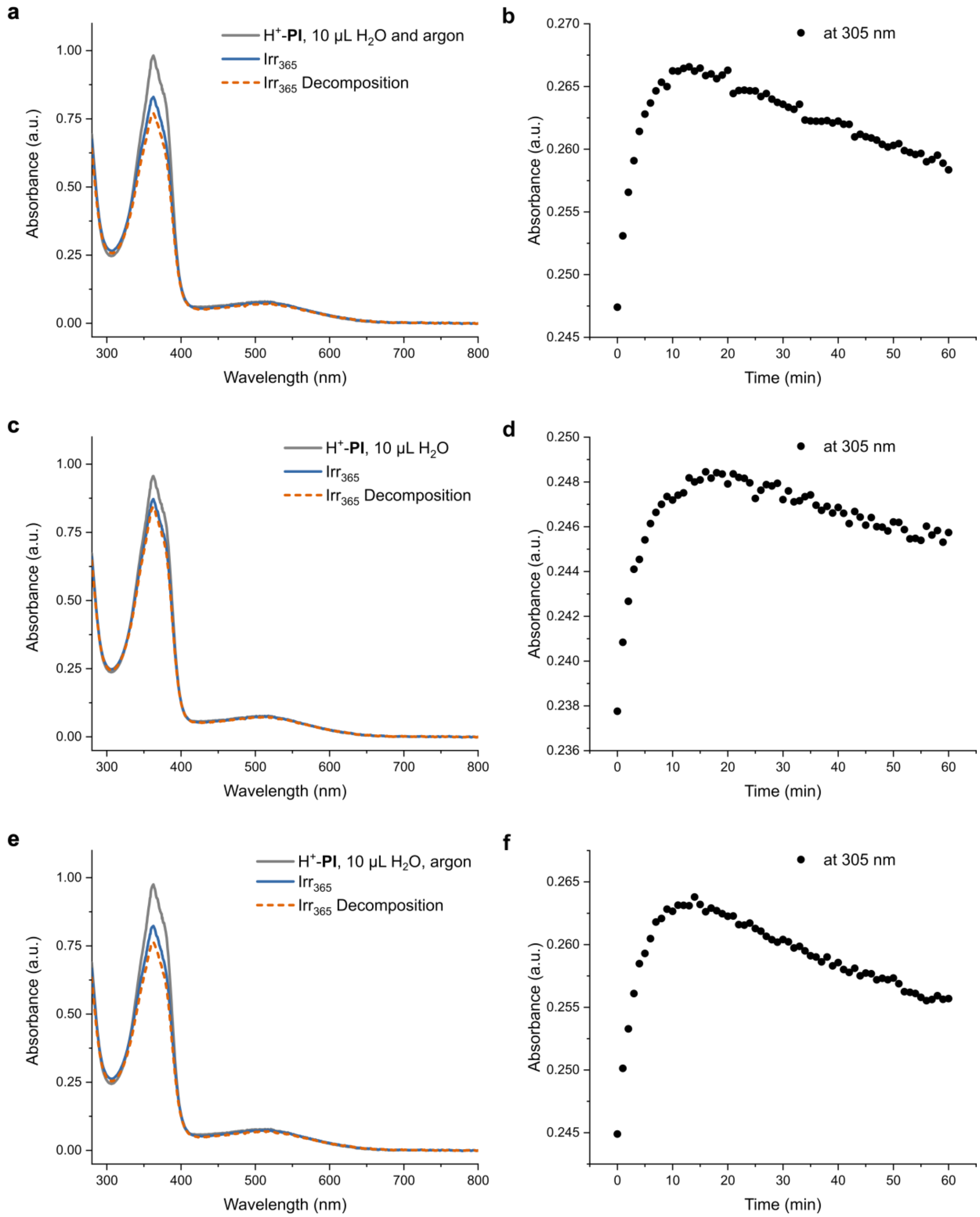


**Figure S77. a** The reached PSS (blue) and degradation (dashed orange) spectra after irradiation of $H^+$-**PI** (4.3x$10^{-2}$ mM) (1.5 eq. *p*TsOH) in MeOH sparged with argon at $\lambda$ = 365 nm. **b** The evolution of the wavelength at $\lambda$ = 305 nm at $Irr_{365}$. **c** The reached PSS (blue) and degradation (dashed orange) spectra after irradiation of $H^+$-**PI** (4.3x$10^{-2}$ mM) (1.5 eq. *p*TsOH and 10 µL $H_2O$) in MeOH at $\lambda$ = 365 nm. **d** The evolution of the wavelength at $\lambda$ = 305 nm at $Irr_{365}$. **e** The reached PSS (blue) and degradation (dashed orange) spectra after irradiation of $H^+$-**PI** (4.3x$10^{-2}$ mM) (1.5 eq. *p*TsOH and 10 µL $H_2O$) in MeOH sparged with argon at $\lambda$ = 365 nm. **f** The evolution of the wavelength at $\lambda$ = 305 nm at $Irr_{365}$.

Comparable behaviour is observed under ambient conditions (see **Figures S74c** and **d**), indicating that $H_2O$ and argon play no role in the stability of **PI** in MeOH.

## 10. Protonation State Studies
### 10.1 UV-Vis Spectra

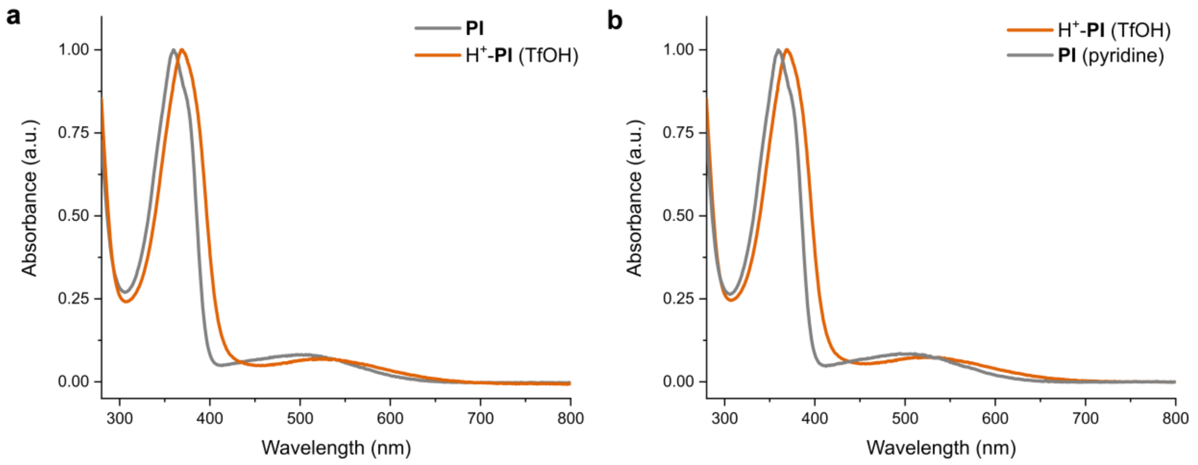


**Figure S78. a** The normalised absorption spectra before (grey) and after (orange) protonation of **PI** with 1.5 eq. triflic acid (TfOH) in spectroscopical grade MeCN. The spectrum of $H^+$-**PI** shows a bathochromic shift of $\Delta\lambda = 9$ nm (360 to 369 nm, respectively). **b** The normalised absorption spectra before (orange) and after (grey) deprotonation of $H^+$-**PI** with pyridine in acetonitrile. A hypsochromic shift of $\Delta\lambda = 9$ nm is observed (369 nm to 360 nm).

## 10.2 FTIR Spectra

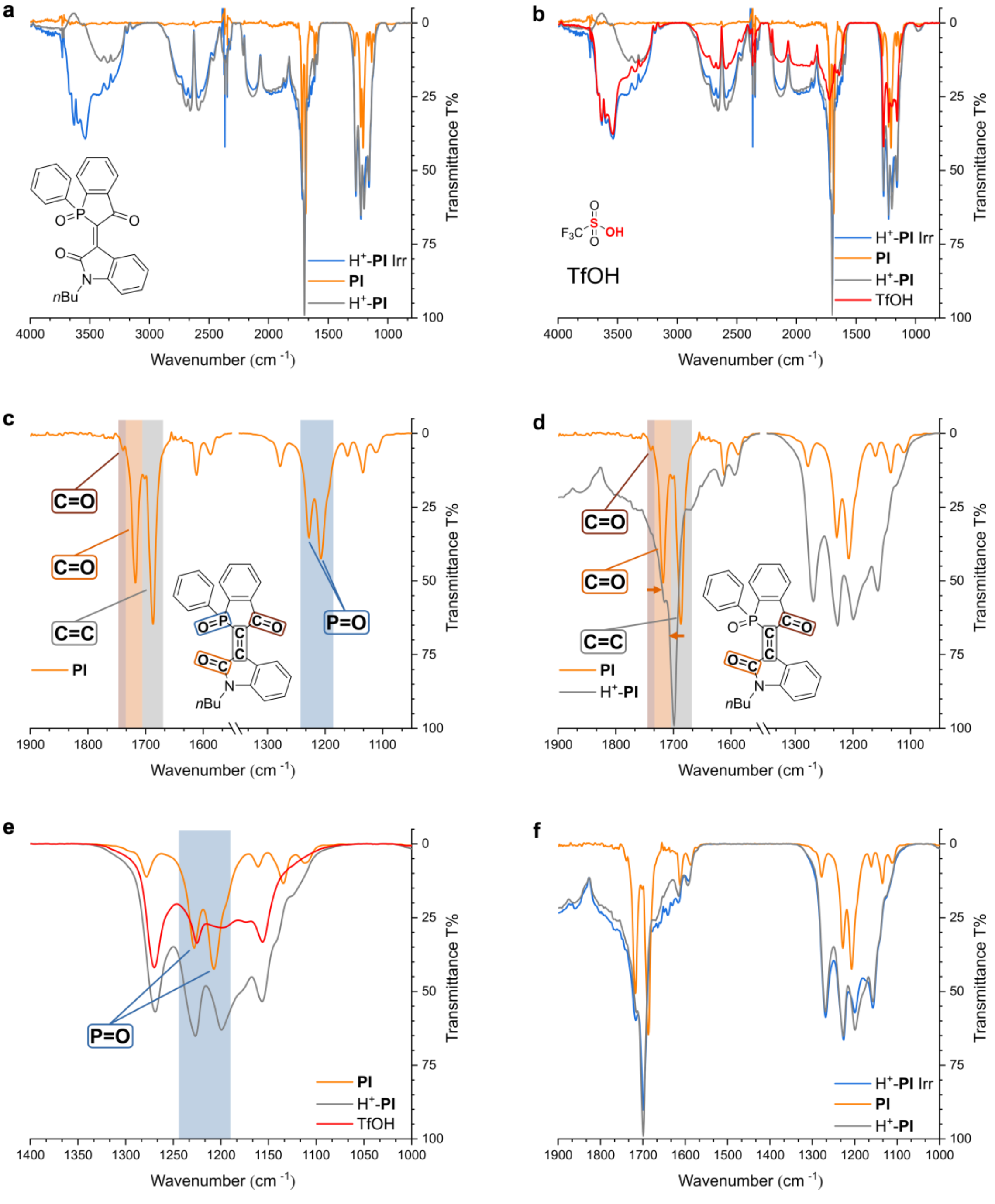


**Figure S79. a** The full FTIR spectra of **PI** (orange), H$^{+}$-**PI** (grey), and irradiated H$^{+}$-**PI** (H$^{+}$-**PI** Irr, blue) in MeCN. Flat areas in the spectra are caused by full absorption by MeCN. The sample was irradiated with a λ = 365 nm LED for 1 hour. **b** The full FTIR spectrum of triflic acid (TfOH, red) overlapped with the other spectra. **c** A zoomed-in FTIR spectrum of **PI** of the region of interest. The indole carbonyl stretch (1739 cm$^{-1}$) is displayed in red, the benzophospholane carbonyl (1717 cm$^{-1}$) in orange, the carbon-carbon double bond (1687 cm$^{-1}$) in grey and the two vibrational modes for the phosphine oxide (1128 and 1208 cm$^{-1}$) in blue, respectively. **d** The overlapped FTIR spectra of **PI** and H$^{+}$-**PI** after the addition of TfOH (1.5 eq.). The benzophospholane carbonyl shifts to a lower wavenumber (1715 cm$^{-1}$), and the C=C double bond shifts to a higher wavenumber (1699 cm$^{-1}$). **e** Overlapped spectra of **PI**, H$^{+}$-**PI** and TfOH; the spectrum of TfOH overlaps with the vibrational modes of the phosphine oxide stretch, obstructing the direct analysis of these modes. **f** The overlapped FTIR spectra of **PI** and (irradiated) H$^{+}$-

**PI**. After 60 minutes of irradiation with a λ = 365 nm LED, no apparent changes were observed in the FTIR spectra.

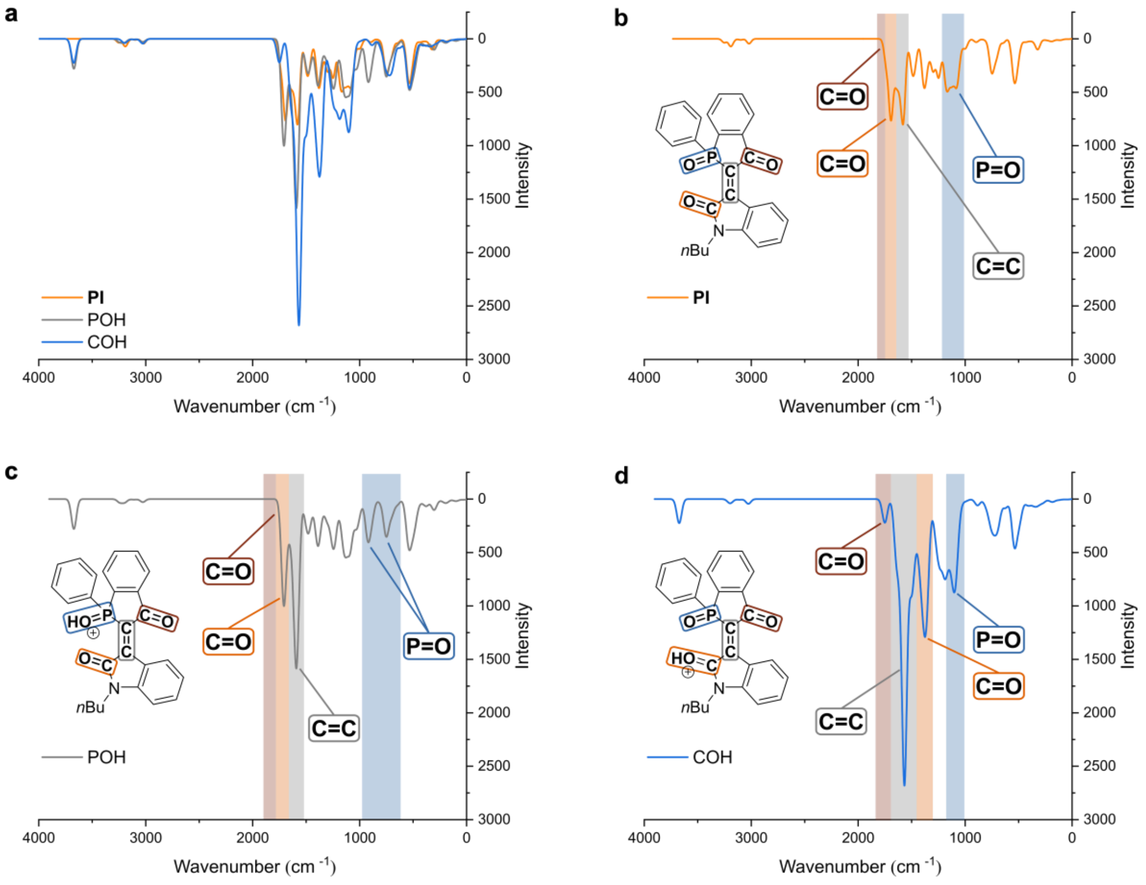


**Figure S80.** Simulated FTIR spectra of *Z*-**PI** (orange), *Z*-**PI** protonated on the phosphine oxide (**POH**, grey), and *Z*-**PI** protonated on the indole carbonyl (**COH**, blue). **a** Full spectra. **b PI**, with highlighted the indole carbonyl stretch (1736 $cm^{-1}$, red) as a weak-intensity shoulder of the stronger-intensity benzophospholane carbonyl stretch (1693 $cm^{-1}$, orange), the carbon-carbon double bond stretch (1577 $cm^{-1}$, grey) and the two-phosphine oxide stretches (1183 and 1174 $cm^{-1}$, blue). **c POH**, with highlighted the frequencies when the phosphine oxide is protonated; the indole carbonyl stretch shifts to lower wavenumber (1724 $cm^{-1}$, red) and due to Gaussian broadening the shoulder resolves, the benzophospholane carbonyl and the C=C double bond stretch shift to higher wavenumbers (1708 $cm^{-1}$, orange; 1586 $cm^{-1}$, grey respectively), and the phosphine oxides shift to lower wavenumbers (925 and 893 $cm^{-1}$, blue). **d COH**, with highlighted the frequencies when the indole carbonyl is protonated; the indole carbonyl stretch shifts to higher wavenumber (1750 $cm^{-1}$, red), the benzophospholane carbonyl and the C=C double bond stretch shift to lower wavenumbers (1510 $cm^{-1}$, blue; and 1566 $cm^{-1}$ grey, respectively), and the phosphine oxides shift to higher wavenumber (1192 $cm^{-1}$).

**Table 3.** Computationally generated FTIR frequencies of **PI**, **POH** and **COH**.

| | **PI** ($cm^{-1}$) | **POH** ($cm^{-1}$) | **COH** ($cm^{-1}$) |
|---|---|---|---|
| Indole-C=O stretch | 1736 | 1724 | 1750 |
| Benzophospholane-C=O stretch | 1693 | 1708 | 1510 |
| C=C stretch | 1577 | 1586 | 1566 |
| P=O stretch | 1183 | 925 | 1192 |
| P=O stretch | 1174 | 893 | - |
| OH wag 1 | - | 1088 | 1247 |
| OH wag 2 | - | 1031 | 1230 |
| OH stretch | - | 3672 | 3673 |

The computational data show strong differences in FTIR spectra between protonation on the phosphine oxide (i.e. **POH**) and protonation on the indole carbonyl (i.e. **COH**). The experimentally measured FTIR spectra show that some regions are unable to be analysed due to the full absorption of MeCN. The phosphine oxide stretches cannot be analysed due to the full overlap by the spectrum of TfOH and the appearance of their bands in the full absorption region for **POH**. The strongest indication is the calculated shift to 1510 $cm^{-1}$ for the benzophospholane carbonyl stretch in **COH**; experimentally, we do not observe the disappearance of this band after addition of acid (see **Figure S79**). In combination with the observed shift of the C=C stretch to higher wavenumbers, we conclude that protonation happens on the P=O.

## 10.3 NMR Studies

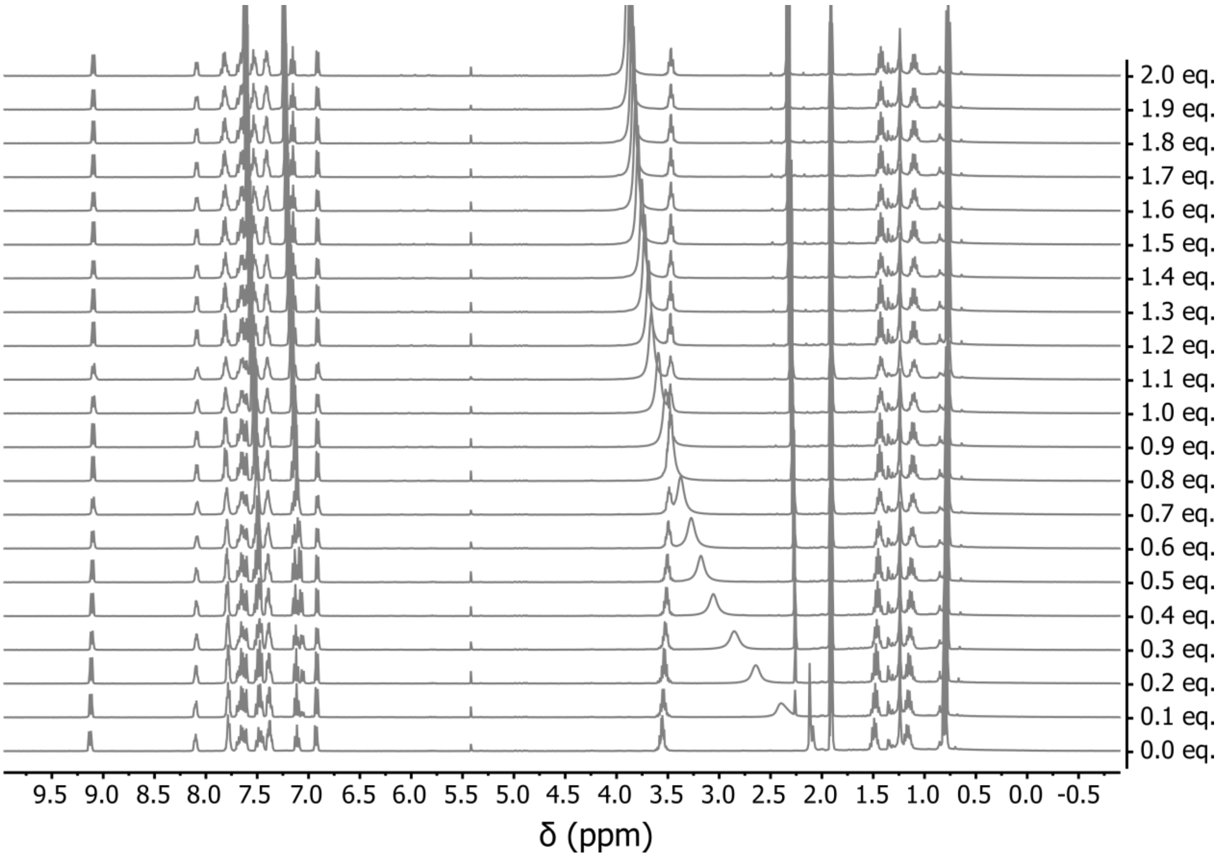


**Figure S81.** The full $^{1}$H-NMR spectra of the titration of **PI** with *p*-toluenesulfonic acid in MeCN-d$_3$.

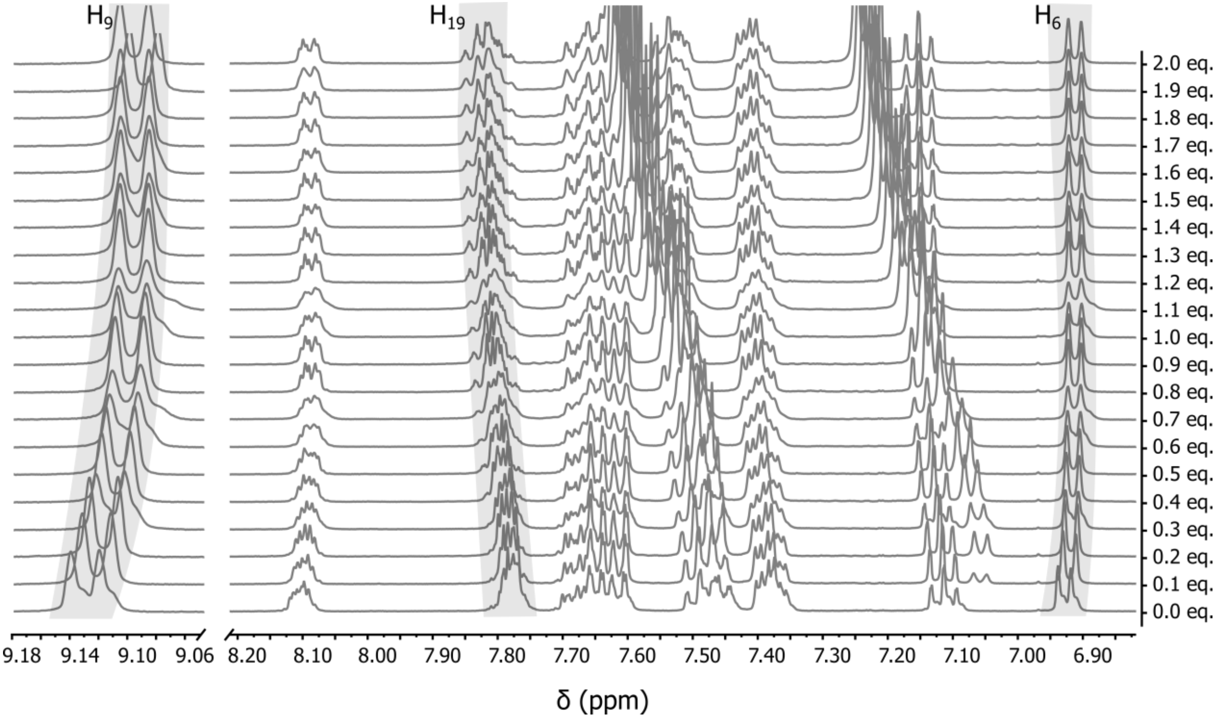


**Figure S82.** Zoomed-in $^{1}$H-NMR spectra of the titration of **PI** with *p*-toluenesulfonic acid in MeCN-d$_3$; protonation of **PI** results in a shielding effect on H$_6$ and H$_9$, whereas H$_{19}$ is slightly deshielded.

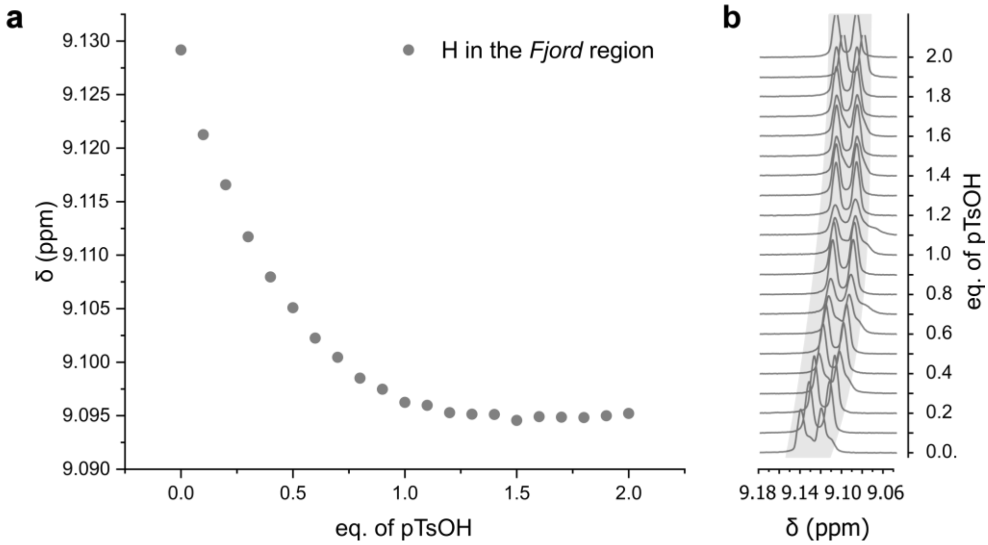


**Figure S83.** Zoomed-in $^{1}$H-NMR spectra of $H_9$ of the titration of **PI** with *p*-toluenesulfonic acid (*p*TsOH) in MeCN-$d_3$; accompanied by the Jobs plot of *p*TsOH versus the chemical shift of $H_9$, where it shows that after the addition of 1.5 eq. of *p*TsOH, there is no further effect on **PI**.

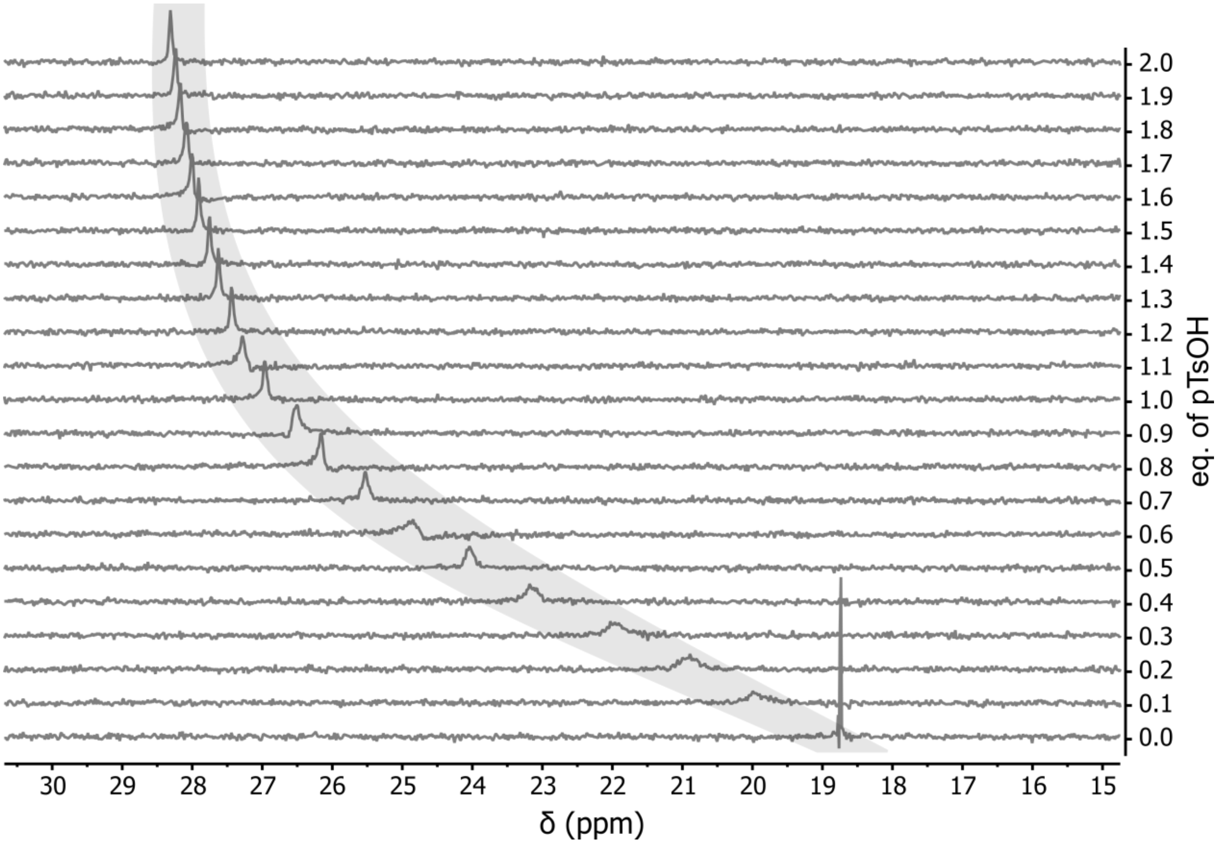


**Figure S84.** The full $^{31}$P-NMR spectra of the titration of **PI** with *p*-toluenesulfonic acid in MeCN-$d_3$; protonation of **PI** results in a deshielding effect on the phosphorus, which is directly related to protonation on the phosphine oxide. Broadening of the peak between 0.1 eq. and 0.6 eq. of *p*TsOH indicates an equilibrium between protonation on the phosphine oxide and its surroundings.

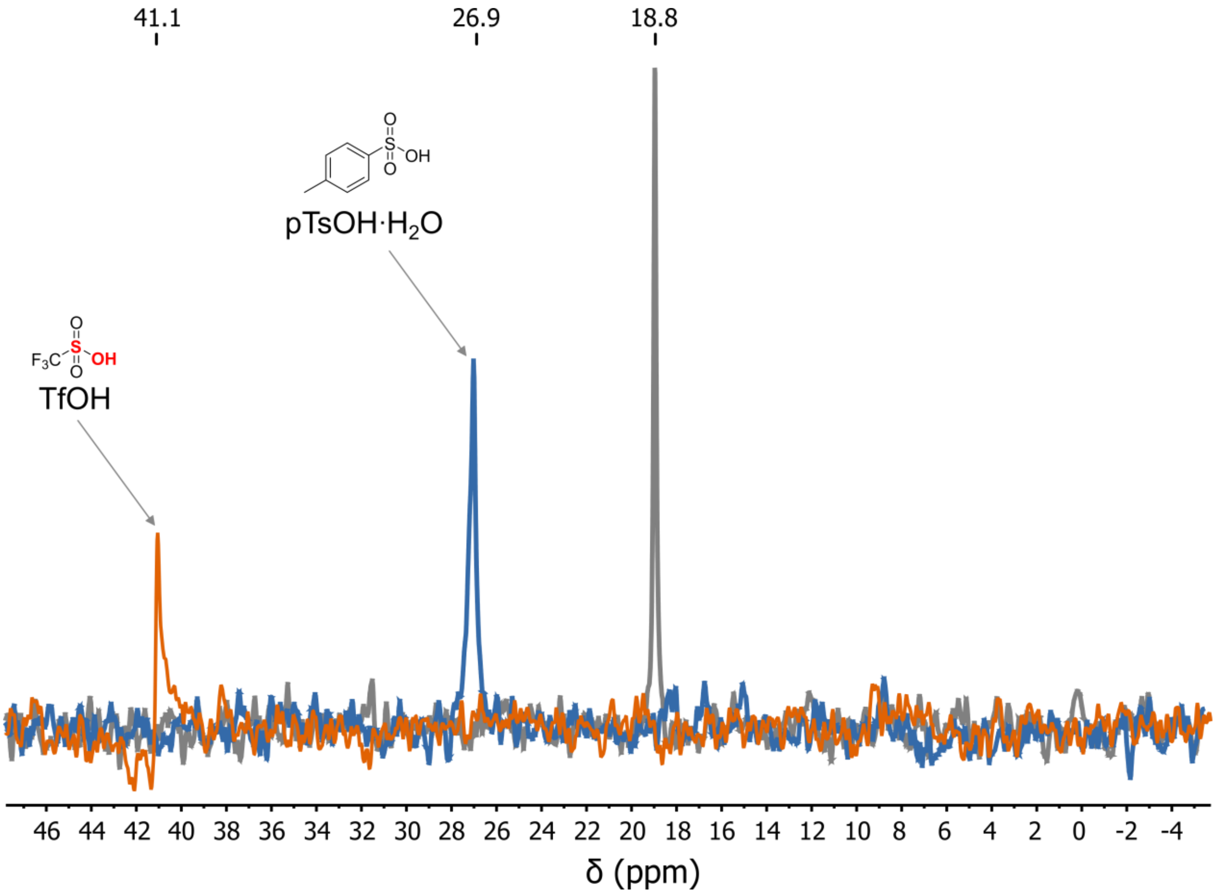


**Figure S85.** The $^{31}$P-NMR spectra in anhydrous MeCN-$d_3$ of **PI** (grey) and protonated-**PI** with 1.5 eq. of *p*-toluenesulfonic acid (*p*TsOH, blue) and protonated-**PI** with 1.5 eq. of triflic acid (orange, TfOH) show that protonation by TfOH results in a more deshielded phosphorus atom.

The pKa values in MeCN of *p*TsOH (8.5)[50] and TfOH (0.70)[51] suggest the extent of protonation of **PI** in MeCN by TfOH is greater than by *p*TsOH. The pKa value of triphenylphosphine oxide (TPPO) is 2.80 (in nitromethane),[52] This value is the closest approximation we have to the pKa of **PI**, and this suggests that the conjugated acid of **PI** is stronger than *p*TsOH or of similar strength. Hence, in anhydrous acetonitrile and the presence of a strong acid, the phosphine oxide of **PI** undergoes complete covalent protonation at the phosphoryl oxygen, forming a well-defined O-H bond. Under these conditions, the proton remains localized on the oxygen due to the lack of competing hydrogen-bonding interactions. However, upon introduction of water, solvation of the acidic proton occurs, leading to partial protonation and increased proton lability. This enhanced dynamic exchange with solvent reduces the strength and persistence of the O-H interaction, resulting in diminished photoreactivity and favoring non-destructive, reversible processes or rearrangements upon irradiation.

## 11. NMR Irradiation

### 11.1 THF

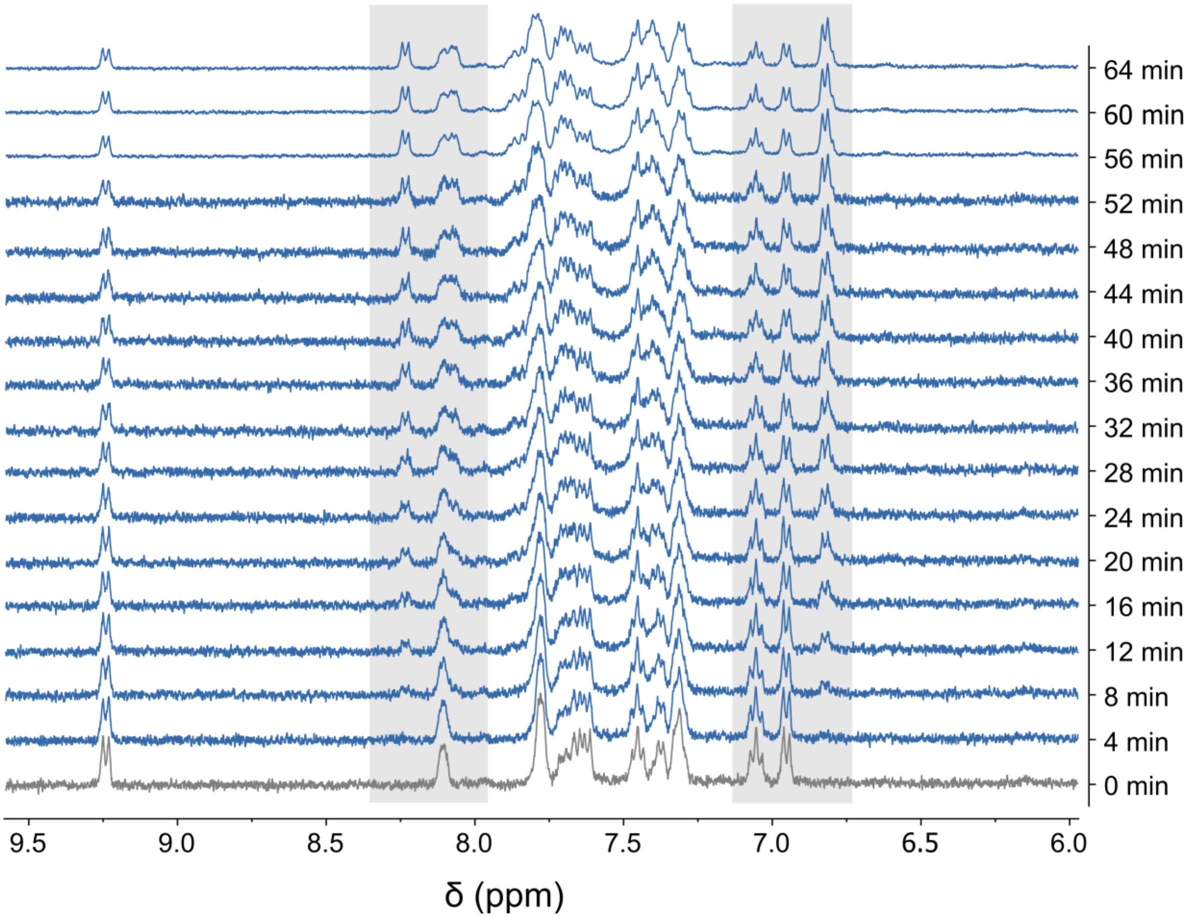


**Figure S86.** The $^{1}$H-NMR spectra of the *in-situ* NMR irradiation of (2 mM) **PI** in THF-$d_8$ with $\lambda$ = 395 nm. After 64 minutes, a PSS is reached of 51% stable and 49% metastable **PI**.

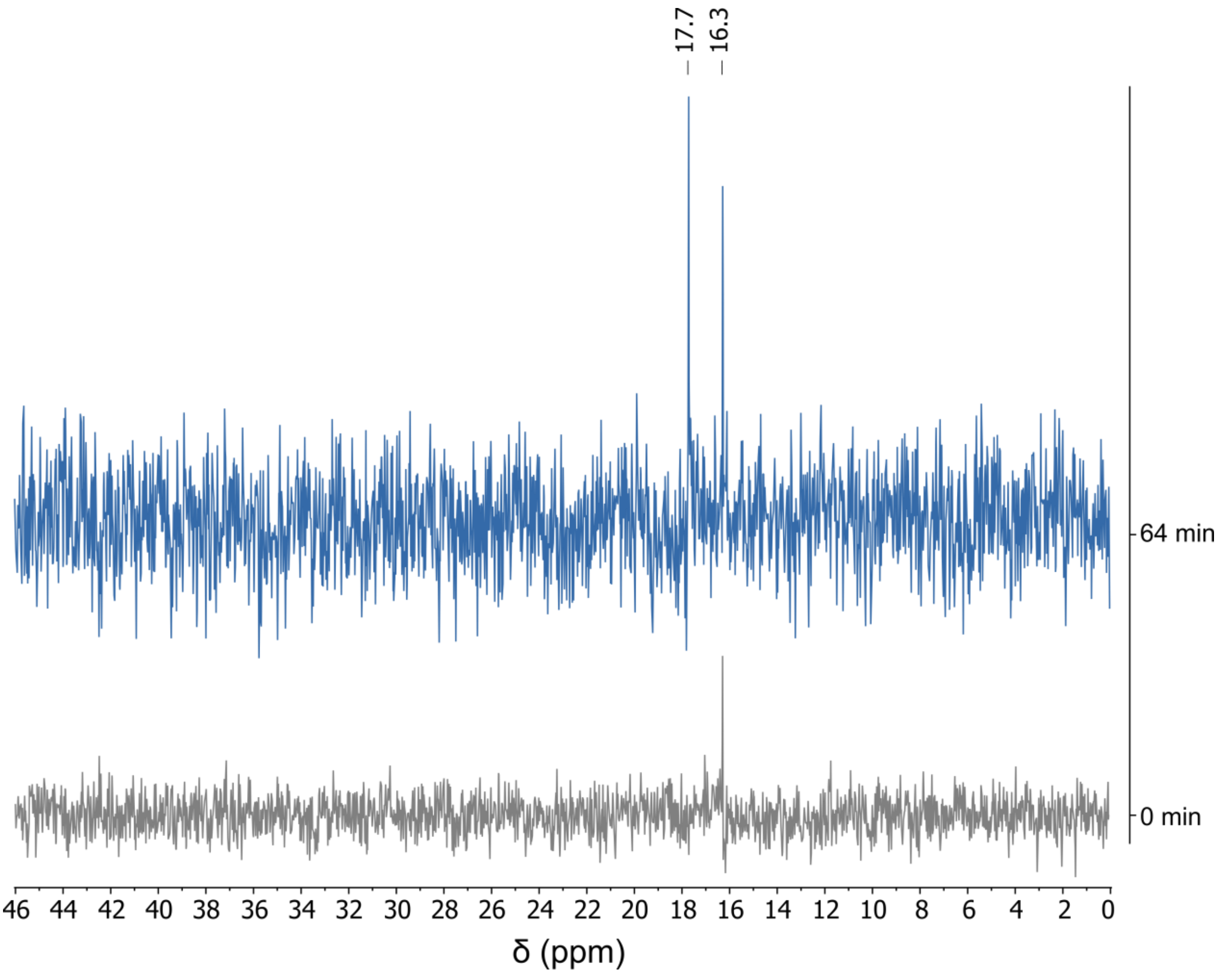


**Figure S87.** The $^{31}$P-NMR spectra of the *in-situ* NMR irradiation of **PI** in THF-$d_8$ with λ = 365 nm. Displaying the stable **PI** ($\delta$ = +16.3 ppm) and metastable **PI** ($\delta$ = +17.7 ppm).

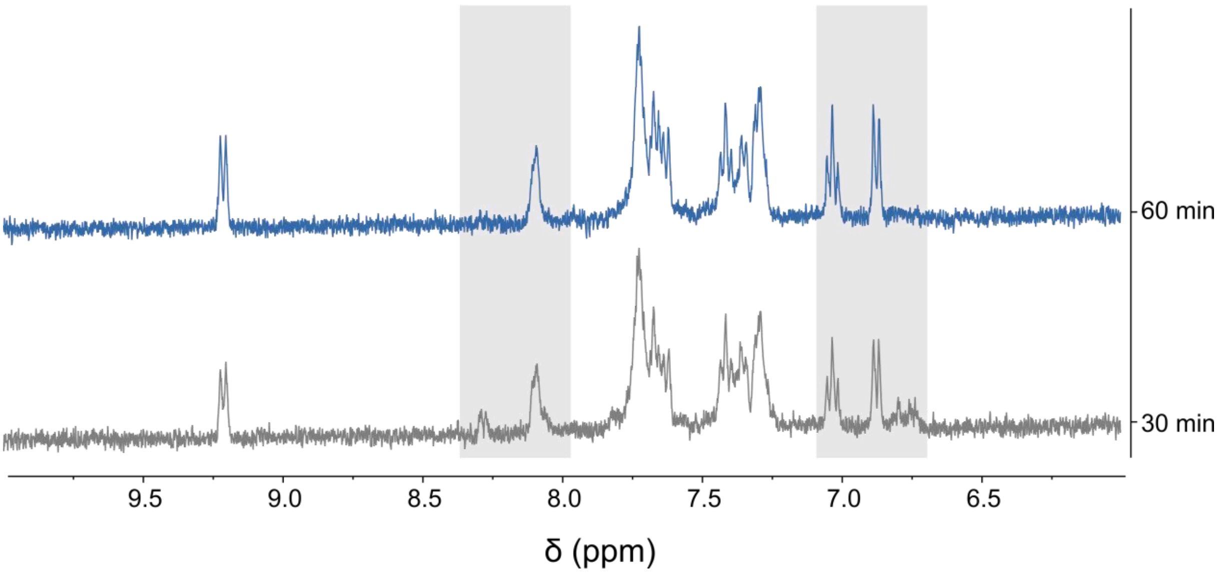


**Figure S88.** The $^{1}$H-NMR spectra of the thermal back-isomerization at 25 °C of **PI** in THF-$d_8$ after irradiation with λ = 395 nm. After 60 minutes, **PI** has fully recovered.

## 11.2 Chloroform

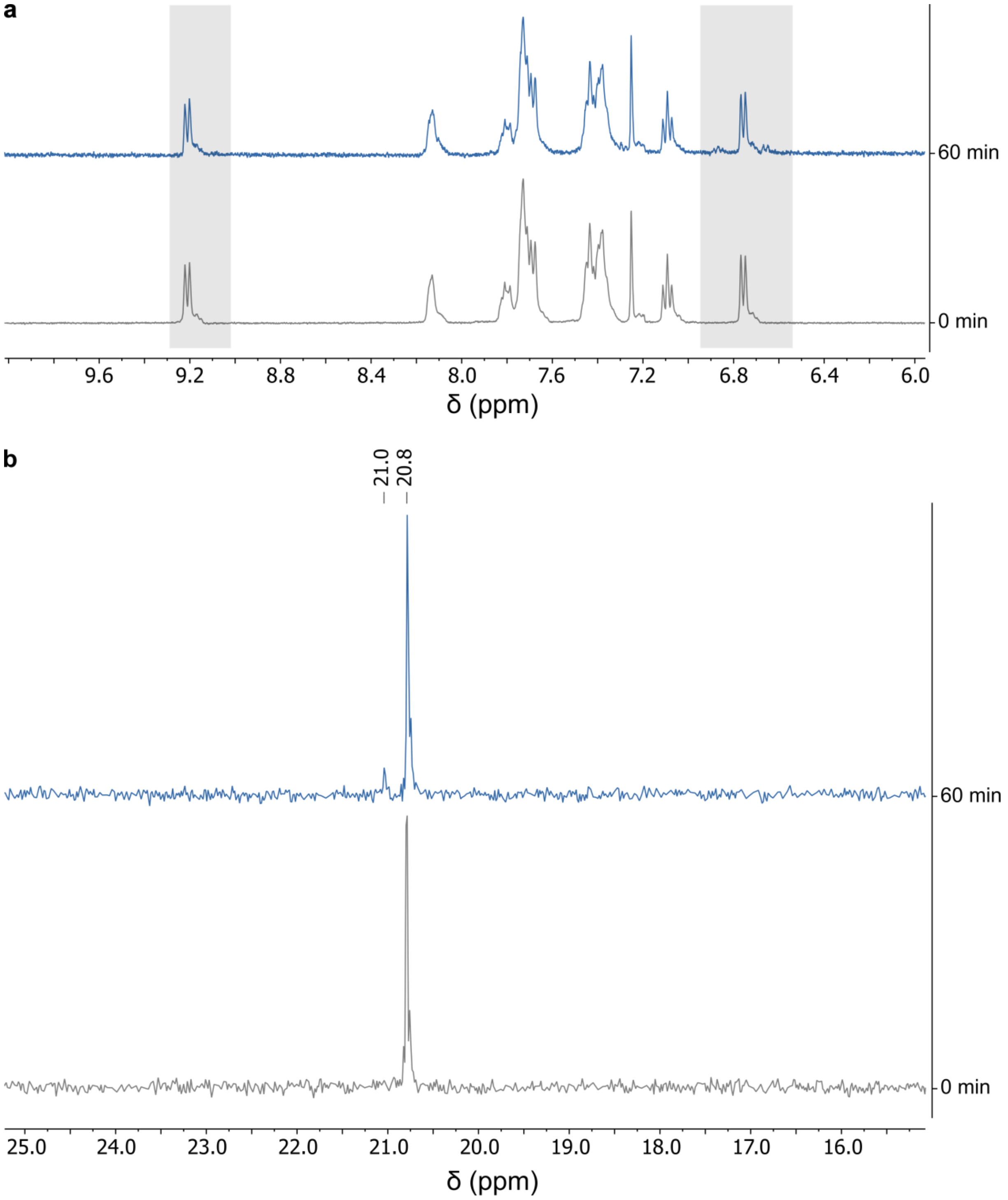


**Figure S89. a** The $^{1}$H-NMR spectra of the *in-situ* NMR irradiation of (14 mM) **PI** in $CDCl_3$ with λ = 365 nm. After 60 minutes, minimal switching was observed. **b** The $^{31}$P-NMR spectra of the in-situ NMR irradiation of **PI** in $CDCl_3$ with λ = 365 nm. Displaying the stable **PI** ($\delta$ = +20.8 ppm) and metastable **PI** ($\delta$ = +21.0 ppm).

### 11.3 Acetonitrile

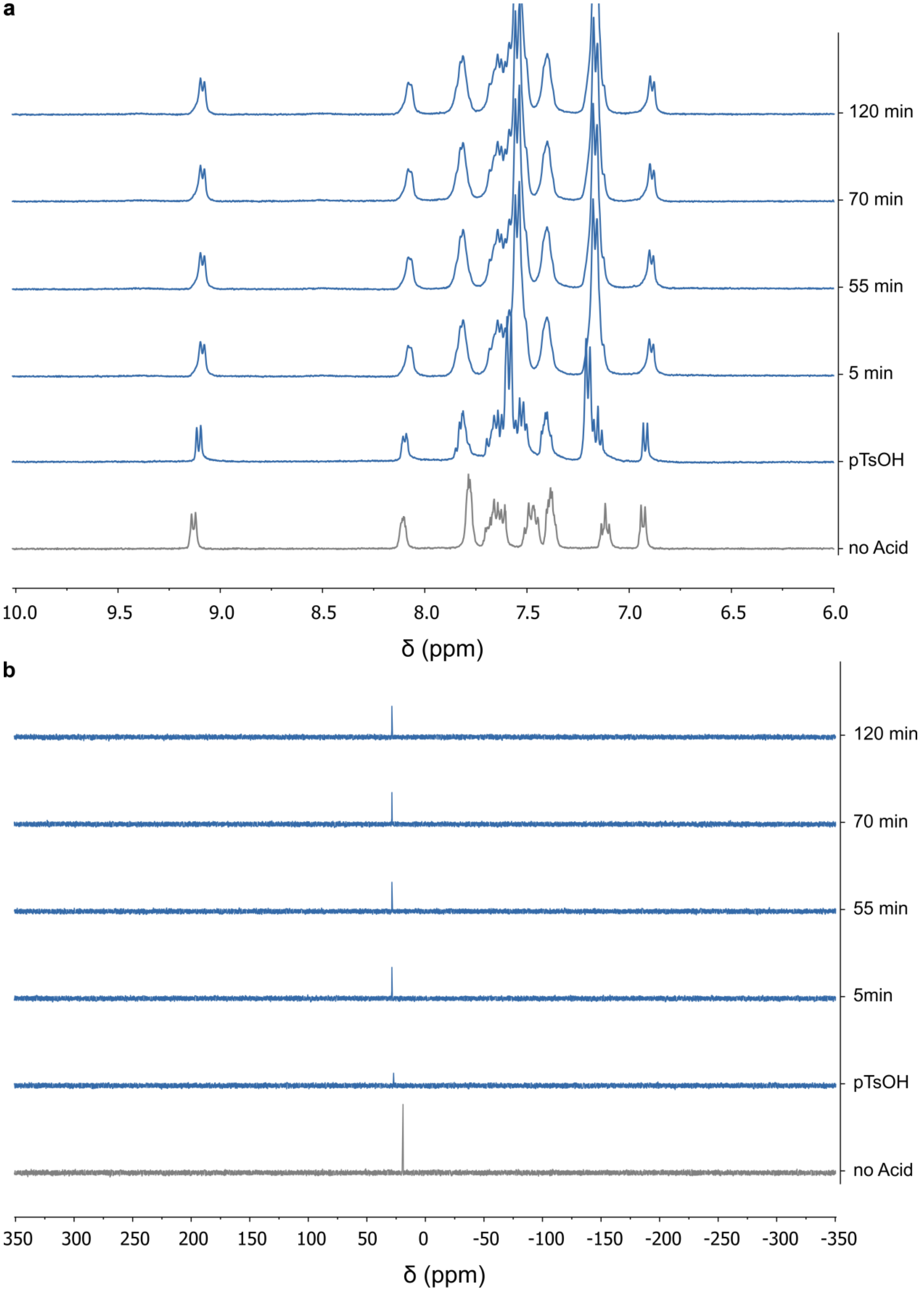


**Figure S90.** *In-situ* NMR irradiation of protonated **PI** (7 mM, 1.5 eq. *p*TsOH) in MeCN-$d_3$ with λ = 365 nm. **a** $^{1}$H-NMR spectra. **b** $^{31}$P-NMR spectra. After 120 minutes, no switching was observed.

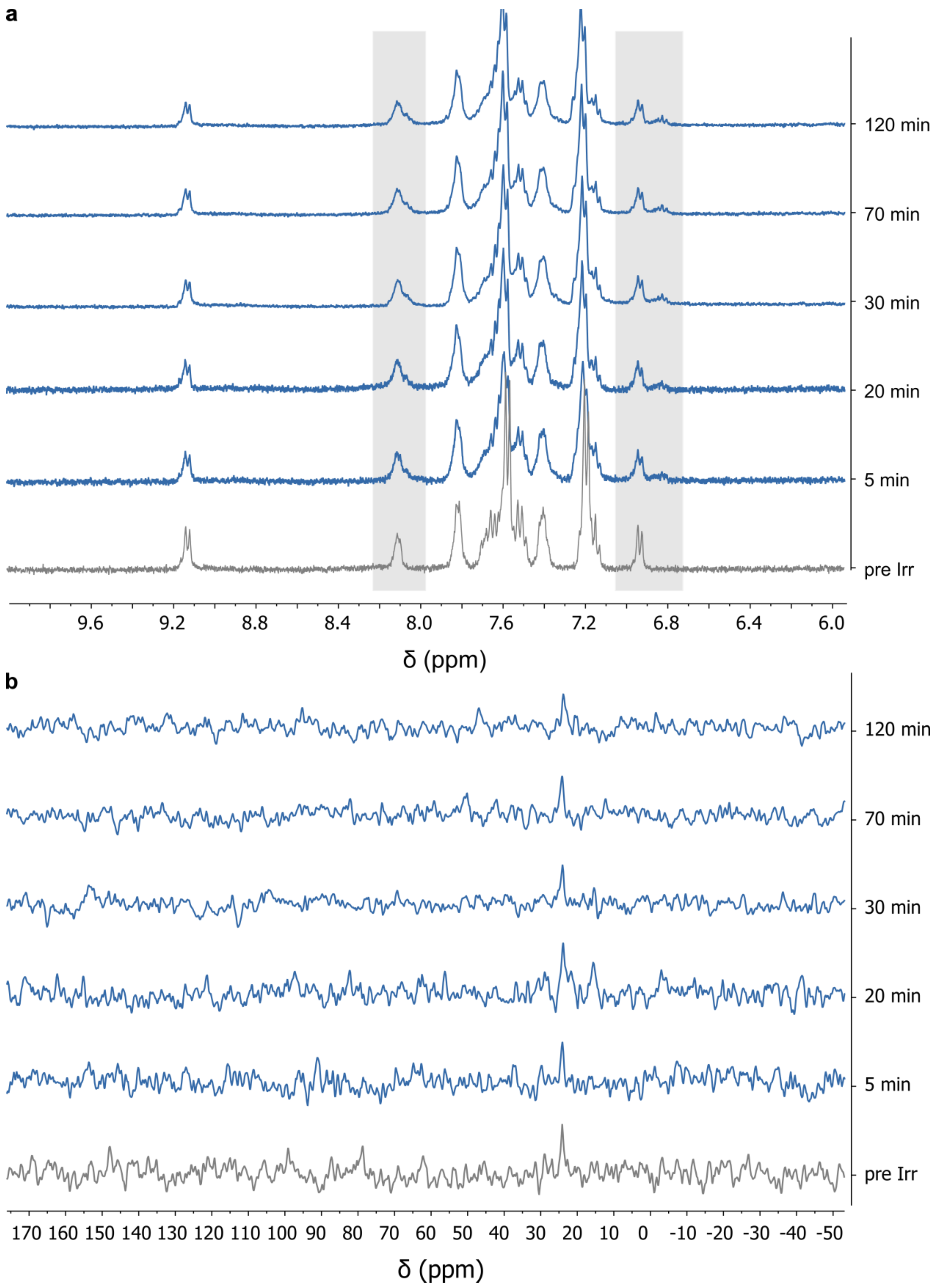


**Figure S91. a** The $^{1}$H-NMR spectra of the *in-situ* NMR irradiation at -25 °C of protonated (1.5 eq. *p*TsOH) **PI** (3 mM) in MeCN-$d_3$ with $\lambda = 365$ nm. After 120 minutes, minimal switching was observed. **b** The $^{31}$P-NMR spectra of **a**. After 120 minutes, only the stable **PI** ($\delta$ = +23.4 ppm) was observed.

## 12. LCMS-data

### 12.1 THF

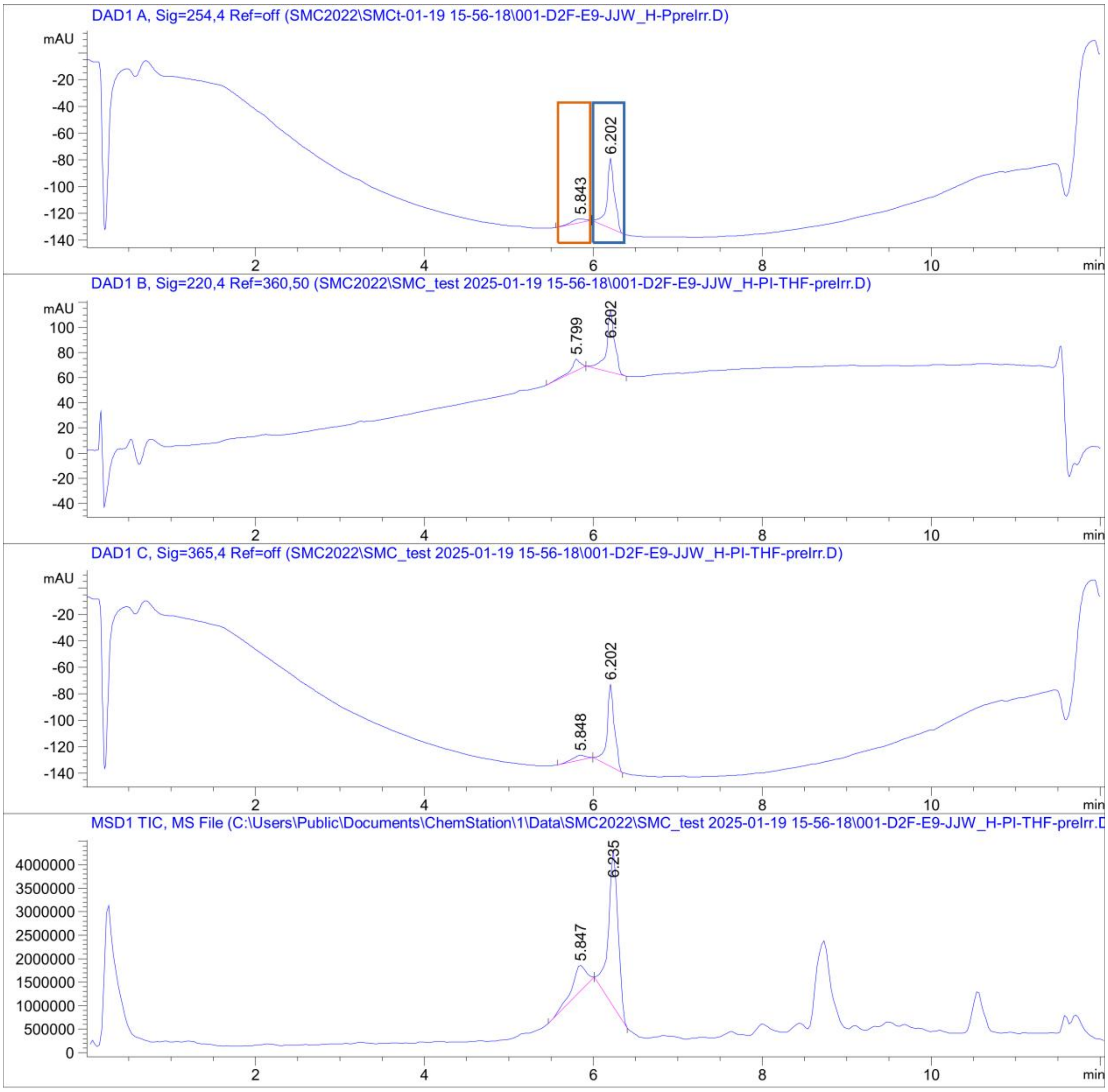


**Figure S92.** The LC-MS spectra of **PI** in THF before irradiation. The DAD spectra show two peaks belonging to the stable *Z*-isomer (blue, 6.2 min) and metastable *E*-isomer (orange, 5.8 min) of **PI**. (For the determination of the retention time of the stable and metastable isomer, refer to **Section 12.2.1**).

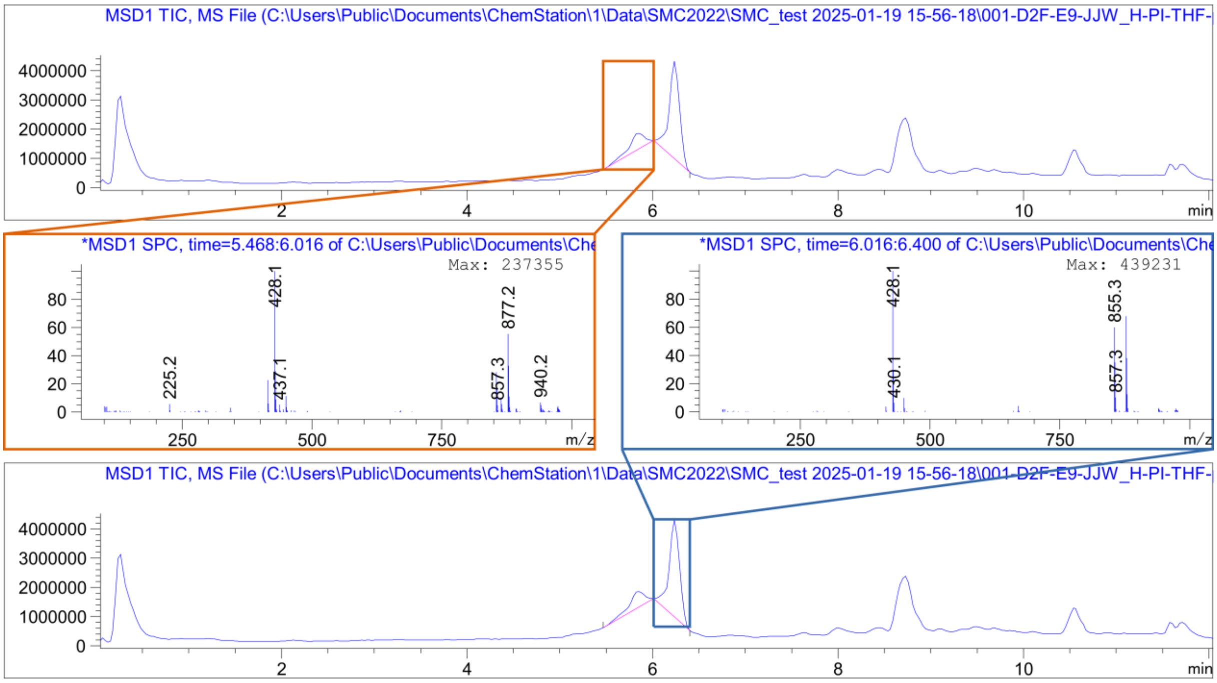


**Figure S93.** The LC-MS spectra of **PI** in THF before irradiation. The MS spectra show the mass for [**PI**+H]$^+$ (428.1), indicating the presence of both isomers before irradiation in THF.

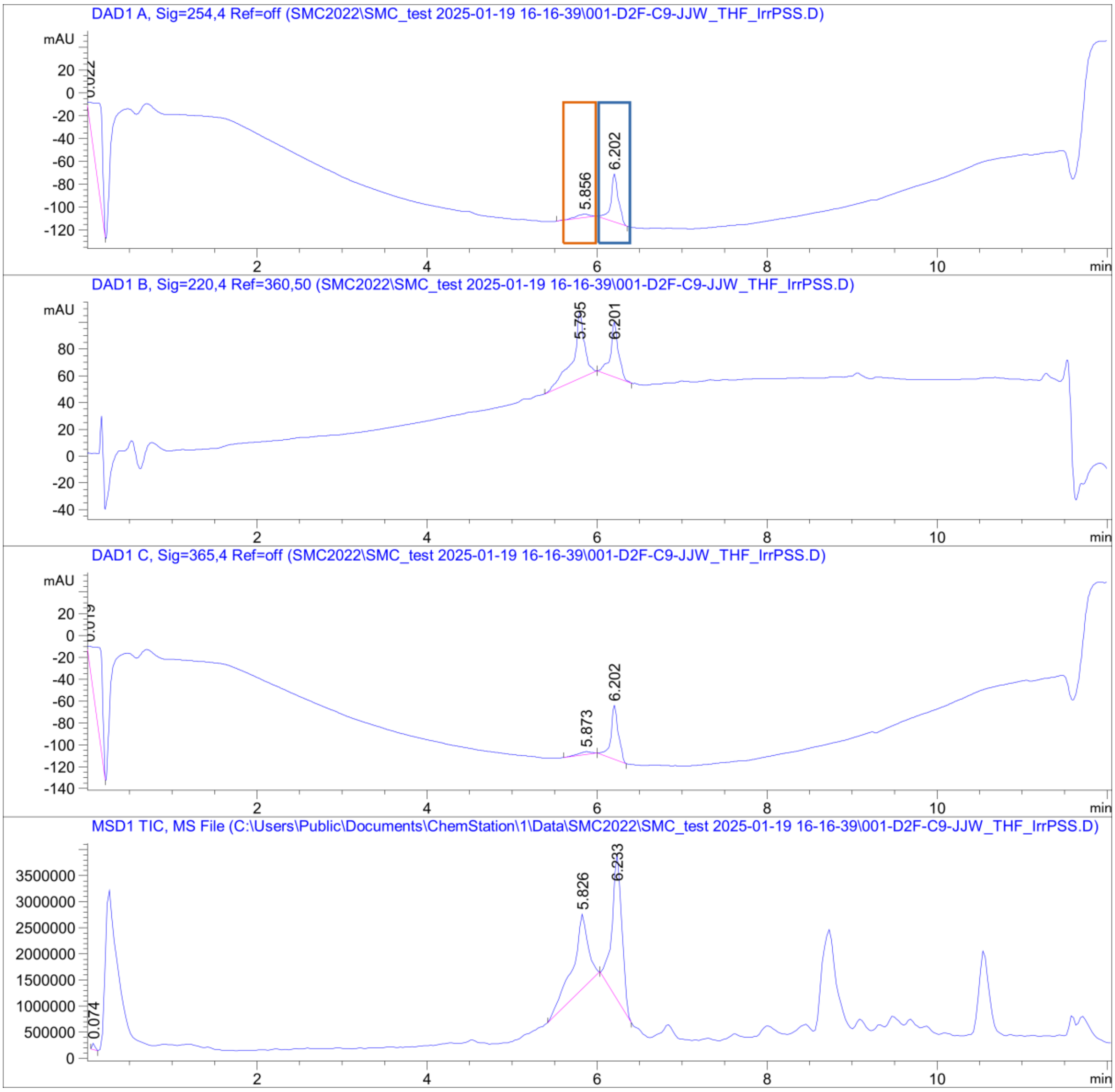


**Figure S94.** The LC-MS spectra of **PI** in THF after irradiation at λ = 395 nm. The DAD spectra show two peaks belonging to the stable (blue, 6.2 min) and metastable (orange, 5.8 min) isomers of **PI**. The MS spectrum shows a larger relative intensity between the two isomers than before switching, indicating a larger contribution of the metastable isomer after irradiation. No peaks for decomposition products were observed.

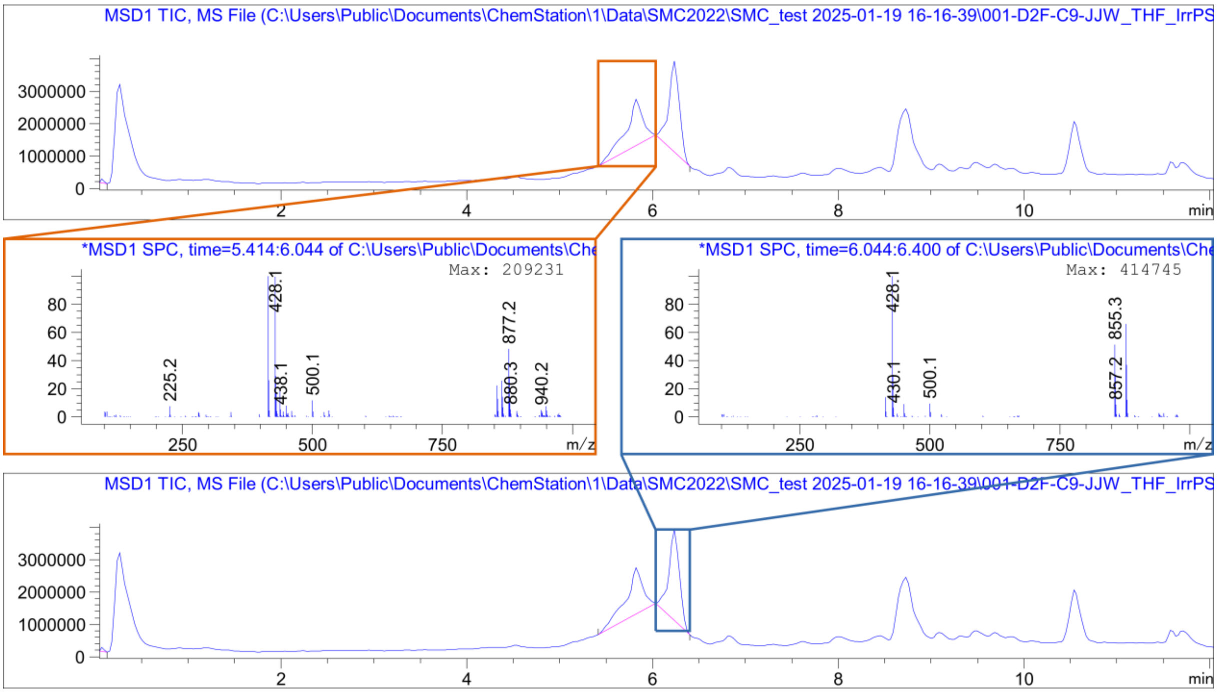


**Figure S95.** The LC-MS spectra of **PI** in THF after irradiation. The MS spectra show the mass for [**PI**+H]$^+$ (428.1), indicating the presence of both isomers.

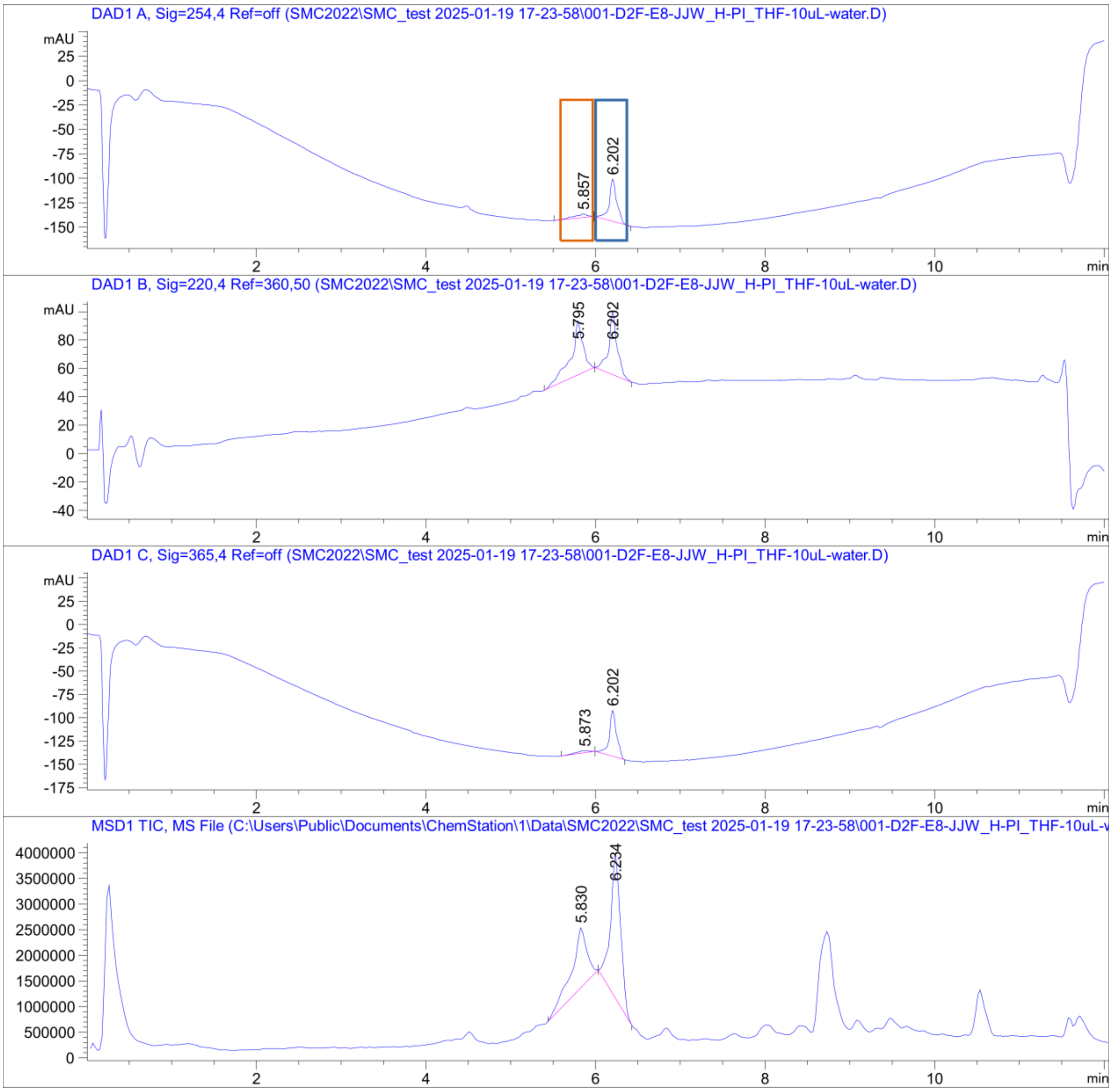


**Figure S96.** The LC-MS spectra of **PI** in THF (with 10 µL of $H_2O$) after irradiation at λ = 395 nm. The DAD spectra show two peaks belonging to the stable (blue, 6.2 min) and metastable (orange, 5.8 min) isomers of **PI**. The MS spectrum shows a bigger relative intensity between the two isomers than before switching, indicating a larger contribution of the metastable isomer after irradiation. No peaks for decomposition products were observed.

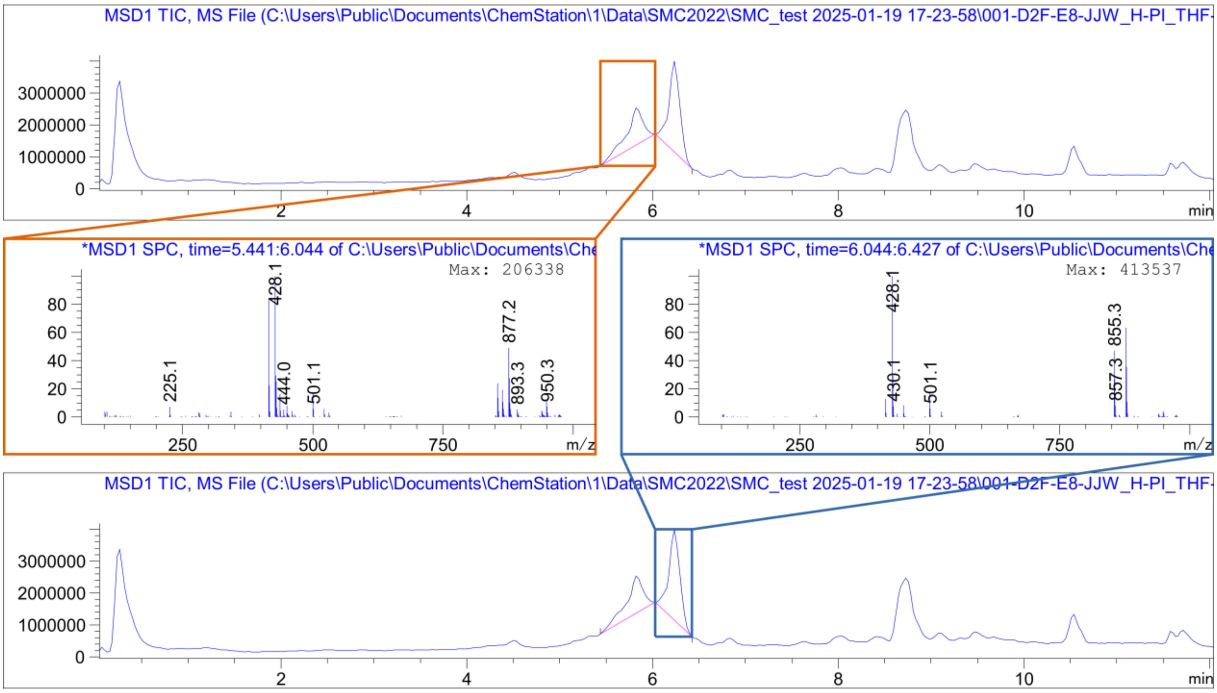


**Figure S97.** The LC-MS spectra of **PI** in THF after irradiation. The MS spectra show the mass for [**PI**+H]$^+$ (428.1), indicating the presence of both isomers.

## 12.2 Acetonitrile

### 12.2.1 The Determination of the Stable and Metastable Isomer in LCMS

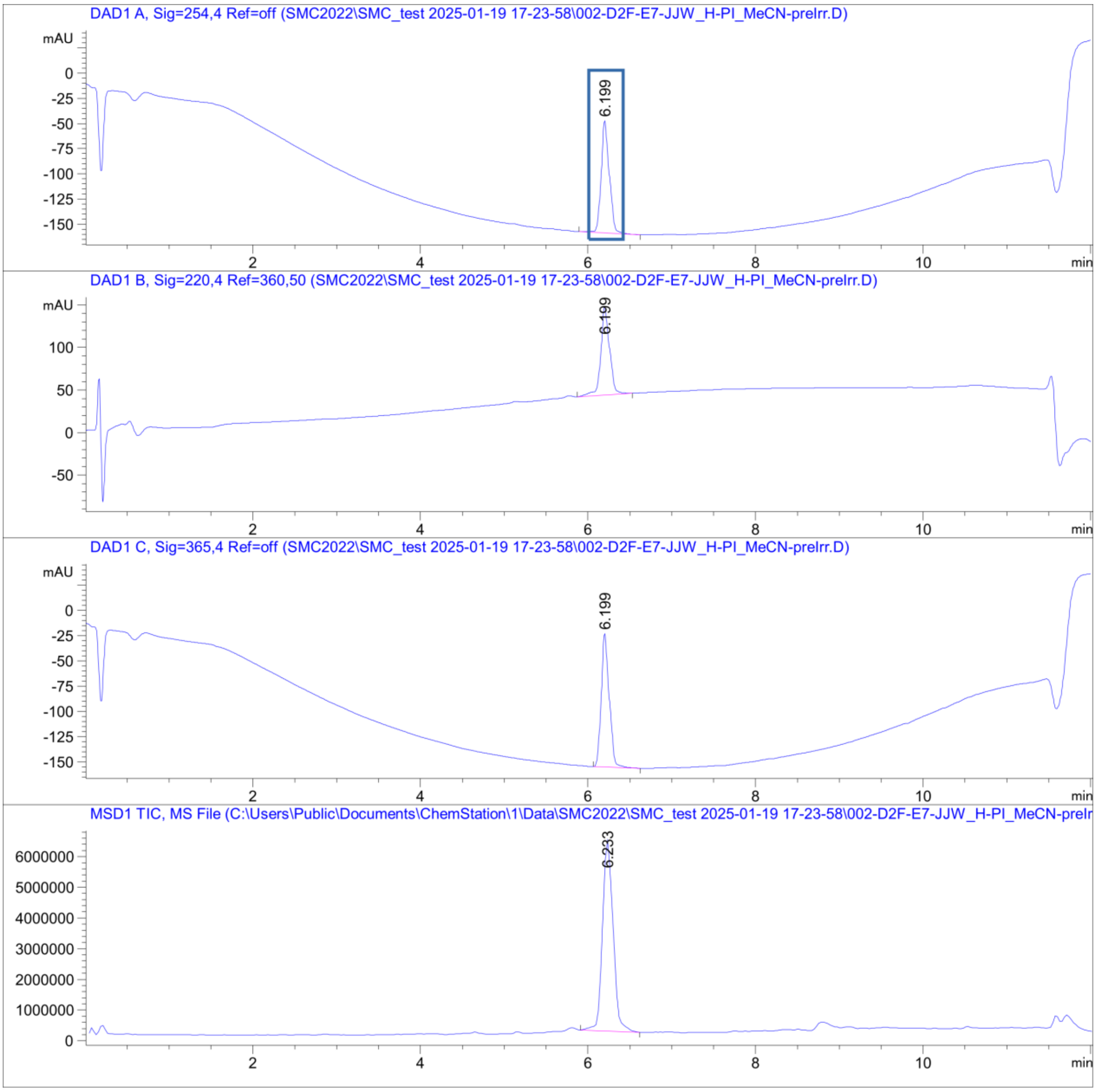


**Figure S98.** The LC-MS spectra of **PI** in MeCN (with 1.5 eq. of *p*TsOH) before irradiation. The DAD spectra show one peak belonging to the stable isomer (blue, 6.2 min).

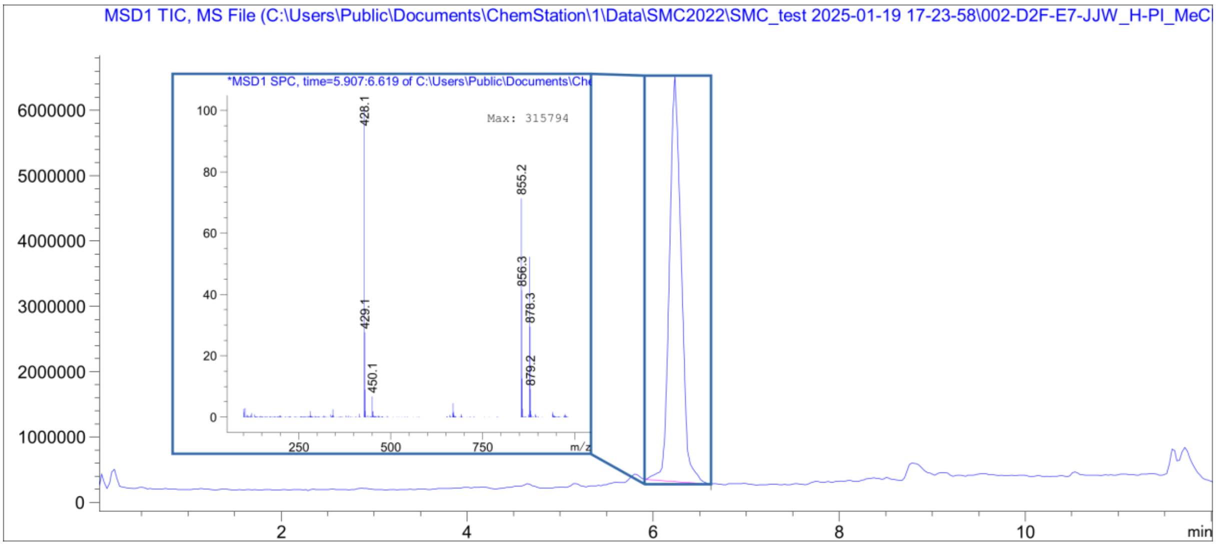


**Figure S99.** The LC-MS spectra of **PI** in MeCN (with 1.5 eq. of *p*TsOH) before irradiation. The MS spectra show the mass for [**PI**+H]$^+$ (428.1).

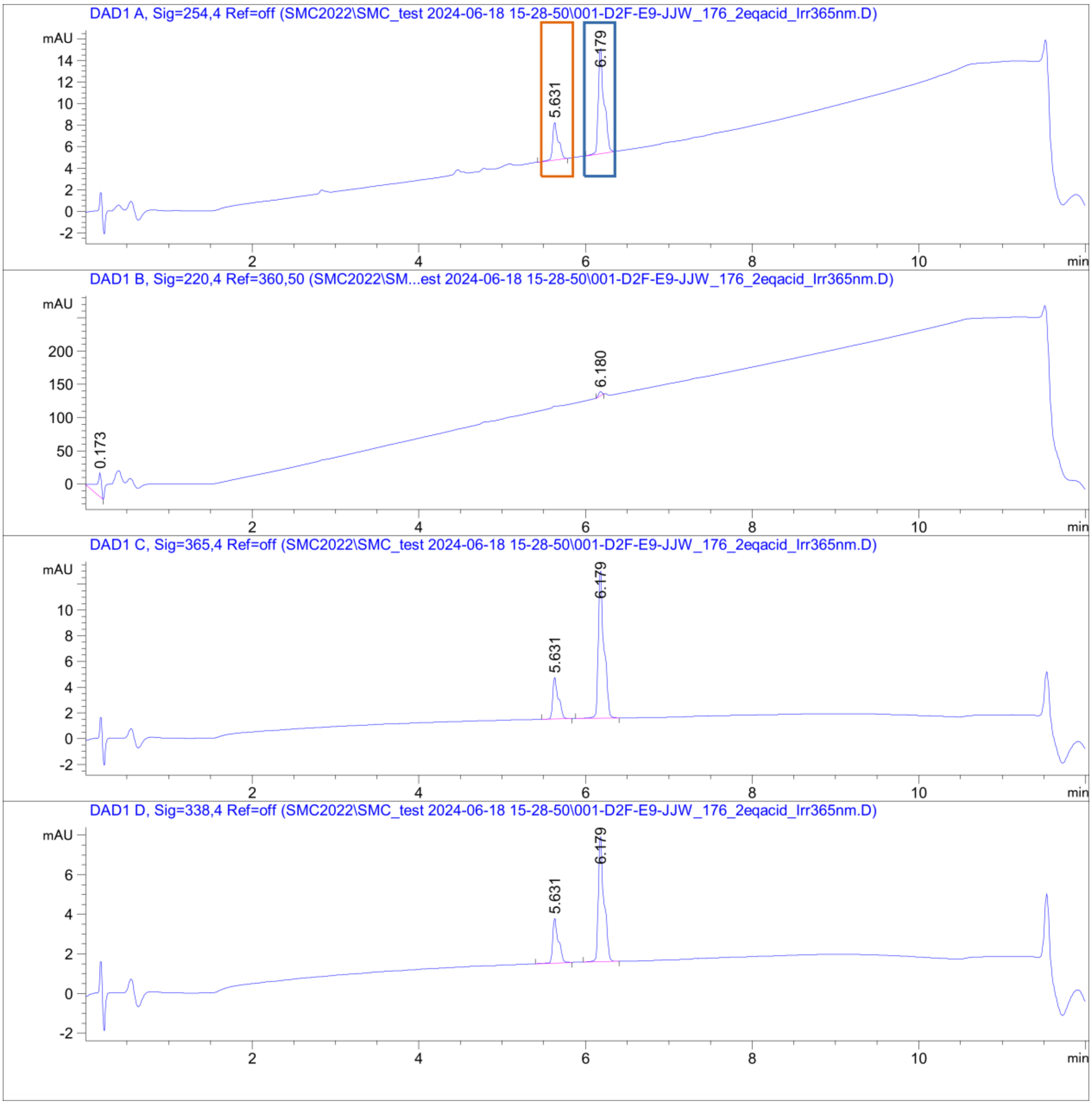


**Figure S100.** The LC-MS spectra of **PI** in MeCN (with 1.5 eq. of *p*TsOH) after irradiation at $\lambda = 365$ nm. The DAD spectra show two peaks belonging to the stable (blue, 6.2 min) and metastable (orange, 5.8 min) isomers of **PI**.

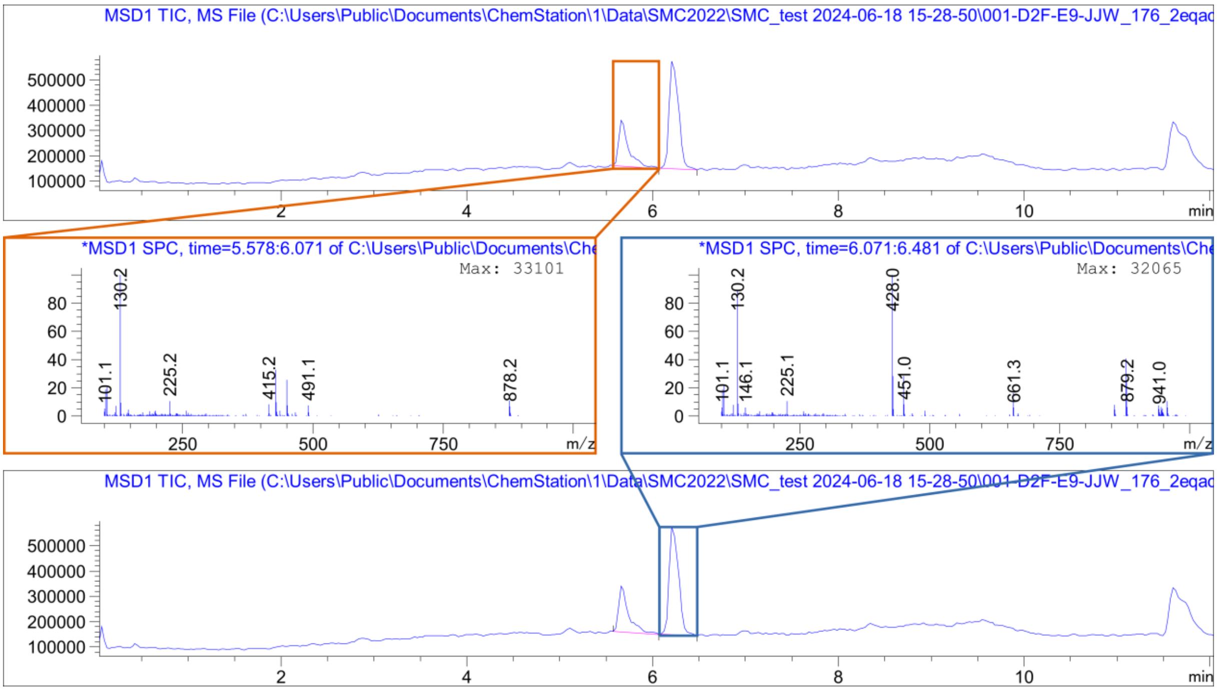


**Figure S101.** The LC-MS spectra of **PI** in MeCN (with 1.5 eq. of *p*TsOH) after irradiation at $\lambda = 365$ nm. The MS spectra show the mass for [**PI**+H]+ (428.1), indicating the presence of both isomers. Confirming that the retention times belong to the metastable (orange, 5.8 min) and the stable (blue, 6.2 min) isomer of **PI**. The LC-MS was monitored at the isosbestic point ($\lambda = 338$ nm), and the area underneath the peaks of the two isomers led to a photostationary distribution of 22% metastable and 78% stable. [(8.38/(8.38+28.95)*100% = 22%].

### 12.2.2 The Difference in PSS Determination in LCMS

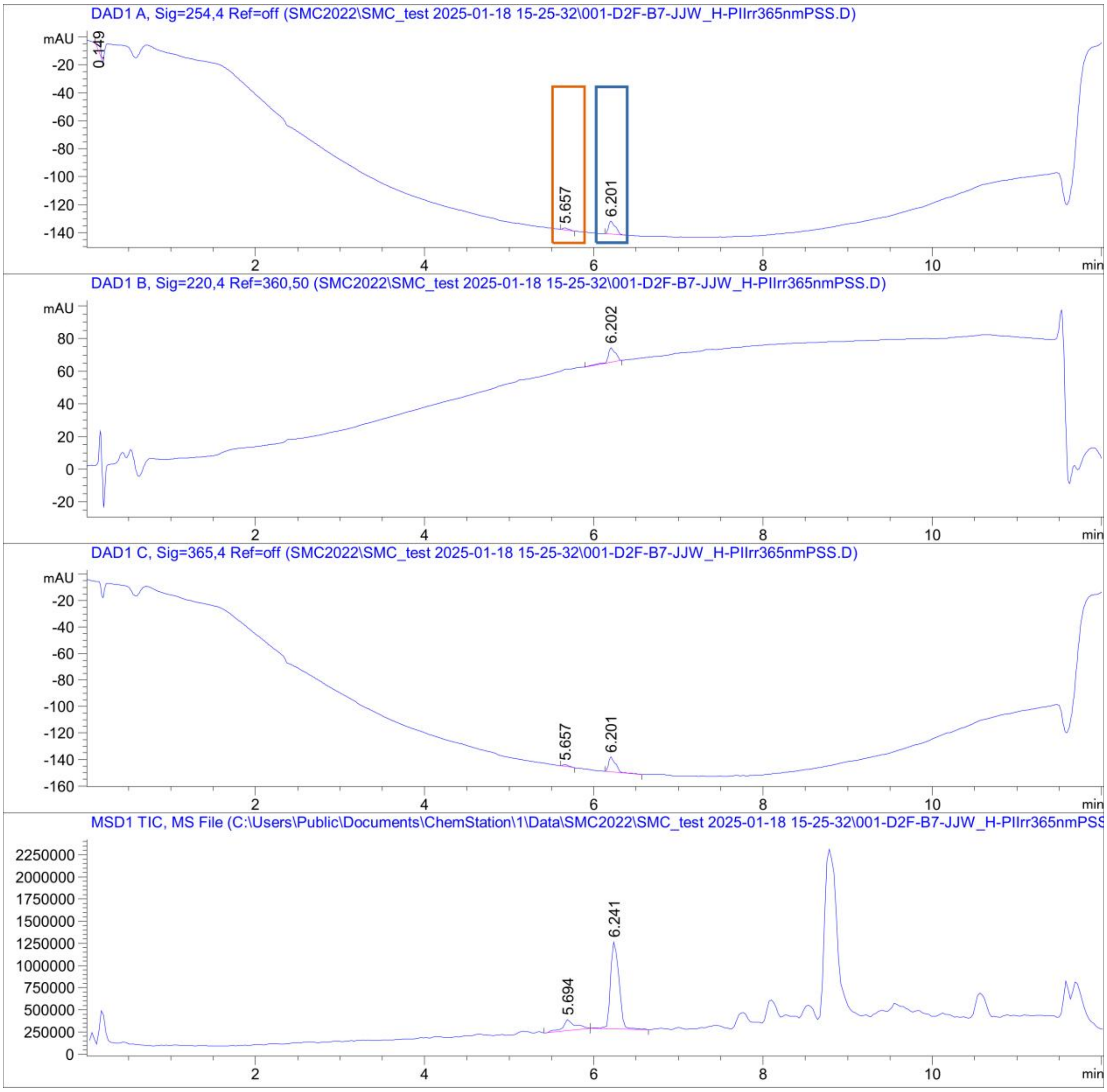


**Figure S102.** The LC-MS spectra of **PI** in MeCN (with 1.5 eq. of *p*TsOH) after irradiation at $\lambda = 365$ nm. The DAD spectra show two peaks belonging to the stable (blue, 6.2 min) and metastable (orange, 5.8 min) isomers of **PI**.

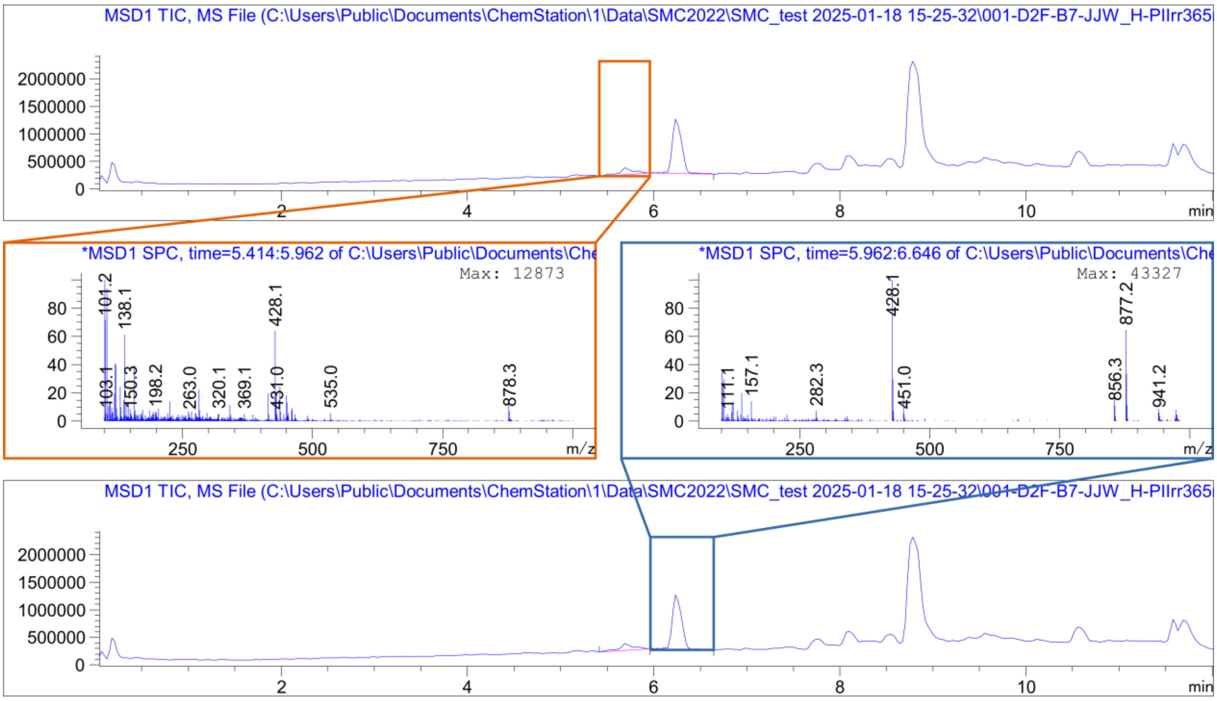


**Figure S103.** The LC-MS spectra of **PI** in MeCN (with 1.5 eq. of *p*TsOH) after irradiation at λ = 365 nm. The MS spectra show the mass for [**PI**+H]$^+$ (428.1), indicating the presence of both isomers. Confirming the retention times belong to the metastable (orange, 5.8 min) and the stable (blue, 6.2 min) isomer of **PI**. The LC-MS was monitored at the isosbestic point (λ = 338 nm), and the area underneath the peaks of the two isomers led to a photostationary distribution of 28% metastable and 72% stable. [(10.6/(10.6+37.8)*100% = 28%]. Resubmission of the same sample leads to a new photostationary distribution of 25% metastable and 75% stable. [(9.99/(9.99+29.84)*100% = 25%].

A comparison of the three PSS determinations by LCMS gives three different values (22%, 28% and 25%, respectively), indicating the difficulty in using LCMS for PSS determination because the values change within the same injected sample and between samples that should reach the same PSS.

## 12.2.3 *In-Situ* NMR Irradiation Sample

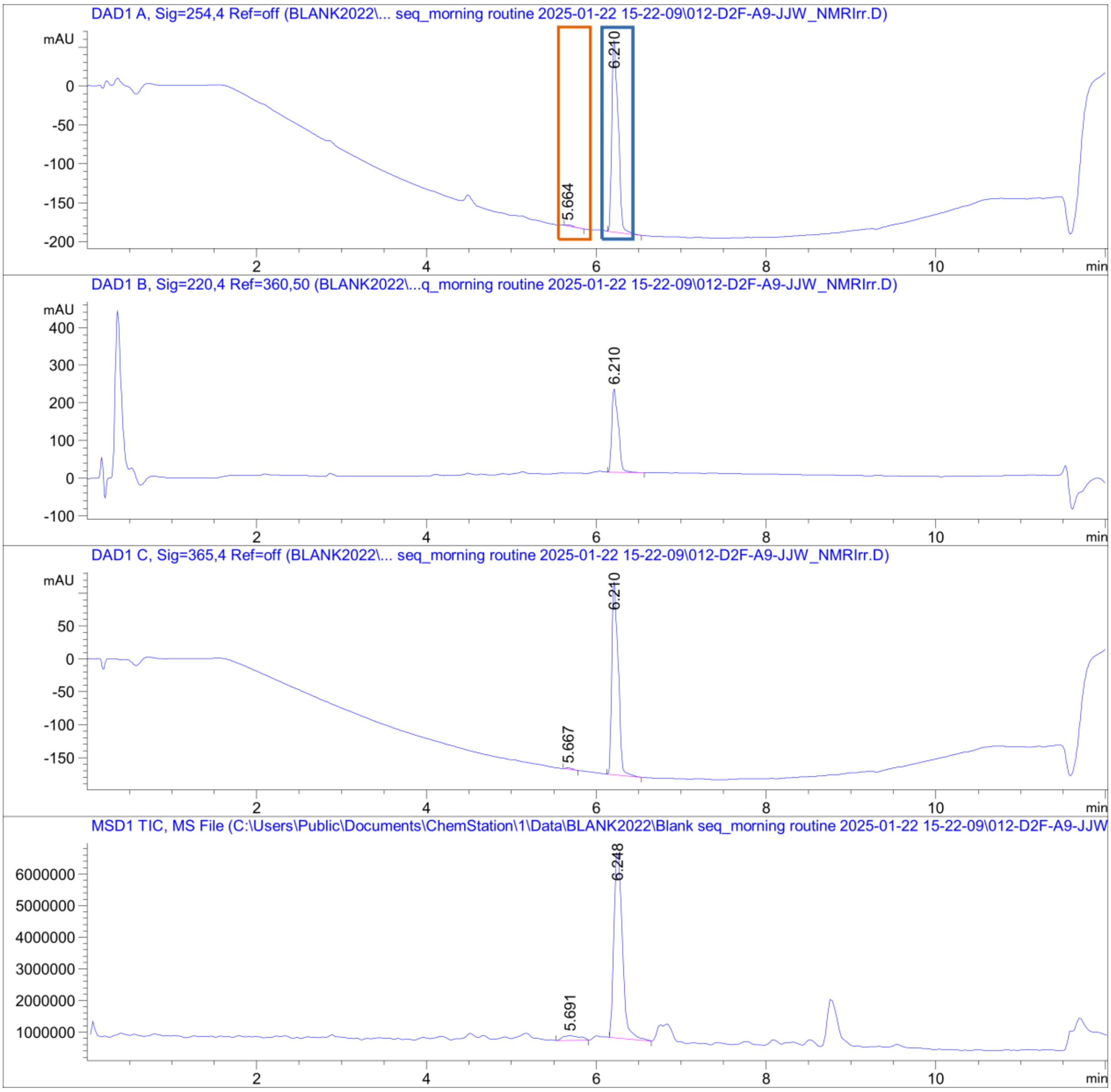


**Figure S104.** The LC-MS spectra of (3 mM) **PI** in MeCN (with 1.5 eq. of *p*TsOH) after *in-situ* NMR irradiation at λ = 365 nm at -25 °C. The DAD spectra show two peaks belonging to the stable (blue, 6.2 min) and metastable (orange, 5.8 min) isomers of **PI**. Showing that at low concentrations, **PI** switches in the NMR.

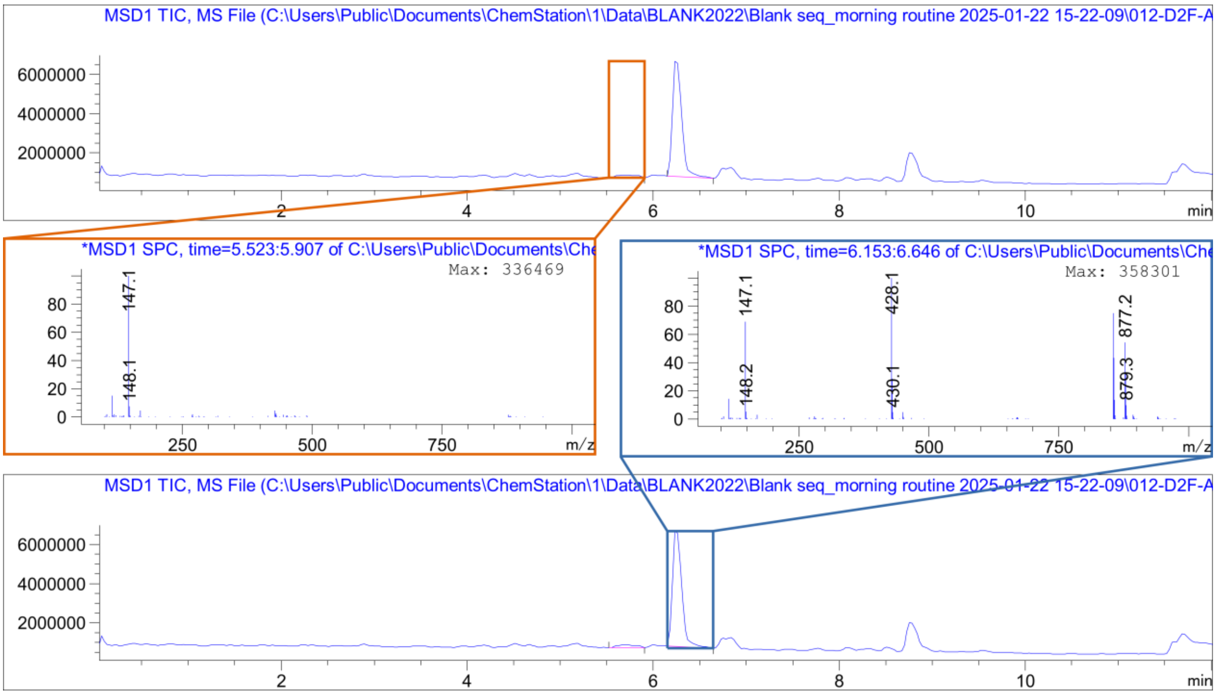


**Figure S105.** The LC-MS spectra of (3 mM) **PI** in MeCN (with 1.5 eq. of *p*TsOH) after *in-situ* NMR irradiation at λ = 365 nm at -25 °C. The MS-spectrum show mass for $[M+H]^+$ (428.1), proving the presence of the stable isomer (blue, 6.2 min). In the MS-spectrum for the metastable isomer, a very minimal mass peak for $[M+H]^+$ (428.1) is seen, proving the presence of the metastable isomer.

## 12.2.4 Pyridine

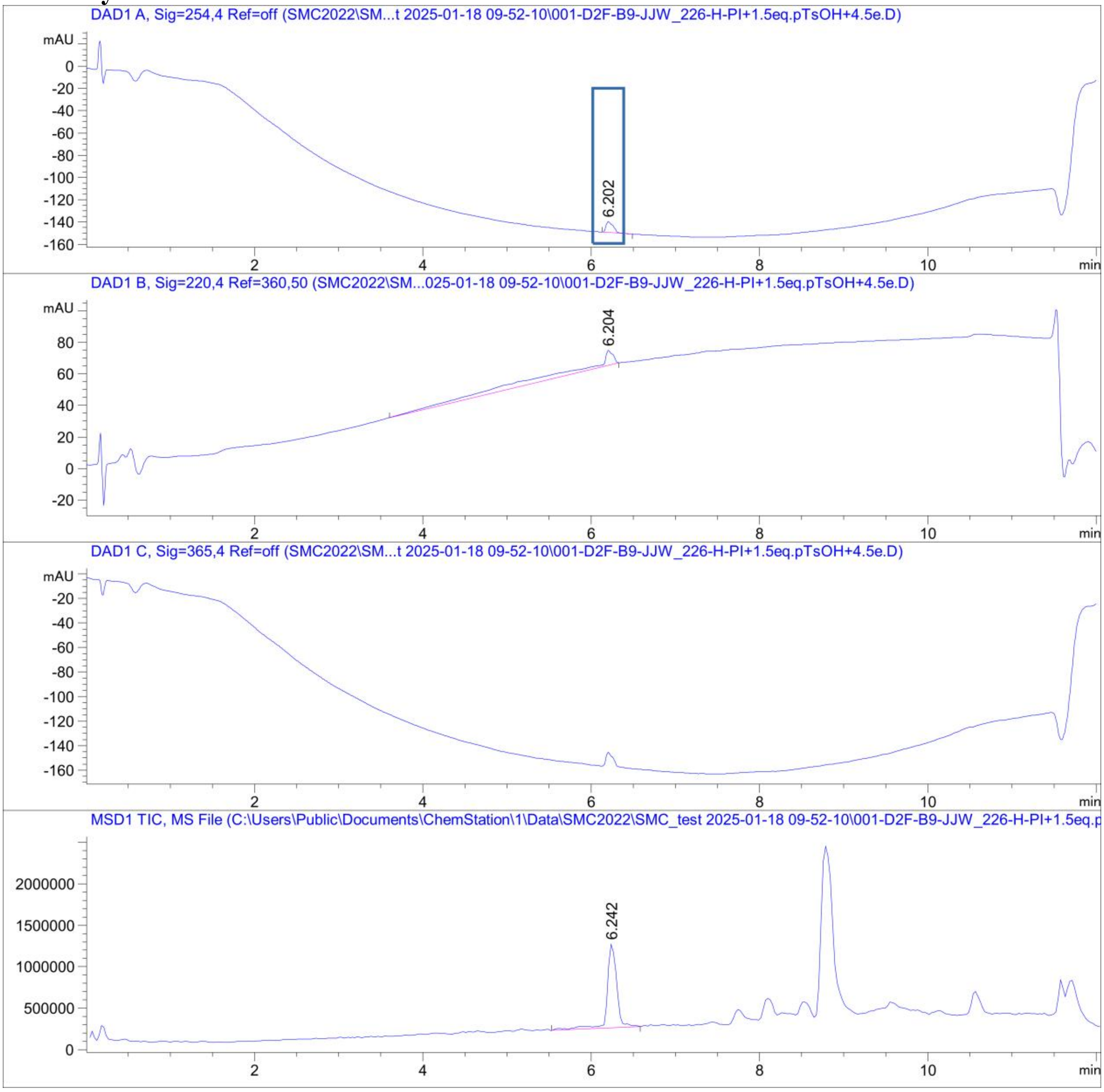


**Figure S106.** The LC-MS spectra of **PI** in MeCN (with 1.5 eq. of *p*TsOH and 4.5 eq. of pyridine) before irradiation. The DAD spectra show one peak belonging to the stable isomer (blue, 6.2 min). (For the use of bases and nucleophiles, see **Section 14**)

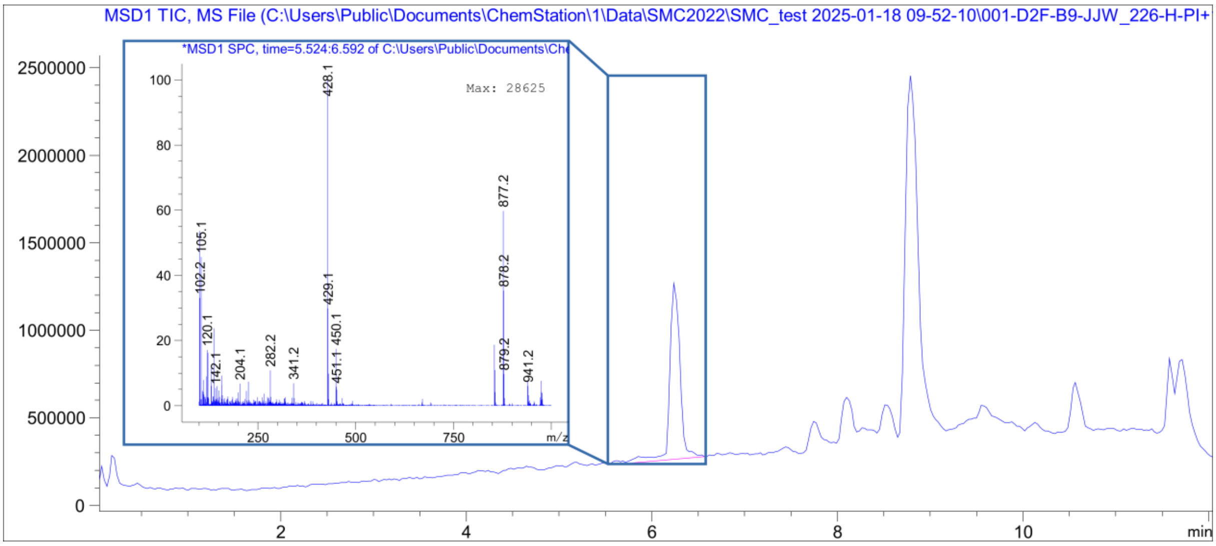


**Figure S107.** The LC-MS spectra of **PI** in MeCN (with 1.5 eq. of *p*TsOH and 4.5 eq. of pyridine) before irradiation. The MS spectra show the mass for [**PI**+H]$^+$ (428.1). Indicating that the pyridine is not covalently bound to **PI**.

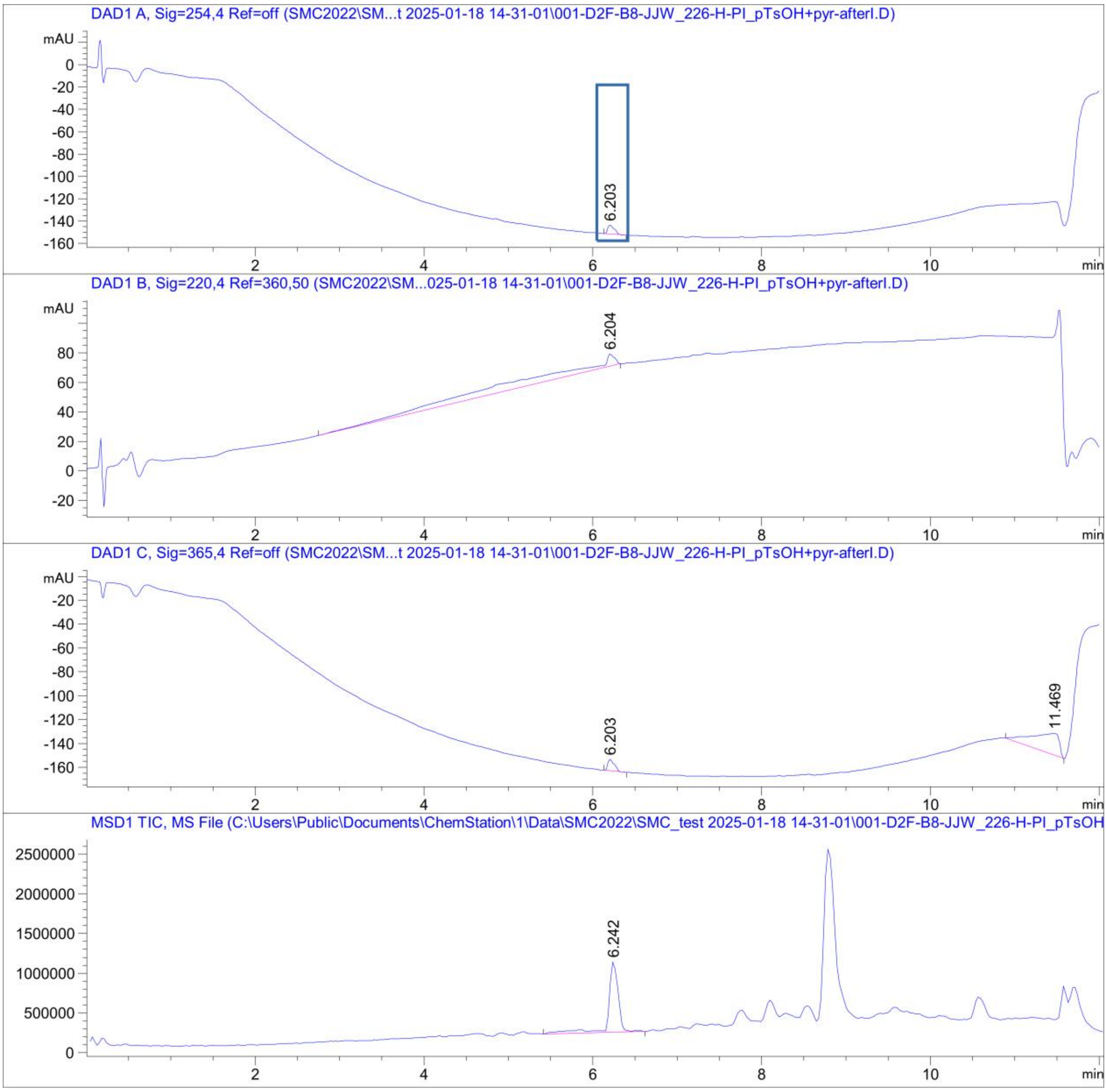


**Figure S108.** The LC-MS spectra of **PI** in MeCN (with 1.5 eq. of *p*TsOH and 4.5 eq. of pyridine) after irradiation at $\lambda$ = 365 nm and back-isomerization at 25 °C. The DAD spectra show one peak belonging to the stable isomer (blue, 6.2 min).

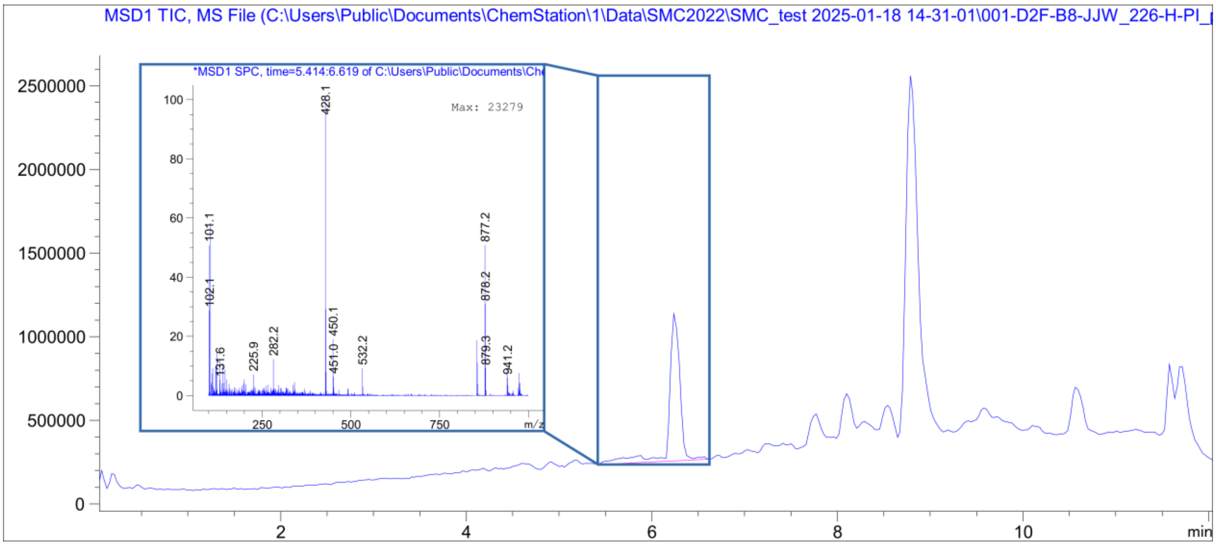


**Figure S109.** The LC-MS spectra of **PI** in MeCN (with 1.5 eq. of *p*TsOH and 4.5 eq. of pyridine) after irradiation at λ = 365 nm and back-isomerization at 25 °C. The MS spectra show the mass for [**PI**+H]$^+$ (428.1). Indicating that the pyridine is not covalently bound to **PI**.

## 13. Quantum Yields

### 13.1 UV-Vis Spectrum Metastable State

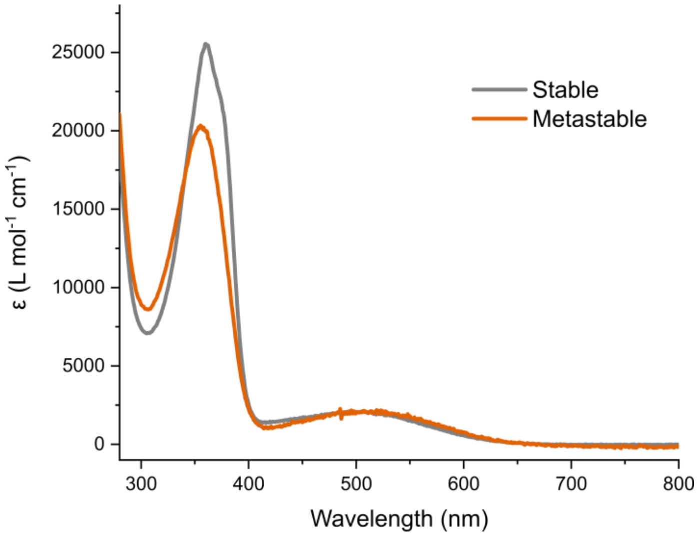


**Figure S110.** The overlapped spectra of the stable (***Z*-**) and metastable (***E*-**) states of **PI**, obtained by constrained non-negative matrix factorization.

### 13.2 Quantum Yields at 365 nm

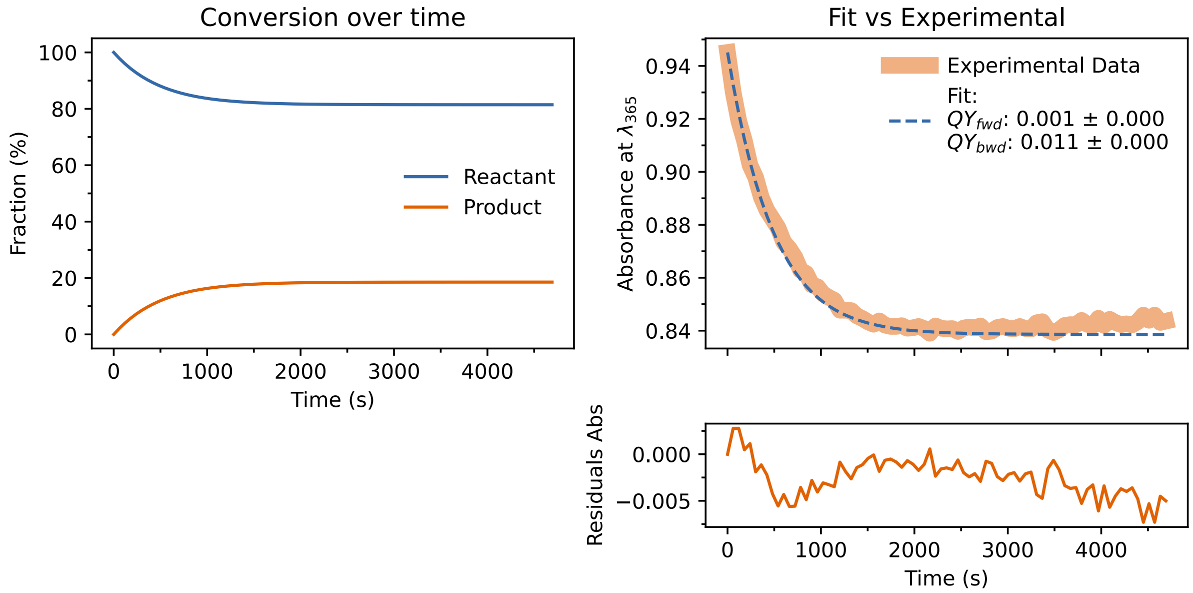


**Figure S111.** Relative concentration over time of *E*- and *Z*-isomers, experimental absorbance at the irradiation wavelength and fit of the integrated absorbances over the emission spectrum of the LED, and residuals of the fit of the irradiation of **PI** in MeCN (1.5 eq. *p*TsOH) with λ = 365 nm. The PSS for determining the spectrum of the metastable state comes from non-negative matrix factorisation, which was used to construct the spectrum of the metastable isomer from the spectrum at the PSS.

### 13.3 Quantum Yields at 395 nm

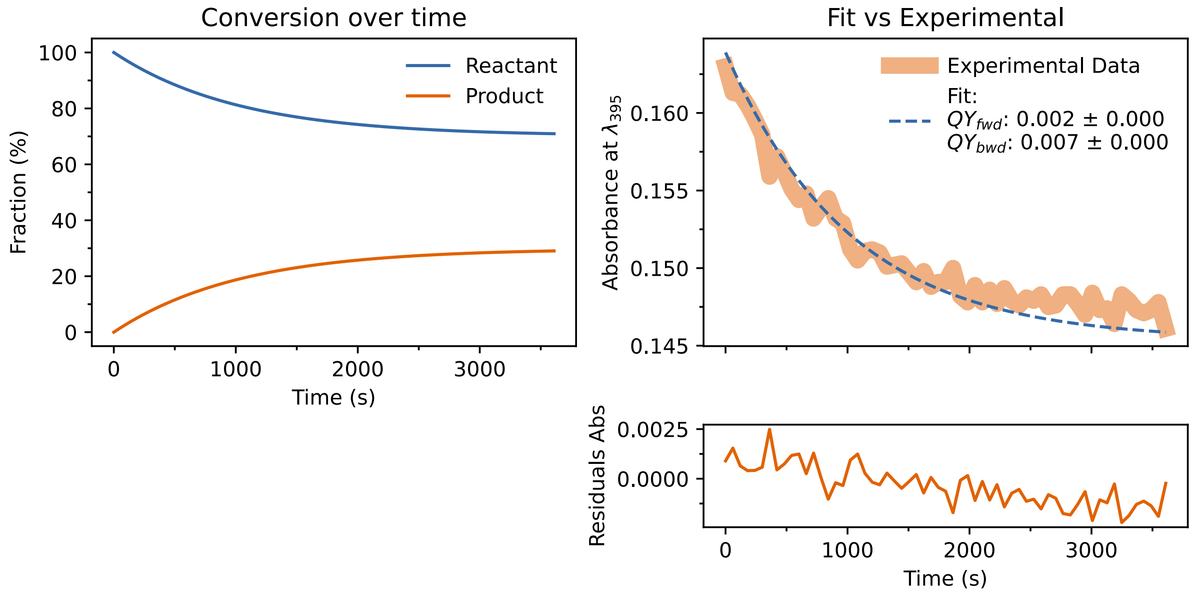


**Figure S112.** Relative concentration over time of *E*- and *Z*-isomers, experimental absorbance at the irradiation wavelength and fit of the integrated absorbances over the emission spectrum of the LED, and residuals of the fit of the irradiation of **PI** in MeCN (1.5 eq. of *p*TsOH) with λ = 395 nm. The PSS for determining the spectrum of the metastable state comes from non-negative matrix factorisation, which was used to construct the spectrum of the metastable isomer from the spectrum at the PSS.

## 14. Back-Isomerization
## 14.1 The Effect of Water

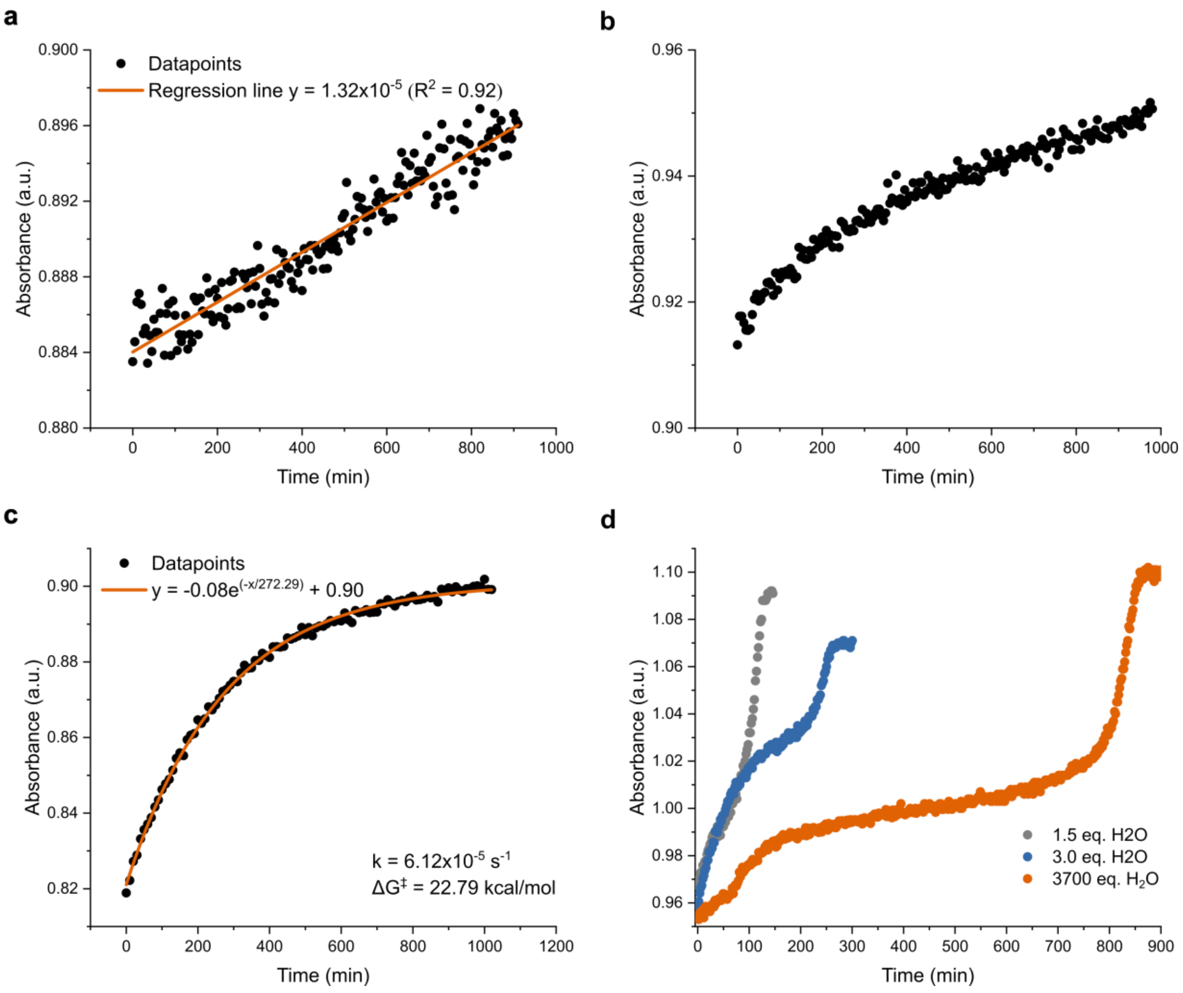


**Figure S113. a** Shows The evolution of the wavelength at λ = 360 nm during the thermal back-isomerization at 25 °C in SPS-grade MeCN (1.5 eq. *p*TsOH); $H_2O$ is provided by the *p*TsOH monohydrate. **b** Shows The evolution of the wavelength at λ = 360 nm during the thermal back-isomerization at 25 °C in SPS-grade MeCN (1.5 eq. *p*TsOH), 1.5 eq. of $H_2O$ is added. **c** Shows the evolution of the absorbance at λ = 360 nm during the thermal back-isomerization at 25 °C in spectroscopical grade MeCN (1.5 eq. pTsOH); $H_2O$ content is unknown, but present in large excess. **d** Shows the spectra for The evolution of the wavelength at λ = 360 nm during the thermal back-isomerization at 25 °C in MeCN (1.5 eq. *p*TsOH) in the presence of $H_2O$: 1.5 eq. (grey), 3.0 eq. (blue), and 3700 eq. (orange).

These spectra show that, depending on the amount of $H_2O$ present in the MeCN, the evolution of the metastable state to the stable state is significantly influenced. Indicating the role water plays in the thermal back-isomerization.

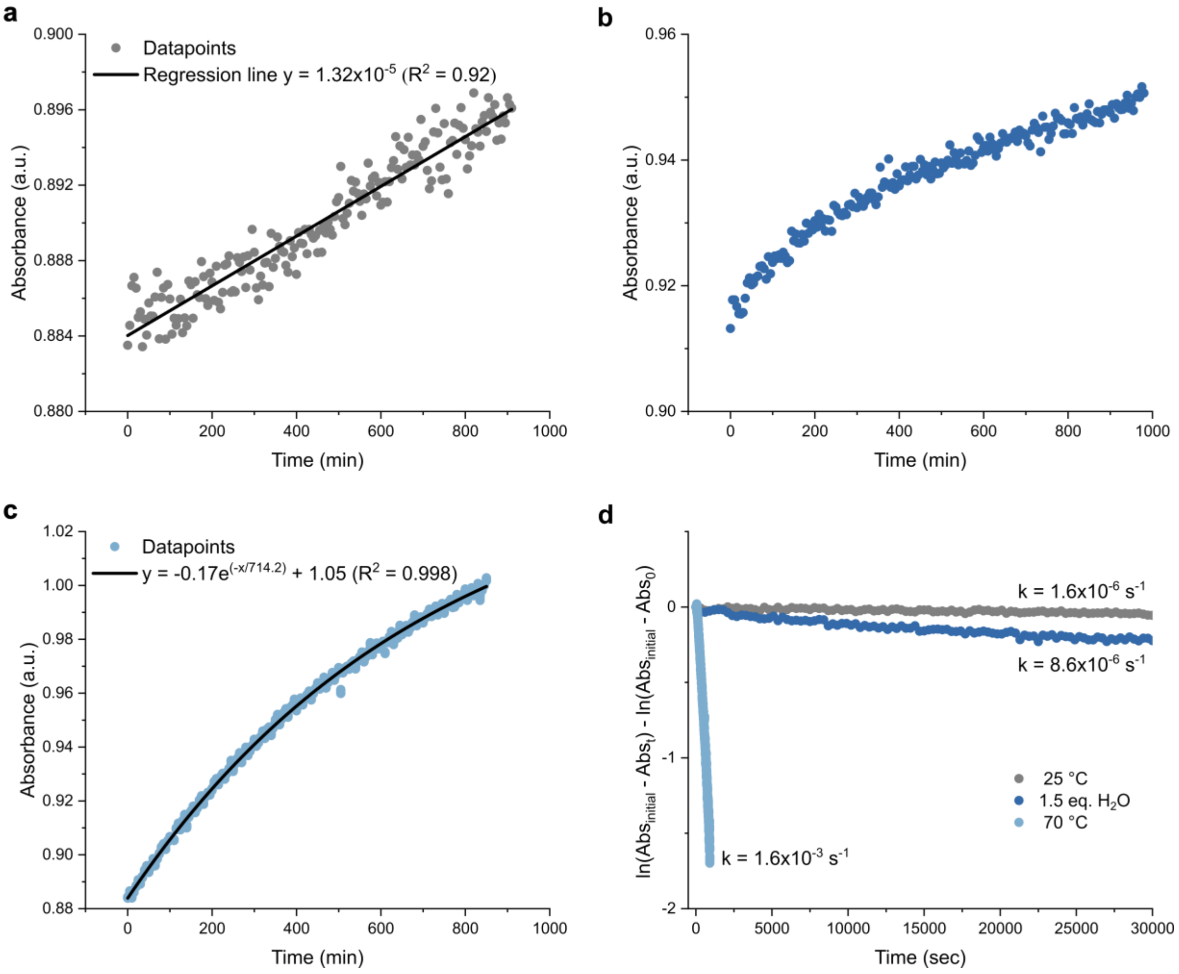


**Figure S114. a** Shows The evolution of the wavelength at λ = 360 nm during the thermal back-isomerization at 25 °C in SPS-grade MeCN. **b** Shows The evolution of the wavelength at λ = 360 nm during the thermal back-isomerization at 25 °C in SPS-grade MeCN. **c** Shows The evolution of the wavelength at λ = 360 nm during the thermal back-isomerization at 70 °C in spectroscopical grade MeCN. **d** Shows the kinetics for **a**, **b** and **c** calculated for the initial rates. Highlighting the effect water has on catalysing the thermal back-isomerization (25 °C: $k = 1.6\text{x}10^{-6}$ $s^{-1}$, 25 °C + 1.5 eq. $H_2O$: $k = 8.6\text{x}10^{-6}$ $s^{-1}$, 70 °C: $k = 1.6\text{x}10^{-3}$ $s^{-1}$).

## 14.2 The Effect of Light

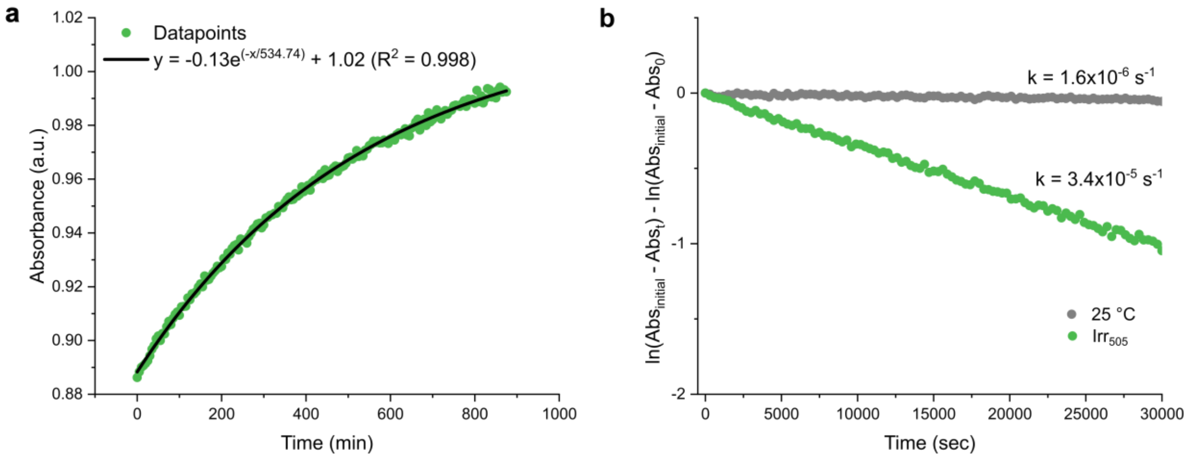


**Figure S115. a** Shows The evolution of the wavelength at $\lambda$ = 360 nm during the back-isomerization irradiated with an LED $\lambda$ = 505 nm at 25 °C in SPS-grade MeCN (1.5 eq. *p*TsOH). **b** Shows the kinetic trace calculated for the initial rate ($k$ = 3.4x10$^{-5}$ s$^{-1}$). Highlighting the photoisomerization from metastable to stable **PI** can be achieved by $Irr_{505}$, when compared to the initial rate at 25 °C (grey).

## 14.3 The Effect of Bases and Nucleophiles

### 14.3.1 Pyridine

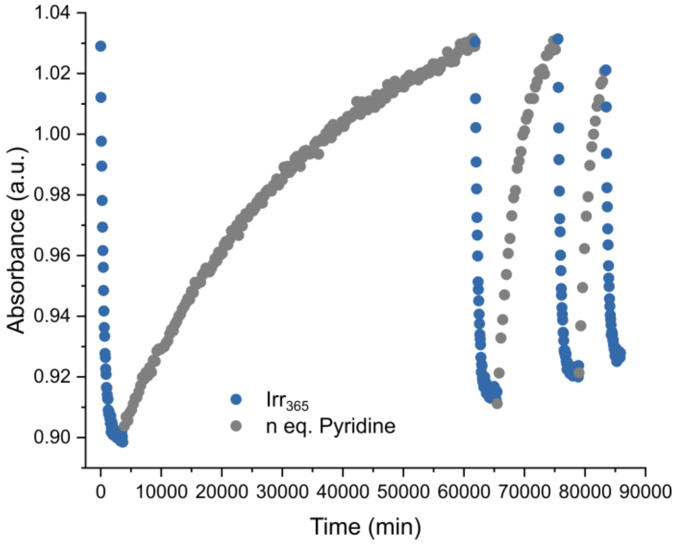


**Figure S116.** Shows the cycle of forward photoisomerization of **PI** with 1.5 eq. *p*TsOH followed by back-isomerization catalysed by increasing equivalents of added pyridine (1.5 eq., 3.0 eq. and 4.5 eq., respectively).

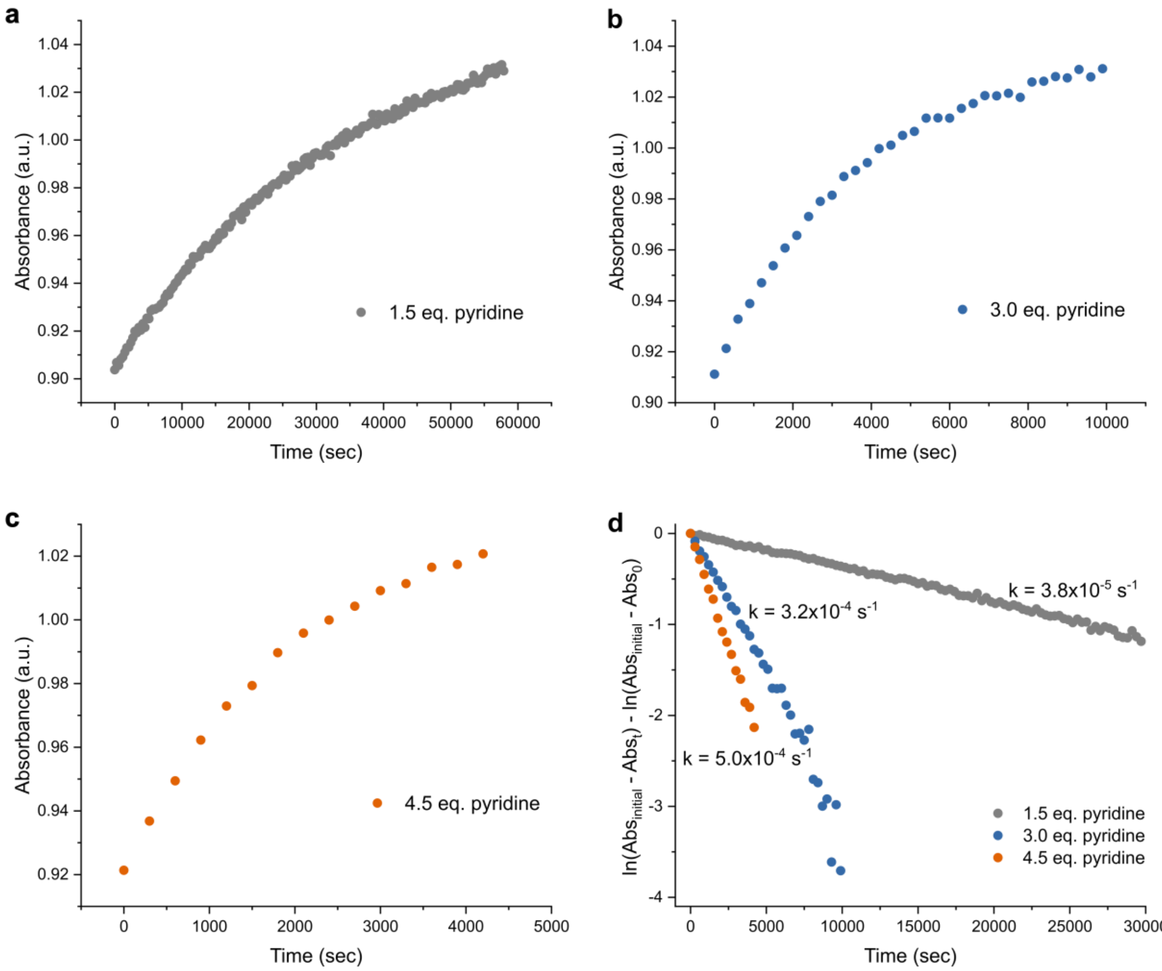


**Figure S117. a** Shows The evolution of the wavelength at λ = 360 nm during the thermal back-isomerization at 25 °C in SPS-grade MeCN (1.5 eq. *p*TsOH) in the presence of 1.5 eq. of pyridine. **b** Shows The evolution of the wavelength at λ = 360 nm during the thermal back-isomerization at 25 °C in SPS-grade MeCN in the presence of 3.0 eq. of pyridine. **c** Shows the evolution of the absorbance at λ = 360 nm during the thermal back-isomerization at 70 °C in spectroscopical grade MeCN in the presence of 1.5 eq. of pyridine. **d** Shows the kinetics for **a**, **b** and **c** calculated for the initial rates. Highlighting the effect pyridine has on catalysing the thermal back-isomerization (1.5 eq. pyridine: $k = 3.8x10^{-5}\ s^{-1}$, 3.0 eq. pyridine: $k = 3.2x10^{-4}\ s^{-1}$, 4.5 eq. pyridine: $k = 5.0x10^{-3}\ s^{-1}$). Highlighting the effect pyridine has on speeding up the thermal back-isomerization and the modulation of the rate by the addition of varying equivalents of pyridine.

### 14.3.2 Caesium Carbonate (Hydroxy Anion)

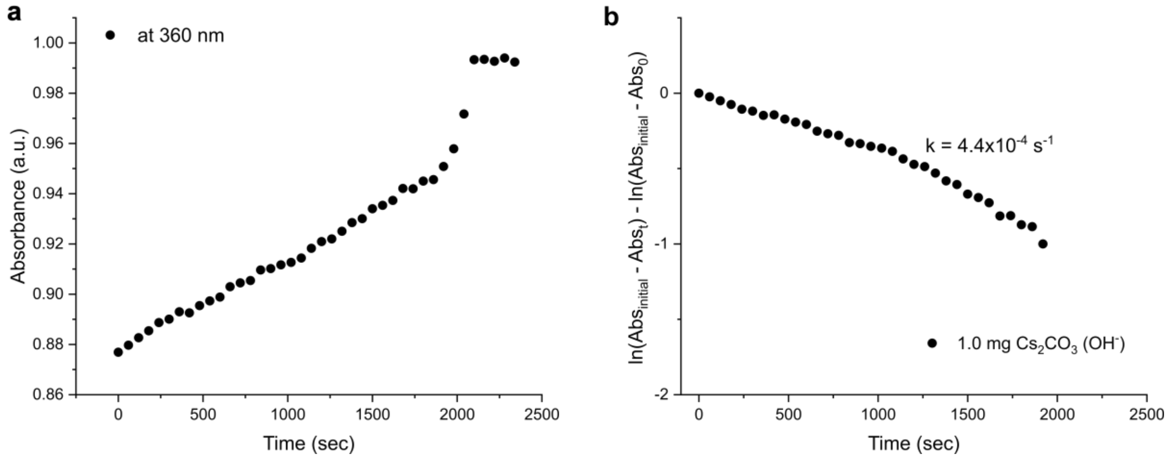


**Figure S118. a** Shows The evolution of the wavelength at λ = 360 nm during the thermal back-isomerization at 25 °C in SPS-grade MeCN (1.5 eq. *p*TsOH) when 1.0 mg caesium carbonate is added to the system. Caesium carbonate does not dissolve in MeCN and will deprotonate the $H_2O$ present in MeCN, rendering the effective nucleophile a hydroxyl anion. Further displaying the role of $H_2O$. The auto-accelerated part of the curve around 2000 seconds is attributed to the influence of $H_2O$ in the system. (Refer to **Section 14.1** for information about the effect of water) **b** Shows the kinetic trace calculated for the initial rate ($k = 4.4x10^{-4}$ $s^{-1}$). Highlighting the effect that caesium carbonate (hydroxy anion) has on catalysing the thermal back-isomerization.

### 14.3.3 Schreiner's Thiourea Catalyst (STC)

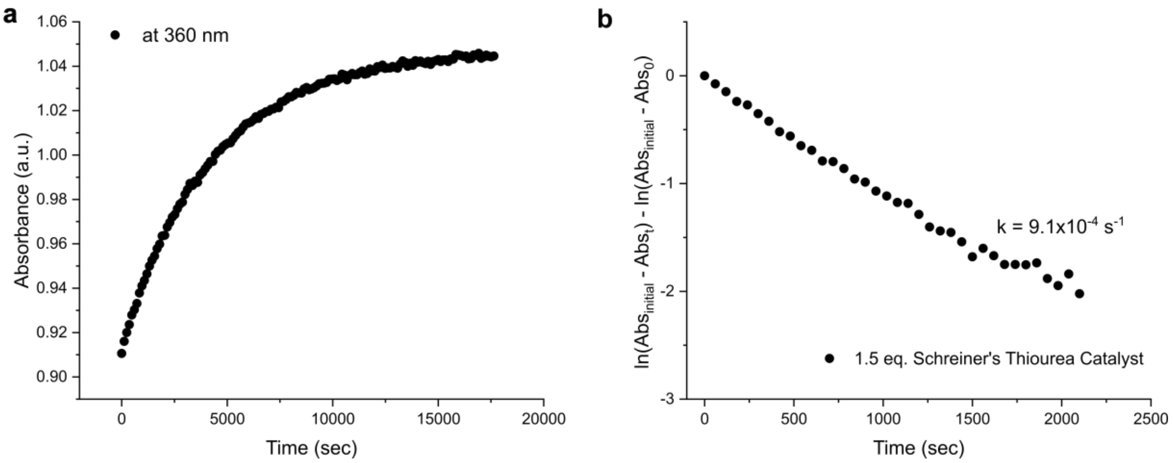


**Figure S119. a** Shows The evolution of the wavelength at λ = 360 nm during the thermal back-isomerization at 25 °C in SPS-grade MeCN (1.5 eq. *p*TsOH) when 1.5 eq. Schreiner's Thiourea Catalyst (STC) is added to the system. STC was added to stabilise the metastable state; it instead acted as a Lewis acid, activating the water present in the system, further highlighting the role of water. **b** Shows the kinetic trace calculated for the initial rate ($k = 9.1x10^{-4}$ $s^{-1}$). Highlighting the effect STC has on catalysing the thermal back-isomerization.

### 14.3.4 1,4-Diazabicyclo[2.2.2]octane (DABCO)

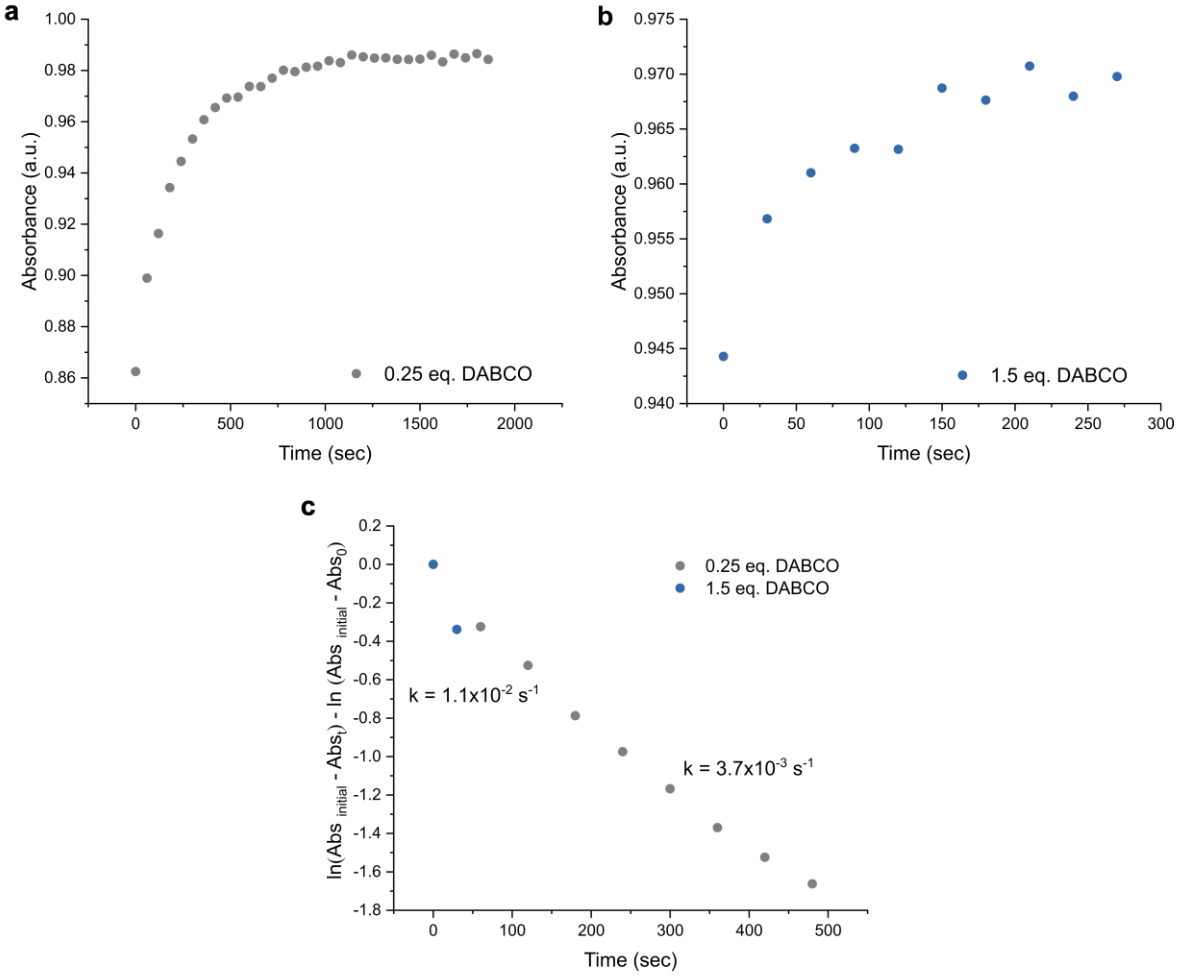


**Figure S120. a** and **b** show the evolution of the absorbance at λ = 360 nm during the thermal back-isomerization at 25 °C in SPS-grade MeCN (1.5 eq. *p*TsOH) when 0.25 and 1.5 eq. of DABCO are added to the system. **c** Shows the kinetic traces calculated for the initial rates (0.25 eq. DABCO: $k = 3.7x10^{-3}$ $s^{-1}$, 1.5 eq. DABCO: $k = 1.1x10^{-2}$ $s^{-1}$). Highlighting the effect DABCO has on catalysing the thermal back-isomerization.

### 14.3.5 Tetrabutylammonium Iodide (TBAI)

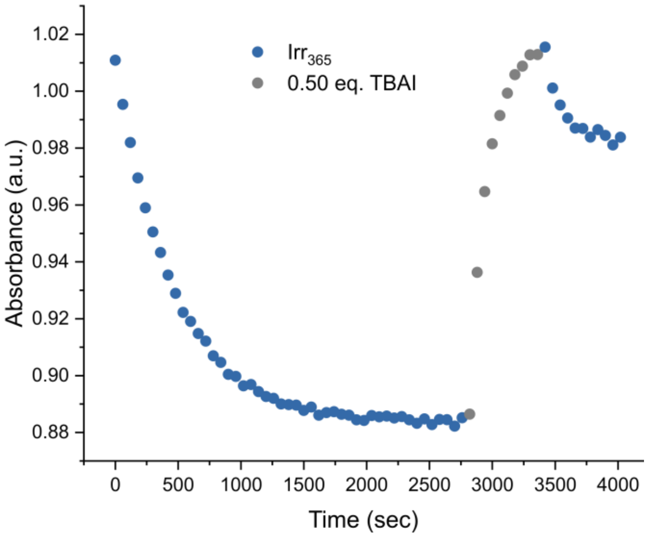


**Figure S121.** Shows the cycle of forward photoisomerization of **PI** with 1.5 eq. *p*TsOH at λ = 365 nm, followed by back-isomerization catalysed by 0.50 eq. of TBAI. And consequent irradiation at λ = 365 nm.

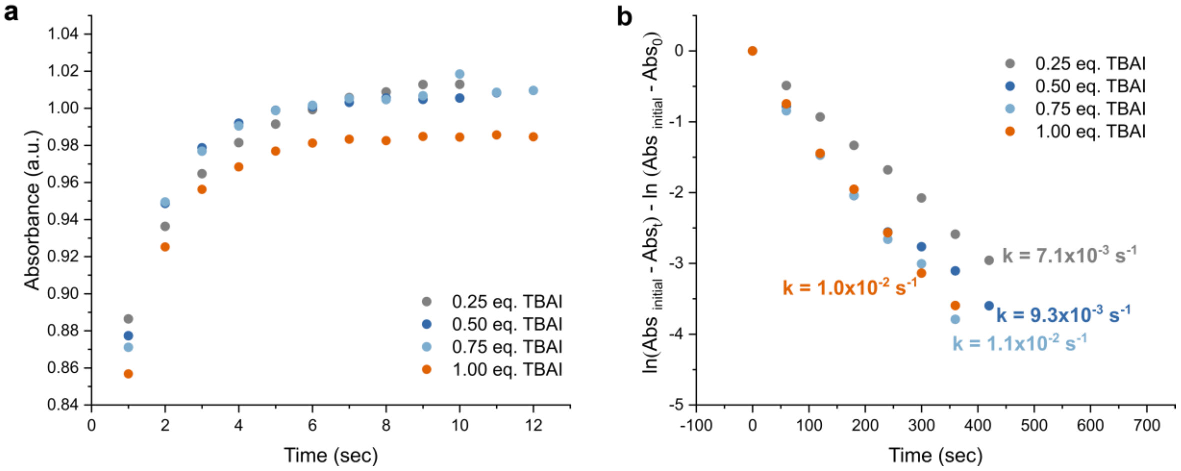


**Figure S122. a** Shows the evolution of the absorbance at λ = 360 nm during the thermal back-isomerization at 25 °C in SPS-grade MeCN (1.5 eq. *p*TsOH) when varying amounts of TBAI are added to the system. **b** Shows the kinetic traces calculated for the initial rates (0.25 eq. TBAI: $k = 7.1\text{x}10^{-3}$ $s^{-1}$, 0.50 eq. TBAI: $k = 9.3\text{x}10^{-3}$ $s^{-1}$, 0.75 eq. TBAI: $k = 1.1\text{x}10^{-2}$ $s^{-1}$, 1.0 eq. TBAI: $k = 1.0\text{x}10^{-2}$ $s^{-1}$). Highlighting the effect TBAI has on catalysing the thermal back-isomerization and the modulation of the rate by the addition of varying equivalents of TBAI. TBAI is solely a nucleophile (in comparison to the other compounds added, which are also bases), showcasing that the thermal back-isomerization is catalysed by nucleophiles.

## 15. Cycles with Pyridine

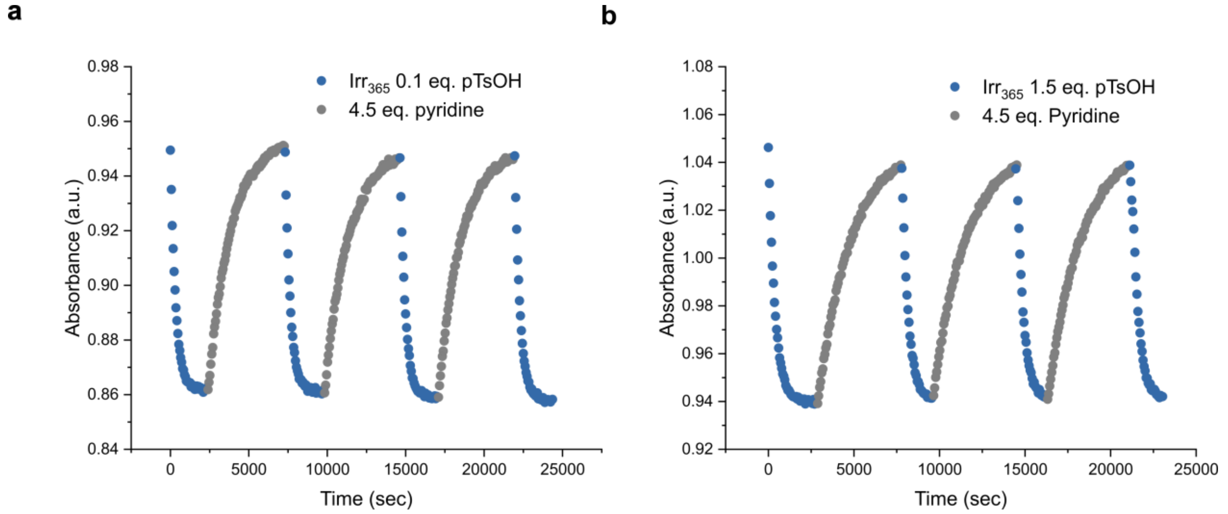


**Figure S123. a** Shows the three complete cycles of forward isomerization of the system including **PI**, 1.5 eq. of *p*TsOH and 4.5 eq. of pyridine. This indicates that the system can be switched forward and backwards continuously without noticeable fatigue. **b** Shows the three complete cycles of forward isomerization of the system, including **PI**, 0.1 eq. of *p*TsOH and 4.5 eq. of pyridine. This indicates that the system can be switched forward and backwards continuously without noticeable fatigue, even in a system with a catalytic amount of acid present.

## 16. Cycles with TBAI

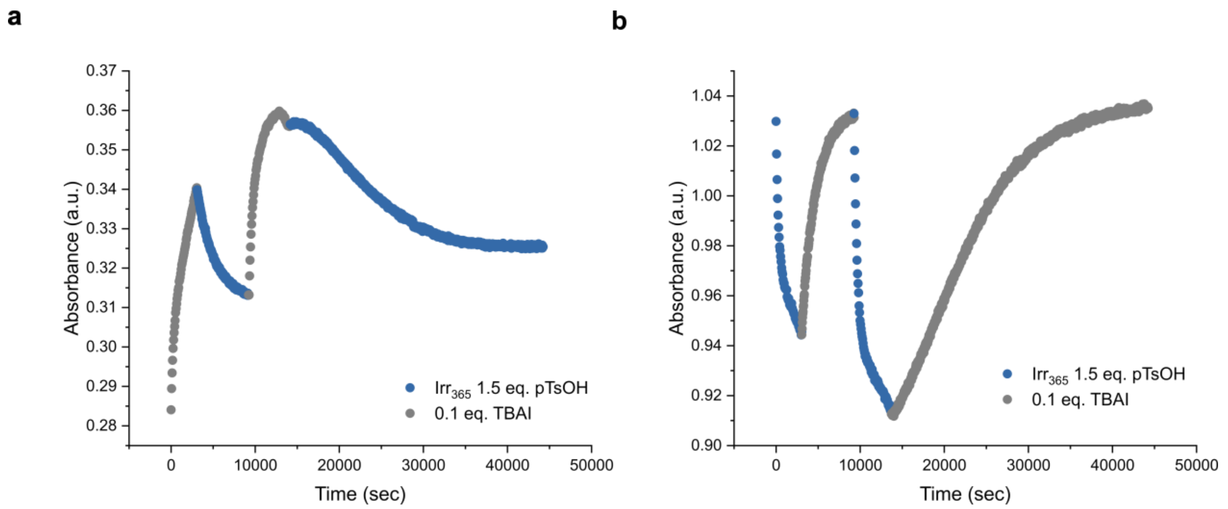


**Figure S124. a** and **b** show two complete cycles of forward isomerization of the system including **PI**, 1.5 eq. of *p*TsOH and 0.1 eq. of TBAI monitored at either side of the isosbestic point (λ = 305 and 360 nm, respectively). The data indicates that the combination of TBAI, acid and light is not innocent, a PSS is not reached, and it seems that the system is undergoing chemical reactions. Whilst in the λ = 360 nm trace, it seems that the molecule is fully recovering, the trace at λ = 305 nm shows a different story.

## 17. Electrochemistry

There are two main reduction processes observable in the cyclic voltammogram of **PI** at -0.40 V and at -1.0 V. At a higher scan rate of 1.0 V $s^{-1}$, it becomes clear that a new species with an oxidation at -0.10 V is generated upon the reduction of **PI**.

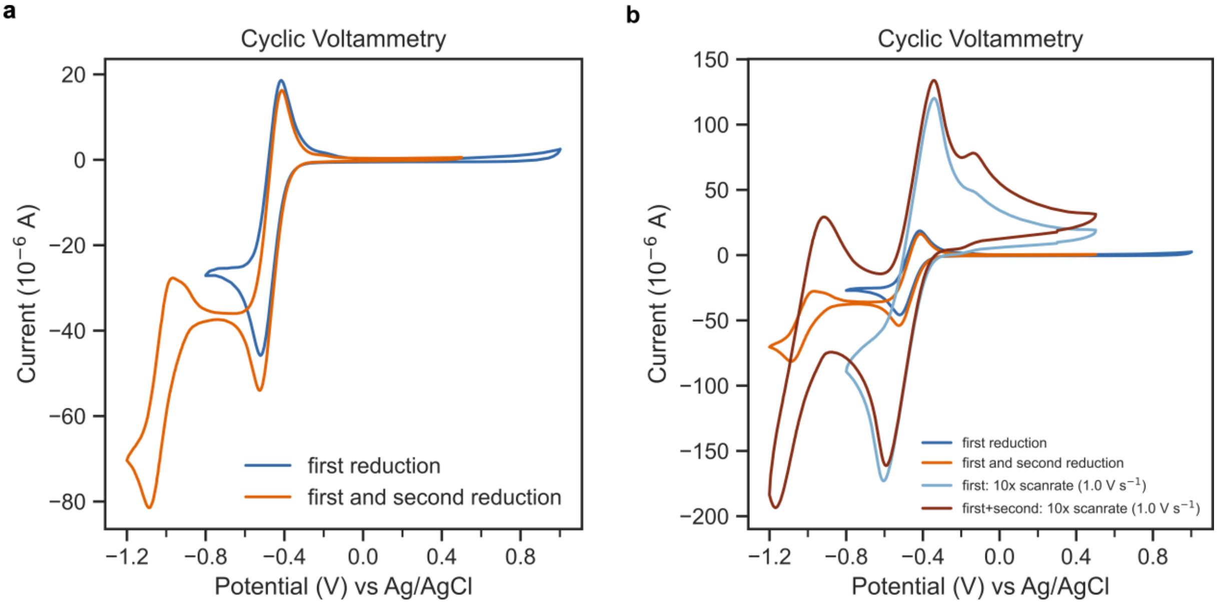


**Figure S125. a** Cyclic voltammetry (3rd cycles are shown) of **PI** (1 mM) in acetonitrile (0.1 M $NBu_4PF_6$) at 0.1 V $s^{-1}$. **b** Cyclic voltammetry (3rd cycles are shown) of **PI** (1 mM) in acetonitrile (0.1 M $NBu_4PF_6$) at 0.1 V $s^{-1}$ (blue, orange) and 1.0 V $s^{-1}$ (light blue, maroon).

## 19. References


[1] A. Volker, J. D. Steen, S. Crespi, “A fiber-optic spectroscopic setup for isomerization quantum yield determination” *Beilstein J. Org. Chem.* **2024**, *20*, 1684–1692.

[2] "autoQY," can be found under https://github.com/CrespiLab/autoQY, **2025**.

[3] P. M. Maffettone, A. C. Daly, D. Olds, “Constrained non-negative matrix factorization enabling real-time insights of in situ and high-throughput experiments” *Appl. Phys. Rev.* **2021**, *8*, 041410.

[4] NSLS2, “Release v0.1.2 NSLS2/constrained-matrix-factorization,” can be found under https://github.com/NSLS2/constrained-matrix-factorization/releases/tag/v0.1.2(accessed 6 August 2025)

[5] Bruker APEX3 V20191-0 St. Version 840A SADABS Version 20161 Bruker AXS Inc Madison Wis. USA.

[6] G. M. Sheldrick, “A short history of SHELX” *Acta Crystallogr. A* **2008**, *64*, 112–122.

[7] O. V. Dolomanov, L. J. Bourhis, R. J. Gildea, J. a. K. Howard, H. Puschmann, “OLEX2: a complete structure solution, refinement and analysis program” *J. Appl. Crystallogr.* **2009**, *42*, 339–341.

[8] J. J. Snellenburg, S. Laptenok, R. Seger, K. M. Mullen, I. H. M. van Stokkum, “Glotaran: A Java-Based Graphical User Interface for the R Package TIMP” *J. Stat. Softw.* **2012**, *49*, 1–22.

[9] C. Bannwarth, S. Ehlert, S. Grimme, “GFN2-xTB—An Accurate and Broadly Parametrized Self-Consistent Tight-Binding Quantum Chemical Method with Multipole Electrostatics and Density-Dependent Dispersion Contributions” *J. Chem. Theory Comput.* **2019**, *15*, 1652–1671.

[10] C. Bannwarth, E. Caldeweyher, S. Ehlert, A. Hansen, P. Pracht, J. Seibert, S. Spicher, S. Grimme, “Extended tight-binding quantum chemistry methods” *WIREs Comput. Mol. Sci.* **2021**, *11*, e1493.

[11] F. Neese, F. Wennmohs, U. Becker, C. Riplinger, “The ORCA quantum chemistry program package” *J. Chem. Phys.* **2020**, *152*, 224108.

[12] F. Neese, “Software update: The ORCA program system—Version 5.0” *WIREs Comput. Mol. Sci.* **2022**, *12*, e1606.

[13] J. Steinmetzer, S. Kupfer, S. Gräfe, “pysisyphus: Exploring potential energy surfaces in ground and excited states” *Int. J. Quantum Chem.* **2021**, *121*, e26390.

[14] S. Grimme, A. Hansen, S. Ehlert, J.-M. Mewes, “r2SCAN-3c: A ‘Swiss army knife’ composite electronic-structure method” *J. Chem. Phys.* **2021**, *154*, 064103.

[15] F. Neese, “Definition of corresponding orbitals and the diradical character in broken symmetry DFT calculations on spin coupled systems” *J. Phys. Chem. Solids* **2004**, *65*, 781–785.

[16] J.-D. Chai, M. Head-Gordon, “Systematic optimization of long-range corrected hybrid density functionals” *J. Chem. Phys.* **2008**, *128*, 084106.

[17] J.-D. Chai, M. Head-Gordon, “Long-range corrected hybrid density functionals with damped atom–atom dispersion corrections” *Phys. Chem. Chem. Phys.* **2008**, *10*, 6615–6620.

[18] O. A. Vydrov, T. Van Voorhis, “Nonlocal van der Waals density functional: the simpler the better” *J. Chem. Phys.* **2010**, *133*, 244103.

[19] F. Weigend, R. Ahlrichs, “Balanced basis sets of split valence, triple zeta valence and quadruple zeta valence quality for H to Rn: Design and assessment of accuracy” *Phys. Chem. Chem. Phys.* **2005**, *7*, 3297–3305.

[20] H. Kruse, S. Grimme, “A geometrical correction for the inter- and intra-molecular basis set superposition error in Hartree-Fock and density functional theory calculations for large systems” *J. Chem. Phys.* **2012**, *136*, 154101.

[21] V. Barone, M. Cossi, “Quantum Calculation of Molecular Energies and Energy Gradients in Solution by a Conductor Solvent Model” *J. Phys. Chem. A* **1998**, *102*, 1995–2001.
[22] Y. Zhao, D. G. Truhlar, “The M06 suite of density functionals for main group thermochemistry, thermochemical kinetics, noncovalent interactions, excited states, and transition elements: two new functionals and systematic testing of four M06-class functionals and 12 other functionals” *Theor. Chem. Acc.* **2008**, *120*, 215–241.
[23] F. Weigend, “Accurate Coulomb-fitting basis sets for H to Rn” *Phys. Chem. Chem. Phys.* **2006**, *8*, 1057–1065.
[24] A. D. Becke, “A new mixing of Hartree–Fock and local density-functional theories” *J. Chem. Phys.* **1993**, *98*, 1372–1377.
[25] A. V. Marenich, C. J. Cramer, D. G. Truhlar, “Universal Solvation Model Based on Solute Electron Density and on a Continuum Model of the Solvent Defined by the Bulk Dielectric Constant and Atomic Surface Tensions” *J. Phys. Chem. B* **2009**, *113*, 6378–6396.
[26] V. Mironov, K. Komarov, J. Li, I. Gerasimov, H. Nakata, M. Mazaherifar, K. Ishimura, W. Park, A. Lashkaripour, M. Oh, M. Huix-Rotllant, S. Lee, C. H. Choi, “OpenQP: A Quantum Chemical Platform Featuring MRSF-TDDFT with an Emphasis on Open-Source Ecosystem” *J. Chem. Theory Comput.* **2024**, *20*, 9464–9477.
[27] W. J. Hehre, R. Ditchfield, J. A. Pople, “Self—Consistent Molecular Orbital Methods. XII. Further Extensions of Gaussian—Type Basis Sets for Use in Molecular Orbital Studies of Organic Molecules” *J. Chem. Phys.* **1972**, *56*, 2257–2261.
[28] P. C. Hariharan, J. A. Pople, “The influence of polarization functions on molecular orbital hydrogenation energies” *Theor. Chim. Acta* **1973**, *28*, 213–222.
[29] R. Ditchfield, W. J. Hehre, J. A. Pople, “Self-Consistent Molecular-Orbital Methods. IX. An Extended Gaussian-Type Basis for Molecular-Orbital Studies of Organic Molecules” *J. Chem. Phys.* **1971**, *54*, 724–728.
[30] A. D. McLean, G. S. Chandler, “Contracted Gaussian basis sets for molecular calculations. I. Second row atoms, $Z$ =11–18” *J. Chem. Phys.* **1980**, *72*, 5639–5648.
[31] M. M. Francl, W. J. Pietro, W. J. Hehre, J. S. Binkley, M. S. Gordon, D. J. DeFrees, J. A. Pople, “Self-consistent molecular orbital methods. XXIII. A polarization-type basis set for second-row elements” *J. Chem. Phys.* **1982**, *77*, 3654–3665.
[32] K. L. Schuchardt, B. T. Didier, T. Elsethagen, L. Sun, V. Gurumoorthi, J. Chase, J. Li, T. L. Windus, “Basis Set Exchange: A Community Database for Computational Sciences” *J. Chem. Inf. Model.* **2007**, *47*, 1045–1052.
[33] D. Feller, “The role of databases in support of computational chemistry calculations” *J. Comput. Chem.* **1996**, *17*, 1571–1586.
[34] B. P. Pritchard, D. Altarawy, B. Didier, T. D. Gibson, T. L. Windus, “New Basis Set Exchange: An Open, Up-to-Date Resource for the Molecular Sciences Community” *J. Chem. Inf. Model.* **2019**, *59*, 4814–4820.
[35] F. Neese, “The ORCA program system” *WIREs Comput. Mol. Sci.* **2012**, *2*, 73–78.
[36] F. Neese, “Software update: the ORCA program system, version 4.0” *WIREs Comput. Mol. Sci.* **2018**, *8*, e1327.
[37] F. Neese, “The SHARK integral generation and digestion system” *J. Comput. Chem.* **2023**, *44*, 381–396.
[38] https://github.com/CrespiLab/ExtOptORCA-OpenQP
[39] P. Pracht, S. Grimme, C. Bannwarth, F. Bohle, S. Ehlert, G. Feldmann, J. Gorges, M. Müller, T. Neudecker, C. Plett, S. Spicher, P. Steinbach, P. A. Wesołowski, F. Zeller, “CREST—A program for the exploration of low-energy molecular chemical space” *J. Chem. Phys.* **2024**, *160*, 114110.

[40] P. Pracht, C. Bannwarth, “Finding Excited-State Minimum Energy Crossing Points on a Budget: Non-Self-Consistent Tight-Binding Methods” *J. Phys. Chem. Lett.* **2023**, *14*, 4440–4448.
[41] S. Spicher, C. Plett, P. Pracht, A. Hansen, S. Grimme, “Automated Molecular Cluster Growing for Explicit Solvation by Efficient Force Field and Tight Binding Methods” *J. Chem. Theory Comput.* **2022**, *18*, 3174–3189.
[42] Y. Li **2022**, DOI 10.5281/zenodo.7187658.
[43] T. Lu, F. Chen, “Multiwfn: A multifunctional wavefunction analyzer” *J. Comput. Chem.* **2012**, *33*, 580–592.
[44] T. Lu, “A comprehensive electron wavefunction analysis toolbox for chemists, Multiwfn” *J. Chem. Phys.* **2024**, *161*, 082503.
[45] A. Chelouan, R. Recio, E. Álvarez, N. Khiar, I. Fernández, “Stereoselective Synthesis of P-Stereogenic N-Phosphinyl Compounds” *Eur. J. Org. Chem.* **2016**, *2016*, 255–259.
[46] H. Adams, R. C. Collins, S. Jones, C. J. A. Warner, “Enantioselective Preparation of P-Chiral Phosphine Oxides” *Org. Lett.* **2011**, *13*, 6576–6579.
[47] S. Crespi, N. A. Simeth, M. Di Donato, S. Doria, C. N. Stindt, M. F. Hilbers, F. L. Kiss, R. Toyoda, S. Wesseling, W. J. Buma, B. L. Feringa, W. Szymański, “Phenylimino Indolinone: A Green-Light-Responsive T-Type Photoswitch Exhibiting Negative Photochromism” *Angew. Chem. Int. Ed.* **2021**, *60*, 25290–25295.
[48] W. Hu, E.-Q. Li, Z. Duan, F. Mathey, “Concise Synthesis of Phospholene and Its P-Stereogenic Derivatives” *J. Org. Chem.* **2020**, *85*, 14772–14778.
[49] S. Thumser, L. Köttner, N. Hoffmann, P. Mayer, H. Dube, “All-Red-Light Photoswitching of Indirubin Controlled by Supramolecular Interactions” *J. Am. Chem. Soc.* **2021**, *143*, 18251–18260.
[50] A. Kütt, S. Tshepelevitsh, J. Saame, M. Lõkov, I. Kaljurand, S. Selberg, I. Leito, “Strengths of Acids in Acetonitrile” *Eur. J. Org. Chem.* **2021**, *2021*, 1407–1419.
[51] E. Raamat, K. Kaupmees, G. Ovsjannikov, A. Trummal, A. Kütt, J. Saame, I. Koppel, I. Kaljurand, L. Lipping, T. Rodima, V. Pihl, I. A. Koppel, I. Leito, “Acidities of strong neutral Brønsted acids in different media” *J. Phys. Org. Chem.* **2013**, *26*, 162–170.
[52] E. I. Matrosov, E. N. Tsvetkov, Z. N. Mironova, R. A. Malevannaya, M. I. Kabachnik, “Acid-base properties of phosphine oxides in nitromethane” *Bull. Acad. Sci. USSR Div. Chem. Sci.* **1975**, *24*, 1231–1234.
